# Supplementary material for: Visible Light Promotes Cascade Trifluoromethylation/Cyclization, Leading to Trifluoromethylated Polycyclic Quinazolinones, Benzimidazoles and Indoles
Source: Molecules. 2022 Dec 1;27(23):8389. doi: 10.3390/molecules27238389 (PMC9737949; doi:10.3390/molecules27238389)

Supporting Information

Visible Light Promotes Cascade Trifluoromethylation/Cyclization, Leading to Trifluoromethylated Polycyclic Quinazolinones, Benzimidazoles and Indoles

Ransong Ma[1], Yuanyuan Ren[1], Zhoubin Deng[1], Ke-Hu Wang[1], Junjiao Wang[1], Danfeng Huang[1], Yulai Hu[1,2]* and Xiaobo Lv[3]

[1]*College of Chemistry and Chemical Engineering, Northwest Normal University, Lanzhou 730070, P. R. China.*
[2]*State Key Laboratory of Applied Organic Chemistry, Lanzhou University, Lanzhou 730000, P. R. China.*
[3]*Shanghai Sinofluoro Chemicals Co., Ltd., Shanghai 201321, P. R. China.*
Email: huyl@nwnu.edu.cn;

Table of contents

1. General information S2
2.Synthesis of alkenyl-substituted substrates **1**, **4** and **6** S2
3. General procedure for the synthesis of products **3**, **5** and **7** S2
4. Procedure for the gram-scale synthesis of **3a** S3
5. Radical trapping experiment S3
6. Characterization data of products S4
7. Reference S29
8. The ^{1}H, ^{13}C and ^{19}F NMR spectra copy of compounds S30

1. General information

The solvents were distilled by standard methods. Reagents were obtained from commercial suppliers and used without further purification unless otherwise noted. Silica gel column chromatography was carried out using silica gel 60 (230−400 mesh). Analytical thin-layer chromatography (TLC) was done using silica gel (silica gel 60 F254). TLC plates were analyzed by exposure to ultraviolet (UV) light and/or submersion in phosphomolybdic acid solution or submersion in $KMnO_4$ solution or iodine vapor. NMR experiments were carried out in deuterated chloroform ($CDCl_3$). ^{1}H NMR, ^{13}C{^{1}H} NMR, and ^{19}F NMR spectra were recorded at 400 or 600 MHz, 100 or 150 MHz, and 376 MHz spectrometers, respectively. Chemical shifts are reported as δ values relative to internal TMS (δ 0.00 ppm for ^{1}H NMR), chloroform (δ 7.26 ppm for ^{1}H NMR), chloroform (δ 77.00 ppm for ^{13}C{^{1}H} NMR) in parts per million (ppm). The following abbreviations are used for the multiplicities: s, singlet; d, doublet; dd, doublet of doublet; t, triplet; q, quartet; and m, multiplet. Coupling constants (*J*) are reported in hertz (Hz). High-resolution mass spectra (HRMS) were recorded on a Micro TOF-QII mass instrument (ESI).

2. Synthesis of alkenyl-substituted substrates 1, 4 and 6

N-alkenyl quinazolinones, *N*–alkenyl pyrroles, *N*–alkenyl indoles, and *N*–alkenyl benzimidazoles were prepared according to the published procedure.[1-3]

3. General procedure for the synthesis of products 3, 5 and 7

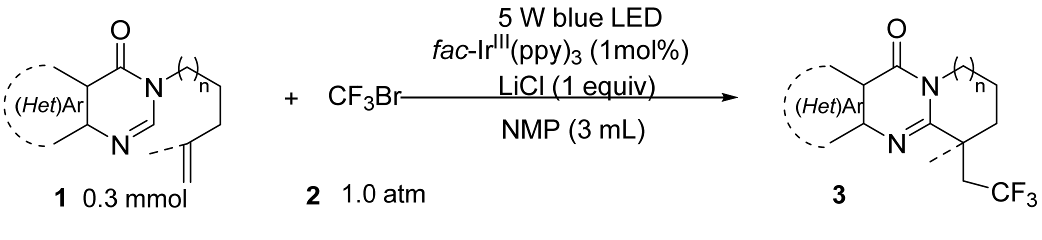

N-alkenyl quinazolinones **1**(0.3 mmol), *fac*-Ir$^{(III)}$(ppy)$_3$ (1.0 mol %), LiCl (0.3 mmol) and NMP (3mL) were added into a 50 mL Schlenk flask, which was degassed with CF_3Br gas 3 times and refilled with CF_3Br (1 atm). Afterward, the reaction mixture was

stirred at room temperature under 5 W blue LED lamp *(hv* = 460 nm) for 16–48 h. The solvent was removed by vacuum distillation and the residue was purified by flash column chromatography on silica gel with petroleum ether and ethyl acetate as eluent. The preparation procedure of compounds **5** and **7** is as same as that of compounds **3**.

4. Procedure for the gram-scale synthesis of 3a

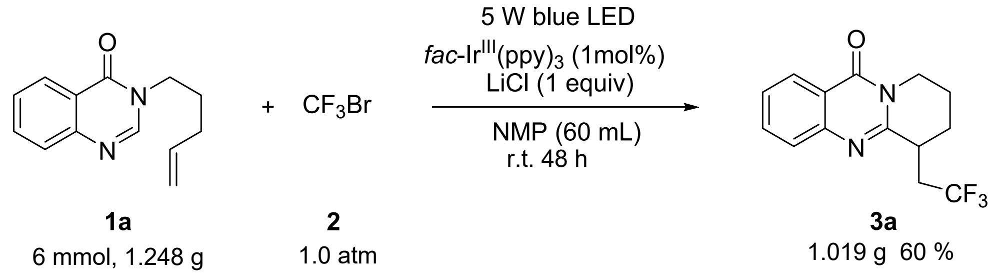

3-(pent-4-en-1-yl) quinazolin-4(3H)-one **1a** (6 mmol), *fac*-Ir(III)(ppy)$_3$ (1.0 mol %), LiCl (6 mmol) and NMP (60 mL) were added into a 100 mL Schlenk flask, which was degassed with CF_3Br gas 3 times and refilled with CF_3Br (1 atm). Afterward, the reaction mixture was stirred at room temperature under 4×5 W blue LED lamp *(hv* = 460 nm) for 48h. The solvent was removed by vacuum distillation and the residue was purified by flash column chromatography on silica gel with petroleum ether/ethyl acetate as eluent (6/1) to obtain **3a** in yield of 60% (1.019 g)

5. Radical trapping experiment

N-Alkenyl quinazolinones **1** (0.3 mmol), *fac*-Ir(III)(ppy)$_3$ (1.0 mol %), LiCl (0.3 mmol), TEMPO or 1,1-diphenylethylene (0.6 mmol, 2 equiv.) and NMP (3 mL) were added into a 50 mL Schlenk flask, which was degassed with CF_3Br gas 3 times and refilled with CF_3Br (1 atm). Afterward, thereaction mixture was stirred at room temperature under 5 W blue LED lamp *(hv* = 460 nm) for 16 h. The solvent was removed by vacuum distillation and the residue was purified by flash column chromatography on silica gel with petroleum ether to obtain (3,3,3-trifluoroprop-1-ene-1,1-diyl)dibenzene **8**.

It is unfortunate that adduct CF_3-TEMPO could not be detected by ^{19}F NMR.

6. Characterization data of products

6-(2,2,2-trifluoroethyl)-6,7,8,9-tetrahydro-11H-pyrido[2,1-b]quinazolin-11-one (**3a**)[4]

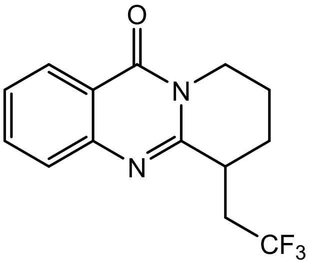

The desired pure product (70 mg, 83% yield) as a white crystal (mp 143–144 °C) was purified by flash column chromatography (PE/EA = 6/1).

^{1}H NMR (400 MHz, $CDCl_3$): (ppm) δ 8.26 (d, J = 8.0, 1H), 7.73 (t, J = 8.4 Hz, 1H), 7.63 (d, J = 8.0 Hz, 1H), 7.45 (t, J = 7.6 Hz, 1H), 4.36 – 4.29 (m, 1H), 3.99 – 3.91 (m, 1H), 3.61 – 3.47 (m, 1H), 3.22 – 3.15 (m, 1H), 2.46 – 2.30 (m, 2H), 2.07 – 2.00 (m, 2H), 1.72 – 1.62 (m, 1H).

^{13}C NMR (150 MHz, $CDCl_3$): (ppm) δ 161.89 (s), 154.70 (s), 146.85 (s), 134.18 (s), 127.05 (q, J_{C-F} = 275.3 Hz), 126.96 (s), 126.64 (s), 126.57 (s), 120.22 (s), 41.13 (s), 36.09 (q, J_{C-F} = 28.2 Hz), 35.33 (q, J_{C-F} = 2.7 Hz), 25.10 (s), 20.60 (s).

^{19}F NMR (376 MHz, $CDCl_3$): (ppm) δ -63.25 (t, J = 11.7 Hz).

1-methyl-6-(2,2,2-trifluoroethyl)-6,7,8,9-tetrahydro-11H-pyrido[2,1-*b*]quinazolin-11-one (**3b**) [4]

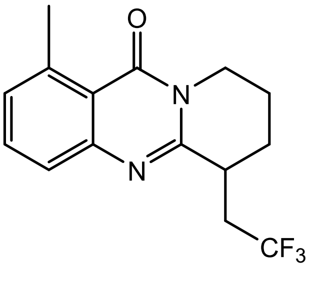

The desired pure product (62 mg, 70% yield) as a white crystal (mp 135–136 °C) was purified by flash column chromatography (PE/EA = 5/1).

^{1}H NMR (400 MHz, $CDCl_3$): (ppm) δ 7.53 (t, J = 8.0, 1H), 7.44 (d, J = 8.0 Hz, 1H), 7.17 (d, J = 7.6 Hz, 1H), 4.22 – 4.16 (m, 1H), 3.94 – 3.88 (m, 1H), 3.59 – 3.45 (m, 1H),

3.17 – 3.09 (m, 1H), 2.86 (s, 3H), 2.41 – 2.30 (m, 2H), 2.09 – 1.93 (m, 2H), 1.69 – 1.59 (m, 1H).

^{13}C NMR (150 MHz, CDCl$_3$): (ppm) δ 162.39 (s), 154.23 (s), 148.31 (s), 140.80 (s), 133.20 (s), 129.05 (s), 127.04 (q, $J_{C\text{-}F}$ = 275.4 Hz), 125.09 (s), 118.69 (s), 41.00 (s), 36.07 (q, $J_{C\text{-}F}$ = 28.1 Hz), 35.24 (s), 25.07 (s), 23.00 (s), 20.71 (s).

^{19}F NMR (376 MHz, CDCl$_3$): (ppm) δ -63.64 (t, J = 11.3 Hz).

HRMS (ESI): m/z calcd for $C_{15}H_{16}F_3N_2O$ [M + H]$^+$ 297.1209, found 297.1204.

1-chloro-6-(2,2,2-trifluoroethyl)-6,7,8,9-tetrahydro-11H-pyrido[2,1-*b*]quinazolin-11-one (**3c**)

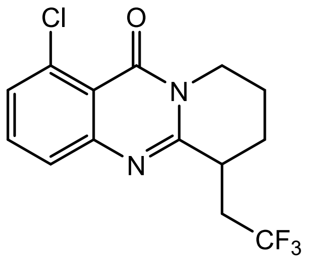

The desired pure product (67 mg, 71% yield) as a white crystal (mp 129–130 °C) was purified by flash column chromatography (PE/EA = 5/1).

^{1}H NMR (400 MHz, CDCl$_3$): (ppm) δ 7.59 – 7.52 (m, 2H), 7.44 (d, J = 7.6 Hz, 1H), 4.26 – 4.19 (m, 1H), 3.98 – 3.92 (m, 1H), 3.56 – 3.42 (m, 1H), 3.19 – 3.12 (m, 1H), 2.42 – 2.32 (m, 2H), 2.10 – 1.95 (m, 2H), 1.71 – 1.63 (m, 1H).

^{13}C NMR (150 MHz, CDCl$_3$): (ppm) δ 159.89 (s), 155.43 (s), 149.15 (s), 133.81 (s), 133.46 (s), 129.16 (s), 126.96 (q, $J_{C\text{-}F}$ = 275.4 Hz), 126.28 (s), 117.29 (s), 41.43 (s), 35.96 (q, $J_{C\text{-}F}$ = 28.4 Hz), 35.39 (q, $J_{C\text{-}F}$ = 2.7 Hz), 24.94 (s), 20.62 (s).

^{19}F NMR (376 MHz, CDCl$_3$): (ppm) δ -63.66 (t, $J_{F\text{-}H}$ = 11.3 Hz).

HRMS (ESI): m/z calcd for $C_{14}H_{12}ClF_3N_2NaO$ [M + Na]$^+$ 339.0482, found 339.0472.

2-methoxy-6-(2,2,2-trifluoroethyl)-6,7,8,9-tetrahydro-11H-pyrido[2,1-*b*]quinazolin-11-one (**3d**) [4]

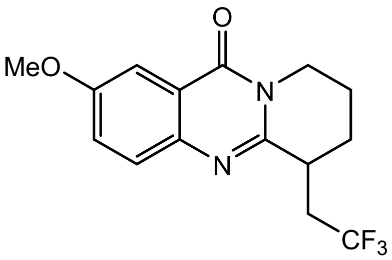

The desired pure product (68 mg, 62% yield) as a white crystal (mp 98–100 °C) was purified by flash column chromatography (PE/EA = 6/1).

^{1}H NMR (400 MHz, $CDCl_3$): (ppm) δ 7.60 (d, J = 2.8 Hz, 1H), 7.54 (d, J = 9.2 Hz, 1H), 7.31 (dd, J = 9.2, 2.8 Hz, 1H), 4.34 – 4.28 (m, 1H), 4.00 – 3.88 (m, 4H), 3.58 – 3.44 (m, 1H), .3.20 – 3.13 (m, 1H), 2.42 – 2.30 (m, 2H), 2.05 – 1.99 (m, 2H), 1.70 – 1.60 (m, 1H).

^{13}C NMR (150 MHz, $CDCl_3$): (ppm) δ 161.74 (s), 158.19 (s), 152.33 (s), 141.54 (s), 128.55 (s), 127.07 (q, J_{C-F} = 275.4 Hz), 124.58 (s), 120.88 (s), 105.78 (s), 55.77 (s), 41.25 (s), 36.10 (q, J_{C-F} = 28.2 Hz), 35.13 (q, J_{C-F} = 2.7 Hz), 25.16 (s), 20.64 (s).

^{19}F NMR (376 MHz, $CDCl_3$): (ppm) δ -63.66 (t, J_{F-H} = 11.7 Hz).

HRMS (ESI): m/z calcd for $C_{15}H_{16}F_3N_2O_2$ $[M + H]^+$ 313.1158, found 313.1160.

2-methyl-6-(2,2,2-trifluoroethyl)-6,7,8,9-tetrahydro-11H-pyrido[2,1-*b*]quinazolin-11-one (**3e**) [4]

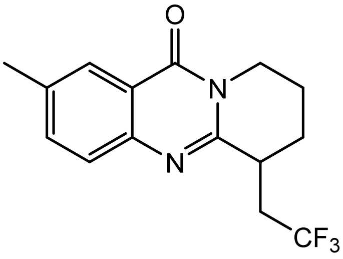

The desired pure product (56 mg, 63% yield) as a white crystal (mp 109–110 °C) was purified by flash column chromatography (PE/EA = 5/1).

^{1}H NMR (400 MHz, $CDCl_3$): (ppm) δ 8.03 (s, 1H), 7.55 – 7.50 (m, 2H), 4.34 – 4.28 (m, 1H), 3.97 – 3.90 (m, 1H), 3.59 – 3.45 (m, 1H), 3.20 – 3.12 (m, 1H), 2.47 (s, 3H), 2.44 – 2.28 (m, 2H), 2.05 – 1.98 (m, 2H), 1.70 – 1.60 (m, 1H).

^{13}C NMR (150 MHz, $CDCl_3$): (ppm) δ 161.84 (s), 153.77 (s), 144.84 (s), 136.65 (s), 135.62 (s), 127.06 (q, $J_{C\text{-}F}$ = 275.6 Hz), 126.73 (s), 125.94 (s), 119.89 (s), 41.06 (s), 36.06 (q, $J_{C\text{-}F}$ = 28.2 Hz), 35.20 (q, $J_{C\text{-}F}$ = 2.7 Hz), 25.10 (s), 21.25 (s), 20.59 (s).

^{19}F NMR (376 MHz, $CDCl_3$): (ppm) δ -63.66 (t, $J_{F\text{-}H}$ = 11.3 Hz).

HRMS (ESI): m/z calcd for $C_{15}H_{16}F_3N_2O$ $[M + H]^+$ 297.1209, found 297.1212.

2-fluoro-6-(2,2,2-trifluoroethyl)-6,7,8,9-tetrahydro-11H-pyrido[2,1-*b*]quinazolin-11-one (**3f**) [4]

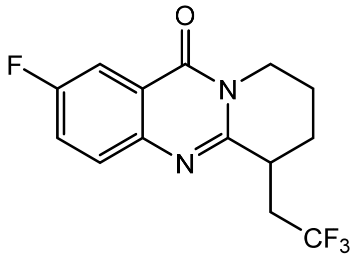

The desired pure product (47 mg,52% yield) as a white crystal (mp 117–119 °C) was purified by flash column chromatography (PE/EA = 7/1).

^{1}H NMR (400 MHz, $CDCl_3$): (ppm) δ 7.85 (dd, J = 8.4, 2.8 Hz, 1H), 7.62 (dd, J = 9.2, 5.2 Hz, 1H), 7.45 – 7.40 (m, 1H), 4.33 – 4.27 (m, 1H), 3.98 – 3.91 (m, 1H), 3.57 – 3.43 (m, 1H), 3.20 – 3.12 (m, 1H), 2.45 – 2.31 (m, 2H), 2.06 – 1.99 (m, 2H), 1.73 – 1.61 (m, 1H).

^{13}C NMR (150 MHz, $CDCl_3$): (ppm) δ 161.19 (d, $J_{C\text{-}F}$ = 3.6 Hz), 160.65 (d, $J_{C\text{-}F}$ = 246.5 Hz), 154.02 (d, $J_{C\text{-}F}$ = 2.1 Hz), 143.52 (d, $J_{C\text{-}F}$ = 2.0 Hz), 129.39 (d, $J_{C\text{-}F}$ = 8.1 Hz), 126.98 (q, $J_{C\text{-}F}$ = 275.4 Hz), 122.79 (d, $J_{C\text{-}F}$ = 24.2 Hz), 121.34 (d, $J_{C\text{-}F}$ = 8.7 Hz), 111.32 (d, $J_{C\text{-}F}$ = 23.4 Hz), 41.27 (s), 36.01 (q, $J_{C\text{-}F}$ = 28.2 Hz), 35.24 (q, $J_{C\text{-}F}$ = 2.7 Hz), 25.06 (s), 20.55 (s).

^{19}F NMR (376 MHz, $CDCl_3$): (ppm) δ -63.70 (t, $J_{F\text{-}H}$ = 11.7 Hz), -113.43 – -113.49 (m).

HRMS (ESI): m/z calcd for $C_{14}H_{12}F_4N_2NaO$ $[M + Na]^+$ 323.0778, found 323.0774.

2-chloro-6-(2,2,2-trifluoroethyl)-6,7,8,9-tetrahydro-11H-pyrido[2,1-*b*]quinazolin-11-one (**3g**) [4]

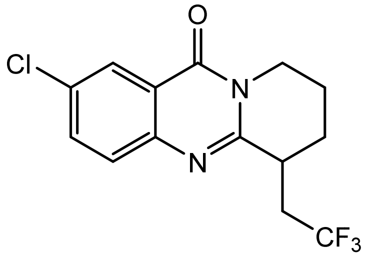

The desired pure product (65 mg, 69% yield) as a white crystal (mp 100–102 °C) was purified by flash column chromatography (PE/EA = 4/1).

^{1}H NMR (400 MHz, $CDCl_3$): (ppm) δ 8.21 (d, J = 2.4 Hz, 1H), 7.65 (dd, J = 8.4 Hz, 2.4 Hz, 1H), 7.57 (d, J = 8.8 Hz, 1H), 4.34 – 4.28 (m, 1H), 3.99 – 3.92 (m, 1H), 3.57 – 3.44 (m, 1H), 3.21 – 3.14 (m, 1H), 2.46 – 2.30 (m, 2H), 2.07 – 2.01 (m, 2H), 1.72 – 1.63 (m, 1H).

^{13}C NMR (150 MHz, $CDCl_3$): (ppm) δ 160.82 (s), 155.05 (s), 145.31 (s), 134.57 (s), 132.20 (s), 128.66 (s), 126.95 (q, J_{C-F} = 275.6 Hz), 125.90 (s), 121.17 (s), 41.31 (s), 35.99 (q, J_{C-F} = 28.2 Hz), 35.34 (q, J_{C-F} = 2.7 Hz), 25.00 (s), 20.52 (s).

^{19}F NMR (376 MHz, $CDCl_3$): (ppm) δ -63.68 (t, J_{F-H} = 11.3 Hz).

HRMS (ESI): m/z calcd for $C_{14}H_{12}ClF_3N_2NaO$ $[M + Na]^+$ 339.0482, found 339.0486.

3-methyl-6-(2,2,2-trifluoroethyl)-6,7,8,9-tetrahydro-11H-pyrido[2,1-*b*]quinazolin-11-one (**3h**) [4]

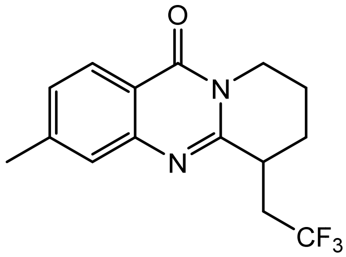

The desired pure product (63 mg, 71% yield) as a white crystal (mp 139–140 °C) was purified by flash column chromatography (PE/EA = 5/1).

^{1}H NMR (400 MHz, $CDCl_3$): (ppm) δ 8.13 (d, J = 8.0 Hz, 1H), 7.42 (s, 1H), 7.26 (d, J = 7.2 Hz, 1H), 4.34 – 4.27 (m, 1H), 3.97 – 3.90 (m, 1H), 3.59 – 3.45 (m, 1H), 3.20 – 3.12 (m, 1H), 2.49 (s, 3H), 2.42 – 2.31 (m, 2H), 2.05 – 1.98 (m, 2H), 1.71 – 1.61 (m, 1H).

^{13}C NMR (150 MHz, $CDCl_3$): (ppm) δ 161.78 (s), 154.72 (s), 146.93 (s), 145.11 (s), 128.15 (s), 127.05 (q, $J_{C\text{-}F}$ = 275.3 Hz), 126.62 (s), 126.43 (s), 117.81 (s), 40.99 (s), 36.07 (q, $J_{C\text{-}F}$ = 28.2 Hz), 35.28 (q, $J_{C\text{-}F}$ = 2.6 Hz), 25.11 (s), 21.81 (s), 20.59 (s).

^{19}F NMR (376 MHz, $CDCl_3$): (ppm) δ -63.67 (t, $J_{F\text{-}H}$ = 11.7 Hz).

HRMS (ESI): m/z calcd for $C_{15}H_{16}F_3N_2O$ $[M + H]^+$ 297.1209, found 297.1200.

3-methoxy-6-(2,2,2-trifluoroethyl)-6,7,8,9-tetrahydro-11H-pyrido[2,1-*b*]quinazolin-11-one (**3i**) [4]

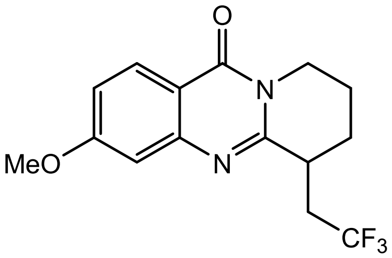

The desired pure product (46 mg, 49% yield) as a white crystal (mp 129–130 °C) was purified by flash column chromatography (PE/EA = 5/1).

^{1}H NMR (400 MHz, $CDCl_3$): (ppm) δ 8.15 (d, J = 8.8 Hz, 1H), 7.04 – 7.00 (m, 2H), 4.32 – 4.26 (m, 1H), 3.98 – 3.91 (m, 4H), 3.59 – 3.45 (m, 1H), 3.20 – 3.13 (m, 1H), 2.44 – 2.32 (m, 2H), 2.06 – 1.99 (m, 2H), 1.72 – 1.62 (m, 1H).

^{13}C NMR (150 MHz, $CDCl_3$): (ppm) δ 164.49 (s), 161.44 (s), 155.42 (s), 149.10 (s), 128.20 (s), 127.04 (q, $J_{C\text{-}F}$ = 275.4 Hz), 116.85 (s), 113.81 (s), 107.37 (s), 55.62 (s), 41.00 (s), 36.17 (q, $J_{C\text{-}F}$ = 28.2 Hz), 35.36 (q, $J_{C\text{-}F}$ = 2.6 Hz), 25.11 (s), 20.58 (s).

^{19}F NMR (376 MHz, $CDCl_3$): (ppm) δ -63.66 (t, $J_{F\text{-}H}$ = 11.3 Hz).

HRMS (ESI): m/z calcd for $C_{15}H_{16}F_3N_2O_2$ $[M + H]^+$ 313.1158, found 313.1159.

3-fluoro-6-(2,2,2-trifluoroethyl)-6,7,8,9-tetrahydro-11H-pyrido[2,1-*b*] quinazolin-11-one (**3j**) [4]

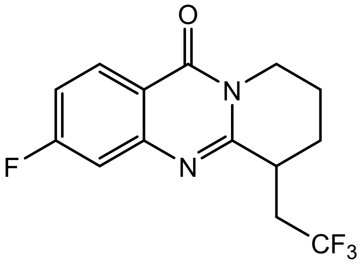

The desired pure product (64 mg, 71% yield) as a white crystal (mp 118–119 °C) was purified by flash column chromatography (PE/EA = 5/1).

^{1}H NMR (400 MHz, $CDCl_3$): (ppm) δ 8.26 (dd, J = 8.8, 6.4 Hz, 1H), 7.26 (dd, J = 10.0, 2.8 Hz, 1H), 7.16 (td, J = 8.4, 2.4 Hz, 1H), 4.34 – 4.27(m, 1H), 3.98 – 3.91 (m, 1H), 3.57 – 3.32 (m, 1H), 3.21 – 3.13 (m, 1H), 2.44 – 2.33 (m, 2H), 2.07 – 2.02 (m, 2H), 1.72 – 1.62 (m, 1H).

^{13}C NMR (150 MHz, $CDCl_3$): (ppm) δ 166.39 (d, J_{C-F} = 252.5 Hz), 161.14 (s), 156.13 (s), 148.94 (d, J_{C-F} = 13.1 Hz), 129.38 (d, J_{C-F} = 10.5 Hz), 126.96 (q, J_{C-F} = 275.4 Hz), 116.95 (d, J_{C-F} = 1.8 Hz), 115.42 (d, J_{C-F} = 23.6 Hz), 112.14 (d, J_{C-F} = 21.6 Hz), 41.14 (s), 36.01 (q, J_{C-F} = 28.4 Hz), 35.41 (q, J_{C-F} = 2.7 Hz), 25.00 (s), 20.54 (s).

^{19}F NMR (376 MHz, $CDCl_3$): (ppm) δ -63.67 (t, J_{F-H} = 11.7 Hz), -104.04 – -104.10. (m).

HRMS (ESI): m/z calcd for $C_{14}H_{12}F_4N_2NaO$ $[M + Na]^+$ 323.0778, found 323.0762.

3-chloro-6-(2,2,2-trifluoroethyl)-6,7,8,9-tetrahydro-11H-pyrido[2,1-*b*]quinazolin-11-one (**3k**) [4]

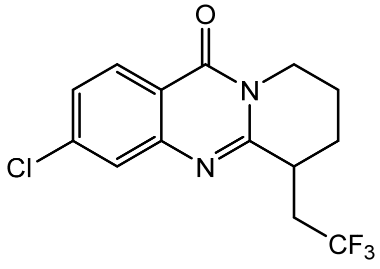

The desired pure product (56 mg, 59% yield) as a white crystal (mp 147–148 °C) was purified by flash column chromatography (PE/EA = 5/1).

^{1}H NMR (400 MHz, $CDCl_3$): (ppm) δ 8.18 (d, J = 8.4 Hz, 1H), 7.63 (d, J = 2.0 Hz, 1H), 7.39 (dd, J = 8.8, 2.0 Hz, 1H), 4.33 – 4.27 (m, 1H), 3.98 – 3.91 (m, 1H), 3.56 – 3.43 (m, 1H), 3.21 – 3.13 (s, 1H), 2.44 – 2.32 (m, 2H), 2.07 – 2.02 (m, 2H), 1.72 – 1.62 (m, 1H).

^{13}C NMR (150 MHz, $CDCl_3$): (ppm) δ 161.26 (s), 156.13 (s), 147.76 (s), 140.35 (s), 128.14 (s), 127.21 (s), 126.94 (q, J_{C-F} = 275.6 Hz), 126.52 (s), 118.66 (s), 41.22 (s), 35.99 (q, J_{C-F} = 28.2 Hz), 35.43 (q, J_{C-F} = 2.7 Hz), 25.01 (s), 20.54 (s).

^{19}F NMR (376 MHz, $CDCl_3$): (ppm) δ -63.68 (t, $J_{F\text{-}H}$ = 11.7 Hz).
HRMS (ESI): m/z calcd for $C_{14}H_{13}ClF_3N_2O$ $[M + H]^+$ 317.0663, found 317.0670.

6-(2,2,2-trifluoroethyl)-3-(trifluoromethyl)-6,7,8,9-tetrahydro-11H-pyrido[2,1-*b*]quinazolin-11-one (**3l**) [4]

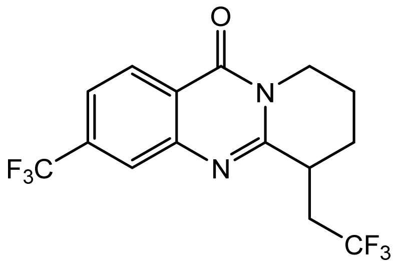

The desired pure product (62 mg, 59% yield) as a white crystal (mp 115–117 °C) was purified by flash column chromatography (PE/EA = 5/1).

^{1}H NMR (400 MHz, $CDCl_3$): (ppm) δ 8.36 (d, J = 8.4 Hz, 1H), 7.93 (s, 1H), 7.65 (d, J = 8.4 Hz, 1H), 4.37 – 4.30 (m, 1H), 4.02 – 3.95 (m, 1H), 3.59 – 3.45 (m, 1H), 3.24 – 3.16 (m, 1H), 2.48 – 2.33 (m, 2H), 2.10 – 2.03 (m, 2H), 1.74 – 1.64 (m, 1H).

^{13}C NMR (150 MHz, $CDCl_3$): (ppm) δ 161.08 (s), 156.34 (s), 146.76 (s), 135.77 (q, $J_{C\text{-}F}$ = 32.7 Hz), 127.84 (s), 126.91 (q, $J_{C\text{-}F}$ = 275.7 Hz), 124.63 (q, $J_{C\text{-}F}$ = 4.0 Hz), 123.38 (q, $J_{C\text{-}F}$ = 271.4 Hz), 122.50 (q, $J_{C\text{-}F}$ = 3.5 Hz), 122.44 (s), 41.40 (s), 35.97 (q, $J_{C\text{-}F}$ = 28.4 Hz), 35.47 (q, $J_{C\text{-}F}$ = 2.6 Hz), 24.99 (s), 20.52 (s).

^{19}F NMR (376 MHz, $CDCl_3$): (ppm) δ -63.62 (s), -63.72 (t, $J_{F\text{-}H}$ = 10.9 Hz).
HRMS (ESI): m/z calcd for $C_{15}H_{13}F_6N_2O$ $[M + H]^+$ 351.0927, found 351.0934.

4-methyl-6-(2,2,2-trifluoroethyl)-6,7,8,9-tetrahydro-11H-pyrido[2,1-*b*]quinazolin-11-one (**3m**) [4]

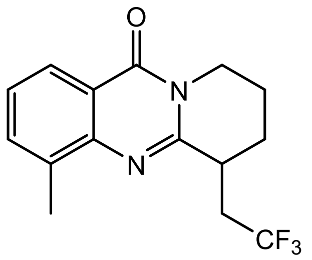

The desired pure product (57 mg, 64% yield) as a white crystal (mp 116–117 °C) was purified by flash column chromatography (PE/EA = 6/1).

^{1}H NMR (400 MHz, $CDCl_3$): (ppm) δ 8.10 (d, J = 8.0 Hz, 1H), 7.56 (d, J = 7.2 Hz, 1H), 7.33 (t, J = 7.6 Hz, 1H), 4.35 – 4.28 (m, 1H), 3.98 – 3.91 (m, 1H), 3.63 – 3.49 (m, 1H), 3.21 – 3.13 (m, 1H), 2.57 (s, 3H), 2.46 – 2.33 (m, 2H), 2.06 – 2.00 (m, 2H), 1.70 – 1.60 (m, 1H).

^{13}C NMR (150 MHz, $CDCl_3$): (ppm) δ 162.17 (s), 153.37 (s), 145.23 (s), 135.49 (s), 134.61 (s), 127.11 (q, $J_{C\text{-}F}$ = 275.4 Hz), 126.11 (s), 124.24 (s), 120.08 (s), 40.94 (s), 36.01 (q, $J_{C\text{-}F}$ = 28.4 Hz), 35.46 (q, $J_{C\text{-}F}$ = 2.6 Hz), 25.26 (s), 20.75 (s), 17.10 (s).

^{19}F NMR (376 MHz, $CDCl_3$): (ppm) δ -63.64 (t, $J_{F\text{-}H}$ = 11.7 Hz).

HRMS (ESI): m/z calcd for $C_{15}H_{15}F_3N_2NaO$ $[M + H]^+$ 319.1029, found 319.1013.

4-fluoro-6-(2,2,2-trifluoroethyl)-6,7,8,9-tetrahydro-11H-pyrido[2,1-*b*]quinazolin-11-one (**3n**)

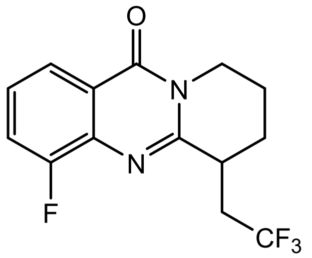

The desired pure product (42 mg, 47% yield) as a white crystal (mp 128–129 °C) was purified by flash column chromatography (PE/EA = 6/1).

^{1}H NMR (400 MHz, $CDCl_3$): (ppm) δ 8.03 (dd, J = 7.6, 0.8 Hz, 1H), 7.47 – 7.35 (m, 2H), 4.35 – 4.28 (m, 1H), 4.01 – 3.94 (m, 1H), 3.63 – 3.49 (m, 1H), 3.26 – 3.18 (m, 1H), 2.49 – 2.34 (m, 2H), 2.09 – 2.00 (m, 2H), 1.74 – 1.64 (m, 1H).

^{13}C NMR (150 MHz, $CDCl_3$): (ppm) δ 160.98 (d, J = 3.3 Hz), 156.74 (d, J = 254.6 Hz), 155.43 (s), 136.39 (d, $J_{C\text{-}F}$ = 11.7 Hz), 126.96 (q, $J_{C\text{-}F}$ = 275.7 Hz), 126.55 (d, $J_{C\text{-}F}$ = 7.5 Hz), 122.13 (s), 122.10 (s), 119.52 (d, $J_{C\text{-}F}$ = 18.8 Hz), 41.36 (s), 35.99 (q, $J_{C\text{-}F}$ = 28.4 Hz), 35.63 (q, $J_{C\text{-}F}$ = 2.7 Hz), 24.91 (s), 20.55 (s).

^{19}F NMR (376 MHz, $CDCl_3$): (ppm) δ -63.52 (t, $J_{F\text{-}H}$ = 11.3 Hz), -126.45 – -126.49 (m).

HRMS (ESI): m/z calcd for $C_{14}H_{12}F_4N_2NaO$ $[M + Na]^+$ 323.0778, found 323.0774.

4-chloro-6-(2,2,2-trifluoroethyl)-6,7,8,9-tetrahydro-11H-pyrido[2,1-*b*]quinazolin-11-one (**3o**)

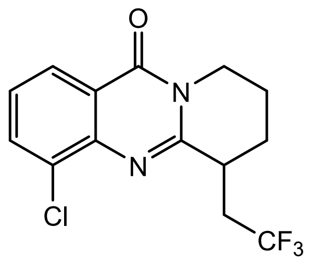

The desired pure product (28 mg, 30% yield) as a white crystal (mp 141–142 °C) was purified by flash column chromatography (PE/EA = 6/1).

^{1}H NMR (400 MHz, $CDCl_3$): (ppm) δ 8.17 (dd, J = 8.0, 1.6 Hz, 1H), 7.81 (dd, J = 7.6, 1.2 Hz, 1H), 7.36 (t, J = 8.0 Hz, 1H), 4.33 – 4.26 (m, 1H), 4.04 – 3.95 (m, 1H), 3.71 – 3.57 (m, 1H), 3.26 – 3.18 (m, 1H), 2.50 – 2.36 (m, 2H), 2.09 – 2.02 (m, 2H), 1.73 – 1.63 (m, 1H).

^{13}C NMR (150 MHz, $CDCl_3$): (ppm) δ 161.23 (s), 155.50 (s), 143.34 (s), 134.30 (s), 131.56 (s), 127.01 (q, J_{C-F} = 275.4 Hz), 126.49 (s), 125.34 (s), 121.61 (s), 41.33 (s), 35.86 (q, J_{C-F} = 28.2 Hz), 35.70 (q, J_{C-F} = 2.6 Hz), 24.93 (s), 20.62 (s).

^{19}F NMR (376 MHz, $CDCl_3$): (ppm) δ -63.54 (t, J_{F-H} = 11.3 Hz).

HRMS (ESI): m/z calcd for $C_{14}H_{12}ClF_3N_2NaO$ $[M + Na]^+$ 339.0482, found 339.0479.

2,3-dimethoxy-6-(2,2,2-trifluoroethyl)-6,7,8,9-tetrahydro-11H-pyrido[2,1-*b*]quinazolin-11-one (**3p**)

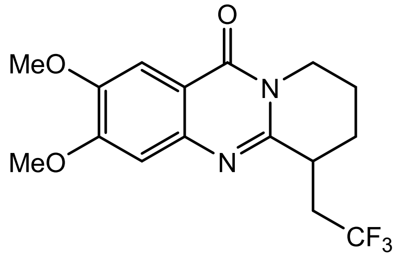

The desired pure product (62 mg, 58% yield) as a white crystal (mp 182–183 °C) was purified by flash column chromatography (PE/EA = 5/2).

^{1}H NMR (400 MHz, $CDCl_3$): (ppm) δ 7.57 (s), 7.03 (s), 4.33 – 4.26 (m, 1H), 4.03 – 3.96 (m, 7H), 3.58 – 3.44 (m, 1H), 3.21 – 3.14 (m, 1H), 2.42 – 2.29 (m, 2H), 2.07 – 1.99 (m, 2H), 1.73 – 1.62 (m, 1H).

^{13}C NMR (150 MHz, $CDCl_3$): (ppm) δ 161.21 (s), 154.90 (s), 153.37 (s), 148.91 (s), 143.17 (s), 127.03 (q, $J_{C\text{-}F}$ = 275.4 Hz), 113.51 (s), 107.25 (s), 105.46 (s), 56.25 (s), 56.22 (s), 41.20 (s), 36.22 (q, $J_{C\text{-}F}$ = 28.1 Hz), 35.20 (q, $J_{C\text{-}F}$ = 2.7 Hz), 25.16 (s), 20.60 (s).

^{19}F NMR (376 MHz, $CDCl_3$): (ppm) δ -63.67 (t, $J_{F\text{-}H}$ = 12.0 Hz).

HRMS (ESI): m/z calcd for $C_{16}H_{18}F_3N_2O_3$ $[M + H]^+$ 343.1264, found 343.1274.

2,4-dimethyl-6-(2,2,2-trifluoroethyl)-6,7,8,9-tetrahydro-11H-pyrido[2,1-*b*]quinazolin-11-one (**3q**)

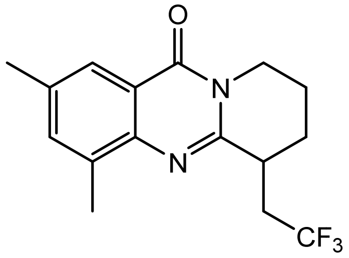

The desired pure product (29 mg, 31% yield) as a white crystal (mp 106–108 °C) was purified by flash column chromatography (PE/EA = 6/1).

^{1}H NMR (400 MHz, $CDCl_3$): (ppm) δ 7.88 (s), 7.39 (s), 4.34 – 4.27 (m, 1H), 3.97 – 3.90 (m, 1H), 3.62 – 3.48 (m, 1H), 3.17 – 3.12 (m, 1H), 2.53 (s, 3H), 2.43(s, 3H), 2.40 – 2.31 (m, 2H), 2.05 – 1.99 (m, 2H), 1.69 – 1.58 (m, 1H).

^{13}C NMR (150 MHz, $CDCl_3$): (ppm) δ 162.17 (s), 152.46 (s), 143.29 (s), 136.19 (s), 136.10 (s), 135.21 (s), 127.15 (q, $J_{C\text{-}F}$ = 275.3 Hz), 123.59 (s), 119.85 (s), 40.92 (s), 36.02 (q, $J_{C\text{-}F}$ = 28.1 Hz), 35.37 (q, $J_{C\text{-}F}$ = 2.7 Hz), 25.30 (s), 21.25 (s), 20.79 (s), 16.97 (s).

^{19}F NMR (376 MHz, $CDCl_3$): (ppm) δ -63.65 (t, $J_{F\text{-}H}$ = 11.3 Hz).

HRMS (ESI): m/z calcd for $C_{16}H_{18}F_3N_2O$ $[M + H]^+$ 311.1366, found 311.1366.

2,3-difluoro-6-(2,2,2-trifluoroethyl)-6,7,8,9-tetrahydro-11H-pyrido[2,1-*b*]quinazolin-11-one (**3r**)

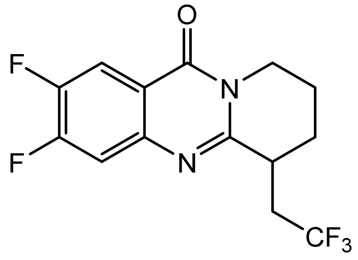

The desired pure product (77 mg, 81% yield) as a white crystal (mp 123–124 °C) was purified by flash column chromatography (PE/EA = 6/1).

^{1}H NMR (400 MHz, $CDCl_3$): (ppm) δ 8.01 – 7.98 (m, 1H), 7.42 – 7.39 (m, 1H), 4.32 – 4.27 (m, 1H), 3.98 – 3.93 (m, 1H), 3.51 – 3.42 (m, 1H), 3.19 – 3.14 (m, 1H), 2.44 – 2.32 (m, 2H), 2.08 – 2.00 (m, 2H), 1.71 – 1.64 (m, 1H).

^{13}C NMR (150 MHz, $CDCl_3$): (ppm) δ 160.43 (d, $J_{C\text{-}F}$ = 3.0 Hz), 155.62 (d, $J_{C\text{-}F}$ = 2.1 Hz), 154.78 (dd, $J_{C\text{-}F}$ = 255.9, 14.7 Hz), 149.46 (dd, $J_{C\text{-}F}$ = 249.9, 14.3 Hz), 144.57 (dd, $J_{C\text{-}F}$ = 11.3, 2.4 Hz), 126.90 (q, $J_{C\text{-}F}$ = 275.4 Hz), 117.05 (dd, $J_{C\text{-}F}$ = 6.6, 2.1 Hz), 114.63 (d, $J_{C\text{-}F}$ = 17.6 Hz), 113.83 (dd, $J_{C\text{-}F}$ = 19.1, 2.3 Hz), 41.32 (s), 35.92 (q, $J_{C\text{-}F}$ = 28.5 Hz), 35.32 (q, $J_{C\text{-}F}$ = 2.7 Hz), 24.93 (s), 20.46 (s).

^{19}F NMR (376 MHz, $CDCl_3$): (ppm) δ -63.77 (t, $J_{F\text{-}H}$ = 10.9 Hz), -127.27 – -127.38 (m), -136.99 – -137.10 (m).

HRMS (ESI): m/z calcd for $C_{14}H_{11}F_5N_2NaO$ $[M + Na]^+$ 341.0684, found 341.0691.

3-(2,2,2-trifluoroethyl)-2,3-dihydropyrrolo[2,1-*b*]quinazolin-9(1H)-one (**3s**) [4]

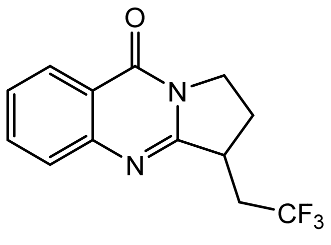

The desired pure product (43 mg, 56% yield) as a white crystal (mp 91–92 °C) was purified by flash column chromatography (PE/EA = 6/1).

^{1}H NMR (600 MHz, $CDCl_3$): (ppm) δ 8.28 (dd, J = 7.8, 1.2 Hz, 1H), 7.75 – 7.72 (m, 1H), 7.66 (d, J = 7.8 Hz, 1H), 7.47 (t, J = 7.8 Hz, 1H), 4.41 – 4.37 (m, 1H), 3.97 – 3.92 (m, 1H), 3.55 – 3.50 (m, 1H), 3.31 – 3.23 (m, 1H), 2.70 – 2.66 (m, 1H), 2.34 – 2.24 (m, 1H), 2.07 – 2.00 (m, 1H).

^{13}C NMR (150 MHz, $CDCl_3$): (ppm) δ 160.69 (s), 158.49 (s), 148.85 (s), 134.25 (s), 127.04 (s), 126.64 (s), 126.55 (q, $J_{C\text{-}F}$ = 275.1 Hz), 126.44 (s), 120.82 (s), 44.68 (s), 38.32 (q, $J_{C\text{-}F}$ = 2.9 Hz), 35.95 (q, $J_{C\text{-}F}$ = 28.8 Hz), 27.46 (s).

^{19}F NMR (376 MHz, $CDCl_3$): (ppm) δ -65.10 (t, $J_{F\text{-}H}$ = 10.5 Hz).

HRMS (ESI): m/z calcd for $C_{13}H_{11}F_3N_2NaO$ $[M + Na]^+$ 291.0716, found 291.0710.

6-methyl-3-(2,2,2-trifluoroethyl)-2,3-dihydropyrrolo[2,1-*b*]quinazolin-9(1H)-one (**3t**)[5]

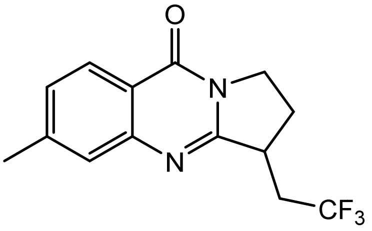

The desired pure product (46 mg, 54% yield) as a white crystal (mp 93–94 °C) was purified by flash column chromatography (PE/EA = 6/1).

^{1}H NMR (600 MHz, $CDCl_3$): (ppm) δ 8.17 (d, J = 8.4 Hz, 1H), 7.46 (s, 1H), 7.29 (d, J = 7.8 Hz, 1H), 4.39 – 4.36 (m, 1H), 3.96 – 3.90 (m, 1H), 3.53 – 3.48 (m, 1H), 3.29 – 3.21 (m, 1H), 2.69 – 2.65 (m, 1H), 2.50 (s, 3H), 2.32 – 2.23 (m, 1H), 2.06 – 1.99 (m, 1H).

^{13}C NMR (150 MHz, $CDCl_3$): (ppm) δ 160.61 (s), 158.53 (s), 148.96 (s), 145.24 (s), 128.16 (s), 126.78 (s), 126.55 (q, $J_{C\text{-}F}$ = 275.1 Hz), 126.18 (s), 118.35 (s), 44.56 (s), 38.26 (q, $J_{C\text{-}F}$ = 2.7 Hz), 35.92 (q, $J_{C\text{-}F}$ = 28.8 Hz), 27.43 (s), 21.78 (s).

^{19}F NMR (376 MHz, $CDCl_3$): (ppm) δ -65.12 (t, $J_{F\text{-}H}$ = 10.5 Hz).

HRMS (ESI): m/z calcd for $C_{14}H_{13}F_3N_2NaO$ $[M + Na]^+$ 305.0872, found 305.0881.

3-methyl-3-(2,2,2-trifluoroethyl)-2,3-dihydropyrrolo[2,1-*b*]quinazolin-9(1H)-one (**3u**) [4]

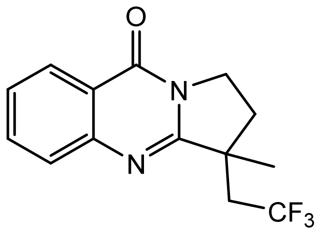

The desired pure product (64 mg, 76% yield) as a white crystal (mp 95–96 °C) was purified by flash column chromatography (PE/EA = 4/1).

^{1}H NMR (600 MHz, $CDCl_3$): (ppm) δ 8.29 (d, J = 8.0 Hz, 1H), 7.74 (t, J = 7.6 Hz, 1H), 7.67 (d, J = 8.4 Hz, 1H), 7.48 – 7.44 (m, 1H), 4.33 – 4.27 (m, 1H), 4.07 – 4.00 (m, 1H), 2.99 – 2.87 (m, 1H), 2.62 – 2.49 (m, 1H), 2.43 – 2.35 (m, 1H), 2.30 – 2.24 (m, 1H), 1.49 (s, 3H).

^{13}C NMR (150 MHz, $CDCl_3$): (ppm) δ 162.38 (s), 160.81 (s), 149.15 (s), 134.13 (s), 127.16 (s), 126.46 (s), 126.42 (q, $J_{C\text{-}F}$ = 276.3 Hz), 126.36 (s), 120.82 (s), 43.86 (q, $J_{C\text{-}F}$ = 2.0 Hz), 44.30 (s), 41.38 (q, $J_{C\text{-}F}$ = 27.6 Hz), 32.76 (s), 23.86 (s).

^{19}F NMR (376 MHz, $CDCl_3$): (ppm) δ -65.47 (t, $J_{F\text{-}H}$ = 11.3 Hz).

HRMS (ESI): m/z calcd for $C_{14}H_{13}F_3N_2NaO$ $[M + Na]^+$ 305.0872, found 305.0871.

6-(2,2,2-trifluoroethyl)-7,8,9,10-tetrahydroazepino[2,1-*b*]quinazolin-12(6H)-one (**3v**)

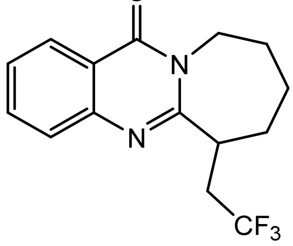

The desired pure product (27 mg, 30% yield) as a white crystal (mp 96–97 °C) was purified by flash column chromatography (PE/EA = 4/1).

^{1}H NMR (600 MHz, $CDCl_3$): (ppm) δ 8.26 (d, J = 7.8 Hz, 1H), 7.72 (t, J = 7.8 Hz, 1H), 7.62 (d, J = 7.8 Hz, 1H), 7.45 (t, J = 7.8 Hz, 1H), 5.35 (d, J =13.8 Hz, 1H), 3.55 (t, J =

13.2 Hz , 1H), 3.41 – 3.32 (m, 2H), 2.49 – 2.39 (m, 1H), 2.21 (d, J =13.8 Hz, 1H), 2.10 – 2.07 (m, 1H), 1.94 – 1.89 (m, 1H), 1.85 – 1.78 (m, 1H), 1.63 – 1.50 (m, 2H).

^{13}C NMR (150 MHz, $CDCl_3$): (ppm) δ 161.87 (s), 158.27 (s), 146.75 (s), 134.12 (s), 127.18 (s), 127.16 (q, J_{C-F} = 276.3 Hz), 126.97 (s), 126.67 (s), 120.36 (s), 41.56 (s), 38.29 (s), 37.02 (q, J_{C-F} = 27.6 Hz), 32.32 (s), 27.93 (s), 27.10 (s).

^{19}F NMR (376 MHz, $CDCl_3$): (ppm) δ -64.14 (t, J_{F-H} = 11.3 Hz).

HRMS (ESI): m/z calcd for $C_{15}H_{15}F_3N_2NaO$ $[M + Na]^+$ 319.1029, found 319.1028.

7-(2,2,2-trifluoroethyl)-7,8,9,10-tetrahydro-5H-dipyrido[1,2-*a*:3',2'-*e*]pyrimidin-5-one (**3w**)

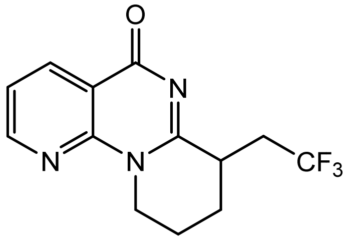

The desired pure product (35 mg, 41% yield) as a colorless liquid was purified by flash column chromatography (PE/EA = 1/1).

^{1}H NMR (400 MHz, $CDCl_3$): (ppm) δ 8.97 (dd, J = 4.4, 2.0 Hz, 1H), 8.60 (dd, J = 7.6, 2.0 Hz, 1H), 7.43 (dd, J = 7.6, 4.4 Hz, 1H), 4.37 – 4.30 (m, 1H), 4.00 – 3.94 (m, 1H), 3.72 – 3.58 (m, 1H), 3.29 – 3.22 (m, 1H), 2.53 – 2.39 (m, 2H), 2.10 – 2.04 (m, 2H), 1.77 – 1.67 (m, 1H).

^{13}C NMR (150 MHz, $CDCl_3$): (ppm) δ 162.05 (s), 158.93 (s), 156.73 (s), 156.07 (s), 136.45 (s), 126.88 (q, J_{C-F} = 275.4 Hz), 122.21 (s), 115.32 (s), 41.45 (s), 35.94 (q, J_{C-F} = 28.5 Hz), 35.64 (q, J_{C-F} = 2.6 Hz), 24.79 (s), 20.41 (s).

^{19}F NMR (376 MHz, $CDCl_3$): (ppm) δ -63.62 (t, J_{F-H} = 11.3 Hz).

HRMS (ESI): m/z calcd for $C_{13}H_{12}F_3N_3NaO$ $[M + Na]^+$ 306.0825, found 306.0812.

9-(2,2,2-trifluoroethyl)-6,7,8,9-tetrahydro-4H-pyrido[1,2-*a*]thieno[2,3-*d*]pyrimidin-4-one (**3x**)

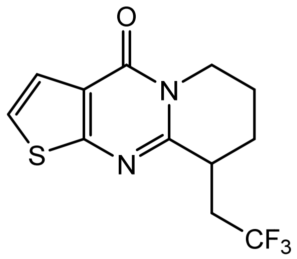

The desired pure product (60 mg, 69% yield) as a white crystal (mp 103–104 °C) was purified by flash column chromatography (PE/EA = 6/1).

^{1}H NMR (400 MHz, $CDCl_3$): (ppm) δ 7.76 (d, J = 5.2 Hz, 1H), 7.26 (d, J = 5.2 Hz, 1H), 4.44 – 3.37 (m, 1H), 3.97 – 3.90 (m, 1H), 3.53 – 3.39 (m, 1H), 3.24 – 3.16 (m, 1H), 2.46 – 2.30 (m, 2H), 2.09 – 1.98 (m, 2H), 1.72 – 1.62 (m, 1H).

^{13}C NMR (150 MHz, $CDCl_3$): (ppm) δ 158.00 (s), 156.44 (s), 155.60 (s), 134.11 (s), 126.89 (q, $J_{C\text{-}F}$ = 275.6 Hz), 124.79 (s), 121.15 (s), 40.92 (s), 36.18 (q, $J_{C\text{-}F}$ = 28.4 Hz), 35.64 (q, $J_{C\text{-}F}$ = 2.7 Hz), 25.01 (s), 20.45 (s).

^{19}F NMR (376 MHz, $CDCl_3$): (ppm) δ -63.69 (t, $J_{F\text{-}H}$ = 11.3 Hz).

HRMS (ESI): m/z calcd for $C_{12}H_{11}F_3N_2NaOS$ $[M + Na]^+$ 311.0436, found 311.0430.

1-(1-(2,2,2-trifluoroethyl)-2,3-dihydro-1H-pyrrolizin-5-yl)ethan-1-one (**5a**)

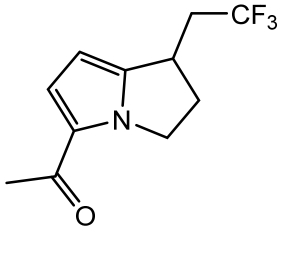

The desired pure product (44 mg, 63% yield) as a white crystal (mp 101–102°C) was purified by flash column chromatography (PE/EA = 4/1).

^{1}H NMR (400 MHz, $CDCl_3$): (ppm) δ 6.92 (d, J = 4.0 Hz, 1H), δ 5.94 (d, J = 4.0 Hz, 1H), 4.52 – 4.46 (m, 1H), 4.19 – 4.12 (m, 1H), 3.47 – 3.40 (m, 1H), 2.85 – 2.77 (m, 1H), 2.69 – 2.55 (m, 1H), 2.37 (s, 3H), 2.32 – 2.23 (m, 2H).

^{13}C NMR (150 MHz, $CDCl_3$): (ppm) δ 187.56 (s), 145.54 (s), 127.87 (s), 126.24 (q, $J_{C\text{-}F}$ = 275.6 Hz), 121.68 (s), 101.44 (s), 47.36 (s), 38.05 (q, $J_{C\text{-}F}$ = 27.9 Hz), 34.78 (s), 31.63 (q, $J_{C\text{-}F}$ = 2.9 Hz), 25.86 (s).

^{19}F NMR (376 MHz, $CDCl_3$): (ppm) δ -65.27 (t, $J_{F\text{-}H}$ = 10.5 Hz).
HRMS (ESI): m/z calcd for $C_{11}H_{13}F_3NO$ $[M + H]^+$ 232.0944, found 232.0945.

1-(8-(2,2,2-trifluoroethyl)-5,6,7,8-tetrahydroindolizin-3-yl)ethan-1-one (**5b**)

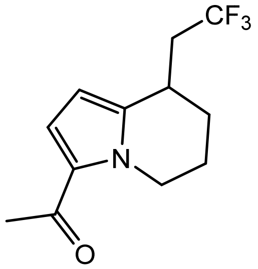

The desired pure product (44 mg, 53% yield) as a white crystal (mp 81–83°C) was purified by flash column chromatography (PE/EA = 4/1).

^{1}H NMR (400 MHz, $CDCl_3$): (ppm) δ 6.95 (d, J = 4.4 Hz, 1H), 6.00 (dd, J = 4.0, 0.8 Hz, 1H), 4.62 – 4.56 (m, 1H), 4.19 – 4.11 (m, 1H), 3.24 – 3.20 (m, 1H), 2.74 – 2.60 (m, 1H), 2.40 (s, 3H), 2.32 – 2.23 (m, 1H), 2.21 – 2.15 (m, 1H), 2.12 – 2.04 (m, 1H), 1.93 – 1.83 (m, 1H), 1.61 – 1.52 (m, 1H).

^{13}C NMR (150 MHz, $CDCl_3$): (ppm) δ 187.91 (s), 139.24 (s), 130.15 (s), 126.45 (q, $J_{C\text{-}F}$ = 276.0 Hz), 119.61 (s), 105.81 (s), 46.03 (s), 39.18 (q, $J_{C\text{-}F}$ = 27.5 Hz), 29.58 (q, $J_{C\text{-}F}$ = 2.9 Hz), 27.06 (s), 25.78 (s), 21.80 (s).

^{19}F NMR (376 MHz, $CDCl_3$): (ppm) δ -63.93 (t, $J_{F\text{-}H}$ = 10.5 Hz).
HRMS (ESI): m/z calcd for $C_{12}H_{14}F_3NNaO$ $[M + Na]^+$ 268.0920, found 268.0912.

1-(9-(2,2,2-trifluoroethyl)-6,7,8,9-tetrahydropyrido[1,2-*a*]indol-10-yl)ethan-1-one (**5e**)

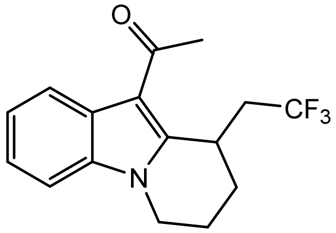

The desired pure product (42 mg, 47% yield) as a white crystal (mp 107–108°C) was purified by flash column chromatography (PE/EA = 4/1).

^{1}H NMR (600 MHz, CDCl$_3$): (ppm) δ 7.91 (d, J = 7.8 Hz, 1H), 7.37 – 7.28 (m, 3H), 4.34 – 4.31 (m, 1H), 4.25 – 4.23 (m, 1H), 3.94 – 3.89 (m, 1H), 2.89 – 2.80 (m, 1H), 2.71 (s, 3H), 2.36 – 2.27 (m, 2H), 2.23 – 2.12 (m, 2H), 1.91 – 1.85 (m, 1H).

^{13}C NMR (150 MHz, CDCl$_3$): (ppm) δ 193.74 (s), 147.00 (s), 136.11 (s), 126.53 (q, J_{C-F} = 276.6 Hz), 125.99 (s), 122.60 (s), 122.19 (s), 120.45 (s), 112.34 (s), 109.78 (s), 42.57 (s), 35.45 (q, J_{C-F} = 27.2 Hz), 31.58 (s), 28.54 (s), 22.49 (s), 17.30 (s).

^{19}F NMR (376 MHz, CDCl$_3$): (ppm) δ -63.31 (t, J_{F-H} = 10.9 Hz).

HRMS (ESI): m/z calcd for $C_{16}H_{16}F_3NNaO$ [M + Na]$^+$ 318.1076, found 318.1076.

1-(1-(2,2,2-trifluoroethyl)-2,3-dihydro-1H-pyrrolo[1,2-*a*]indol-9-yl)ethan-1-one (**5f**)

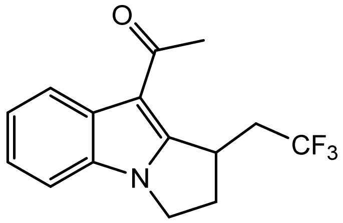

The desired pure product (56 mg, 66% yield) as a white crystal (mp 112–113°C) was purified by flash column chromatography (PE/EA = 6/1).

^{1}H NMR (400 MHz, CDCl$_3$): (ppm) δ 7.95 – 7.93 (m, 1H), 7.32 – 7.24 (m, 3H), 4.22 – 4.11 (m, 2H), 3.99 – 3.93 (m, 1H), 3.26 – 3.13 (m, 1H), 2.95 – 2.85 (m, 1H), 2.70 – 2.62 (m, 4H), 2.29 – 2.15 (m, 1H).

^{13}C NMR (150 MHz, CDCl$_3$): (ppm) δ 193.23 (s), 152.91 (s), 132.46 (s), 129.99 (s), 126.66 (q, J_{C-F} = 276.0 Hz), 122.30 (s), 122.28 (s), 121.03 (s), 110.53 (s), 110.30 (s), 43.27 (s), 35.64 (q, J_{C-F} = 27.5 Hz), 33.85 (q, J_{C-F} = 3.0 Hz), 32.11 (s), 30.42 (s).

^{19}F NMR (376 MHz, CDCl$_3$): (ppm) δ -64.59 (t, J_{F-H} = 10.9 Hz).

HRMS (ESI): m/z calcd for $C_{15}H_{14}F_3NNaO$ [M + Na]$^+$ 304.0920, found 304.0922.

2,2,2-trifluoro-1-(1-(2,2,2-trifluoroethyl)-2,3-dihydro-1H-pyrrolo[1,2-*a*]indol-9-yl)ethan-1-one (**5g**)

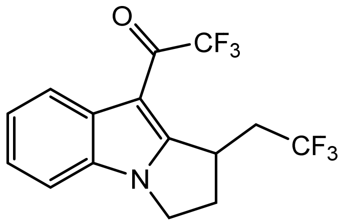

The desired pure product (81 mg, 81% yield) as a white crystal (mp 126–128°C) was purified by flash column chromatography (PE/EA = 4/1).

^{1}H NMR (400 MHz, $CDCl_3$): (ppm) δ 8.08 – 8.05 (m, 1H), 7.36 – 7.32 (m, 3H), 4.25 – 4.22 (m, 2H), 4.05 – 4.00 (m, 1H), 3.01 – 2.89 (m, 2H), 2.75 – 2.72 (m, 1H), 2.36 – 2.22 (m, 1H).

^{13}C NMR (150 MHz, $CDCl_3$): (ppm) δ 174.37 (q, J_{C-F} = 36.5 Hz), 157.35 (s), 132.75 (s), 128.97 (s), 126.29 (q, J_{C-F} = 276.3 Hz), 123.77 (s), 123.68 (s), 121.82 (q, J_{C-F} = 3.9 Hz), 117.10 (q, J_{C-F} = 287.3 Hz), 110.70 (s), 103.90 (s), 43.78 (s), 35.31 (q, J_{C-F} = 27.8 Hz), 34.50 (q, J_{C-F} = 2.9 Hz), 31.76 (s).

^{19}F NMR (376 MHz, $CDCl_3$): (ppm) δ -64.59 (t, J_{F-H} = 10.5 Hz), -75.40 (s).

HRMS (ESI): m/z calcd for $C_{15}H_{11}F_6NNaO$ $[M + Na]^+$ 358.0637, found 358.0641.

Methyl 1-(2,2,2-trifluoroethyl)-2,3-dihydro-1H-pyrrolo[1,2-*a*]indole-9-carboxylate(**5h**)

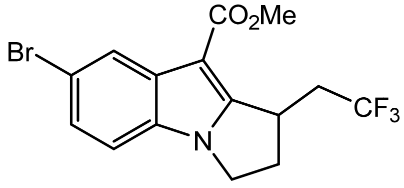

The desired pure product (52 mg, 46% yield) as a white crystal (mp 92–93°C) was purified by flash column chromatography (PE/EA = 4/1).

^{1}H NMR (400 MHz, $CDCl_3$): (ppm) δ 8.24 (d, J = 1.6 Hz, 1H), 7.32 (d, J = 8.4 Hz, 1H), 7.12 (d, J = 8.4 Hz, 1H), 4.22 – 4.08 (m, 2H), 3.92 – 3.88 (m, 4H), 3.36 – 3.24 (m, 1H), 2.99 – 2.90 (m, 1H), 2.65 – 2.60 (m, 1H), 2.33 – 2.19 (m, 1H).

^{13}C NMR (150 MHz, $CDCl_3$): (ppm) δ 164.90 (s), 153.22 (s), 132.13 (s), 130.97 (s), 126.54 (q, J_{C-F} = 276.0 Hz), 125.33 (s), 124.44 (s), 115.66 (s), 111.39 (s), 99.51 (s), 51.05 (s), 43.70 (s), 36.28 (q, J_{C-F} = 27.6 Hz), 33.43 (q, J_{C-F} = 1.5 Hz), 32.60 (s).

^{19}F NMR (376 MHz, $CDCl_3$): (ppm) δ -64.87 (t, $J_{F\text{-}H}$ = 10.5 Hz).

HRMS (ESI): m/z calcd for $C_{15}H_{13}BrF_3NNaO$ $[M + Na]^+$ 397.9974, found 397.9972.

4-(2,2,2-trifluoroethyl)-1,2,3,4-tetrahydrobenzo[4,5]imidazo[1,2-*a*]pyridine (**7a**)[6]

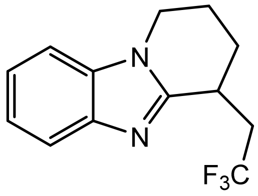

The desired pure product (53 mg, 70% yield) as a white crystal (mp 93 – 94 °C) was purified by flash column chromatography (PE/EA = 2/1).

^{1}H NMR (400 MHz, $CDCl_3$): (ppm) δ 7.72 – 7.70 (m, 1H), 7.29 – 7.22 (m, 3H), 4.21 – 4.16 (m, 1H), 3.96 – 3.89 (m, 1H), 3.55 – 3.45 (m, 1H), 3.42 – 3.34 (m, 1H), 2.46 – 2.43 (m, 1H), 2.37 – 2.23 (m, 2H), 2.11 – 2.00 (m, 1H), 1.75 – 1.65 (m, 1H).

^{13}C NMR (150 MHz, $CDCl_3$): (ppm) δ 152.13 (s), 142.42 (s), 134.75 (s), 126.76 (q, $J_{C\text{-}F}$ = 275.7 Hz), 122.37 (s), 122.24 (s), 119.08 (s), 109.00 (s), 42.29 (s), 37.09 (q, $J_{C\text{-}F}$ = 28.2 Hz), 31.41 (q, $J_{C\text{-}F}$ = 2.8 Hz), 26.64 (s), 21.58 (s).

^{19}F NMR (376 MHz, $CDCl_3$): (ppm) δ -63.89 (t, $J_{F\text{-}H}$ = 11.3 Hz).

8-methyl-4-(2,2,2-trifluoroethyl)-1,2,3,4-tetrahydrobenzo[4,5]imidazo[1,2-*a*]pyridine (**7b**)

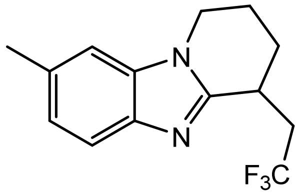

The desired pure product (58 mg, 72% yield) as a white crystal (mp 94 – 95 °C) was purified by flash column chromatography (PE/EA = 10/1).

^{1}H NMR (400 MHz, $CDCl_3$): (ppm) δ 7.18 – 7.12 (m, 2H), 7.06 (d, J = 6.0 Hz, 1H), 4.22 – 4.17 (m, 1H), 3.99 – 3.92 (m, 1H), 3.60 – 3.41 (m, 2H), 2.65 (s, 3H), 2.47 – 2.43 (m, 1H), 2.38 – 2.22 (m, 2H), 2.13 – 2.02 (m, 1H), 1.79 – 1.69 (m, 1H).

^{13}C NMR (150 MHz, $CDCl_3$): (ppm) δ 151.27 (s), 141.85 (s), 134.43 (s), 129.22 (s), 126.82 (q, $J_{C\text{-}F}$ = 275.9 Hz), 122.72 (s), 122.10 (s), 106.43 (s), 42.41 (s), 37.25 (q, $J_{C\text{-}F}$ = 27.9 Hz), 31.41 (q, $J_{C\text{-}F}$ = 2.9 Hz), 26.58 (s), 21.46 (s), 16.58 (s).

^{19}F NMR (376 MHz, $CDCl_3$): (ppm) δ -63.58 (t, $J_{F\text{-}H}$ = 11.3 Hz).

HRMS (ESI): m/z calcd for $C_{14}H_{16}F_3N_2$ $[M + H]^+$ 269.1260, found 269.1255.

8-methoxy-4-(2,2,2-trifluoroethyl)-1,2,3,4-tetrahydrobenzo[4,5]imidazo[1,2-*a*]pyridine (**7c**)

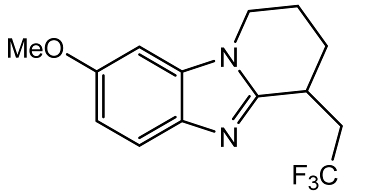

The desired pure product (58 mg, 68% yield) as a white crystal (mp 130 – 131 °C) was purified by flash column chromatography (PE/EA = 5/1).

^{1}H NMR (400 MHz, $CDCl_3$): (ppm) δ 7.59 (d, J = 8.8 Hz, 1H), 6.90 (dd, J = 8.8, 2.0 Hz, 1H), 6.76 (d, J = 2.4 Hz, 1H), 4.19 – 4.15 (m, 1H), 3.95 – 3.89 (m, 1H), 3.86 (s, 3H), 3.54 – 3.35 (m, 2H), 2.48 – 2.40 (m, 1H), 2.37 – 2.26 (m, 2H), 2.14 – 2.02 (m, 1H), 1.76 – 1.66 (m, 1H).

^{13}C NMR (150 MHz, $CDCl_3$): (ppm) δ 156.29 (s), 151.30 (s), 136.85 (s), 135.33 (s), 126.78 (q, $J_{C\text{-}F}$ = 275.7 Hz), 119.50 (s), 111.27 (s), 92.92 (s), 55.82 (s), 42.30 (s), 37.07 (q, $J_{C\text{-}F}$ = 27.9 Hz), 31.44 (q, $J_{C\text{-}F}$ = 2.7 Hz), 26.70 (s), 21.63 (s).

^{19}F NMR (376 MHz, $CDCl_3$): (ppm) δ -63.90 (t, $J_{F\text{-}H}$ = 11.3 Hz).

HRMS (ESI): m/z calcd for $C_{14}H_{16}F_3N_2O$ $[M + H]^+$ 285.1209, found 285.1205.

7,8-dimethoxy-4-(2,2,2-trifluoroethyl)-1,2,3,4-tetrahydrobenzo[4,5]imidazo[1,2-a]pyridine (**7d**)

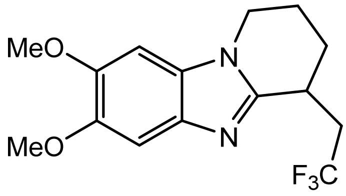

The desired pure product (56 mg, 59% yield) as a white crystal (mp 148 – 149 °C) was purified by flash column chromatography (PE/EA = 3/1).

^{1}H NMR (400 MHz, $CDCl_3$): (ppm) δ 7.23 (s, 1H), 6.77 (s, 1H), 4.19 – 4.15 (m, 1H), 3.94 – 3.92 (m, 7H), 3.51 – 3.35 (m, 2H), 2.47 – 2.44 (m, 1H), 2.36 – 2.27 (m, 2H), 2.14 – 2.03 (m, 1H), 1.75 – 1.66 (m, 1H).

^{13}C NMR (150 MHz, $CDCl_3$): (ppm) δ 150.41 (s), 146.86 (s), 146.75 (s), 135.78 (s), 128.24 (s), 126.76 (q, $J_{C\text{-}F}$ = 275.6 Hz), 101.39 (s), 92.21 (s), 56.37 (s), 56.20 (s), 42.34 (s), 37.12 (q, $J_{C\text{-}F}$ = 27.9 Hz), 31.35 (q, $J_{C\text{-}F}$ = 2.6 Hz), 26.66 (s), 21.65 (s).

^{19}F NMR (376 MHz, $CDCl_3$): (ppm) δ -63.93 (t, $J_{F\text{-}H}$ = 10.9 Hz).

HRMS (ESI): m/z calcd for $C_{15}H_{18}F_3N_2O_2$ $[M + H]^+$ 315.1315, found 315.1309.

7-fluoro-4-(2,2,2-trifluoroethyl)-1,2,3,4-tetrahydrobenzo[4,5]imidazo[1,2-a]pyridine (**7e**)

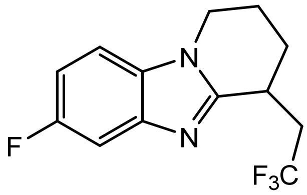

The desired pure product (56 mg, 69% yield) as a white crystal (mp 106 – 107 °C) was purified by flash column chromatography (PE/EA = 5/1).

^{1}H NMR (400 MHz, $CDCl_3$): (ppm) δ 7.62 (dd, J = 8.4, 4.8 Hz, 1H), 7.03 – 6.97 (m, 2H), 4.20 – 4.15 (m, 1H), 3.97 – 3.90 (m, 1H), 3.53 – 3.36 (m, 2H), 2.49 – 2.28 (m, 3H), 2.15 – 2.04 (m, 1H), 1.78 – 1.69 (m, 1H).

^{13}C NMR (150 MHz, $CDCl_3$): (ppm) δ 159.39 (d, $J_{C\text{-}F}$ = 238.2 Hz), 152.88 (d, $J_{C\text{-}F}$ = 2.7 Hz), 138.81 (s), 134.88 (d, $J_{C\text{-}F}$ = 12.9 Hz), 126.72 (q, $J_{C\text{-}F}$ = 275.6 Hz), 119.79 (d,

$J_{C\text{-}F}$ = 10.1 Hz), 110.57 (d, $J_{C\text{-}F}$ = 24.9 Hz), 95.85 (d, $J_{C\text{-}F}$ = 27.2 Hz), 42.51 (s), 37.05 (q, $J_{C\text{-}F}$ = 8.2 Hz), 31.52 (q, $J_{C\text{-}F}$ = 2.9 Hz), 26.63 (s), 21.59 (s).

^{19}F NMR (376 MHz, $CDCl_3$): (ppm) δ -63.93 (t, $J_{F\text{-}H}$ = 10.5 Hz), -119.73 – -119.79(m).

HRMS (ESI): m/z calcd for $C_{13}H_{12}F_4N_2Na$ $[M + Na]^+$ 295.0829, found 295.0819.

Methyl 4-(2,2,2-trifluoroethyl)-1,2,3,4-tetrahydrobenzo[4,5]imidazo[1,2-a]pyridine-8-carboxylate (**7f**)

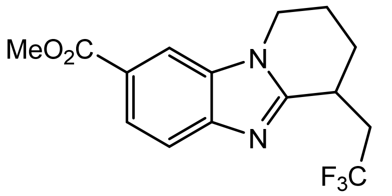

The desired pure product (63 mg, 67% yield) as a white crystal (mp 148 – 152 °C) was purified by flash column chromatography (PE/EA = 3/1).

^{1}H NMR (400 MHz, $CDCl_3$): (ppm) δ 8.43 (s, 1H), 7.99 (dd, J = 8.4, 1.6 Hz, 1H), 7.31 (d, J = 8.4 Hz, 1H), 4.29 – 4.24 (m, 1H), 4.04 – 3.97 (m, 1H), 3.94 (s, 3H), 3.55 – 3.39 (m, 2H), 2.51 – 2.48 (m, 1H), 2.42 – 2.29 (m, 2H), 2.17 – 2.05 (m, 1H), 1.81 – 1.71 (m, 1H).

^{13}C NMR (150 MHz, $CDCl_3$): (ppm) δ 167.59 (s), 154.03 (s), 142.09 (s), 138.00 (s), 126.68 (q, $J_{C\text{-}F}$ = 275.6 Hz), 124.65 (s), 123.98 (s), 121.50 (s), 108.72 (s), 52.06 (s), 42.63 (s), 37.03 (q, $J_{C\text{-}F}$ = 28.4 Hz), 31.60 (q, $J_{C\text{-}F}$ = 2.9 Hz), 26.51 (s), 21.56 (s).

^{19}F NMR (376 MHz, $CDCl_3$): (ppm) δ -68.63 (t, $J_{F\text{-}H}$ = 10.5 Hz).

HRMS (ESI): m/z calcd for $C_{15}H_{15}F_3N_2NaO_2$ $[M + Na]^+$ 335.0978, found 335.0976.

3-(2,2,2-trifluoroethyl)-2,3-dihydro-1H-benzo[*d*]pyrrolo[1,2-*a*]imidazole (**7g**)

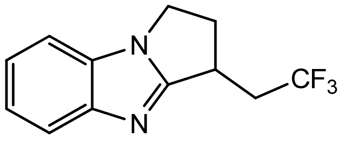

The desired pure product (19 mg, 26% yield) as a white crystal (mp 94 – 98 °C) was purified by flash column chromatography (PE/EA = 4/1).

^{1}H NMR (400 MHz, $CDCl_3$): (ppm) δ 7.72 (t, J = 7.2 Hz, 1H),7.33 – 7.30 (m, 1H), 7.26 – 7.23 (m, 2H), 4.21 – 4.16 (m, 1H), 4.06 – 3.99 (m, 1H), 3.61 – 3.55 (m, 1H), 3.22 – 3.10 (m, 1H), 3.05 – 2.98 (m, 1H), 2.55 – 2.46 (m, 1H), 2.37 – 2.23 (m, 1H).

^{13}C NMR (150 MHz, $CDCl_3$): (ppm) δ 160.54 (s), 148.33 (s), 132.31 (s), 126.39 (q, J_{C-F} = 275.6 Hz), 122.36 (s), 122.05 (s), 119.81 (s), 109.65 (s), 41.98 (s), 36.80 (q, J_{C-F} = 28.5 Hz), 33.65 (s), 30.78 (q, J_{C-F} = 3.0 Hz).

^{19}F NMR (376 MHz, $CDCl_3$): (ppm) δ -65.38 (t, J_{F-H} = 11.3 Hz).

HRMS (ESI): m/z calcd for $C_{12}H_{12}F_3N_2$ $[M + H]^+$ 241.0947, found 241.0945.

3-methyl-3-(2,2,2-trifluoroethyl)-2,3-dihydro-1H-benzo[*d*]pyrrolo[1,2-*a*]imidazole (**7h**)

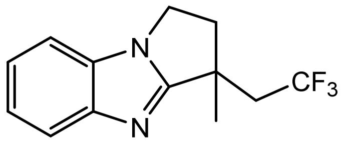

The desired pure product (41 mg, 54% yield) as a white crystal (mp 76 – 77 °C) was purified by flash column chromatography (PE/EA = 4/1).

^{1}H NMR (400 MHz, $CDCl_3$): (ppm) δ 7.74 – 7.70 (m, 1H), 7.34 – 7.31 (m, 1H), 7.27 – 7.23 (m, 2H), 4.18 – 4.07 (m, 2H), 2.94 – 2.82 (m, 2H), 2.65 – 2.54 (m, 2H), 1.55 (s, 3H).

^{13}C NMR (150 MHz, $CDCl_3$): (ppm) δ 164.28 (s), 148.26 (s), 131.98 (s), 126.28 (q, J_{C-F} = 276.5 Hz), 122.31 (s), 122.00 (s), 119.89 (s), 109.71 (s), 41.93 (q, J_{C-F} = 27.5 Hz), 41.05 (s), 39.87 (s), 37.29 (q, J_{C-F} = 2.0 Hz), 24.63 (s).

^{19}F NMR (376 MHz, $CDCl_3$): (ppm) δ -61.23 (t, J_{F-H} = 11.3 Hz).

HRMS (ESI): m/z calcd for $C_{13}H_{14}F_3N_2$ $[M + H]^+$ 255.1104, found 255.1100.

6-(2,2,2-trifluoroethyl)-6,7,8,9-tetrahydroimidazo[1,2-*a*:5,4-*b*']dipyridine (**7i**)

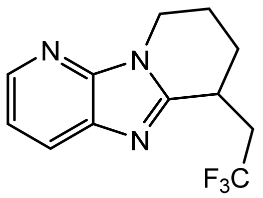

The desired pure product (48 mg, 63% yield) as a white crystal (mp 115 – 116 °C) was purified by flash column chromatography (PE/EA = 2/1).

^{1}H NMR (400 MHz, $CDCl_3$): (ppm) δ 8.35 (d, J = 4.8 Hz, 1H), 7.97 (dd, J = 8.0, 1.2 Hz, 1H), 7.23 (dd, J = 8.0, 4.8 Hz, 1H), 4.51 – 4.46 (m, 1H), 4.08 – 4.01 (m, 1H), 3.53 – 3.40 (m, 2H), 2.52 – 2.30 (m, 3H), 2.14 – 2.03 (m, 1H), 1.82 – 1.72 (m, 1H).

^{13}C NMR (150 MHz, $CDCl_3$): (ppm) δ 153.62 (s), 147.77 (s), 143.44 (s), 134.76 (s), 126.70 (q, $J_{C\text{-}F}$ = 275.7 Hz), 126.57 (s), 118.49 (s), 41.45 (s), 36.91 (q, $J_{C\text{-}F}$ = 28.4 Hz), 31.73 (q, $J_{C\text{-}F}$ = 2.9 Hz), 26.65 (s), 21.48 (s).

^{19}F NMR (376 MHz, $CDCl_3$): (ppm) δ -63.93 (t, $J_{F\text{-}H}$ = 10.5 Hz).

HRMS (ESI): m/z calcd for $C_{12}H_{13}F_3N_3$ $[M + H]^+$ 256.1056, found 256.1063.

1,3-dimethyl-9-(2,2,2-trifluoroethyl)-6,7,8,9-tetrahydropyrido[2,1-*f*]purine-2,4(1H,3H)-dione (**7j**)

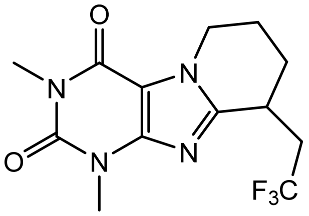

The desired pure product (66 mg, 70% yield) as a white crystal (mp 147 – 151 °C) was purified by flash column chromatography (PE/EA = 3/1).

^{1}H NMR (400 MHz, $CDCl_3$): (ppm) δ 4.58 – 4.54 (m, 1H), 4.16 – 4.11 (m, 1H), 3.55 (s, 3H), 3.38 (s, 3H), 3.31 – 3.22 (m, 2H), 2.41 – 2.39 (m, 1H), 2.33 – 2.19 (m, 2H), 2.05 – 1.97 (m, 1H), 1.74 – 1.67 (m, 1H).

^{13}C NMR (150 MHz, $CDCl_3$): (ppm) δ 155.07 (s), 151.66 (s), 150.58 (s), 148.08 (s), 126.44 (q, $J_{C\text{-}F}$ = 275.9 Hz), 106.99 (s), 44.70 (s), 36.93 (q, $J_{C\text{-}F}$ = 28.2 Hz), 30.94 (q, $J_{C\text{-}F}$ = 2.9 Hz), 29.68 (s), 27.79 (s), 25.83 (s), 21.24 (s).

^{19}F NMR (376 MHz, $CDCl_3$): (ppm) δ -63.86 (t, $J_{F\text{-}H}$ = 11.3 Hz).

HRMS (ESI): m/z calcd for $C_{13}H_{15}F_3N_4NaO_2$ $[M + Na]^+$ 339.1039, found 339.1048.

7. Reference

[1] Yang J.; Sun B.; Ding H.; Huang P.-Y.; Tang X.-L.; Shi R.-C.; Yan Z.-Y.; Yu C.-M.; Jin C. *Green Chem.* **2021**, *23*, 575-581.

[2] Yang R.; Yi D.; Shen K.; Fu Q.; Wei J.; Lu J.; Yang L.; Wang L.; Wei S.; Zhang Z. *Org. Lett.* **2022**, *24*, 2014-2019.

[3] Wang Y.-X.; Qi S.-L.; Luan Y.-X.; Han X.-W.; Wang S.; Chen H.; Ye M. *J. Am. Chem. Soc.* **2018**, *140*, 5360-5364.

[4] Sun B.; Huang P.; Yan Z.; Shi X.; Tang X.; Yang J.; Jin C. *Org. Lett.* **2021**, *23*, 1026-1031.

[5] Zheng J.; Deng Z.; Zhang Y.; Cui S. *Adv. Syn. & Catal.* **2016**, *358*, 746-751.

[6] Lin S.; Cui J.; Chen Y.; Li Y. *J. Org. Chem.* **2021**, *86*, 15768-15776.

8. The ^{1}H, ^{13}C and ^{19}F NMR spectra copy of compounds

6-(2,2,2-trifluoroethyl)-6,7,8,9-tetrahydro-11H-pyrido[2,1-b]quinazolin-11-one (**3a**) :
^{1}H NMR (400 MHz, CDCl$_3$)

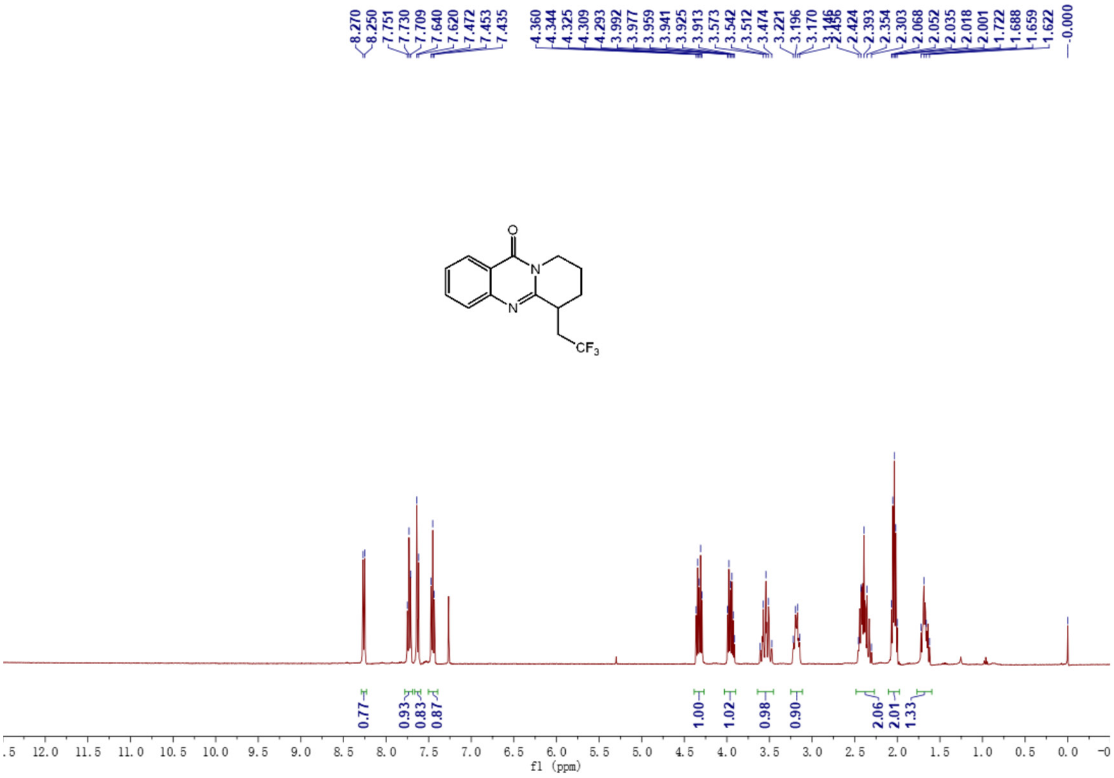

6-(2,2,2-trifluoroethyl)-6,7,8,9-tetrahydro-11H-pyrido[2,1-b]quinazolin-11-one (**3a**) :
^{13}C NMR (150 MHz, CDCl$_3$)

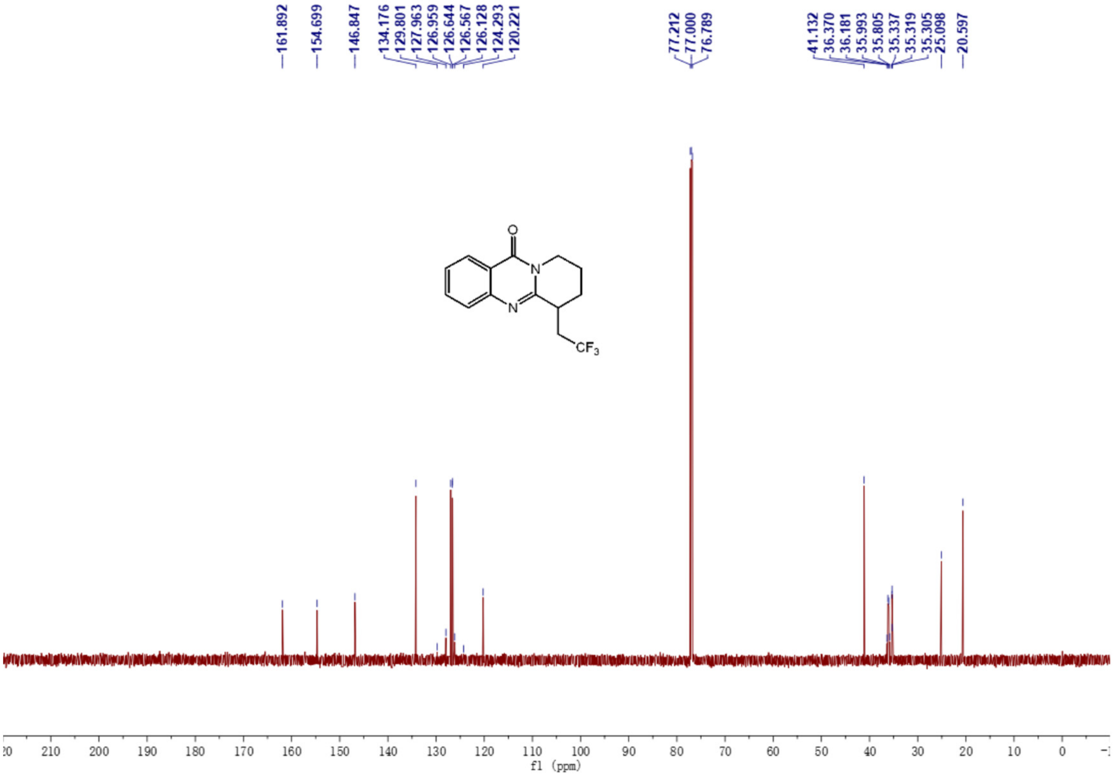

6-(2,2,2-trifluoroethyl)-6,7,8,9-tetrahydro-11H-pyrido[2,1-b]quinazolin-11-one (**3a**) :

^{19}F NMR (376 MHz, $CDCl_3$)

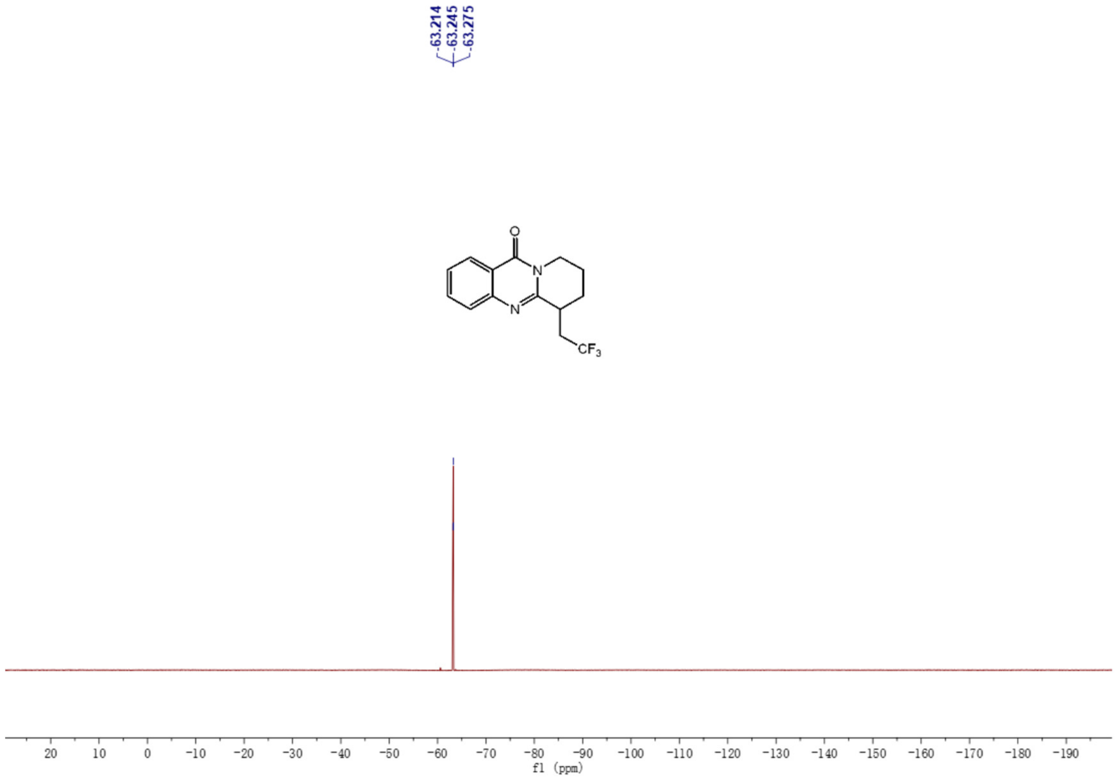

1-methyl-6-(2,2,2-trifluoroethyl)-6,7,8,9-tetrahydro-11H-pyrido[2,1-*b*]quinazolin-11-one (**3b**): ^{1}H NMR (400 MHz, $CDCl_3$)

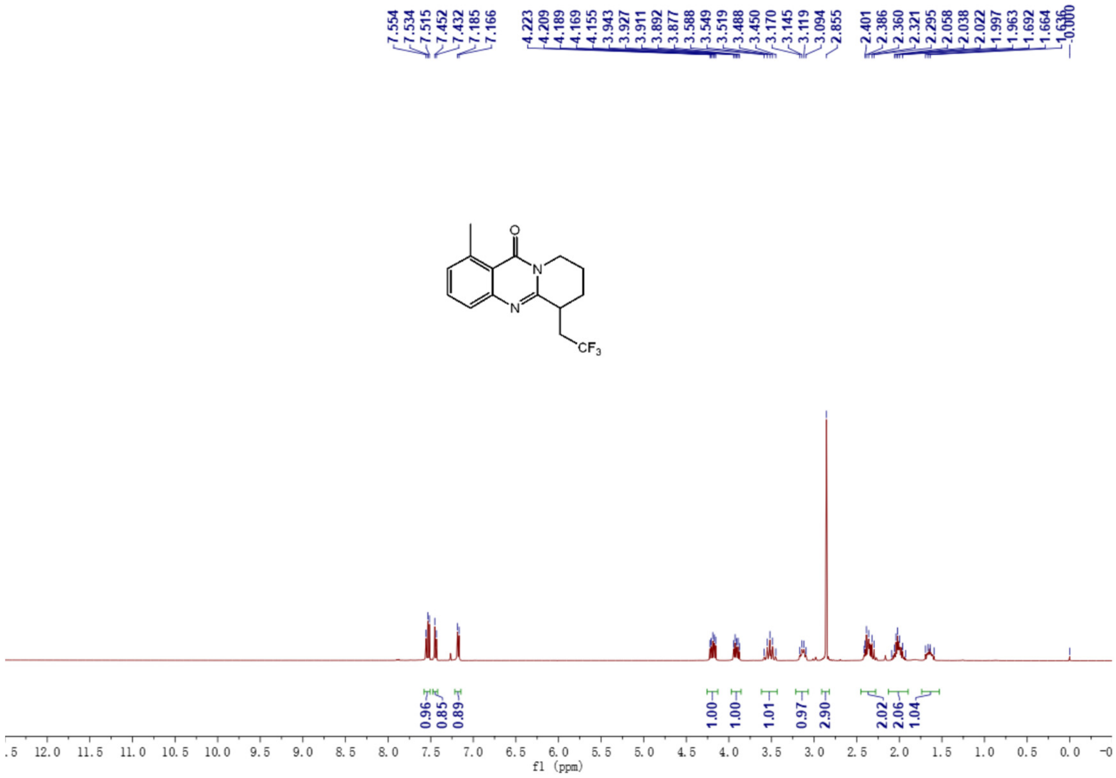

1-methyl-6-(2,2,2-trifluoroethyl)-6,7,8,9-tetrahydro-11H-pyrido[2,1-*b*]quinazolin-11-one (**3b**): ^{13}C NMR (150 MHz, $CDCl_3$)

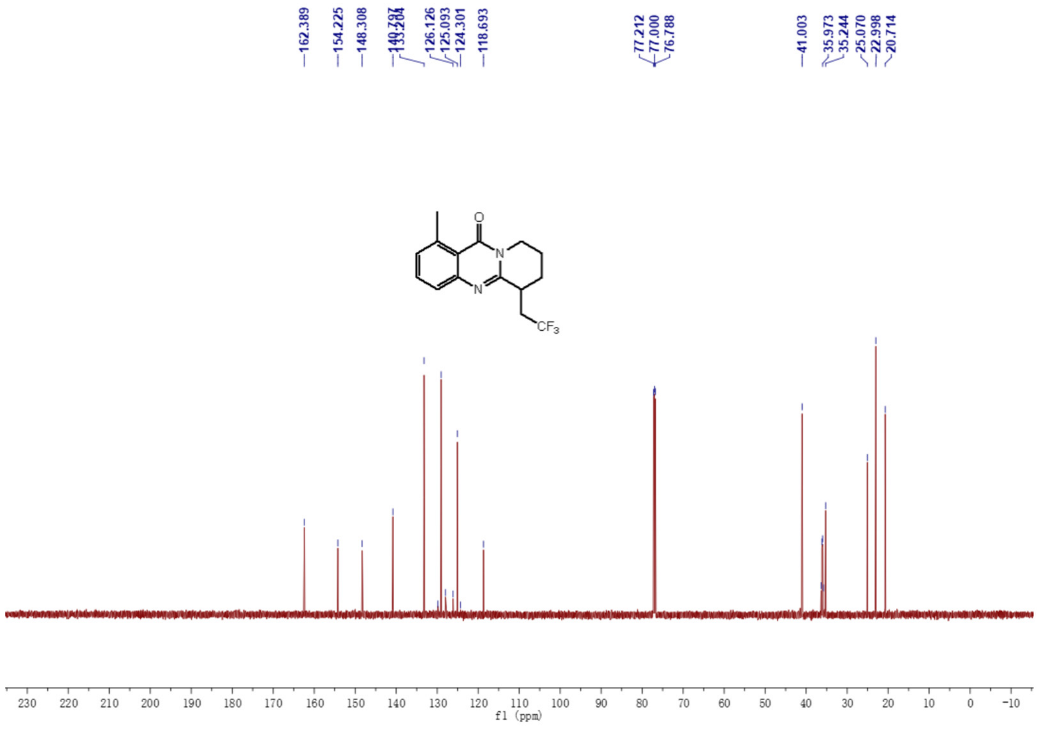

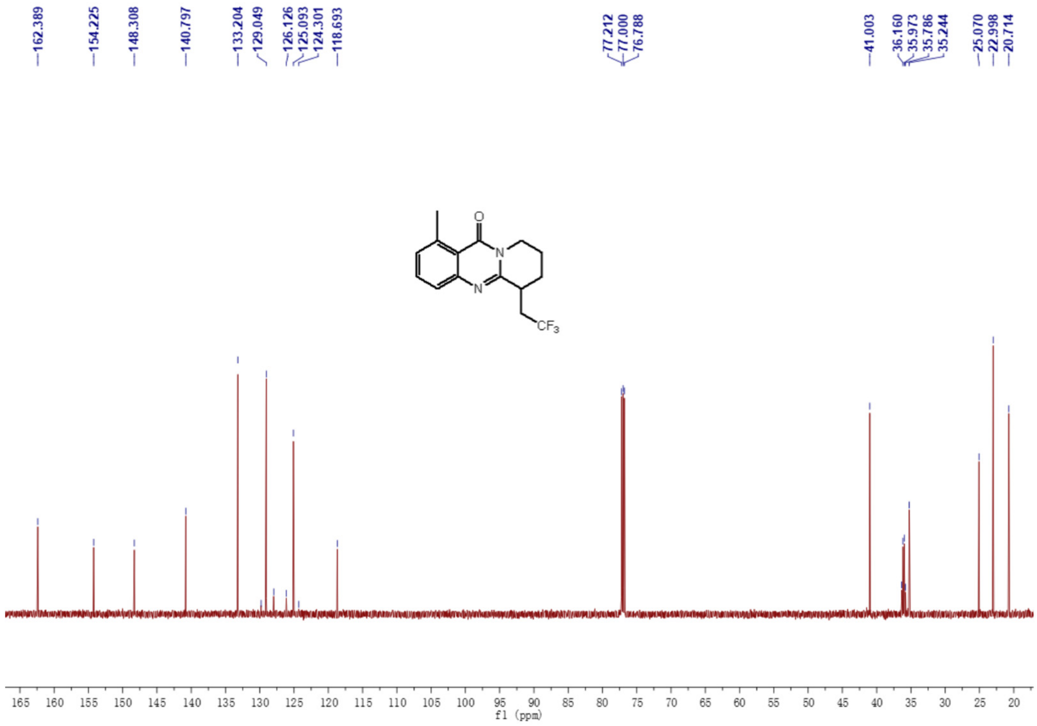

1-methyl-6-(2,2,2-trifluoroethyl)-6,7,8,9-tetrahydro-11H-pyrido[2,1-*b*]quinazolin-11-one (**3b**): ^{19}F NMR (376 MHz, $CDCl_3$)

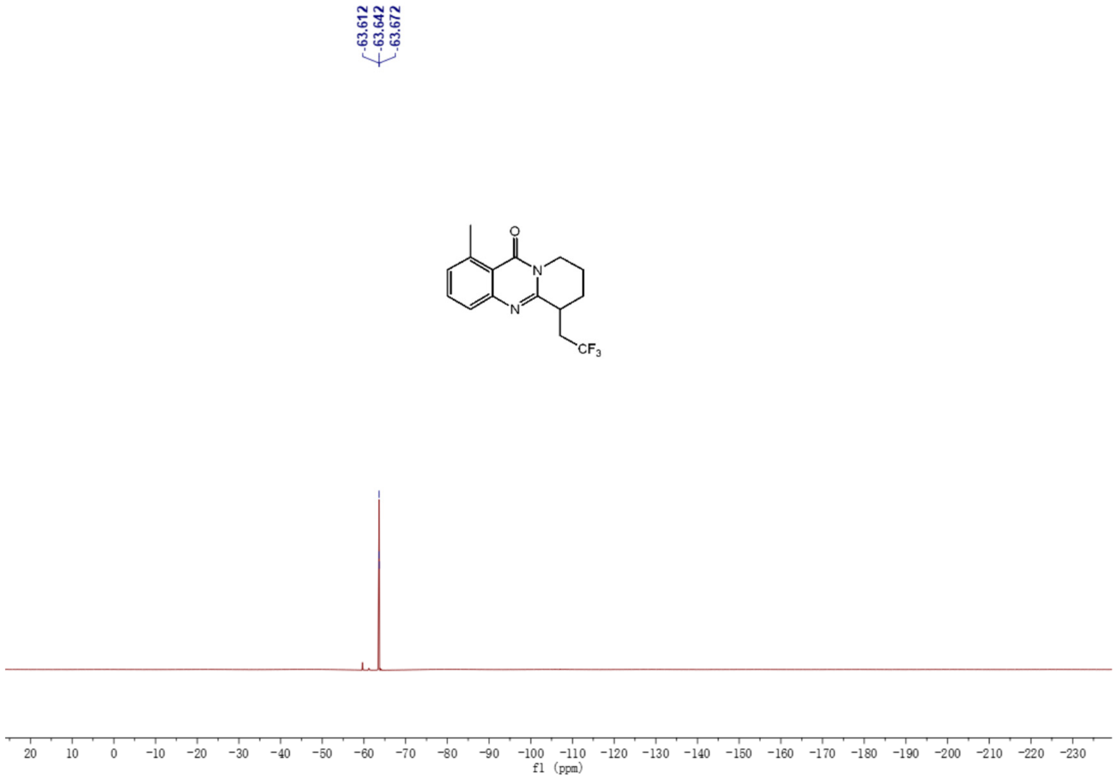

1-chloro-6-(2,2,2-trifluoroethyl)-6,7,8,9-tetrahydro-11H-pyrido[2,1-*b*]quinazolin-11-one (**3c**): ^{1}H NMR (400 MHz, $CDCl_3$)

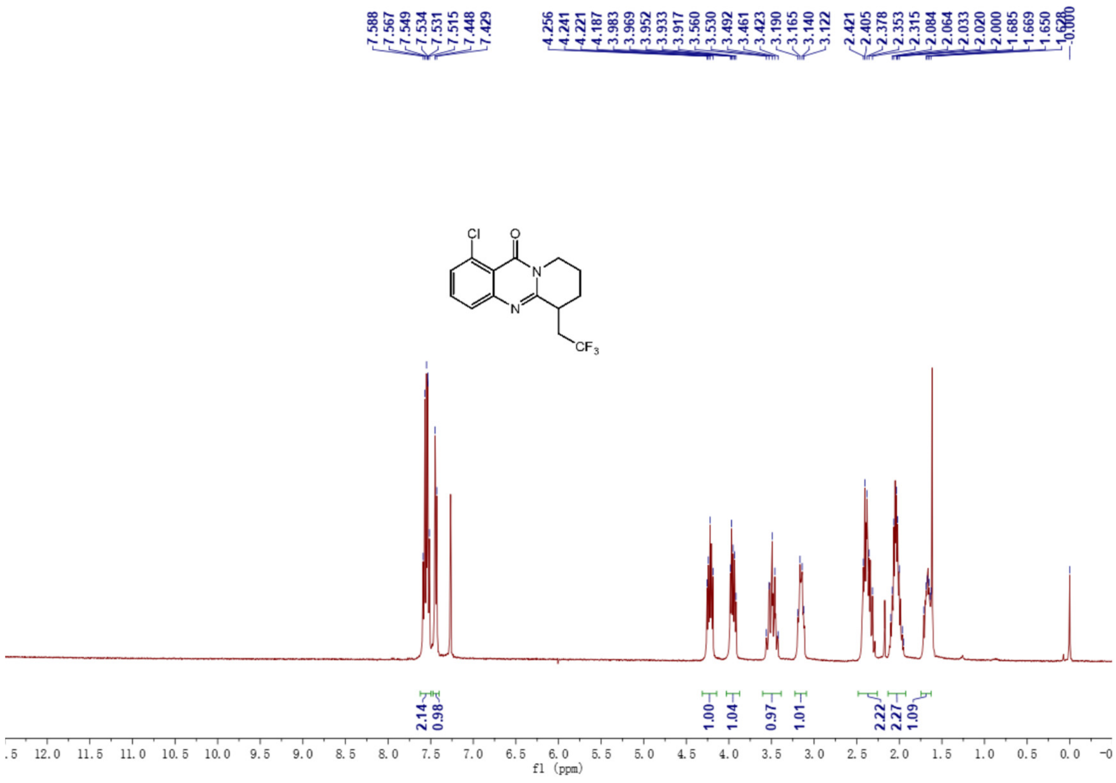

1-chloro-6-(2,2,2-trifluoroethyl)-6,7,8,9-tetrahydro-11H-pyrido[2,1-*b*]quinazolin-11-one (**3c**): ^{13}C NMR (150 MHz, $CDCl_3$)

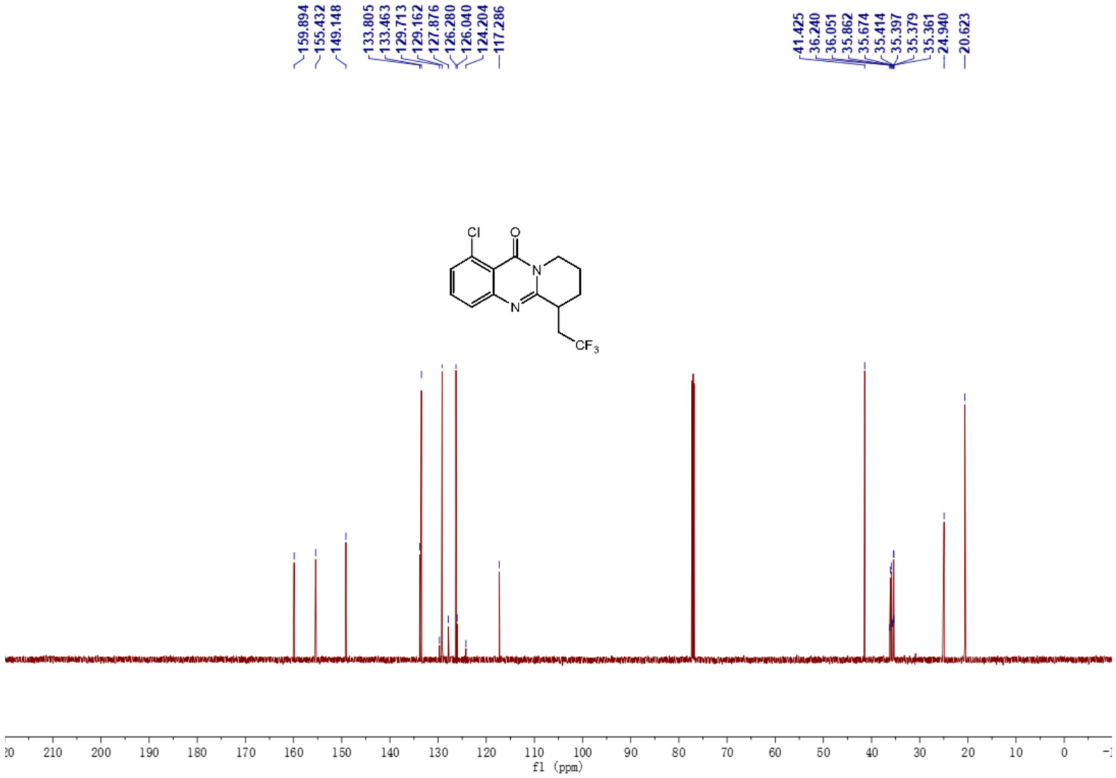

1-chloro-6-(2,2,2-trifluoroethyl)-6,7,8,9-tetrahydro-11H-pyrido[2,1-*b*]quinazolin-11-one (**3c**): ^{19}F NMR (376 MHz, $CDCl_3$)

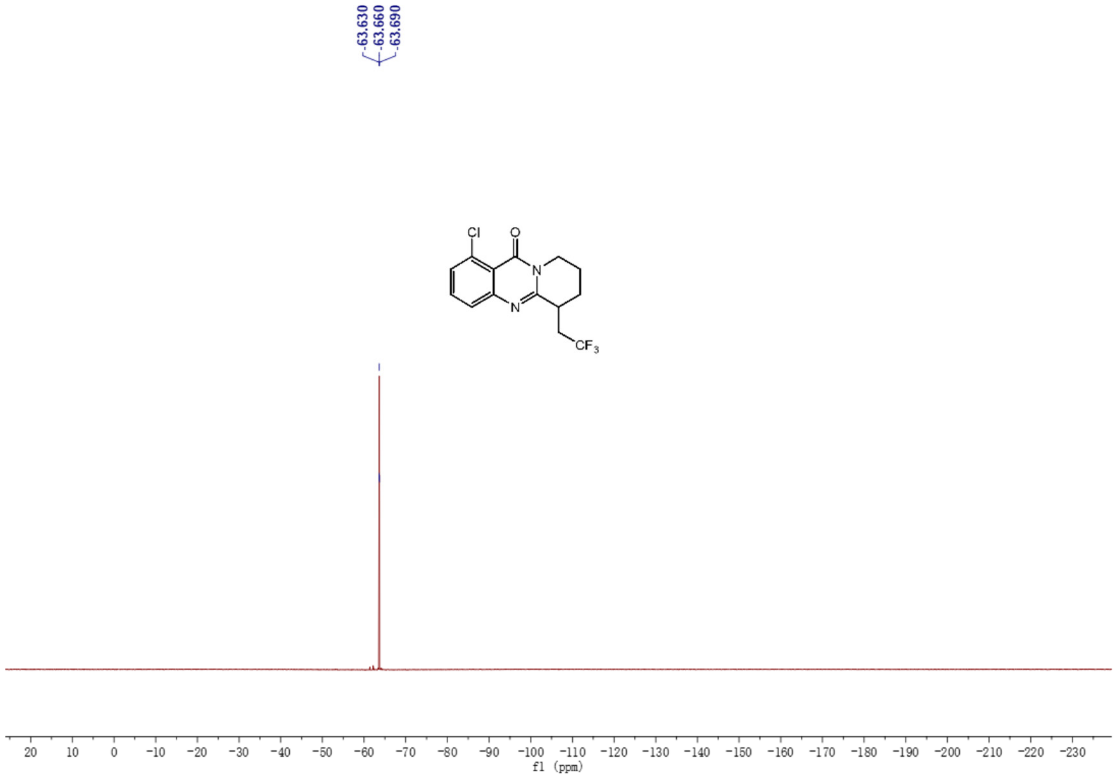

2-methoxy-6-(2,2,2-trifluoroethyl)-6,7,8,9-tetrahydro-11H-pyrido[2,1-*b*]quinazolin-11-one (**3d**) : ^{1}H NMR (400 MHz, $CDCl_3$)

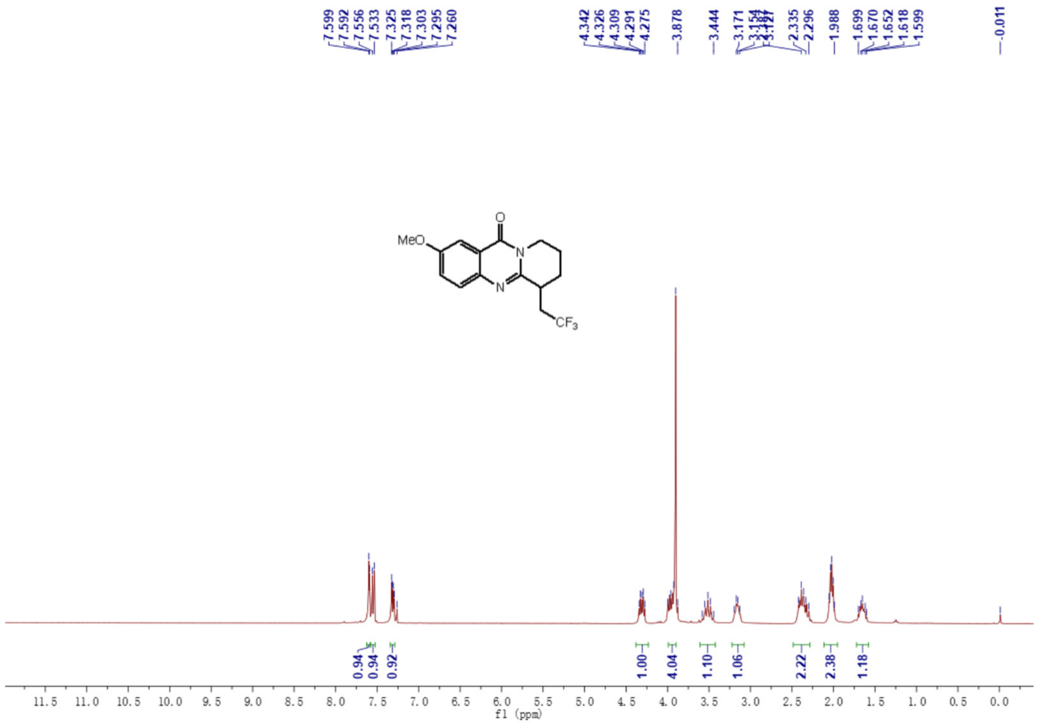

2-methoxy-6-(2,2,2-trifluoroethyl)-6,7,8,9-tetrahydro-11H-pyrido[2,1-*b*]quinazolin-11-one (**3d**) **:** ^{13}C NMR (150 MHz, $CDCl_3$)

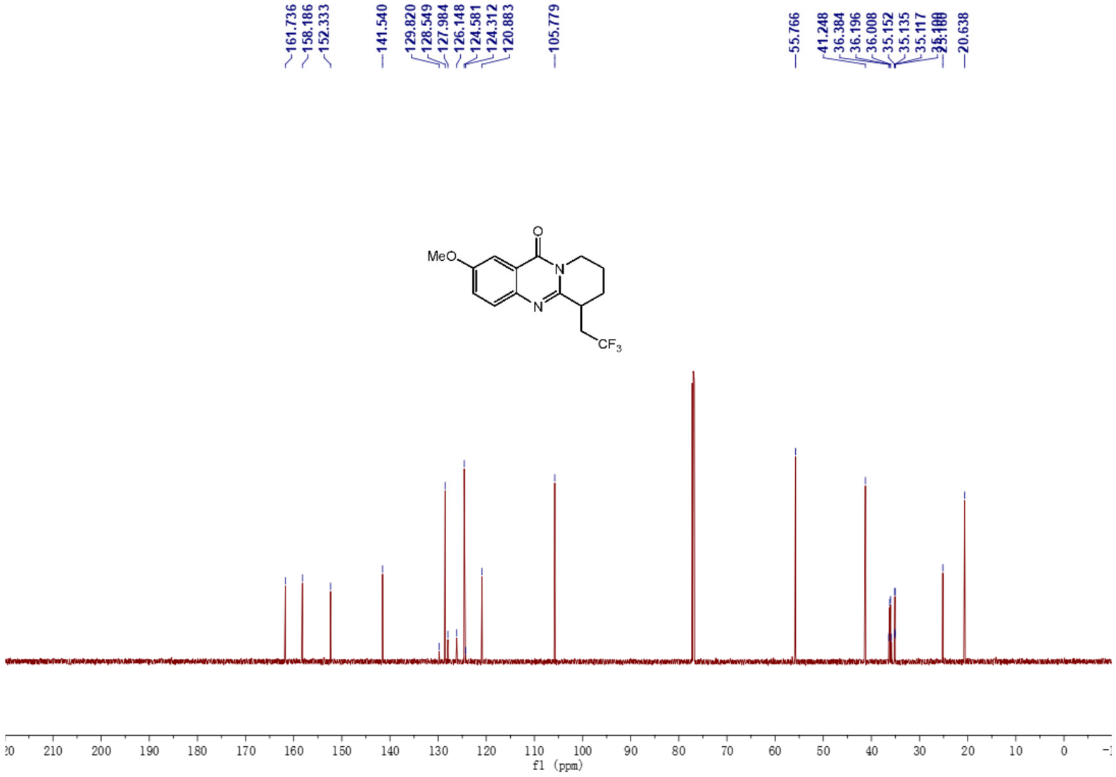

2-methoxy-6-(2,2,2-trifluoroethyl)-6,7,8,9-tetrahydro-11H-pyrido[2,1-*b*]quinazolin-11-one (**3d**) **:** ^{19}F NMR (376 MHz, $CDCl_3$)

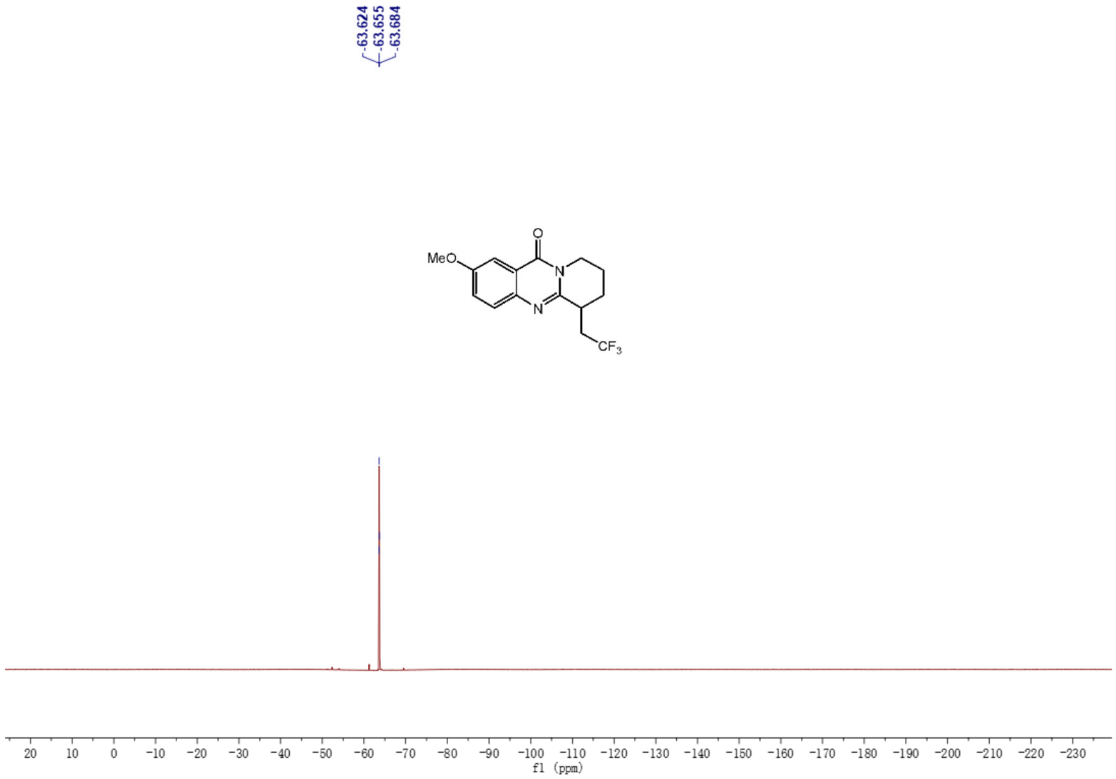

2-methyl-6-(2,2,2-trifluoroethyl)-6,7,8,9-tetrahydro-11H-pyrido[2,1-*b*]quinazolin-11-one (**3e**) : ^{1}H NMR (400 MHz, $CDCl_3$)

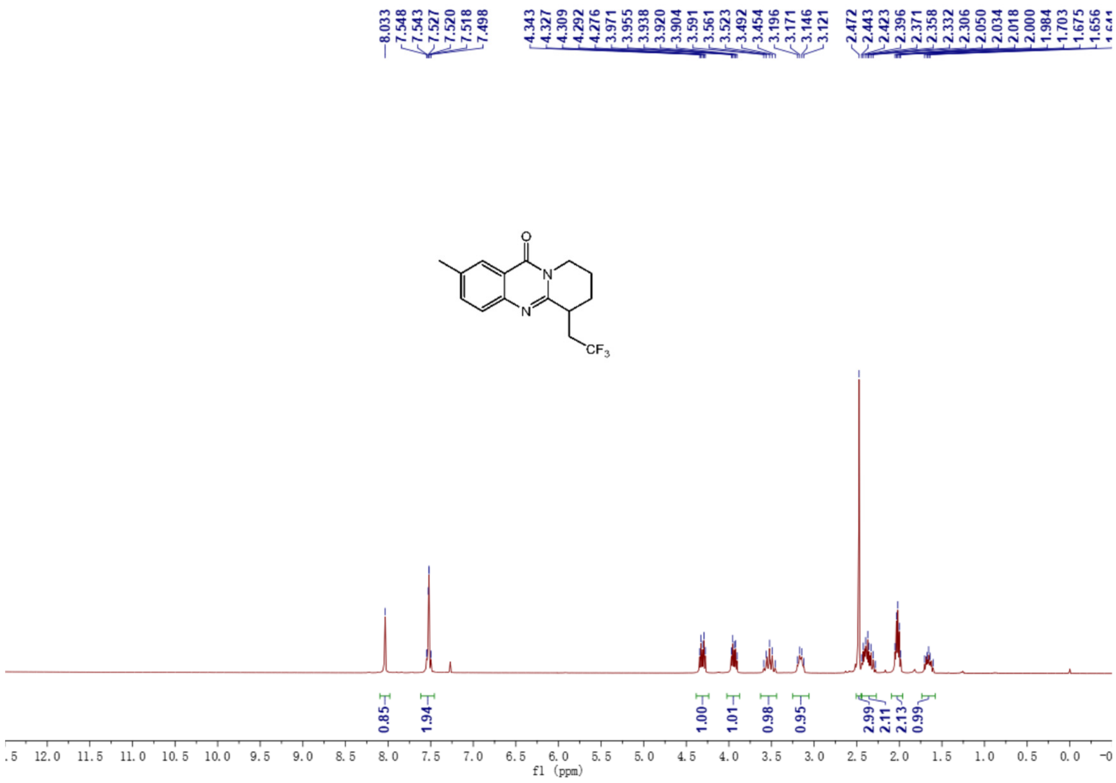

2-methyl-6-(2,2,2-trifluoroethyl)-6,7,8,9-tetrahydro-11H-pyrido[2,1-*b*]quinazolin-11-one (**3e**) : ^{13}C NMR (150 MHz, CDCl$_3$)

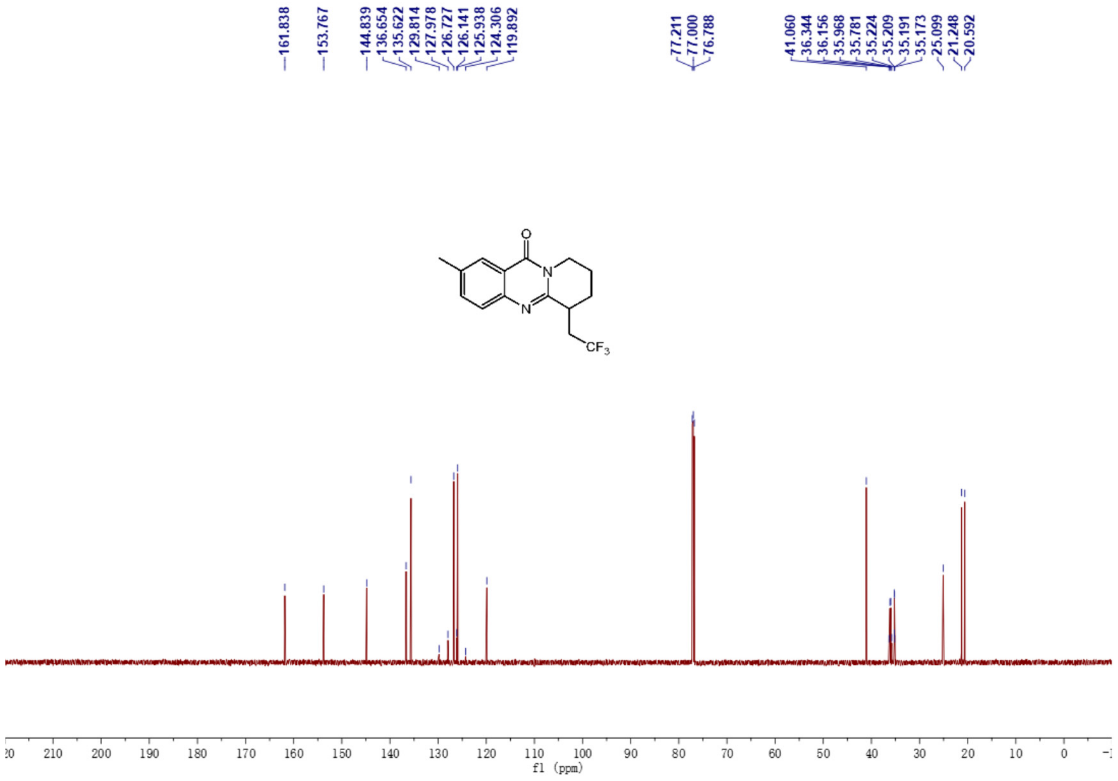

2-methyl-6-(2,2,2-trifluoroethyl)-6,7,8,9-tetrahydro-11H-pyrido[2,1-*b*]quinazolin-11-one (**3e**) : ^{19}F NMR (376 MHz, CDCl$_3$)

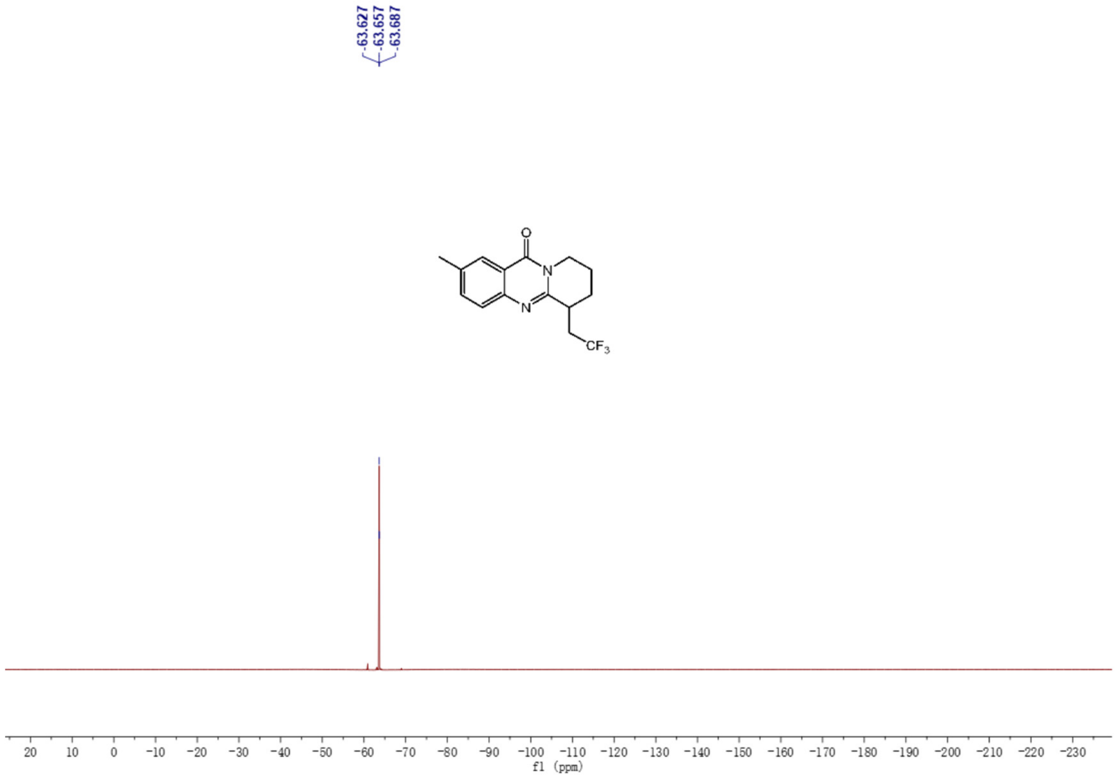

2-fluoro-6-(2,2,2-trifluoroethyl)-6,7,8,9-tetrahydro-11H-pyrido[2,1-*b*]quinazolin-11-one (**3f**) : ^{1}H NMR (400 MHz, $CDCl_3$)

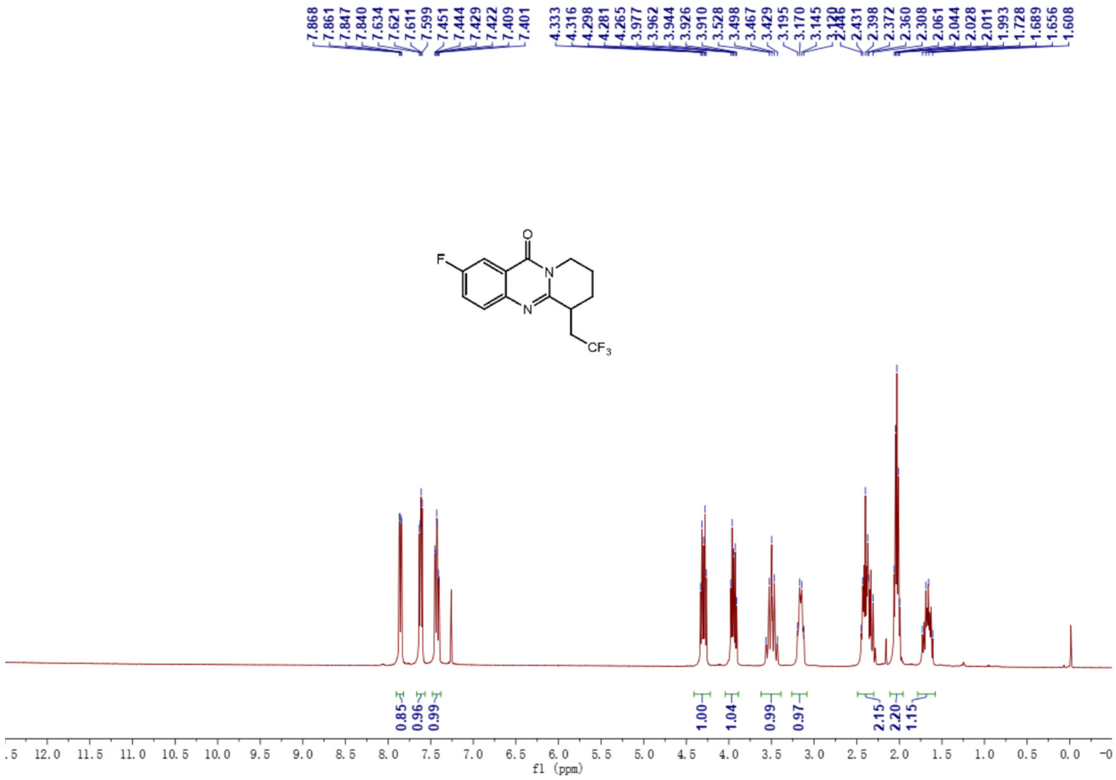

2-fluoro-6-(2,2,2-trifluoroethyl)-6,7,8,9-tetrahydro-11H-pyrido[2,1-*b*]quinazolin-11-one (**3f**) **:** ^{13}C NMR (150 MHz, $CDCl_3$)

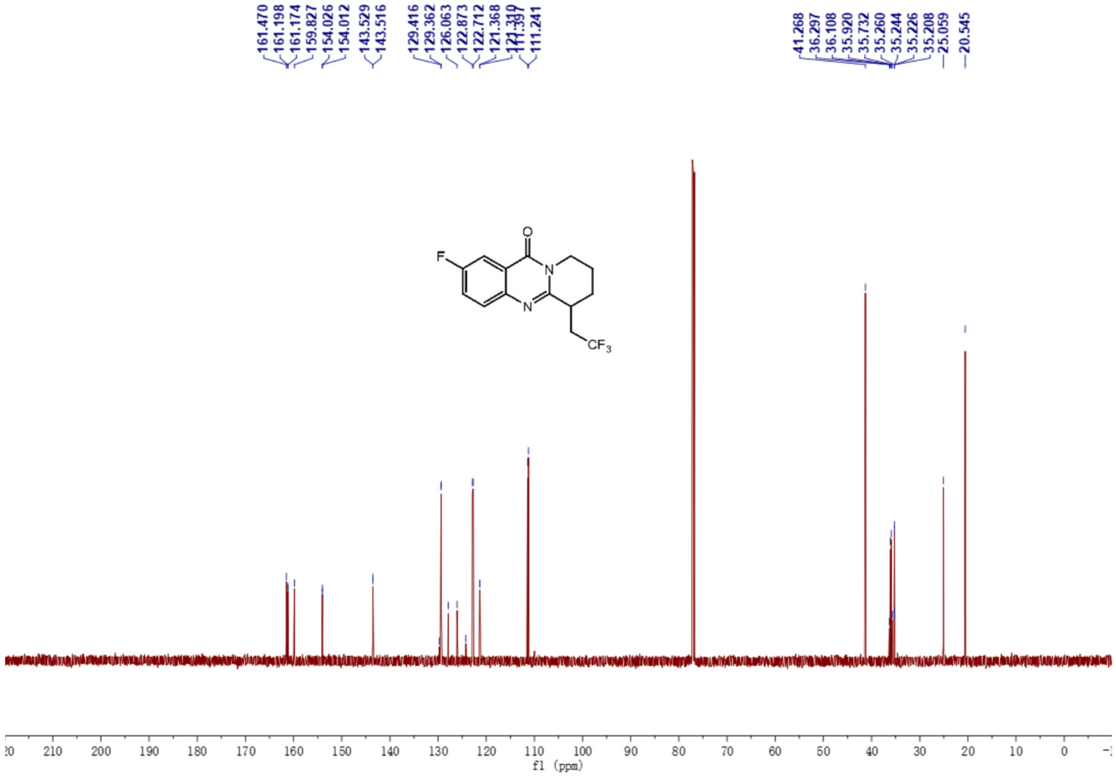

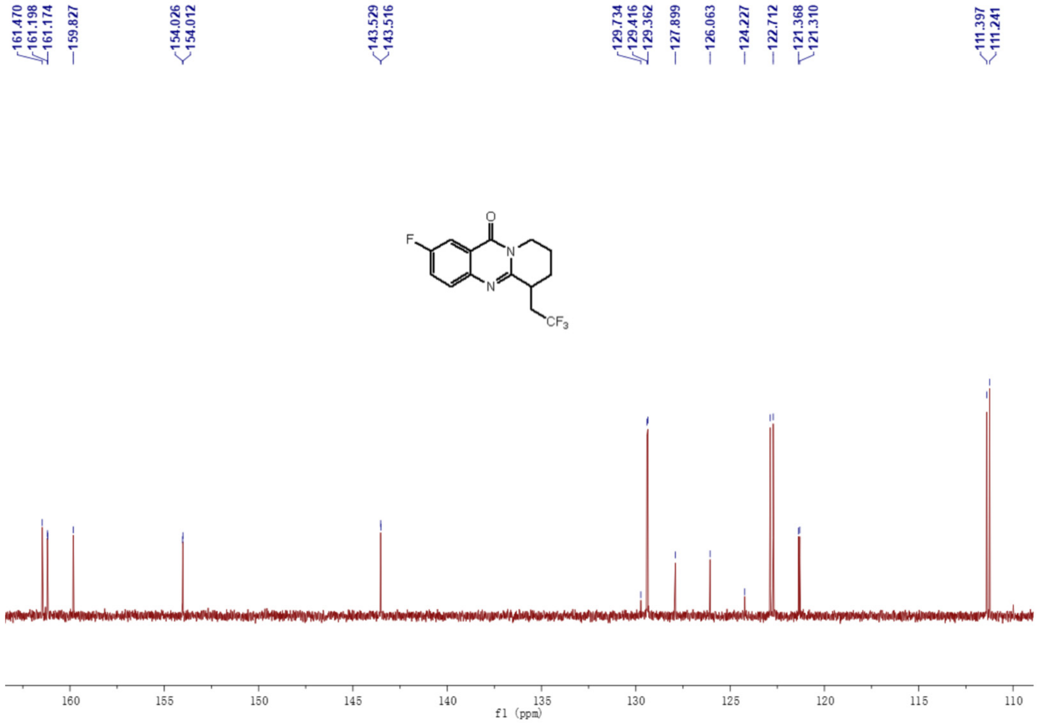

2-fluoro-6-(2,2,2-trifluoroethyl)-6,7,8,9-tetrahydro-11H-pyrido[2,1-*b*]quinazolin-11-one (**3f**) **:** ^{19}F NMR (376 MHz, $CDCl_3$)

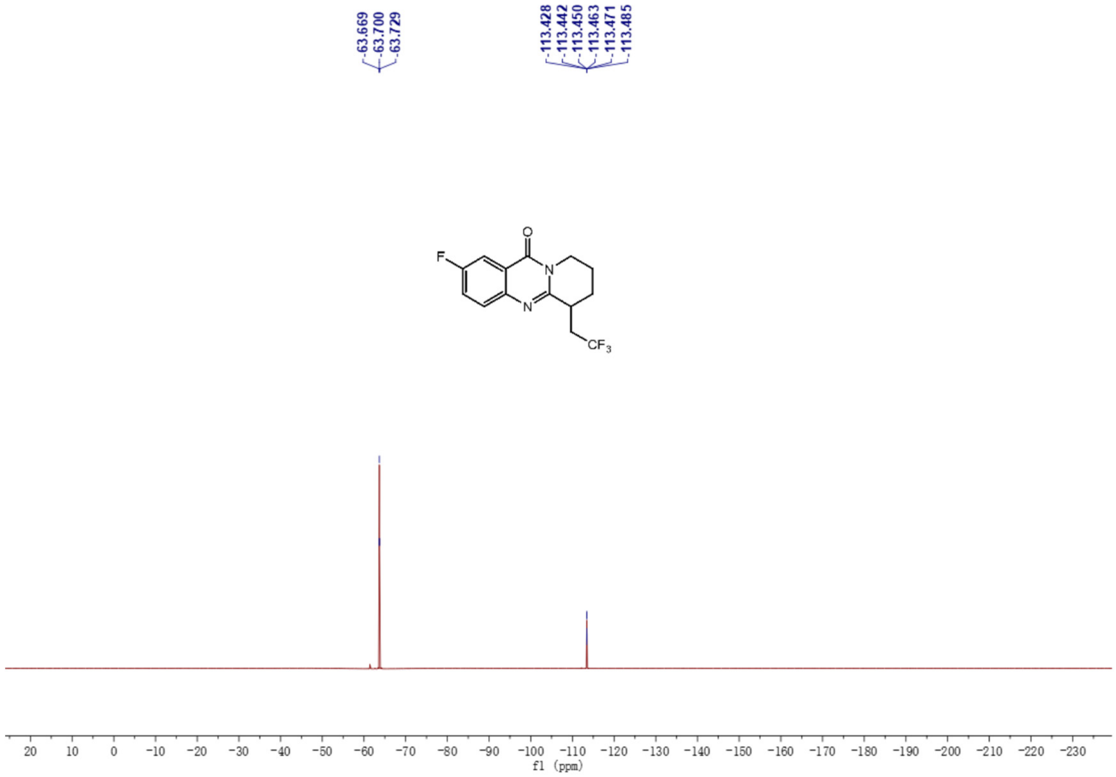

2-chloro-6-(2,2,2-trifluoroethyl)-6,7,8,9-tetrahydro-11H-pyrido[2,1-*b*]quinazolin-11-one (**3g**) : ^{1}H NMR (400 MHz, $CDCl_3$)

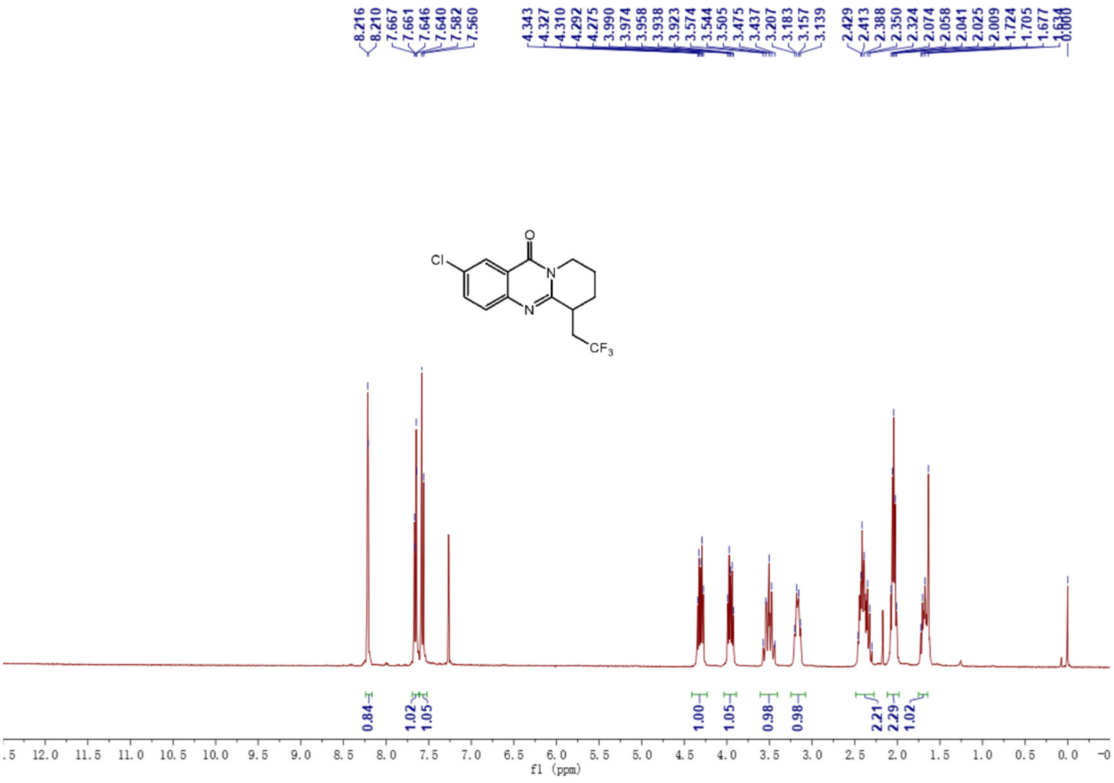

2-chloro-6-(2,2,2-trifluoroethyl)-6,7,8,9-tetrahydro-11H-pyrido[2,1-*b*]quinazolin-11-one (**3g**) : ^{13}C NMR (150 MHz, $CDCl_3$)

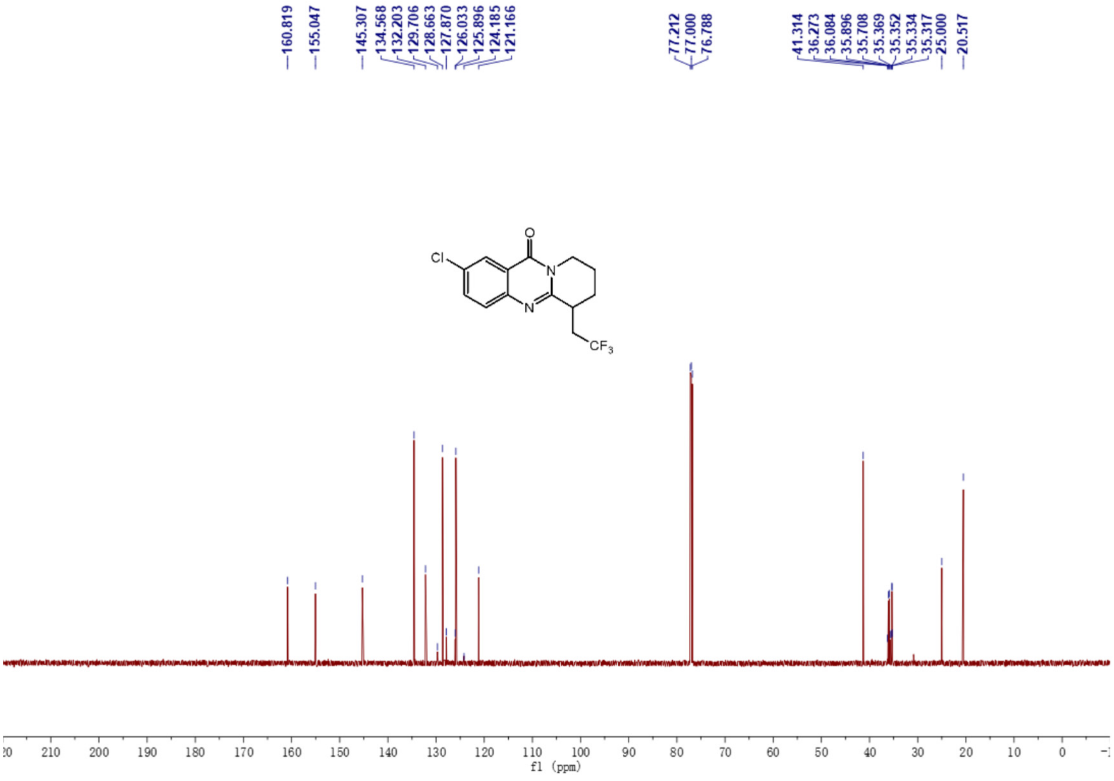

2-chloro-6-(2,2,2-trifluoroethyl)-6,7,8,9-tetrahydro-11H-pyrido[2,1-*b*]quinazolin-11-one (**3g**) : ^{19}F NMR (376 MHz, $CDCl_3$)

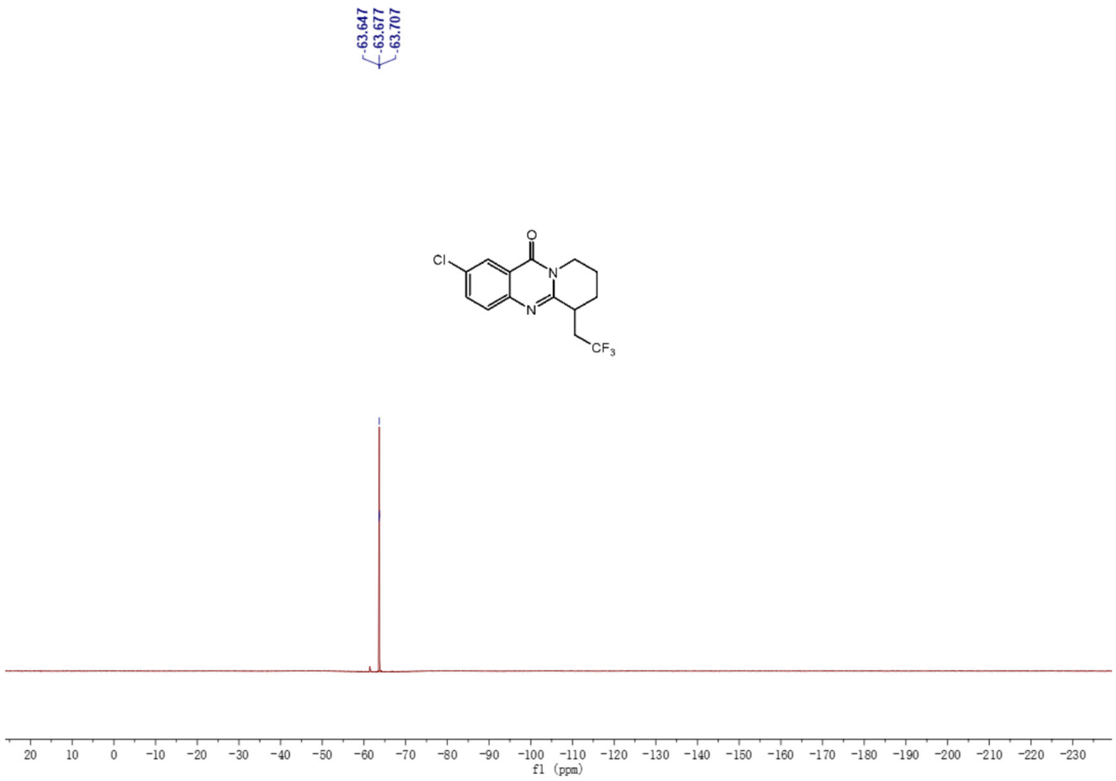

3-methyl-6-(2,2,2-trifluoroethyl)-6,7,8,9-tetrahydro-11H-pyrido[2,1-*b*]quinazolin-11-one (**3h**) : ^{1}H NMR (400 MHz, $CDCl_3$)

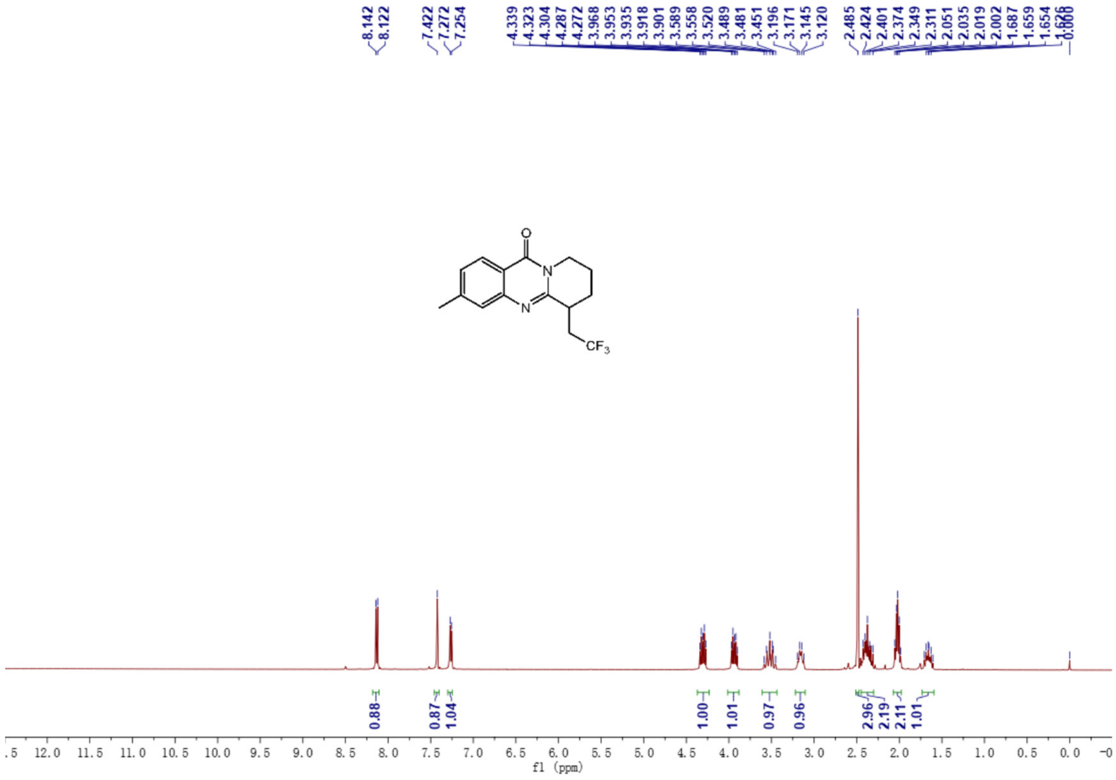

3-methyl-6-(2,2,2-trifluoroethyl)-6,7,8,9-tetrahydro-11H-pyrido[2,1-*b*]quinazolin-11-one (**3h**) : ^{13}C NMR (150 MHz, $CDCl_3$)

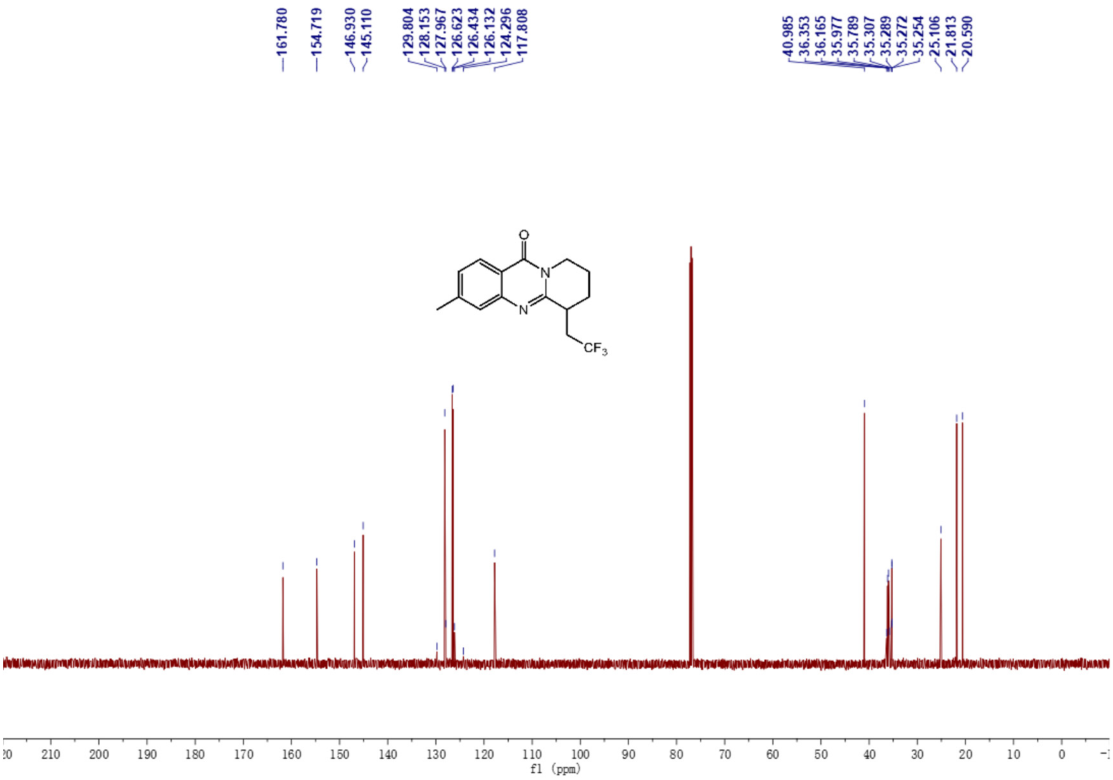

3-methyl-6-(2,2,2-trifluoroethyl)-6,7,8,9-tetrahydro-11H-pyrido[2,1-*b*]quinazolin-11-one (**3h**) : ^{19}F NMR (376 MHz, $CDCl_3$)

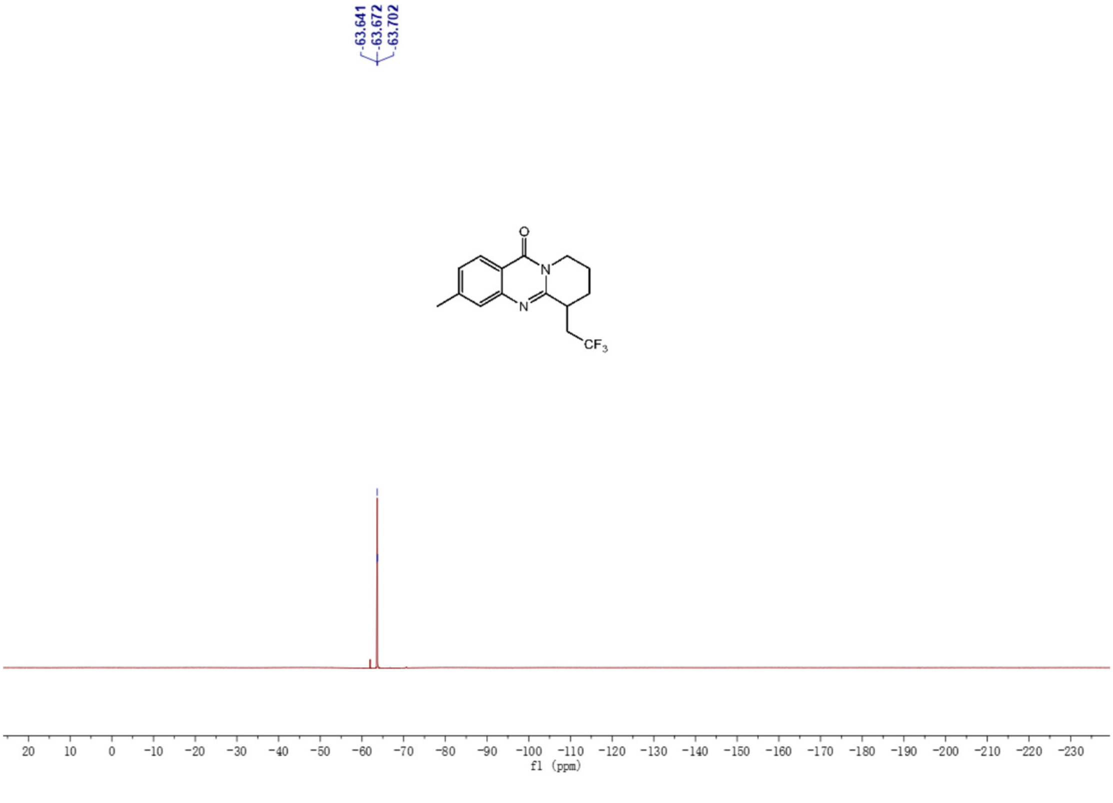

3-methoxy-6-(2,2,2-trifluoroethyl)-6,7,8,9-tetrahydro-11H-pyrido[2,1-*b*]quinazolin-11-one (**3i**) : ^{1}H NMR (400 MHz, $CDCl_3$)

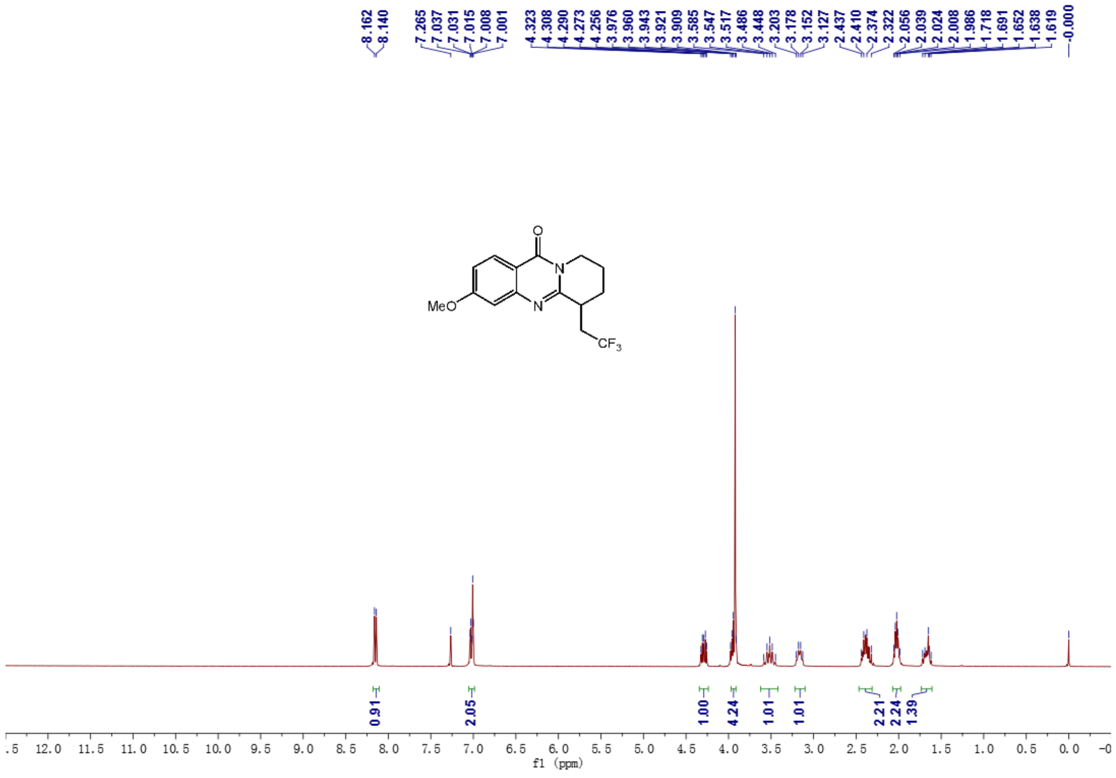

3-methoxy-6-(2,2,2-trifluoroethyl)-6,7,8,9-tetrahydro-11H-pyrido[2,1-*b*]quinazolin-11-one (**3i**) : ^{13}C NMR (150 MHz, $CDCl_3$)

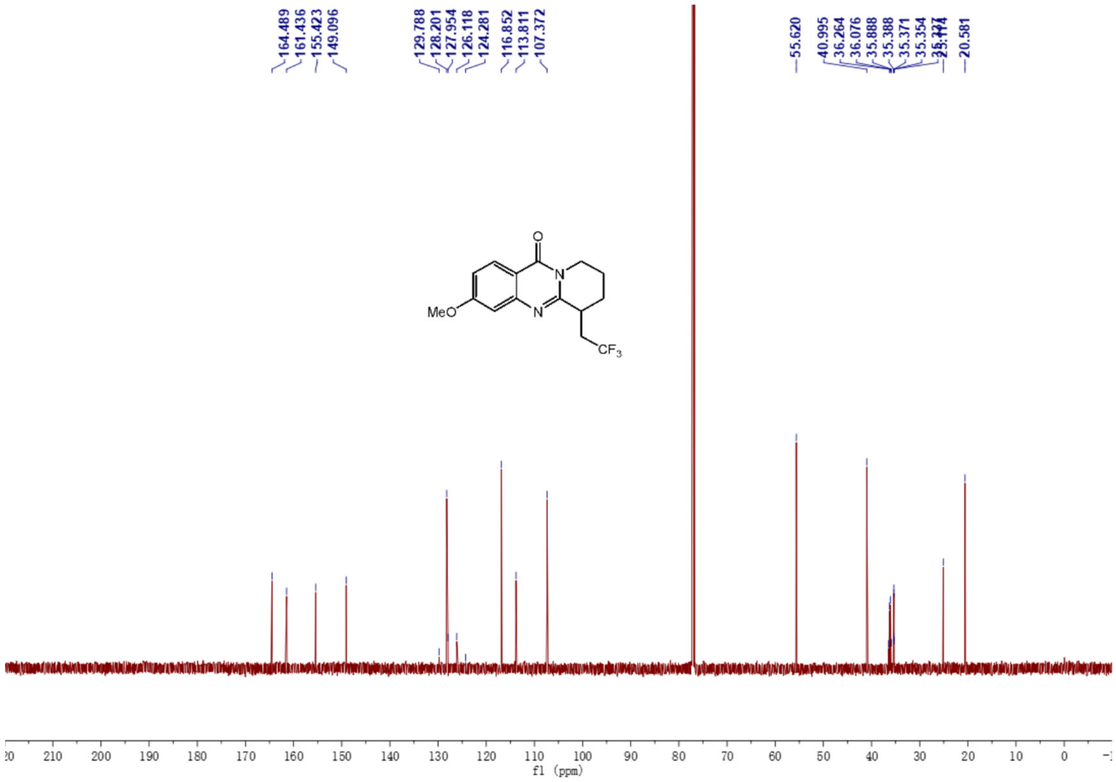

3-methoxy-6-(2,2,2-trifluoroethyl)-6,7,8,9-tetrahydro-11H-pyrido[2,1-*b*]quinazolin-11-one (**3i**) : ^{19}F NMR (376 MHz, $CDCl_3$)

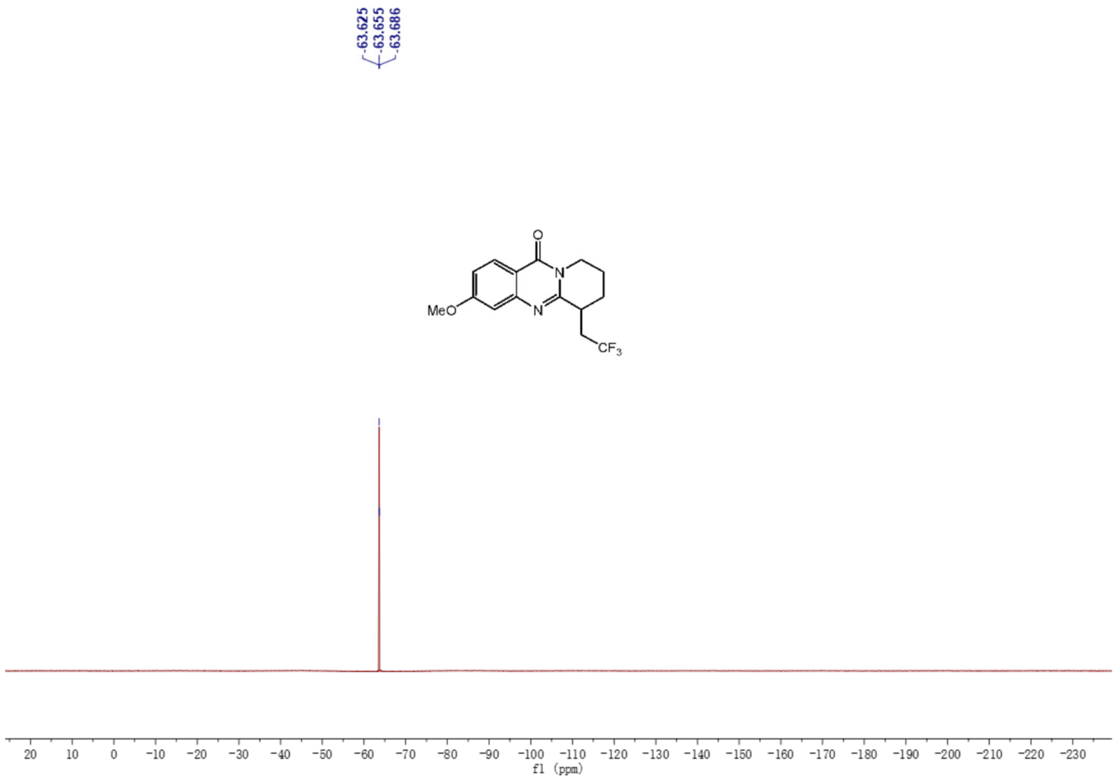

3-fluoro-6-(2,2,2-trifluoroethyl)-6,7,8,9-tetrahydro-11H-pyrido[2,1-*b*] quinazolin-11-one (**3j**) : ^{1}H NMR (400 MHz, CDCl$_3$)

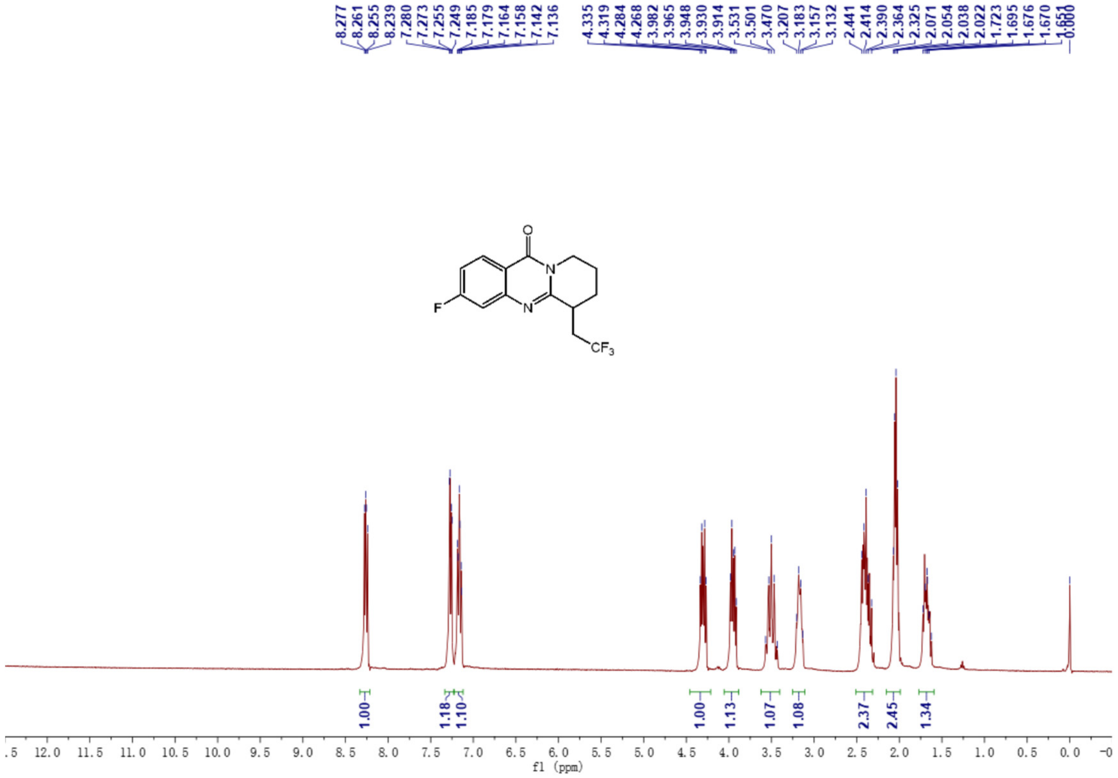

3-fluoro-6-(2,2,2-trifluoroethyl)-6,7,8,9-tetrahydro-11H-pyrido[2,1-*b*] quinazolin-11-one (**3j**) : ^{13}C NMR (150 MHz, CDCl$_3$)

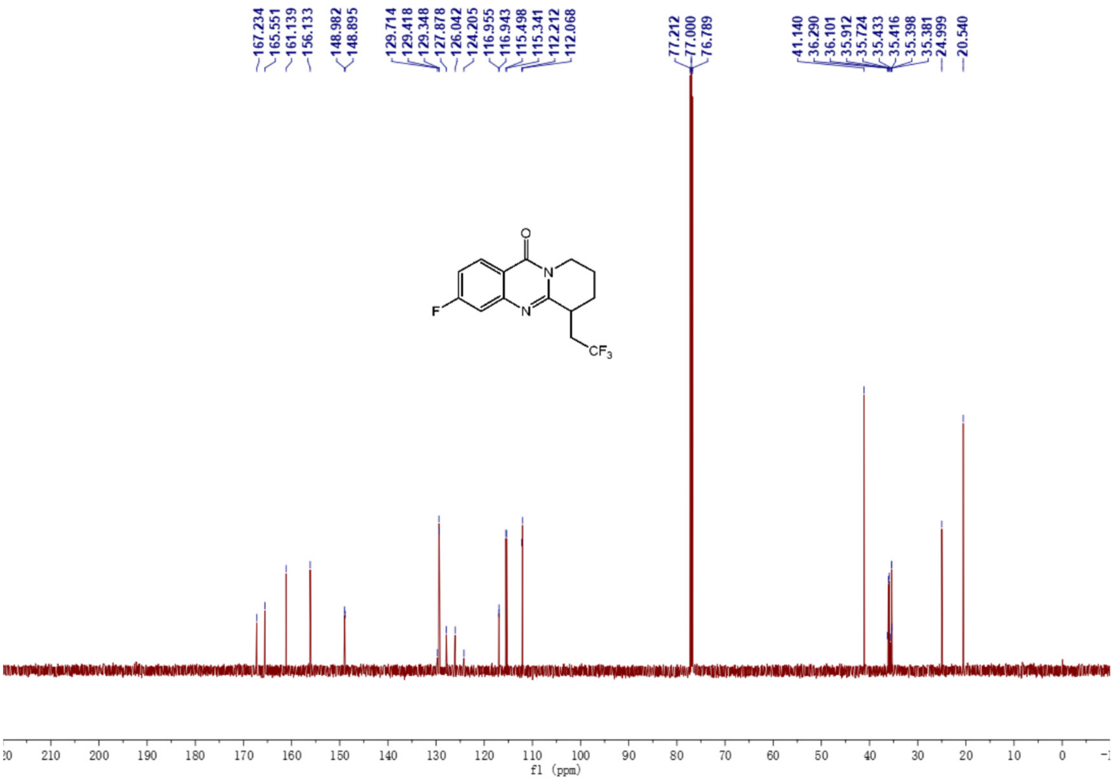

3-fluoro-6-(2,2,2-trifluoroethyl)-6,7,8,9-tetrahydro-11H-pyrido[2,1-*b*] quinazolin-11-one (**3j**) : ^{19}F NMR (376 MHz, $CDCl_3$)

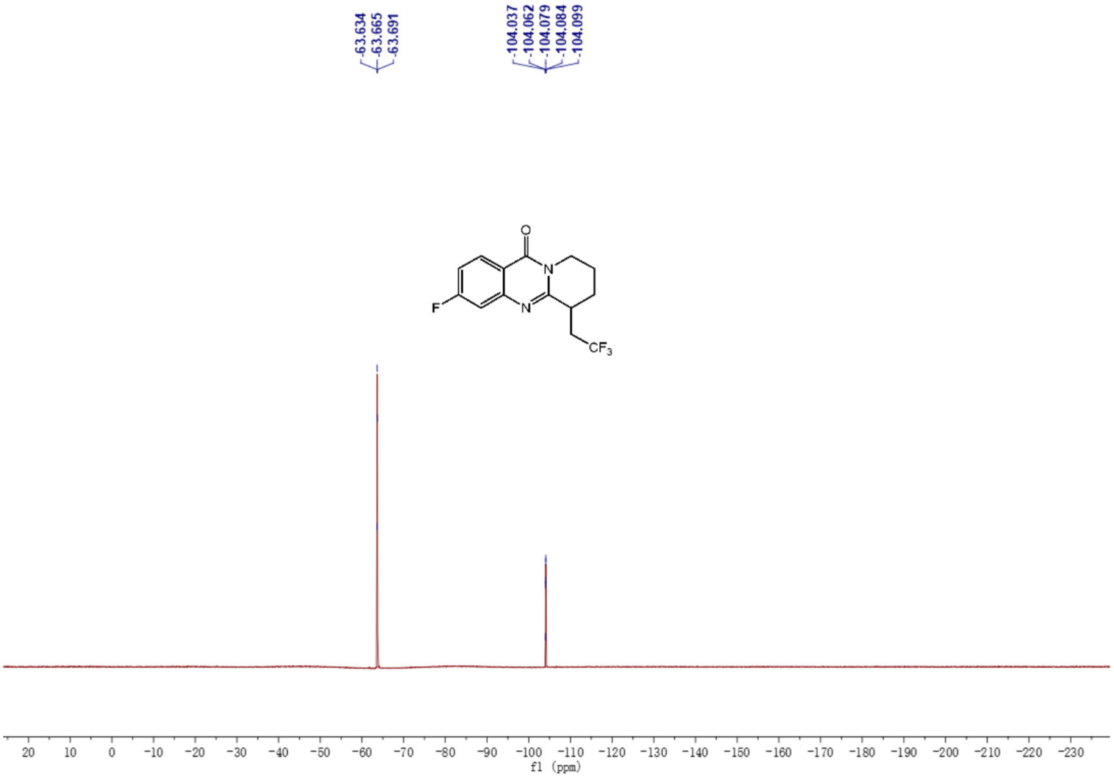

3-chloro-6-(2,2,2-trifluoroethyl)-6,7,8,9-tetrahydro-11H-pyrido[2,1-*b*]quinazolin-11-one (**3k**) : ^{1}H NMR (400 MHz, $CDCl_3$)

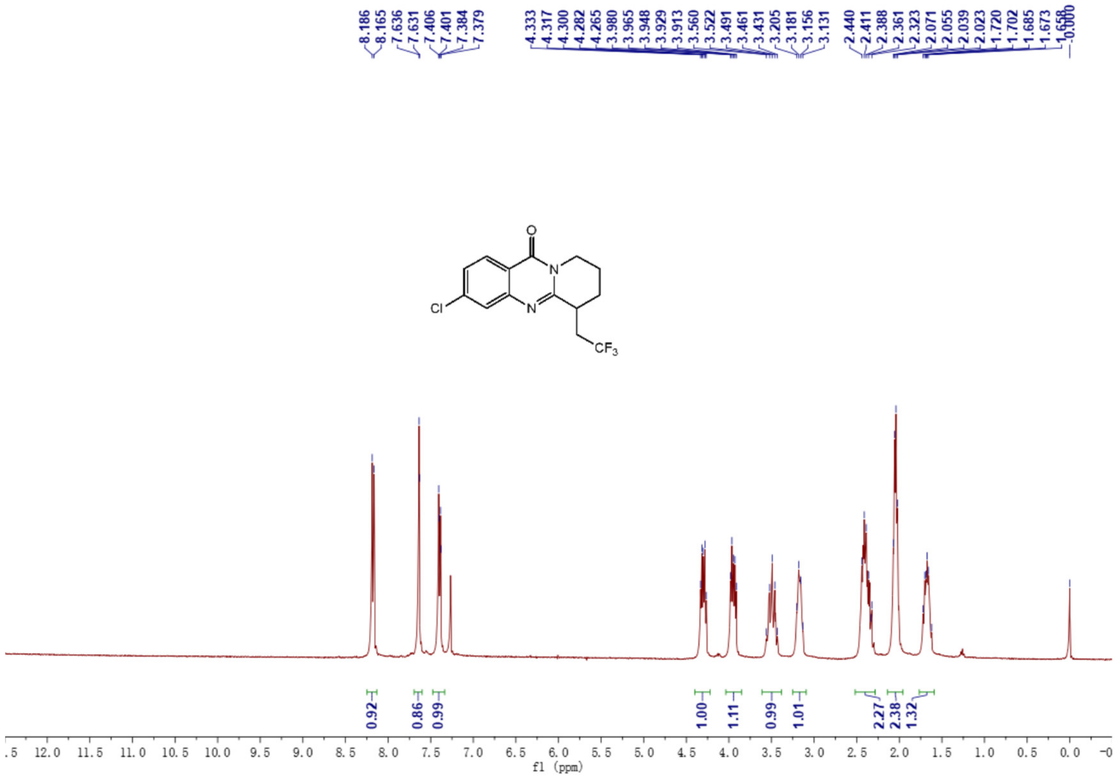

3-chloro-6-(2,2,2-trifluoroethyl)-6,7,8,9-tetrahydro-11H-pyrido[2,1-*b*]quinazolin-11-one (**3k**) : ^{13}C NMR (150 MHz, $CDCl_3$)

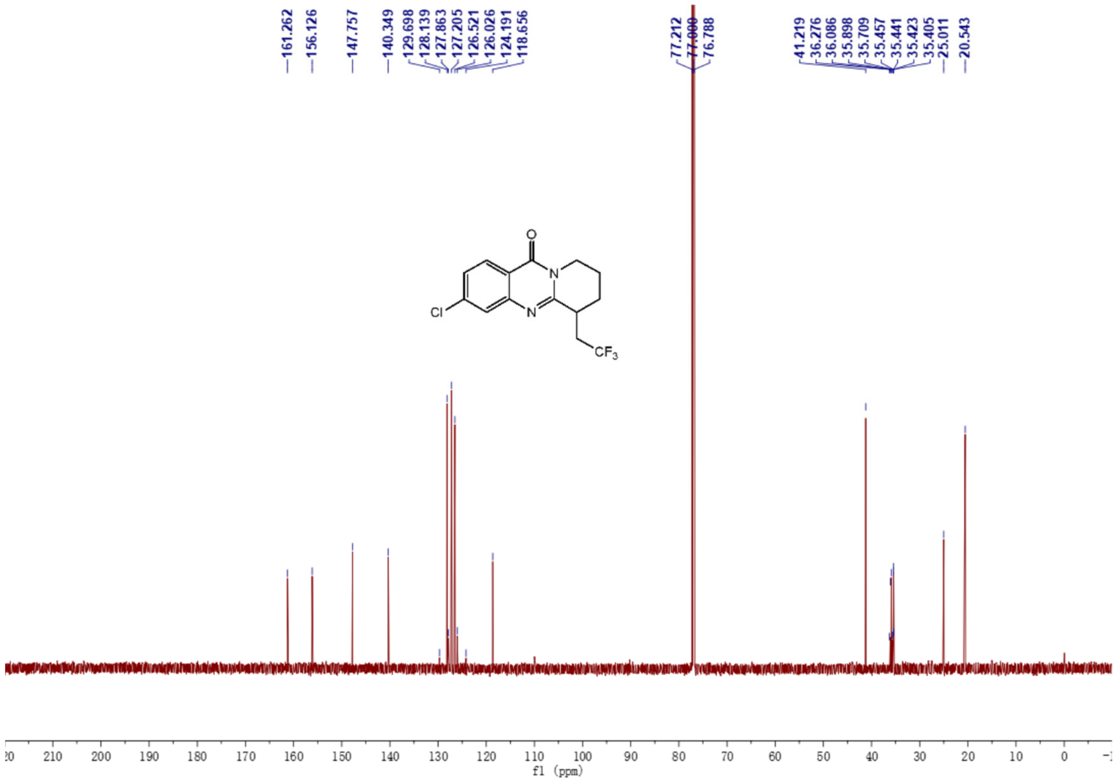

3-chloro-6-(2,2,2-trifluoroethyl)-6,7,8,9-tetrahydro-11H-pyrido[2,1-*b*]quinazolin-11-one (**3k**) : ^{19}F NMR (376 MHz, $CDCl_3$)

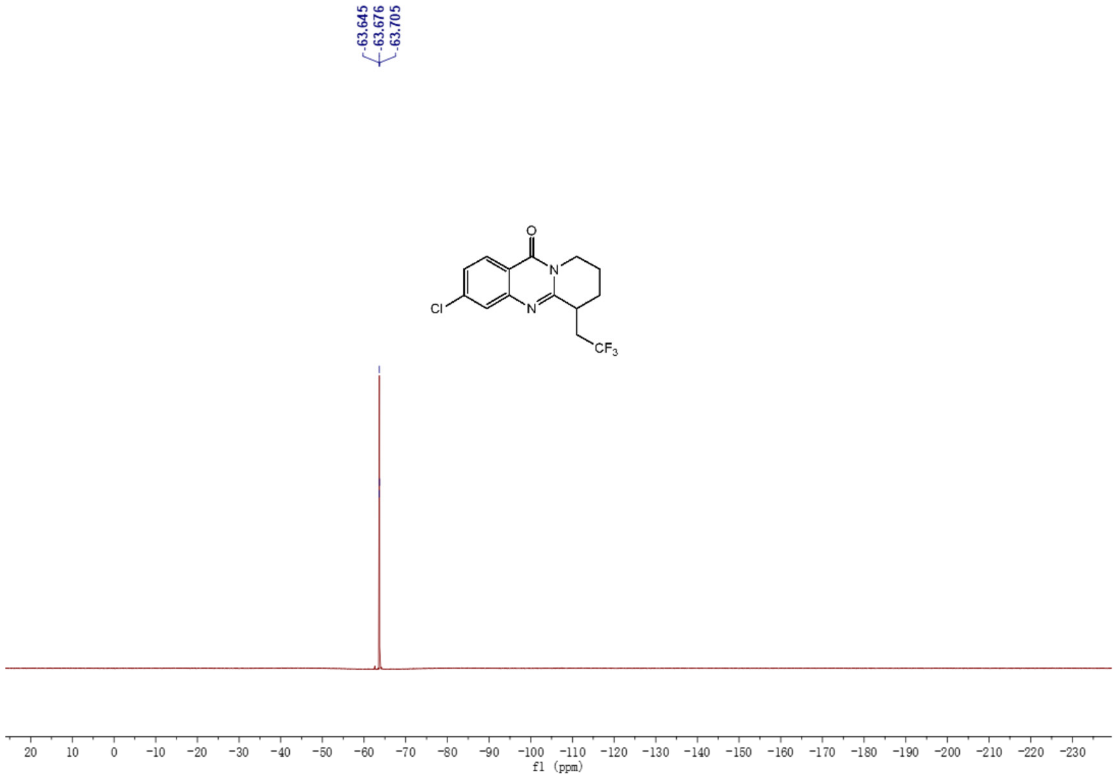

6-(2,2,2-trifluoroethyl)-3-(trifluoromethyl)-6,7,8,9-tetrahydro-11H-pyrido[2,1-*b*]quinazolin-11-one (**3l**) : ^{1}H NMR (400 MHz, $CDCl_3$)

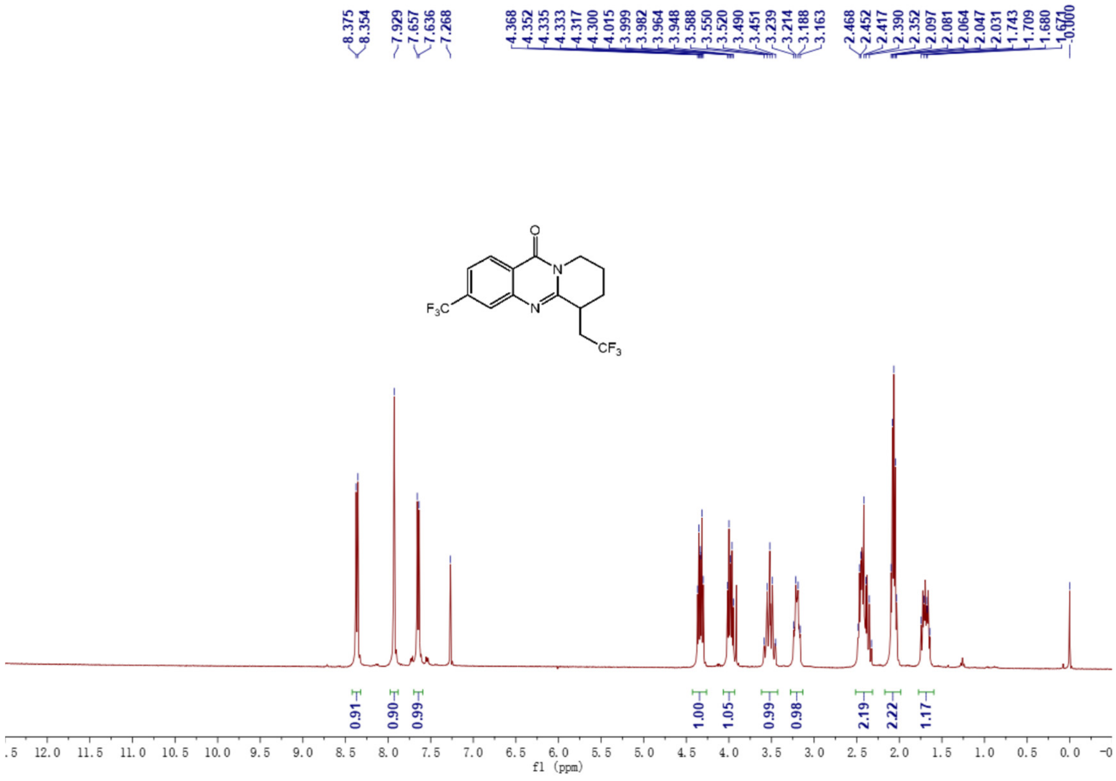

6-(2,2,2-trifluoroethyl)-3-(trifluoromethyl)-6,7,8,9-tetrahydro-11H-pyrido[2,1-*b*]quinazolin-11-one (**3l**) : ^{13}C NMR (150 MHz, $CDCl_3$)

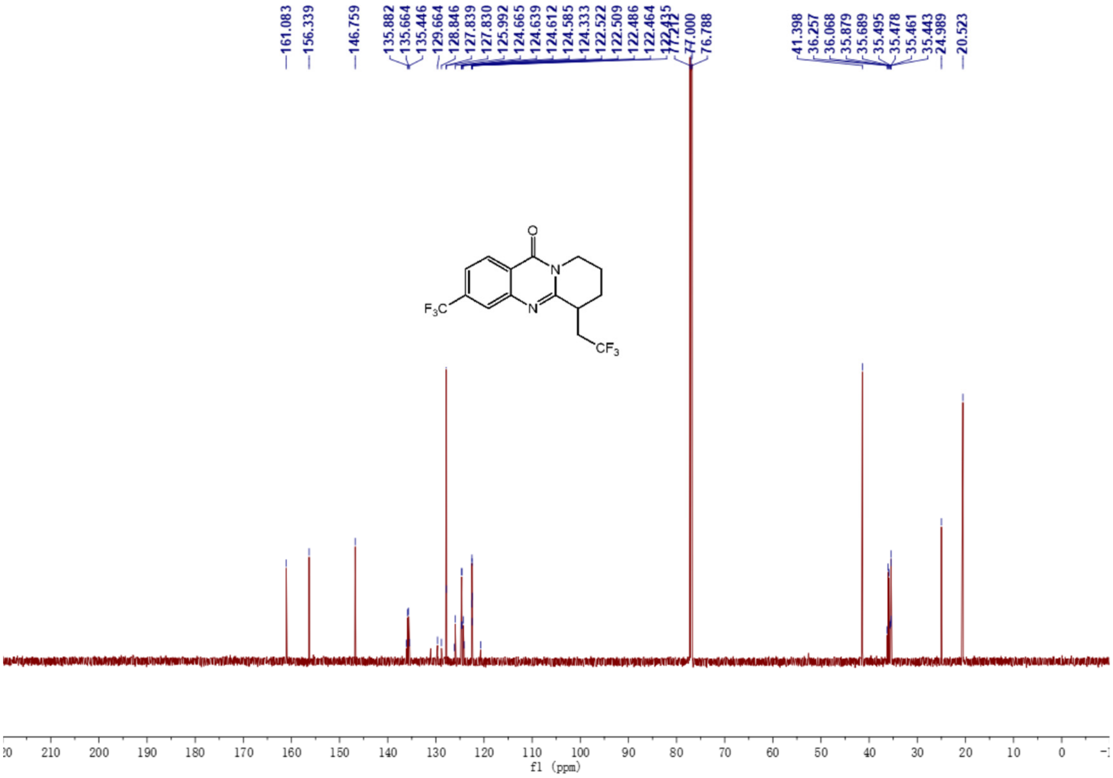

6-(2,2,2-trifluoroethyl)-3-(trifluoromethyl)-6,7,8,9-tetrahydro-11H-pyrido[2,1-*b*]quinazolin-11-one (**3l**) : ^{19}F NMR (376 MHz, $CDCl_3$)

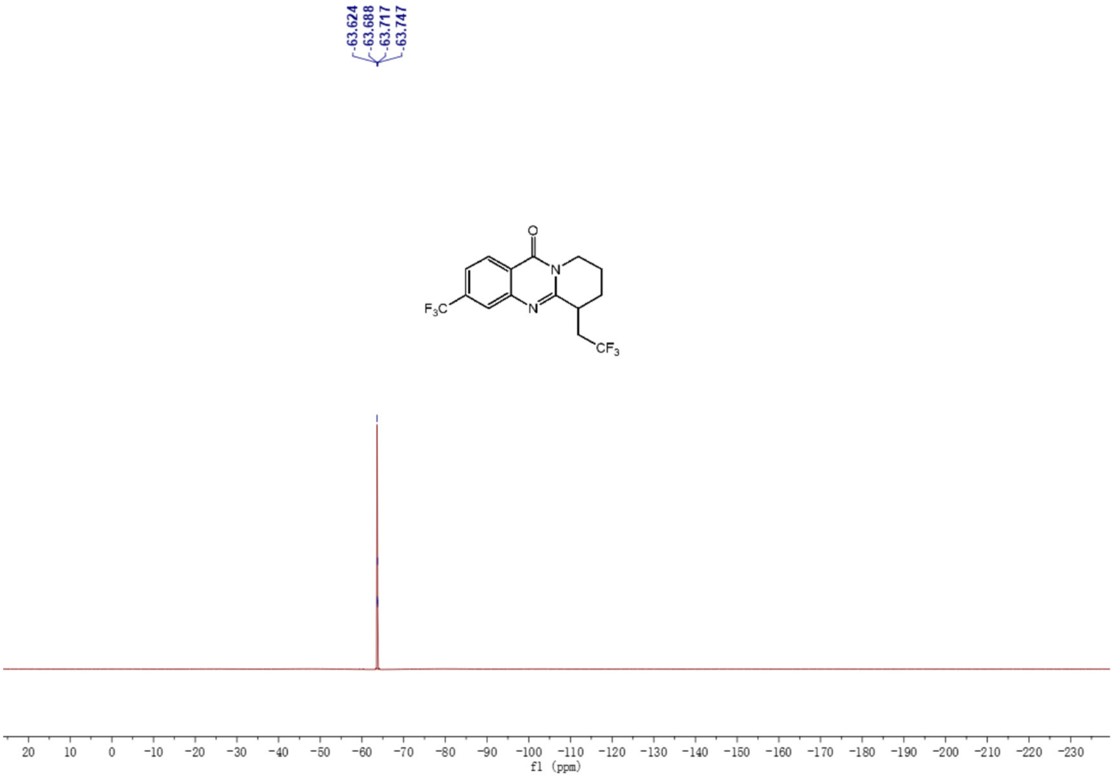

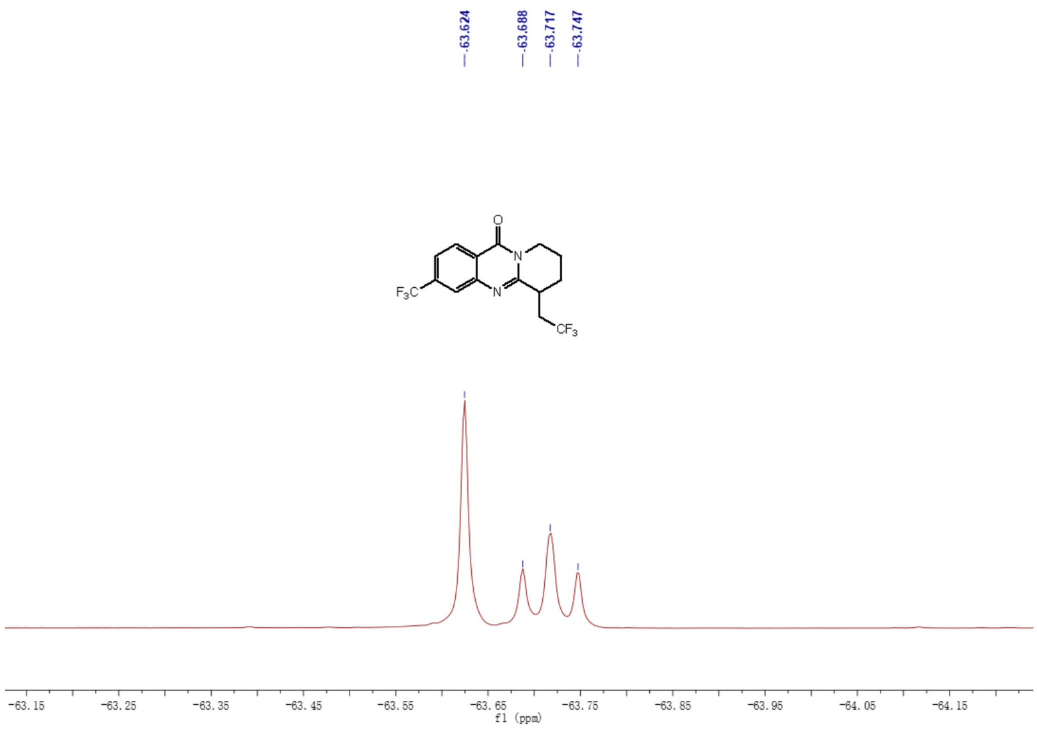

4-methyl-6-(2,2,2-trifluoroethyl)-6,7,8,9-tetrahydro-11H-pyrido[2,1-*b*]quinazolin-11-one (**3m**) : ^{1}H NMR (400 MHz, $CDCl_3$)

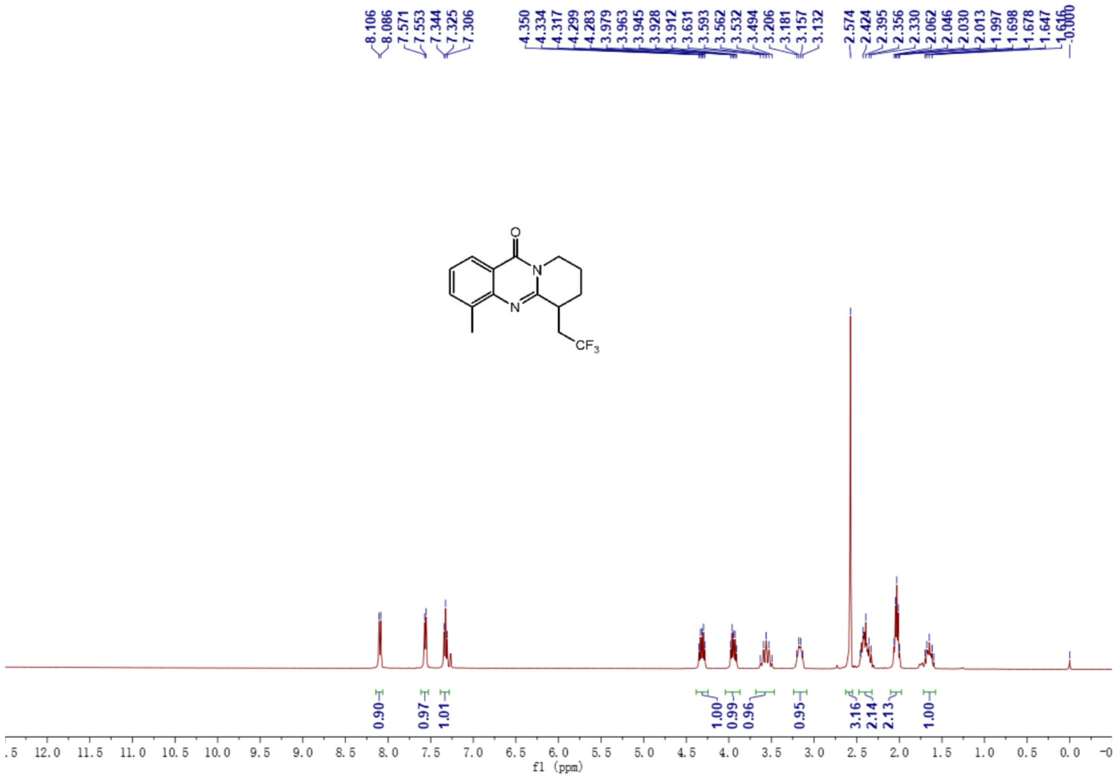

4-methyl-6-(2,2,2-trifluoroethyl)-6,7,8,9-tetrahydro-11H-pyrido[2,1-*b*]quinazolin-11-one (**3m**) **:** ^{13}C NMR (150 MHz, $CDCl_3$)

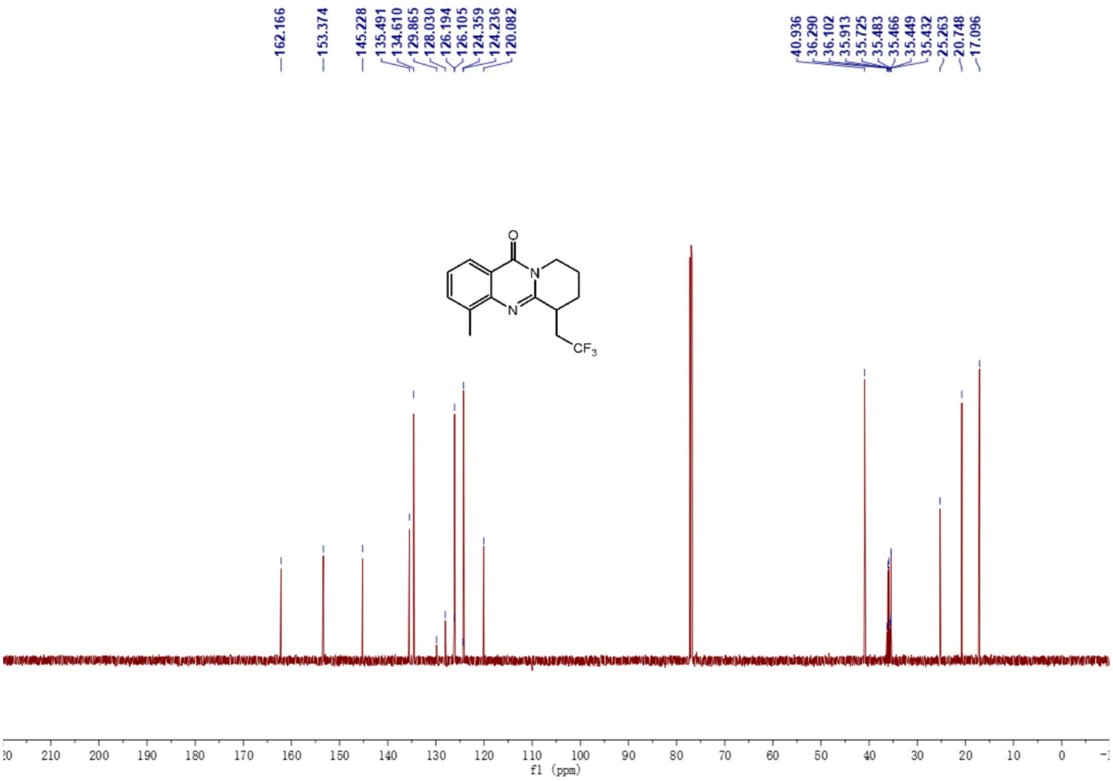

4-methyl-6-(2,2,2-trifluoroethyl)-6,7,8,9-tetrahydro-11H-pyrido[2,1-*b*]quinazolin-11-one (**3m**) **:** ^{19}F NMR (376 MHz, $CDCl_3$)

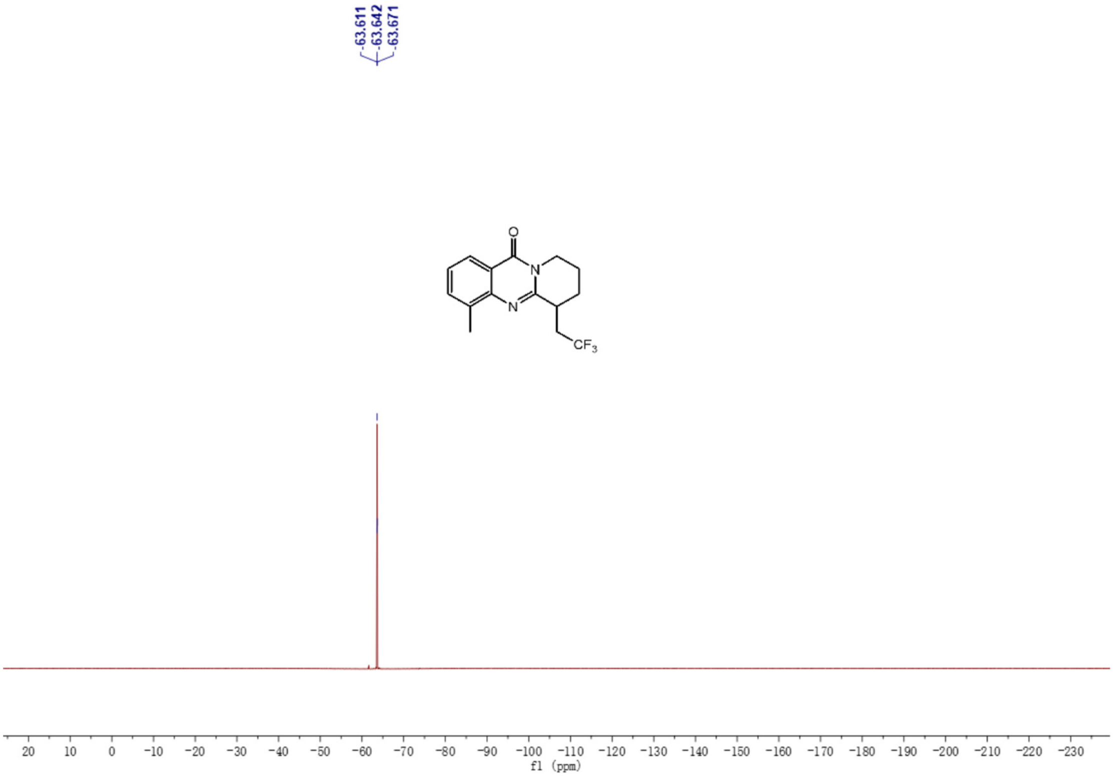

4-fluoro-6-(2,2,2-trifluoroethyl)-6,7,8,9-tetrahydro-11H-pyrido[2,1-*b*]quinazolin-11-one (**3n**) : ^{1}H NMR (400 MHz, $CDCl_3$)

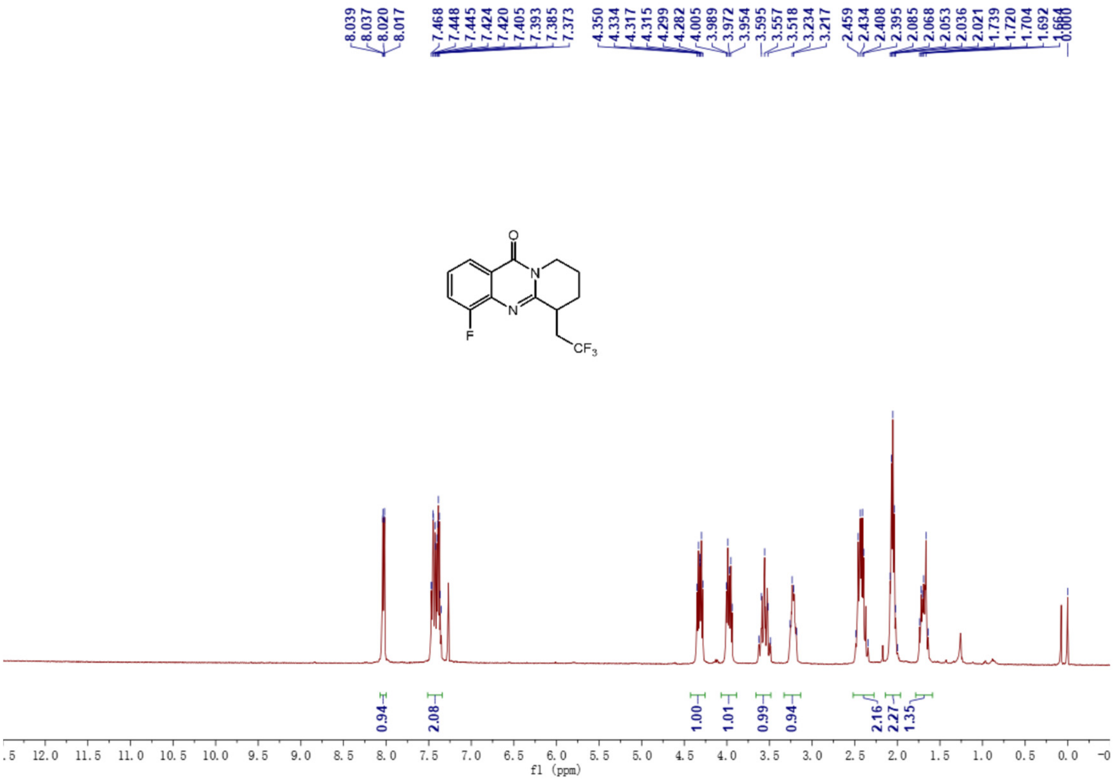

4-fluoro-6-(2,2,2-trifluoroethyl)-6,7,8,9-tetrahydro-11H-pyrido[2,1-*b*]quinazolin-11-one (**3n**) : ^{13}C NMR (150 MHz, $CDCl_3$)

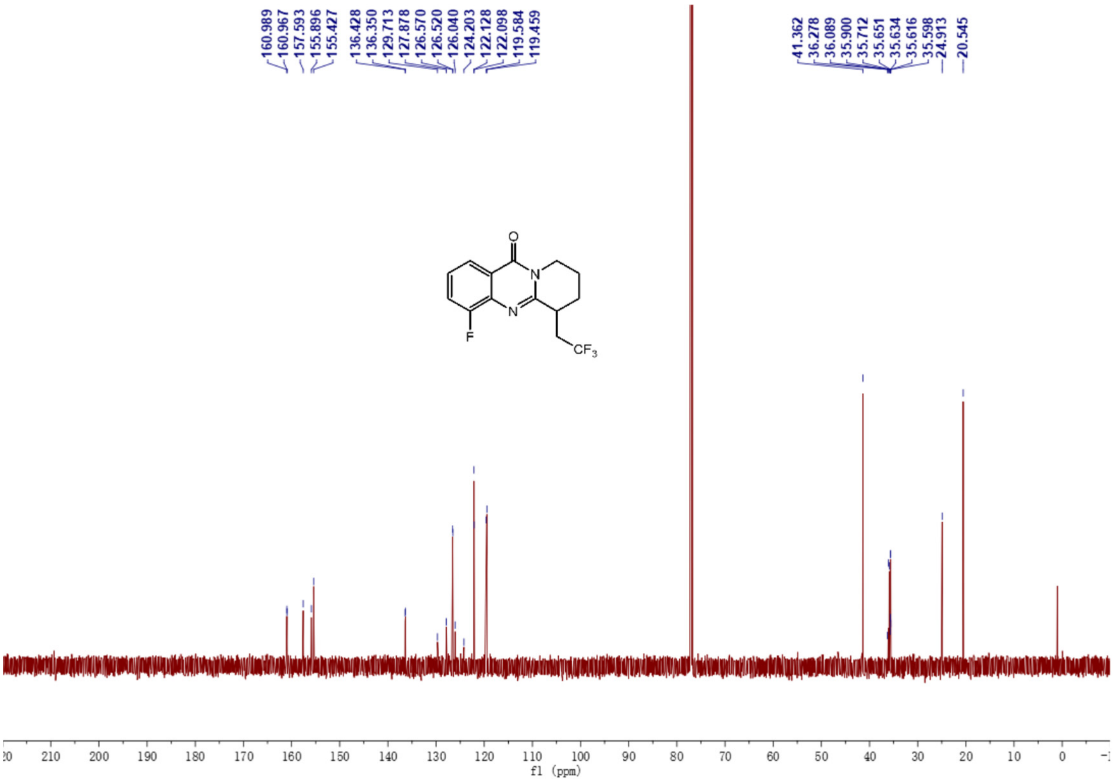

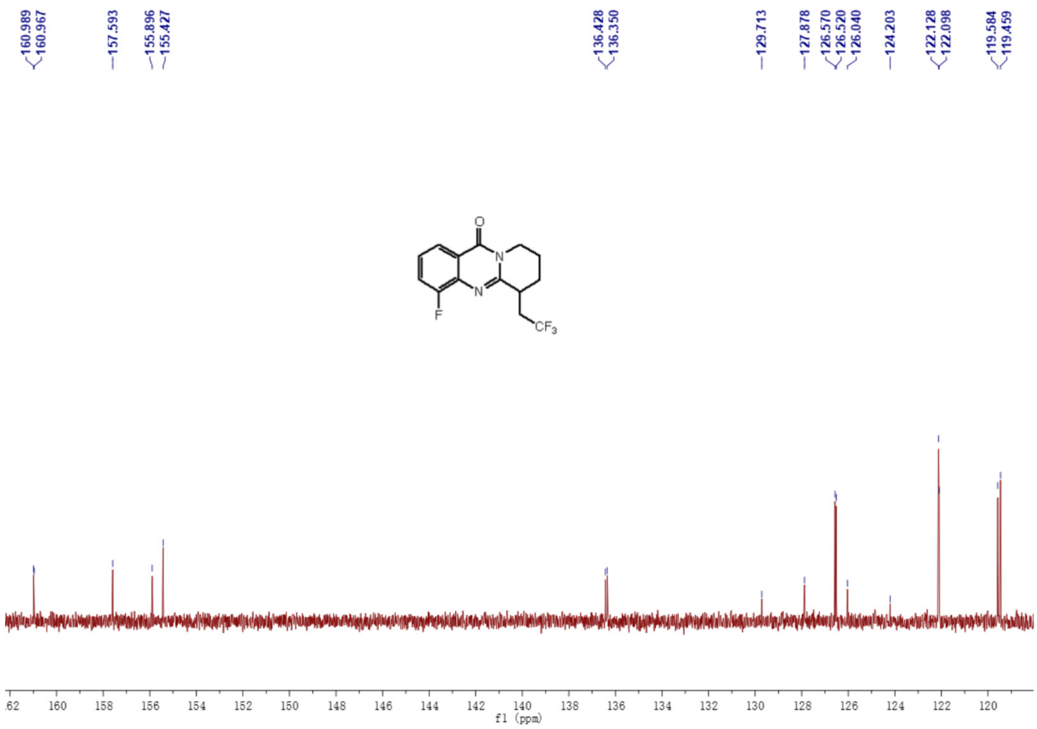

4-fluoro-6-(2,2,2-trifluoroethyl)-6,7,8,9-tetrahydro-11H-pyrido[2,1-*b*]quinazolin-11-one (**3n**) : ^{19}F NMR (376 MHz, $CDCl_3$)

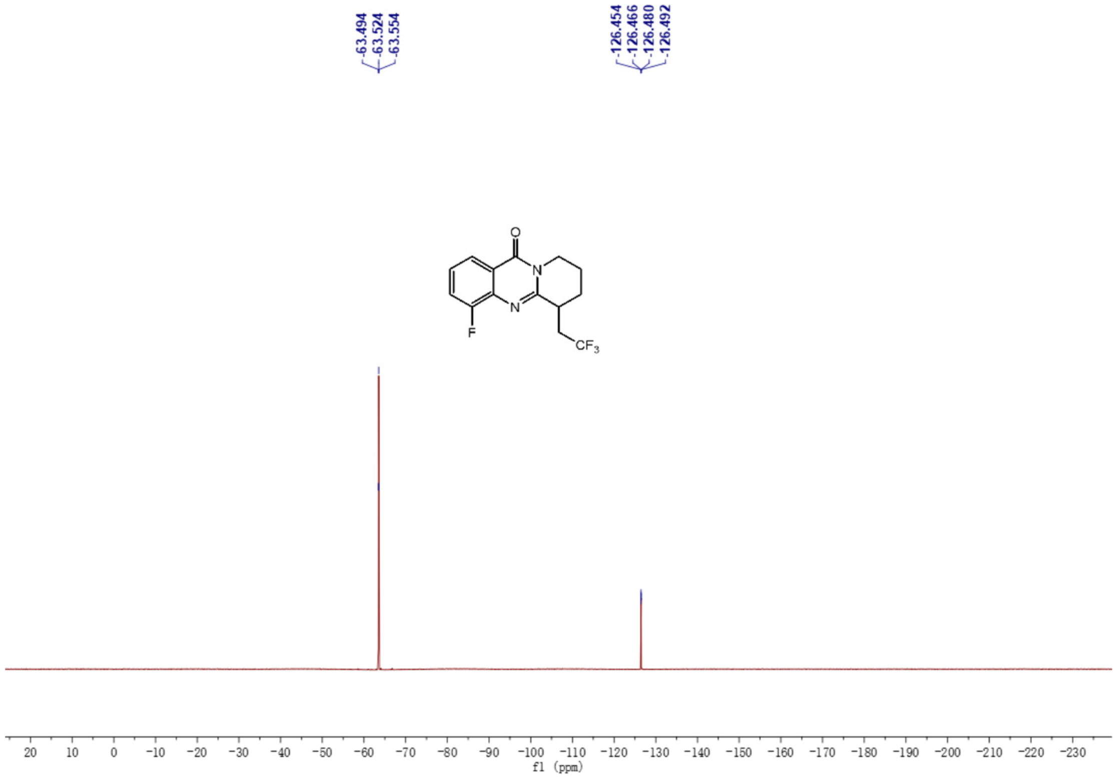

4-chloro-6-(2,2,2-trifluoroethyl)-6,7,8,9-tetrahydro-11H-pyrido[2,1-*b*]quinazolin-11-one (**3o**) : ^{1}H NMR (400 MHz, $CDCl_3$)

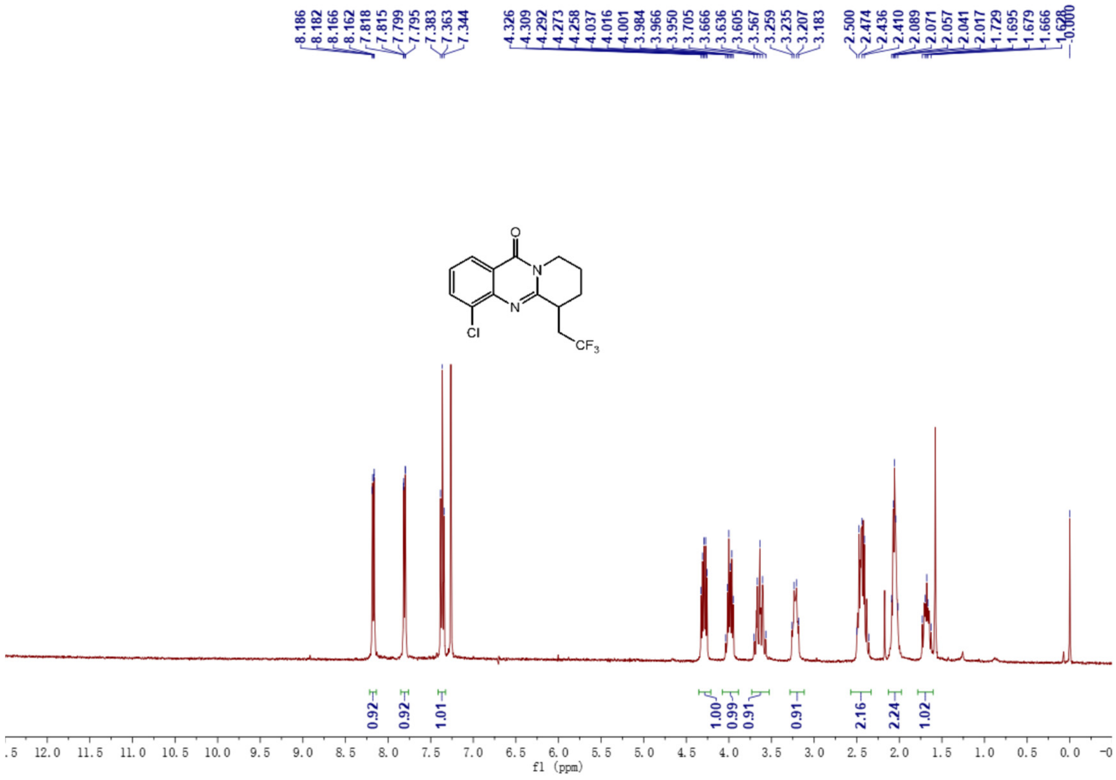

4-chloro-6-(2,2,2-trifluoroethyl)-6,7,8,9-tetrahydro-11H-pyrido[2,1-*b*]quinazolin-11-one (**3o**) : ^{13}C NMR (150 MHz, $CDCl_3$)

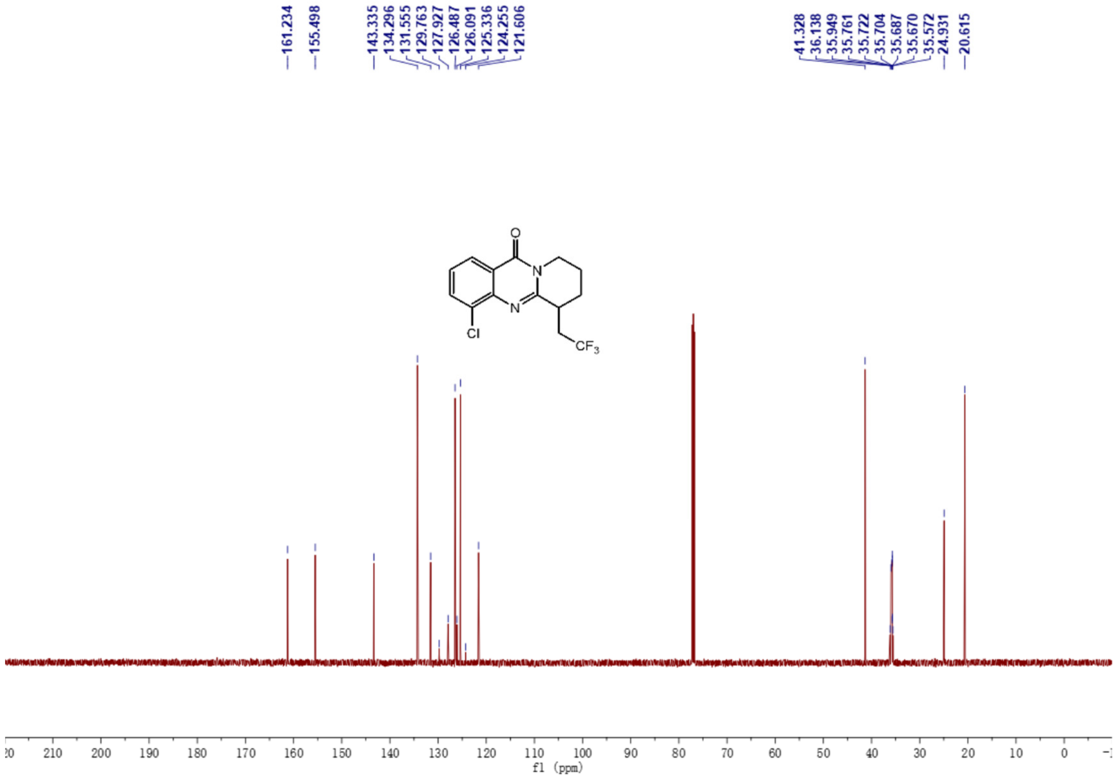

4-chloro-6-(2,2,2-trifluoroethyl)-6,7,8,9-tetrahydro-11H-pyrido[2,1-*b*]quinazolin-11-one (**3o**) : ^{19}F NMR (376 MHz, $CDCl_3$)

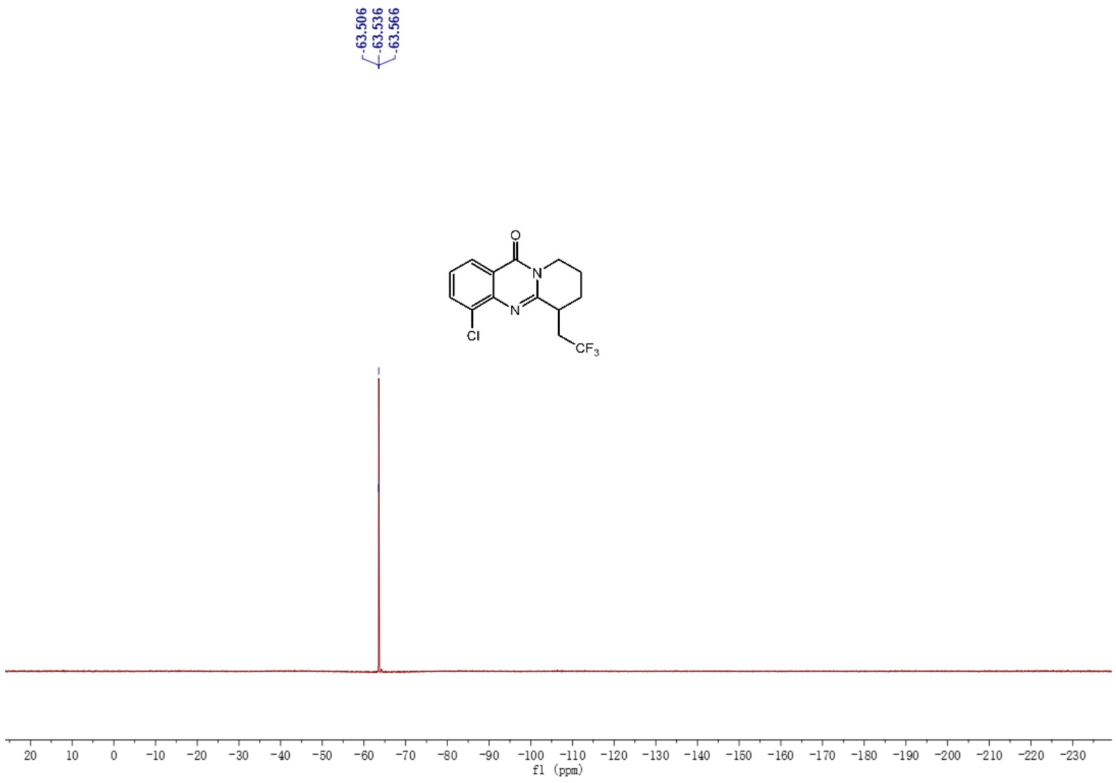

2,3-dimethoxy-6-(2,2,2-trifluoroethyl)-6,7,8,9-tetrahydro-11H-pyrido[2,1-*b*]quinazolin-11-one (**3p**) : ^{1}H NMR (400 MHz, CDCl$_3$)

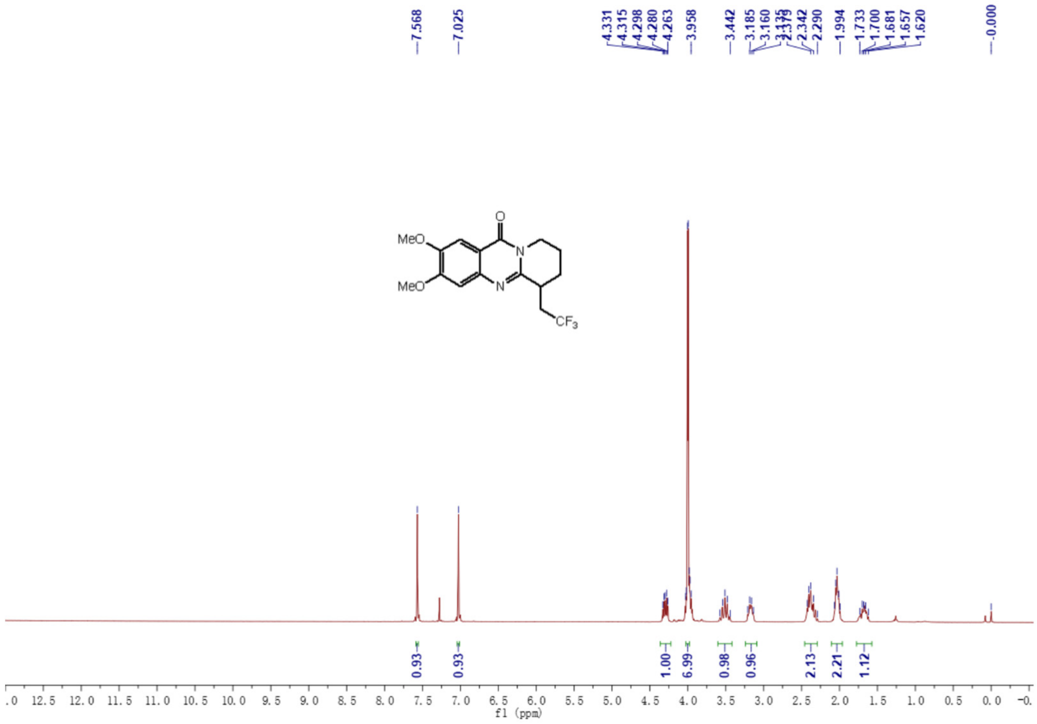

2,3-dimethoxy-6-(2,2,2-trifluoroethyl)-6,7,8,9-tetrahydro-11H-pyrido[2,1-*b*]quinazolin-11-one (**3p**) **:** ^{13}C NMR (150 MHz, CDCl$_3$)

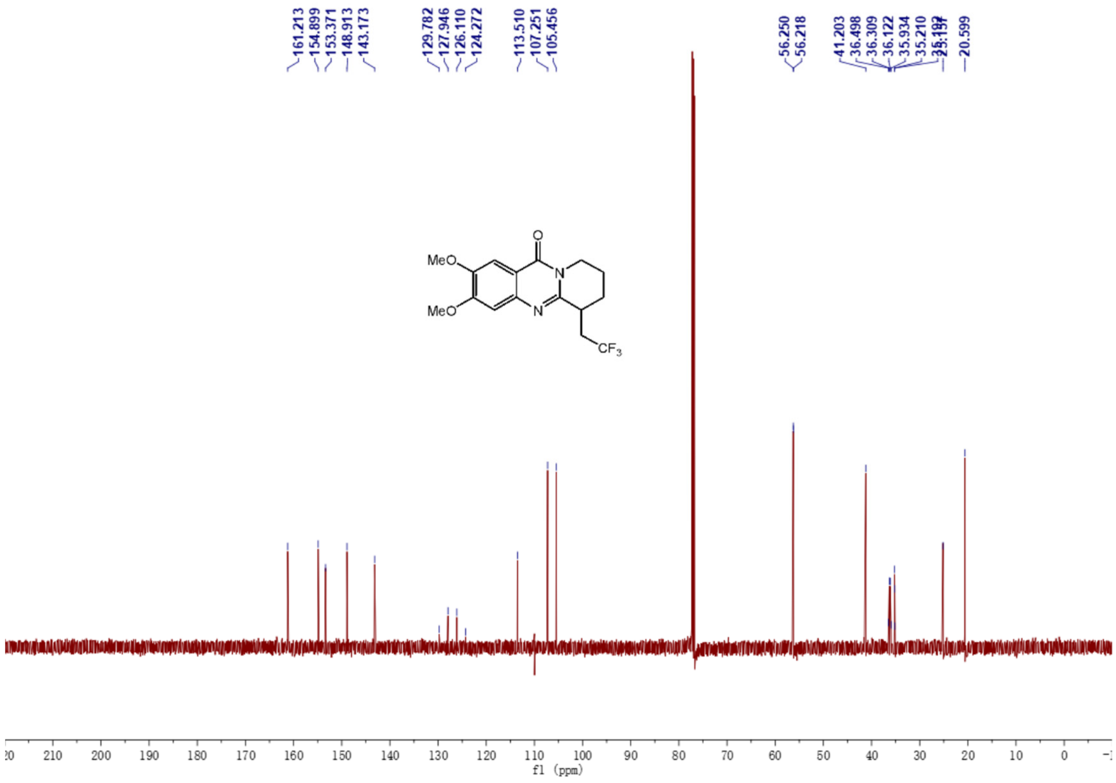

2,3-dimethoxy-6-(2,2,2-trifluoroethyl)-6,7,8,9-tetrahydro-11H-pyrido[2,1-*b*]quinazolin-11-one (**3p**) : ^{19}F NMR (376 MHz, $CDCl_3$)

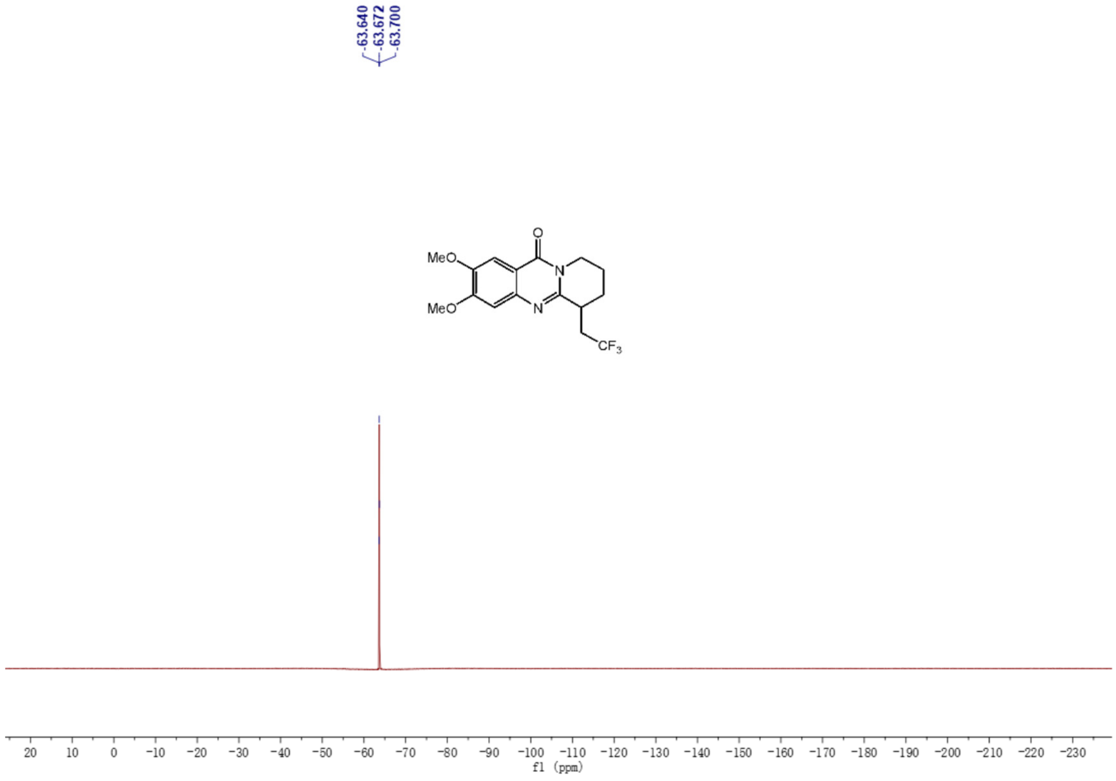

2,4-dimethyl-6-(2,2,2-trifluoroethyl)-6,7,8,9-tetrahydro-11H-pyrido[2,1-*b*]quinazolin-11-one (**3q**) : ^{1}H NMR (400 MHz, $CDCl_3$)

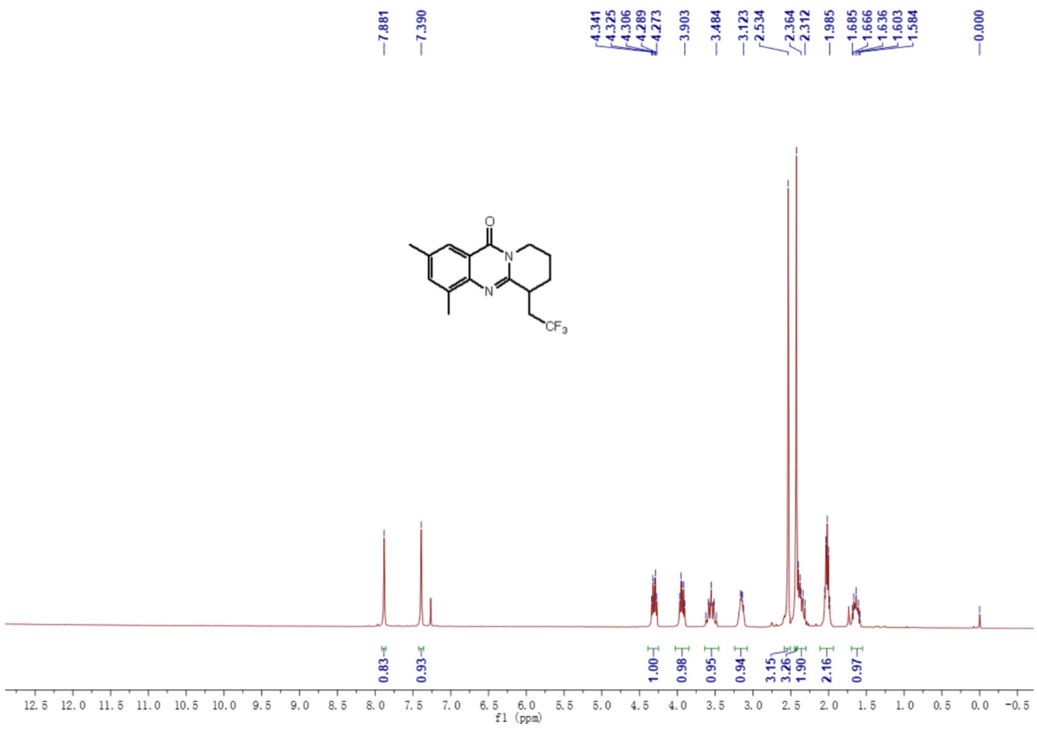

2,4-dimethyl-6-(2,2,2-trifluoroethyl)-6,7,8,9-tetrahydro-11H-pyrido[2,1-*b*]quinazolin-11-one (**3q**) **:** ^{13}C NMR (150 MHz, CDCl$_3$)

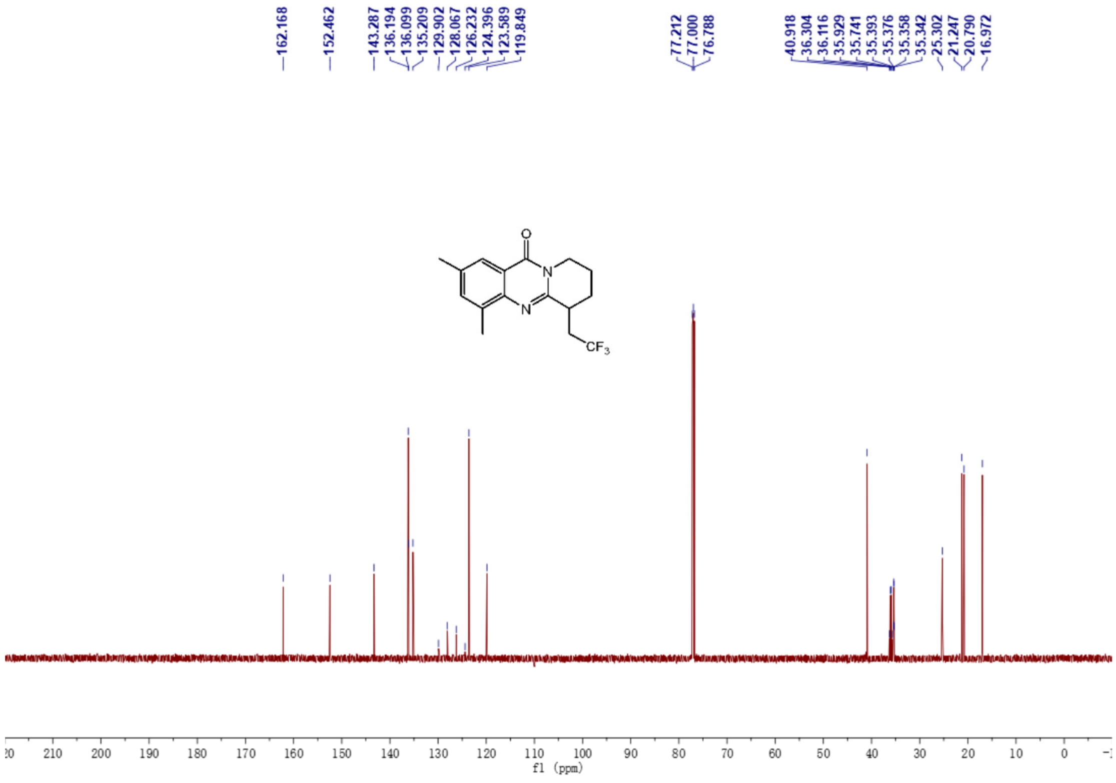

2,4-dimethyl-6-(2,2,2-trifluoroethyl)-6,7,8,9-tetrahydro-11H-pyrido[2,1-*b*]quinazolin-11-one (**3q**) **:** ^{19}F NMR (376 MHz, CDCl$_3$)

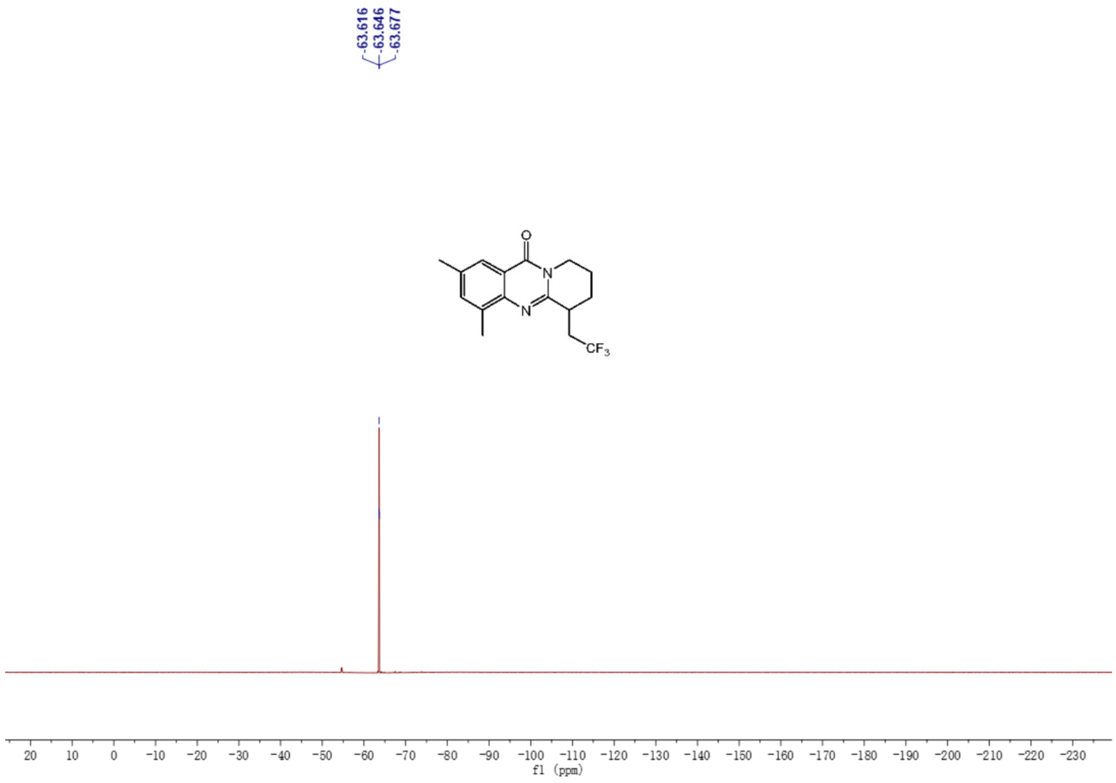

2,3-difluoro-6-(2,2,2-trifluoroethyl)-6,7,8,9-tetrahydro-11H-pyrido[2,1-*b*]quinazolin-11-one (**3r**) : ^{1}H NMR (400 MHz, $CDCl_3$)

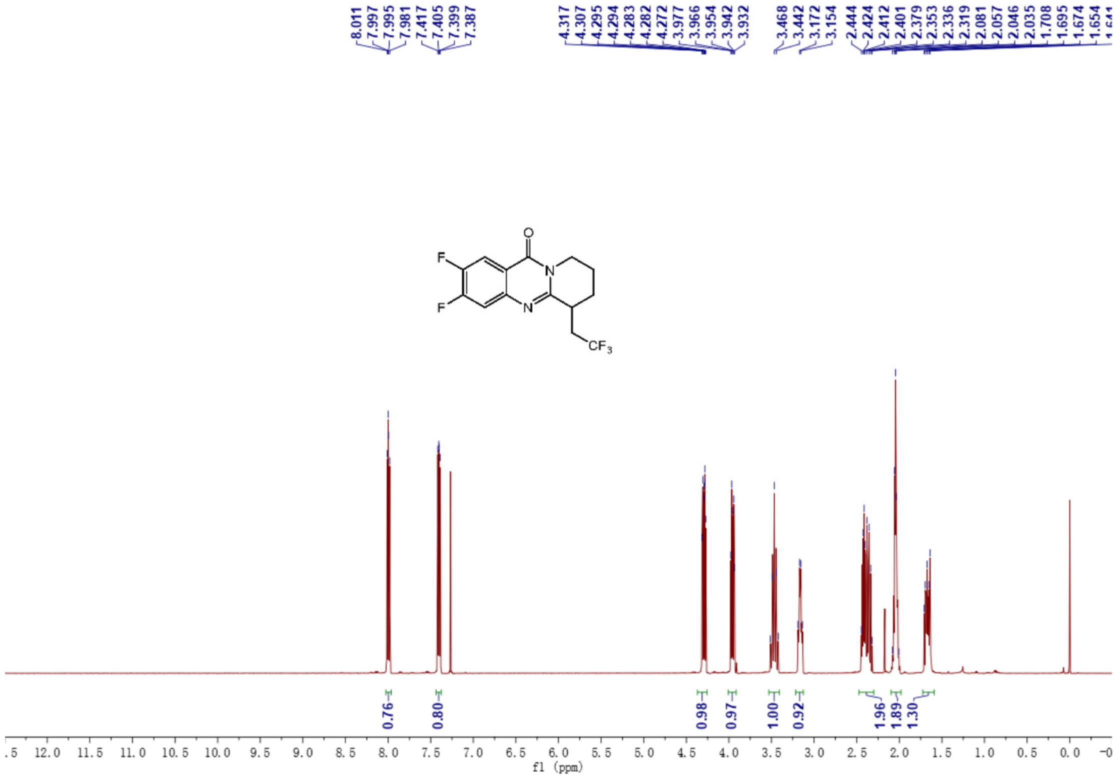

2,3-difluoro-6-(2,2,2-trifluoroethyl)-6,7,8,9-tetrahydro-11H-pyrido[2,1-*b*]quinazolin-11-one (**3r**) **:** ^{13}C NMR (150 MHz, $CDCl_3$)

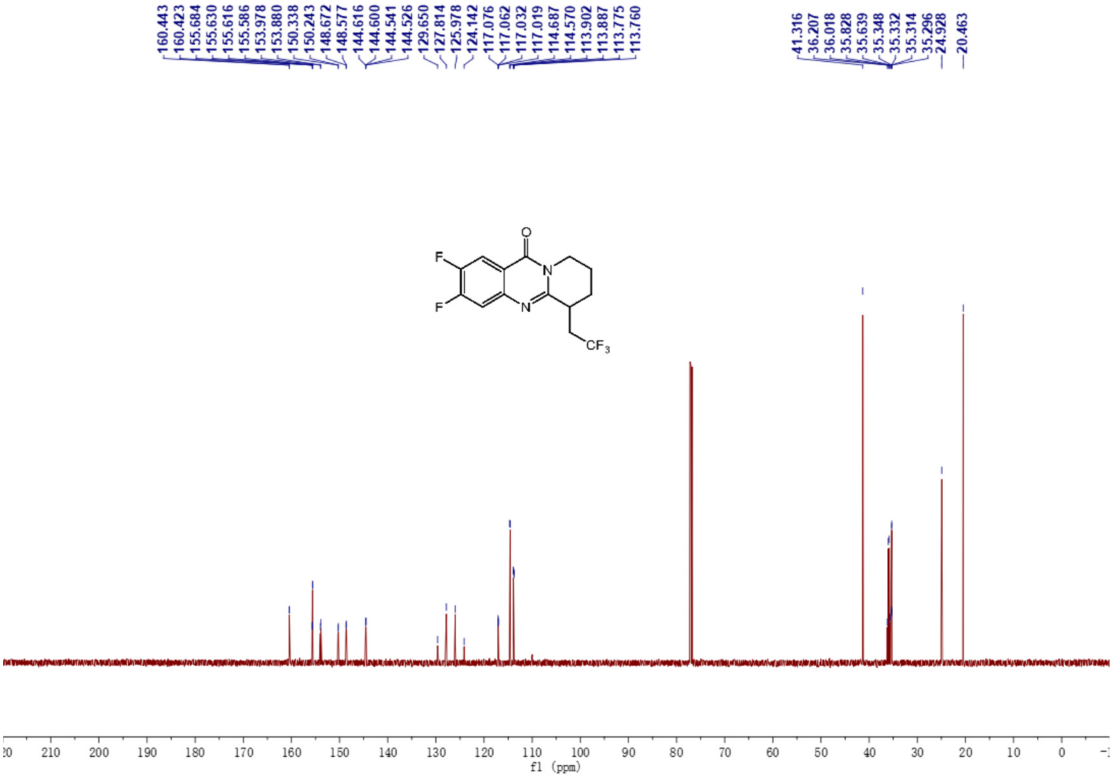
160.443
160.423
155.684
155.630
155.616
155.586
153.978
153.880
150.338
150.243
148.672
148.577
144.616
144.600
144.541
144.526
129.650
127.814
125.978
124.142
117.076
117.062
117.032
117.019
114.687
114.570
113.902
113.887
113.775
113.760
41.316
36.207
36.018
35.828
35.639
35.348
35.332
35.314
35.296
24.928
20.463
F
F
O
N
N
CF3
f1 (ppm)

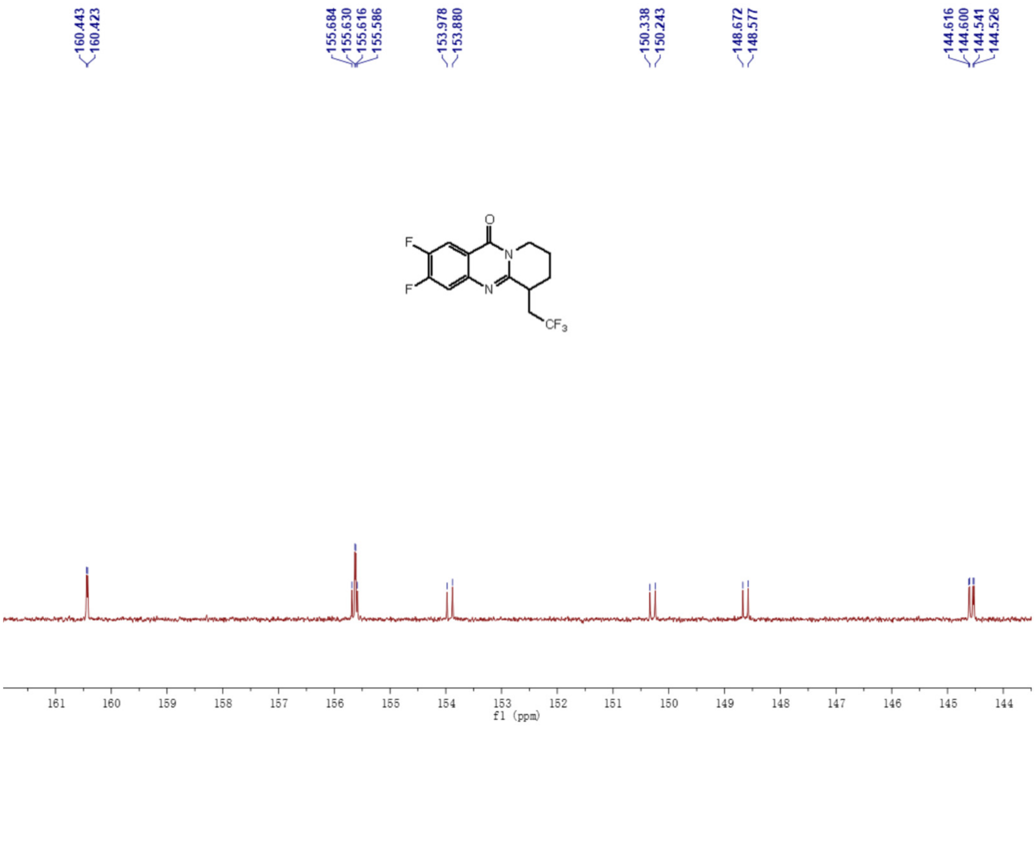
160.443
160.423
155.684
155.630
155.616
155.586
153.978
153.880
150.338
150.243
148.672
148.577
144.616
144.600
144.541
144.526
F
F
O
N
N
CF3
f1 (ppm)

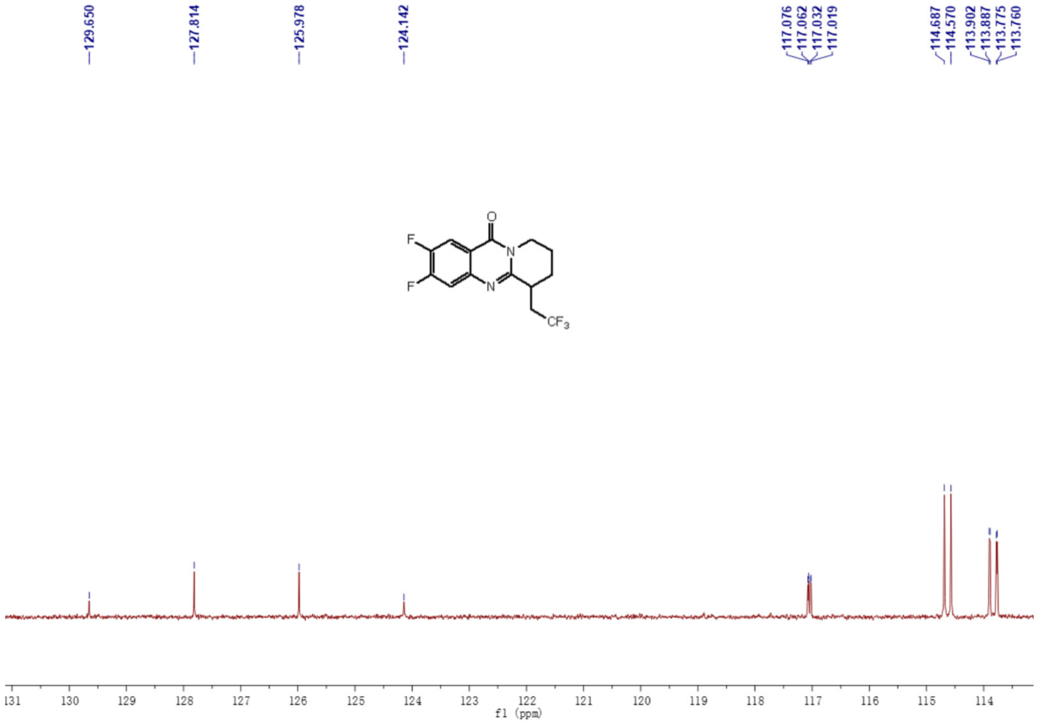

2,3-difluoro-6-(2,2,2-trifluoroethyl)-6,7,8,9-tetrahydro-11H-pyrido[2,1-*b*]quinazolin-11-one (**3r**) : ^{19}F NMR (376 MHz, $CDCl_3$)

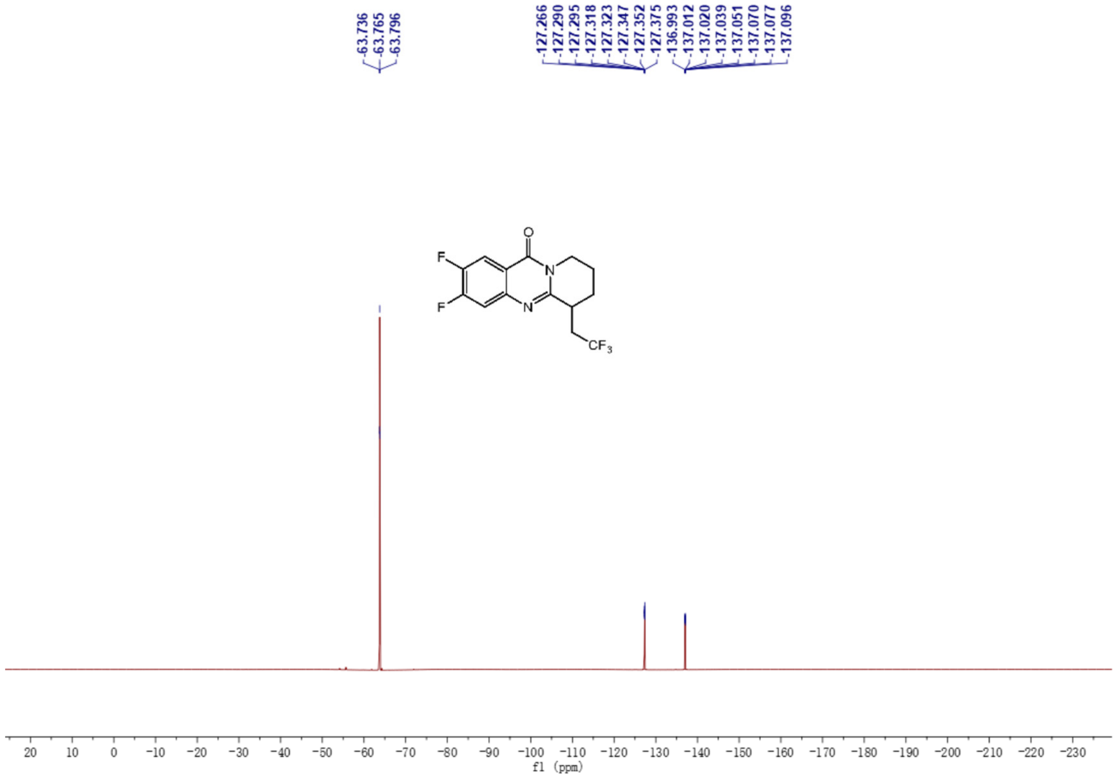

3-(2,2,2-trifluoroethyl)-2,3-dihydropyrrolo[2,1-*b*]quinazolin-9(1H)-one (**3s**) : ^{1}H NMR (400 MHz, $CDCl_3$)

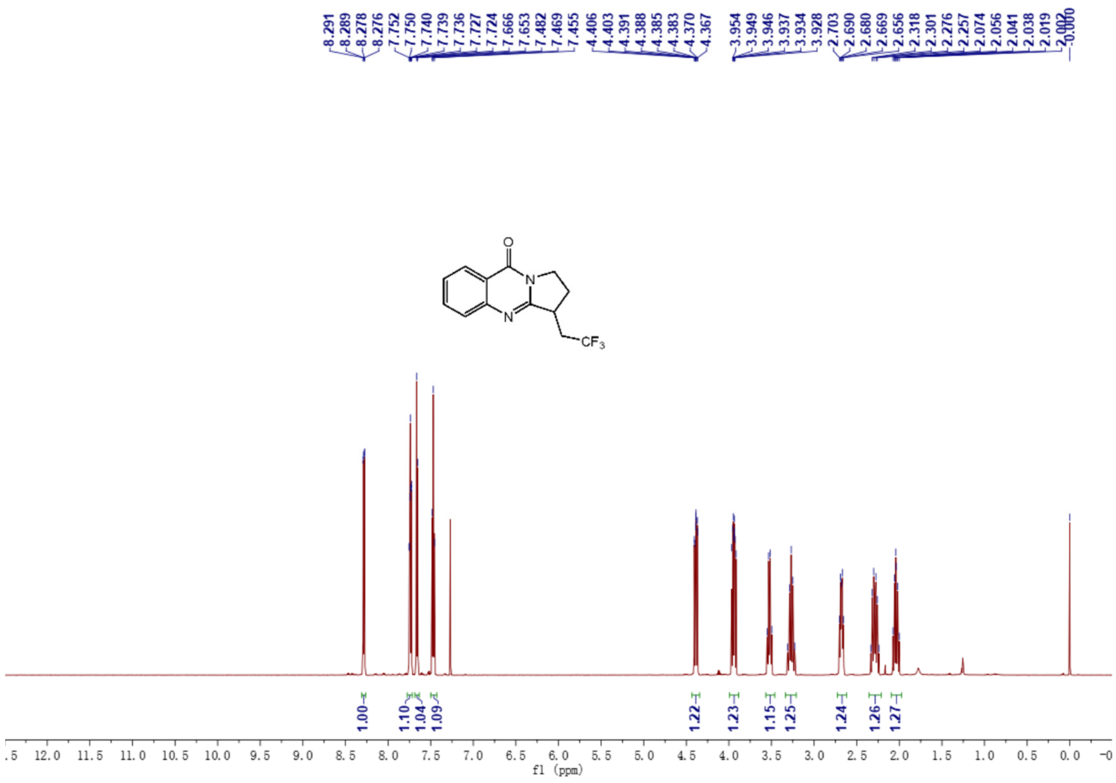

3-(2,2,2-trifluoroethyl)-2,3-dihydropyrrolo[2,1-*b*]quinazolin-9(1H)-one (**3s**) **:** ^{13}C NMR (150 MHz, $CDCl_3$)

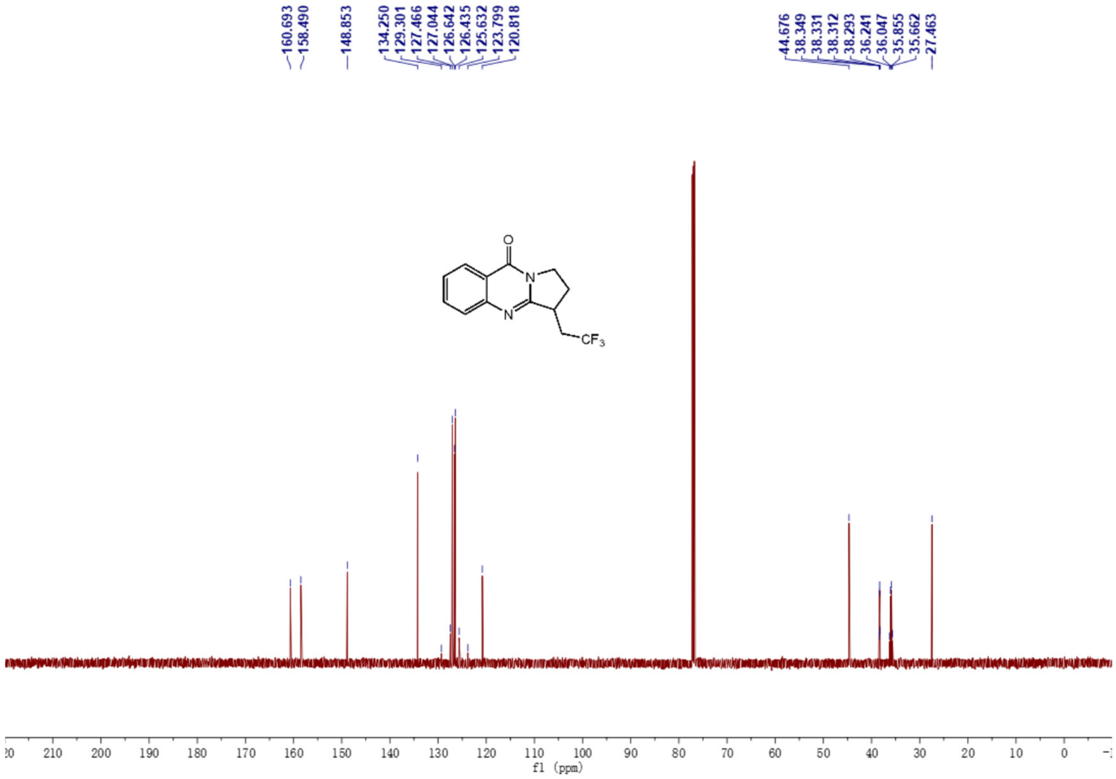

3-(2,2,2-trifluoroethyl)-2,3-dihydropyrrolo[2,1-*b*]quinazolin-9(1H)-one (**3s**) **:** ^{19}F NMR (376 MHz, $CDCl_3$)

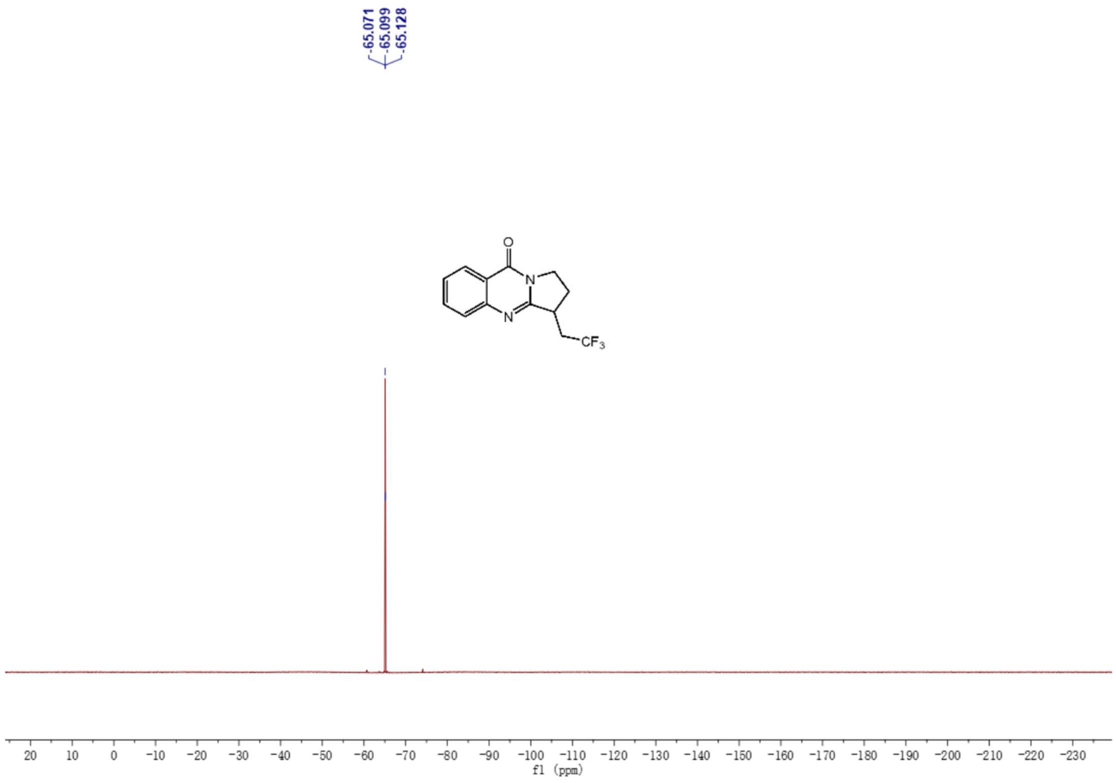

6-methyl-3-(2,2,2-trifluoroethyl)-2,3-dihydropyrrolo[2,1-*b*]quinazolin-9(1H)-one (**3t**) : ^{1}H NMR (600 MHz, CDCl$_3$)

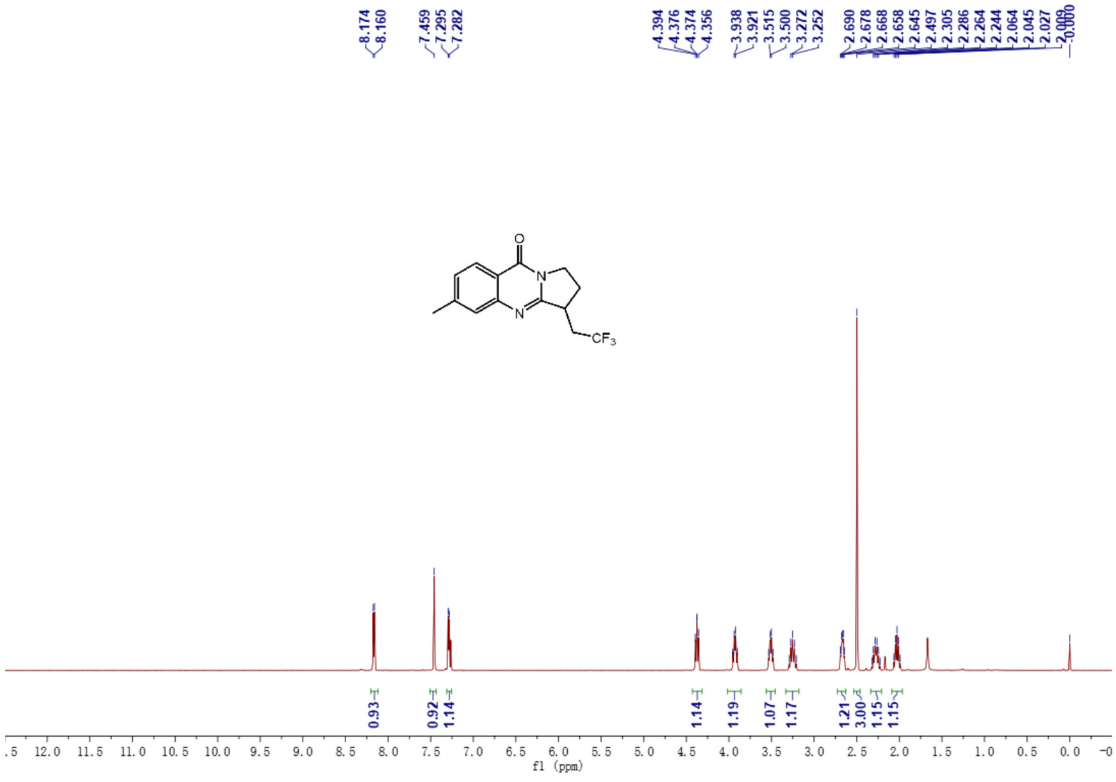

6-methyl-3-(2,2,2-trifluoroethyl)-2,3-dihydropyrrolo[2,1-*b*]quinazolin-9(1H)-one (**3t**) **:** ^{13}C NMR (150 MHz, CDCl$_3$)

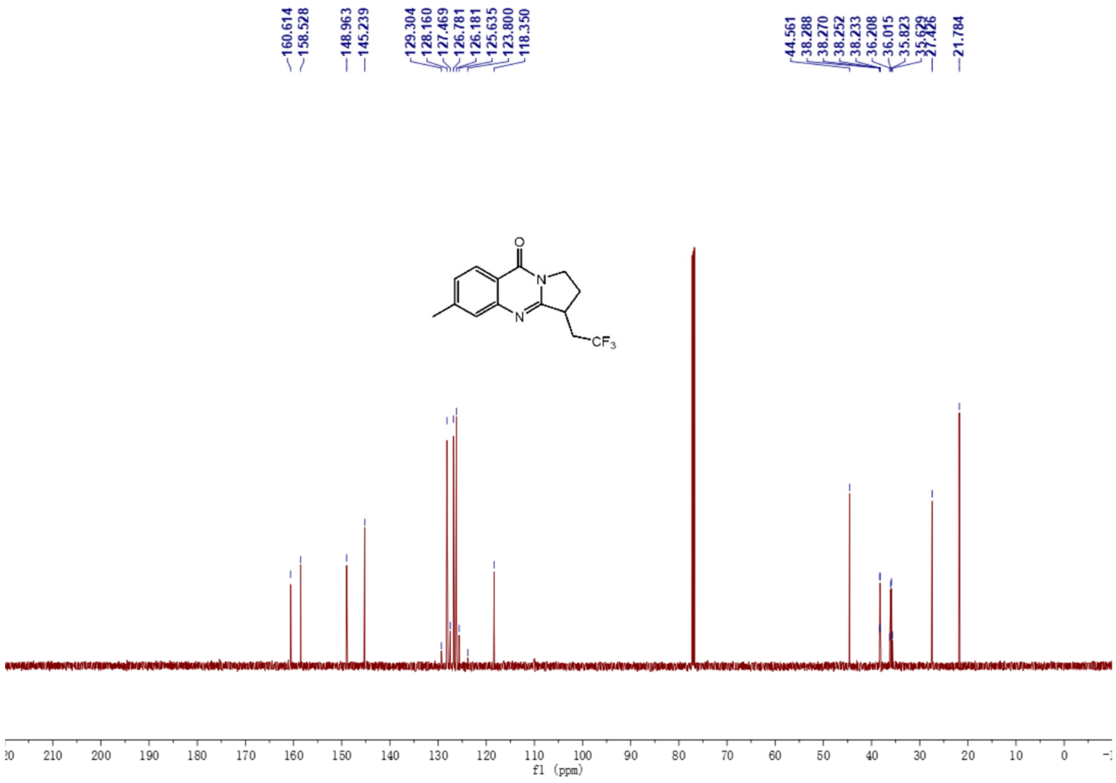

6-methyl-3-(2,2,2-trifluoroethyl)-2,3-dihydropyrrolo[2,1-*b*]quinazolin-9(1H)-one (**3t**) **:** ^{19}F NMR (376 MHz, $CDCl_3$)

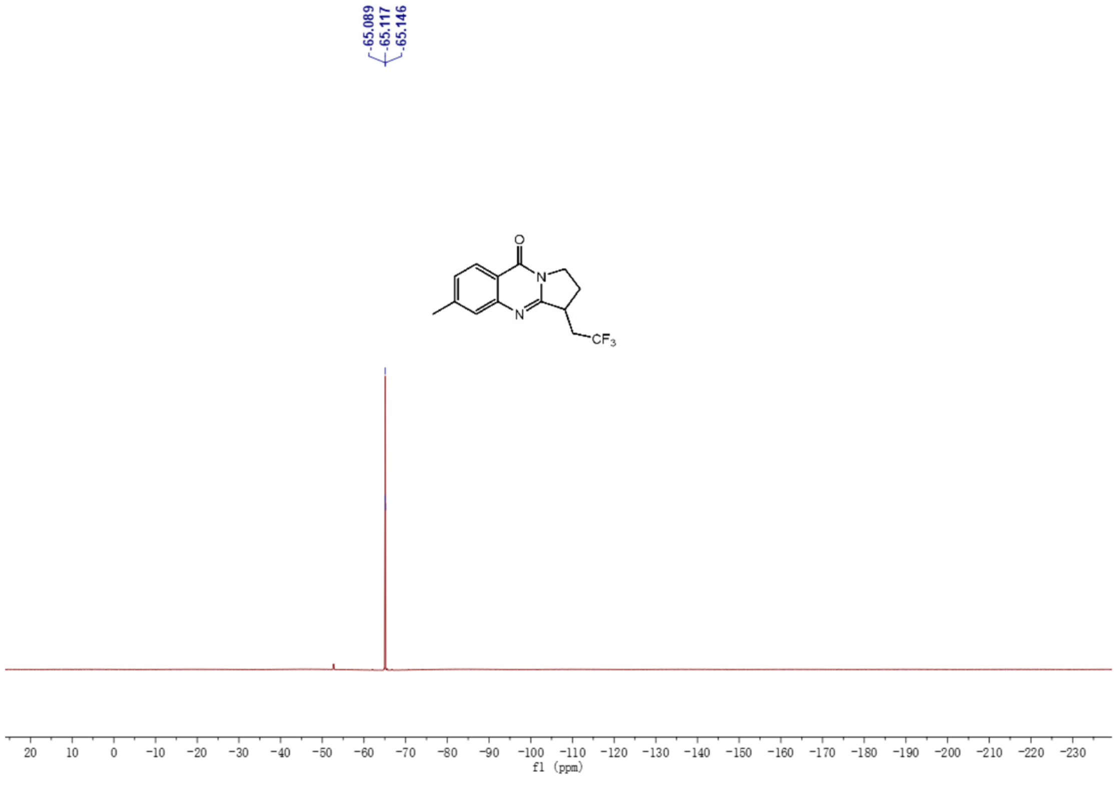

3-methyl-3-(2,2,2-trifluoroethyl)-2,3-dihydropyrrolo[2,1-*b*]quinazolin-9(1H)-one (**3u**) : ^{1}H NMR (400 MHz, $CDCl_3$)

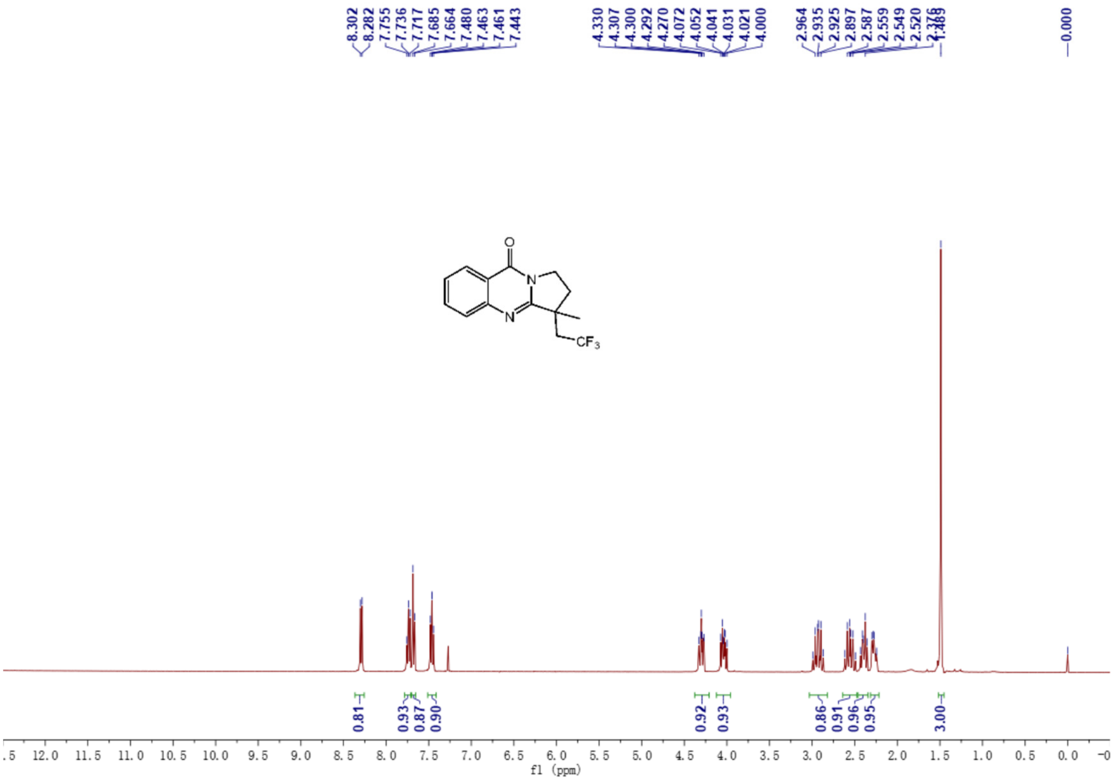

3-methyl-3-(2,2,2-trifluoroethyl)-2,3-dihydropyrrolo[2,1-*b*]quinazolin-9(1H)-one (**3u**) : ^{13}C NMR (150 MHz, CDCl3)

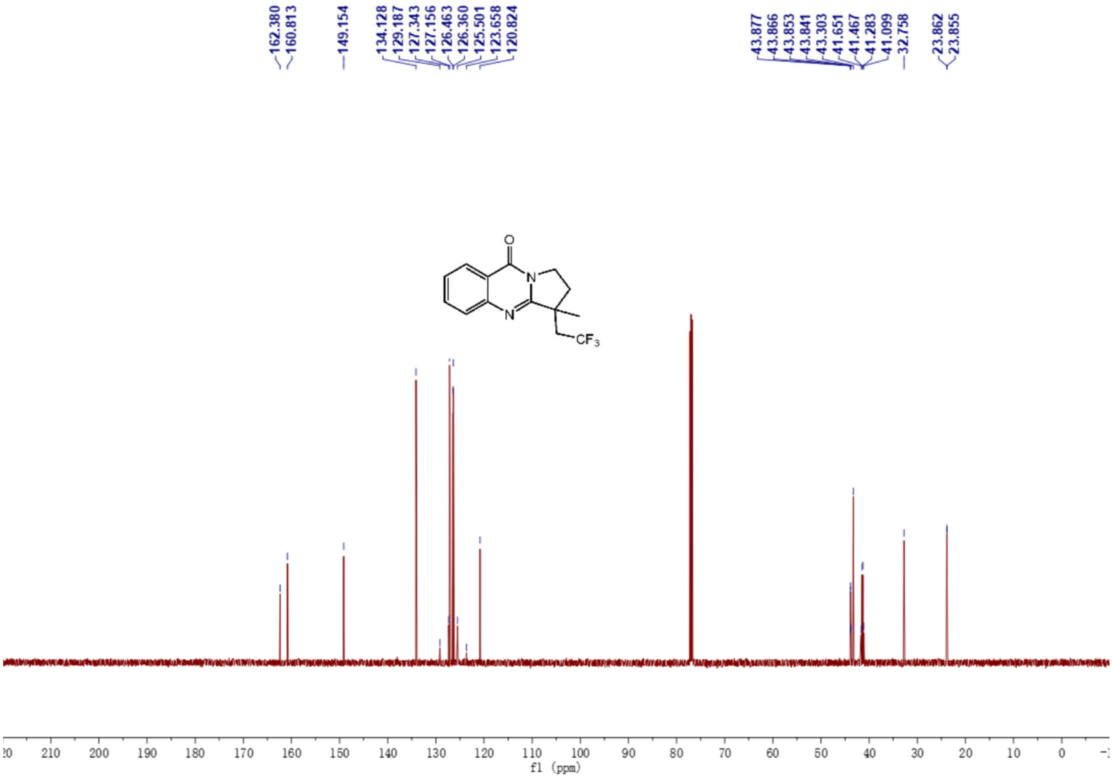

3-methyl-3-(2,2,2-trifluoroethyl)-2,3-dihydropyrrolo[2,1-*b*]quinazolin-9(1H)-one (**3u**) : ^{19}F NMR (376 MHz, CDCl3)

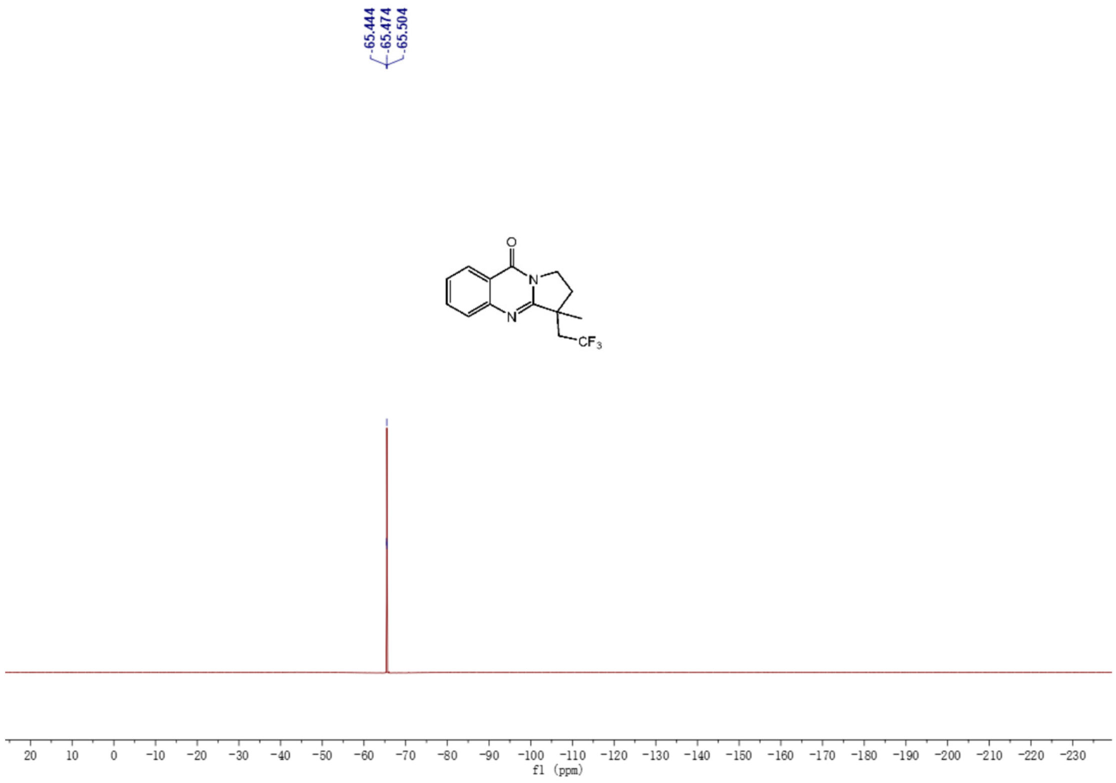

6-(2,2,2-trifluoroethyl)-7,8,9,10-tetrahydroazepino[2,1-*b*]quinazolin-12(6H)-one (**3v**) : ^{1}H NMR (400 MHz, $CDCl_3$)

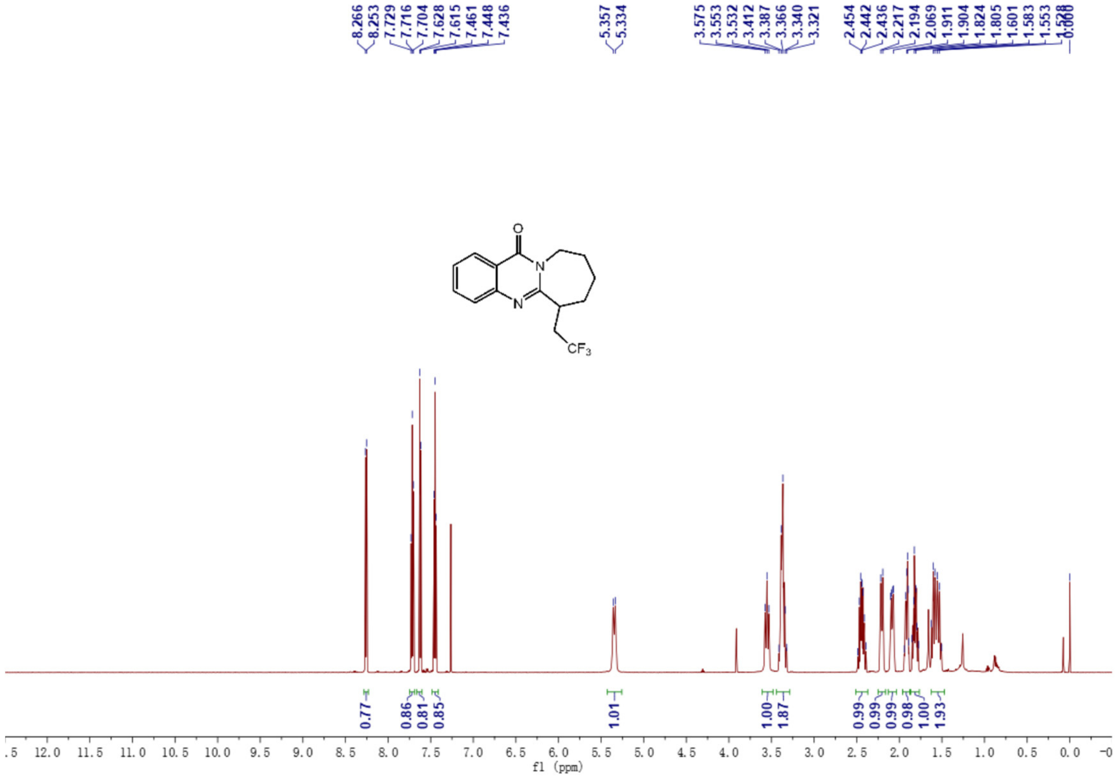

6-(2,2,2-trifluoroethyl)-7,8,9,10-tetrahydroazepino[2,1-*b*]quinazolin-12(6H)-one (**3v**) : ^{13}C NMR (150 MHz, $CDCl_3$)

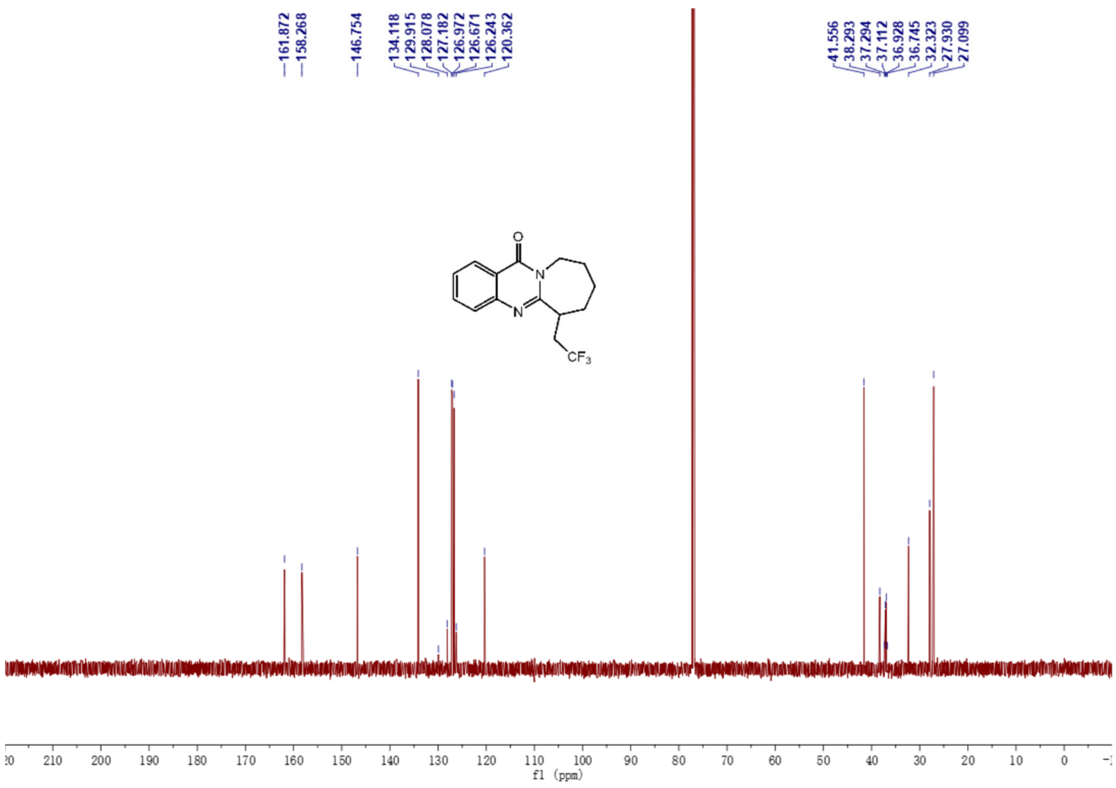

6-(2,2,2-trifluoroethyl)-7,8,9,10-tetrahydroazepino[2,1-*b*]quinazolin-12(6H)-one (**3v**) **:** ^{19}F NMR (376 MHz, $CDCl_3$)

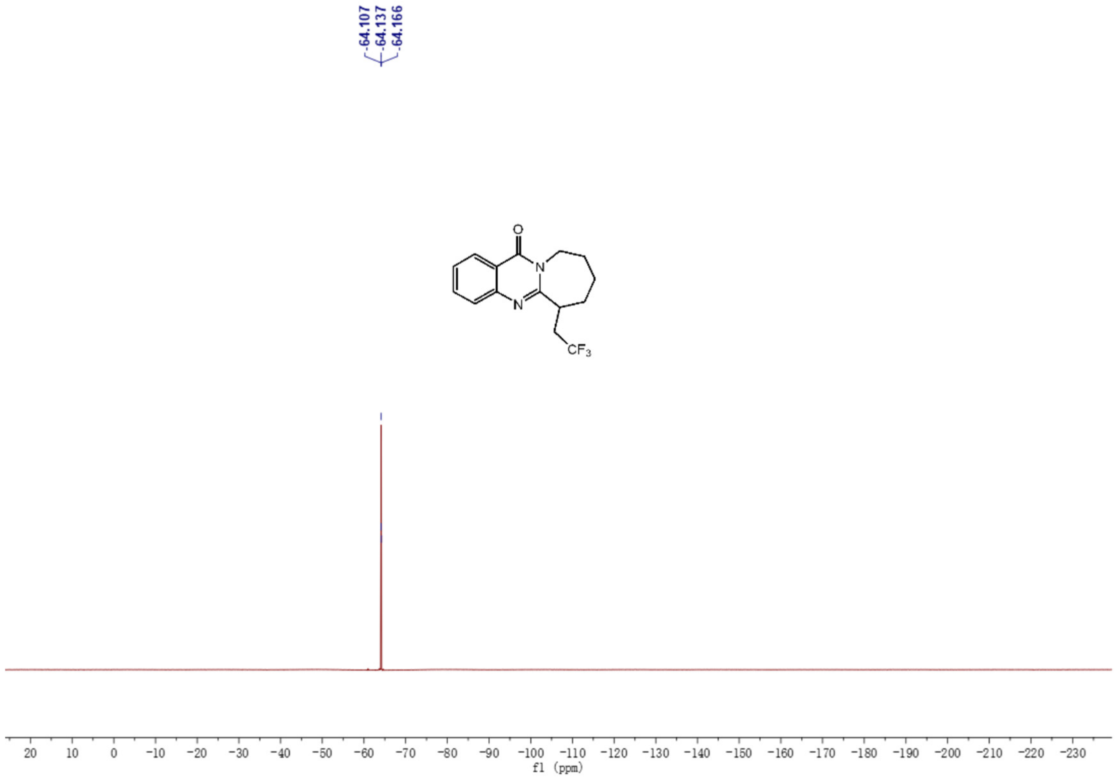

7-(2,2,2-trifluoroethyl)-7,8,9,10-tetrahydro-5H-dipyrido[1,2-*a*:3',2'-*e*]pyrimidin-5-one (**3w**) : ^{1}H NMR (400 MHz, $CDCl_3$)

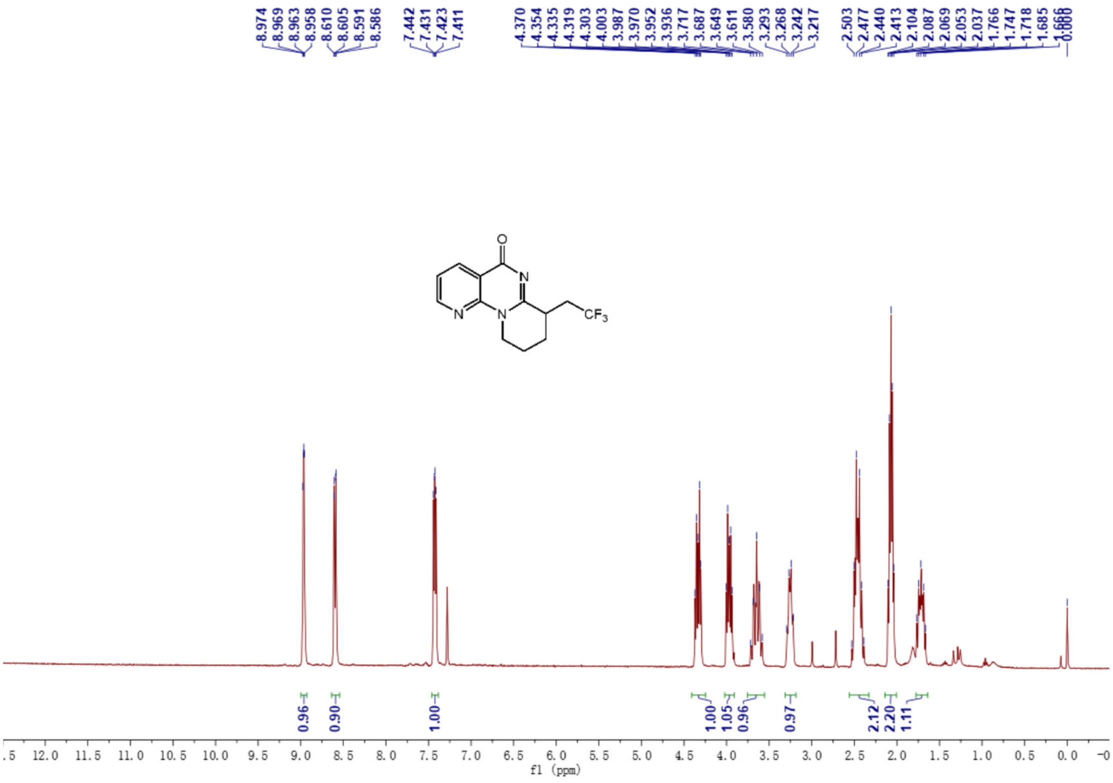

7-(2,2,2-trifluoroethyl)-7,8,9,10-tetrahydro-5H-dipyrido[1,2-*a*:3',2'-*e*]pyrimidin-5-one (**3w**) : ^{13}C NMR (150 MHz, $CDCl_3$)

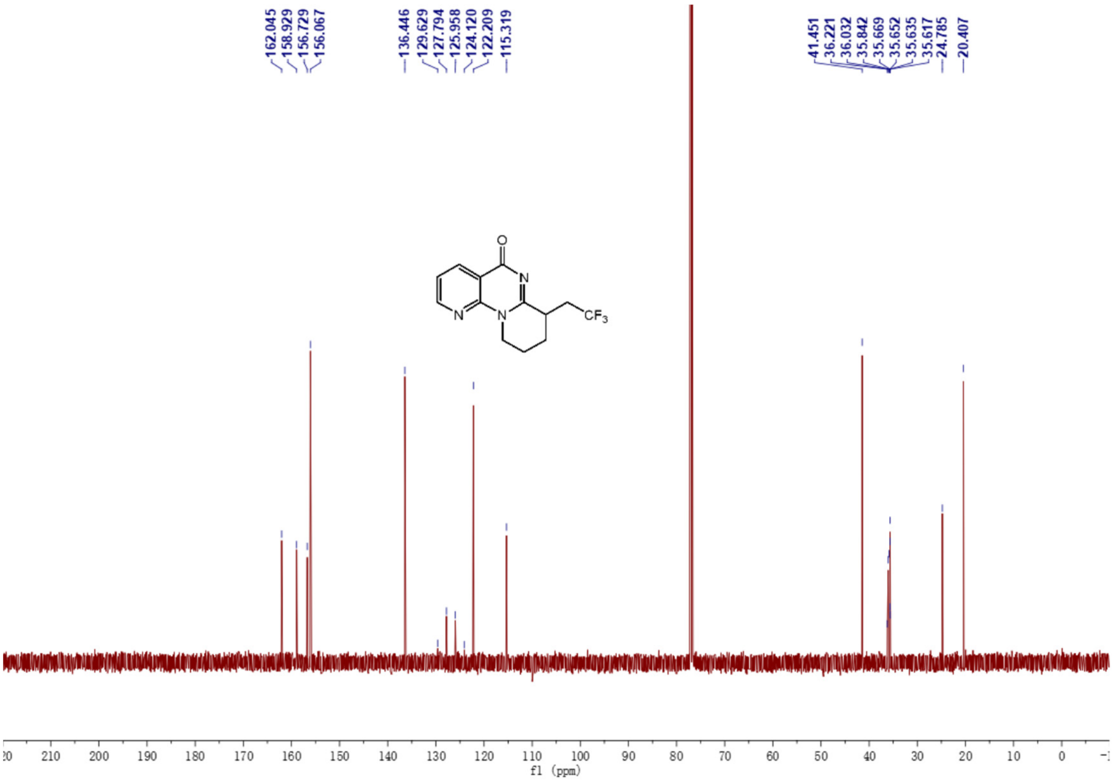

7-(2,2,2-trifluoroethyl)-7,8,9,10-tetrahydro-5H-dipyrido[1,2-*a*:3',2'-*e*]pyrimidin-5-one (**3w**) : ^{19}F NMR (376 MHz, $CDCl_3$)

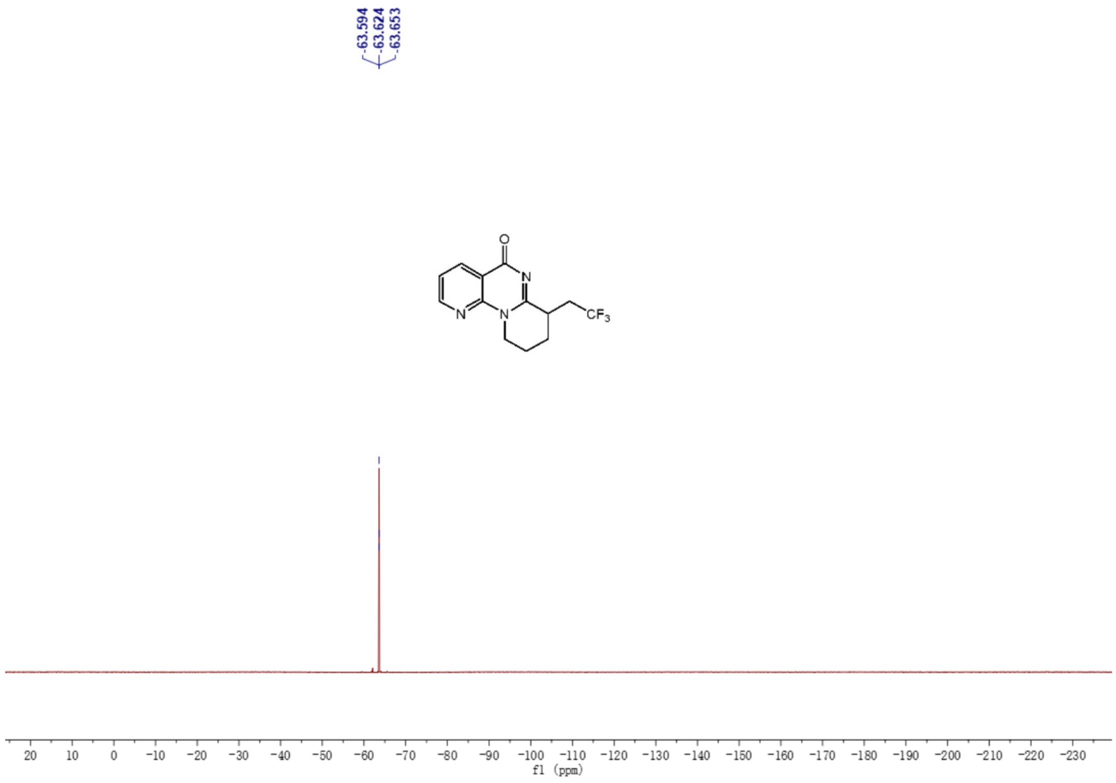

9-(2,2,2-trifluoroethyl)-6,7,8,9-tetrahydro-4H-pyrido[1,2-*a*]thieno[2,3-*d*]pyrimidin-4-one (**3x**) : ^{1}H NMR (400 MHz, $CDCl_3$)

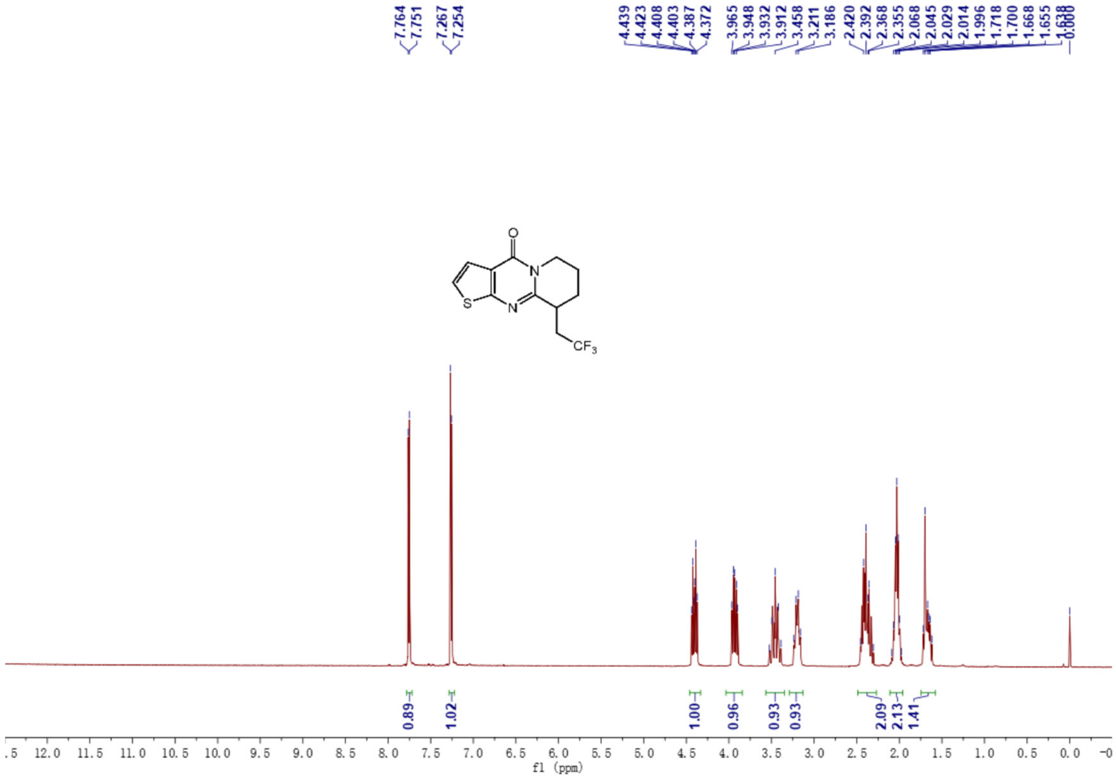

9-(2,2,2-trifluoroethyl)-6,7,8,9-tetrahydro-4H-pyrido[1,2-*a*]thieno[2,3-*d*]pyrimidin-4-one (**3x**) **:** ^{13}C NMR (150 MHz, $CDCl_3$)

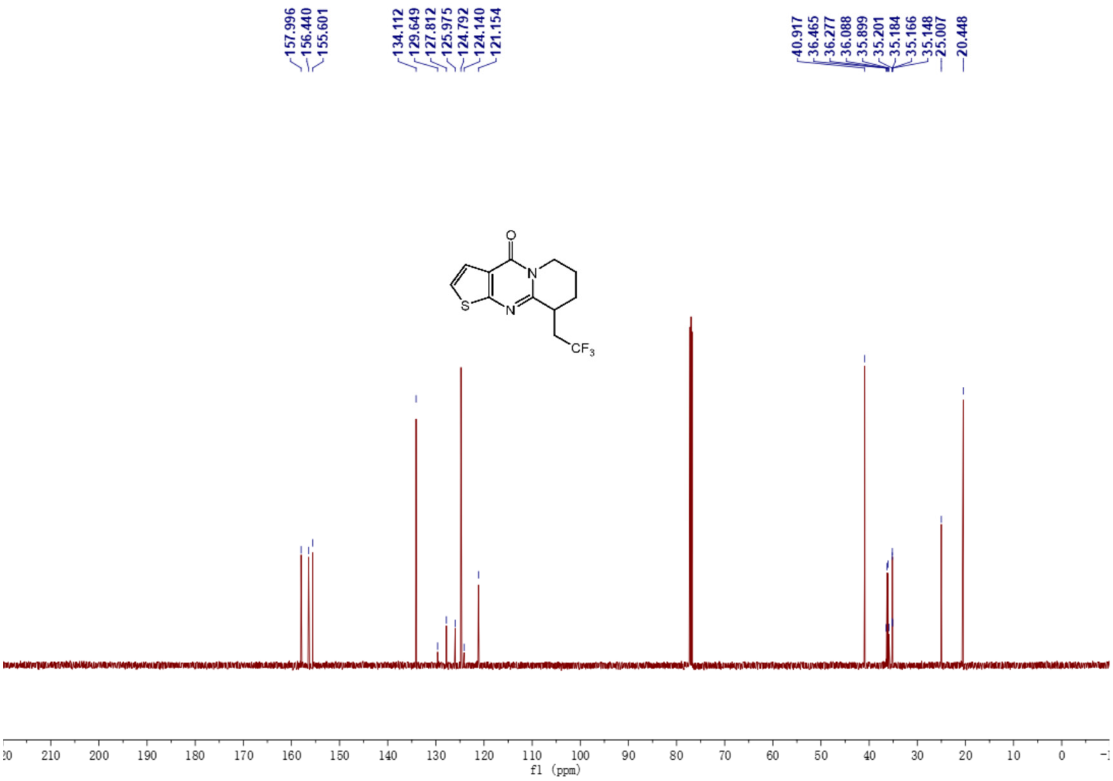

9-(2,2,2-trifluoroethyl)-6,7,8,9-tetrahydro-4H-pyrido[1,2-*a*]thieno[2,3-*d*]pyrimidin-4-one (**3x**) : ^{19}F NMR (376 MHz, $CDCl_3$)

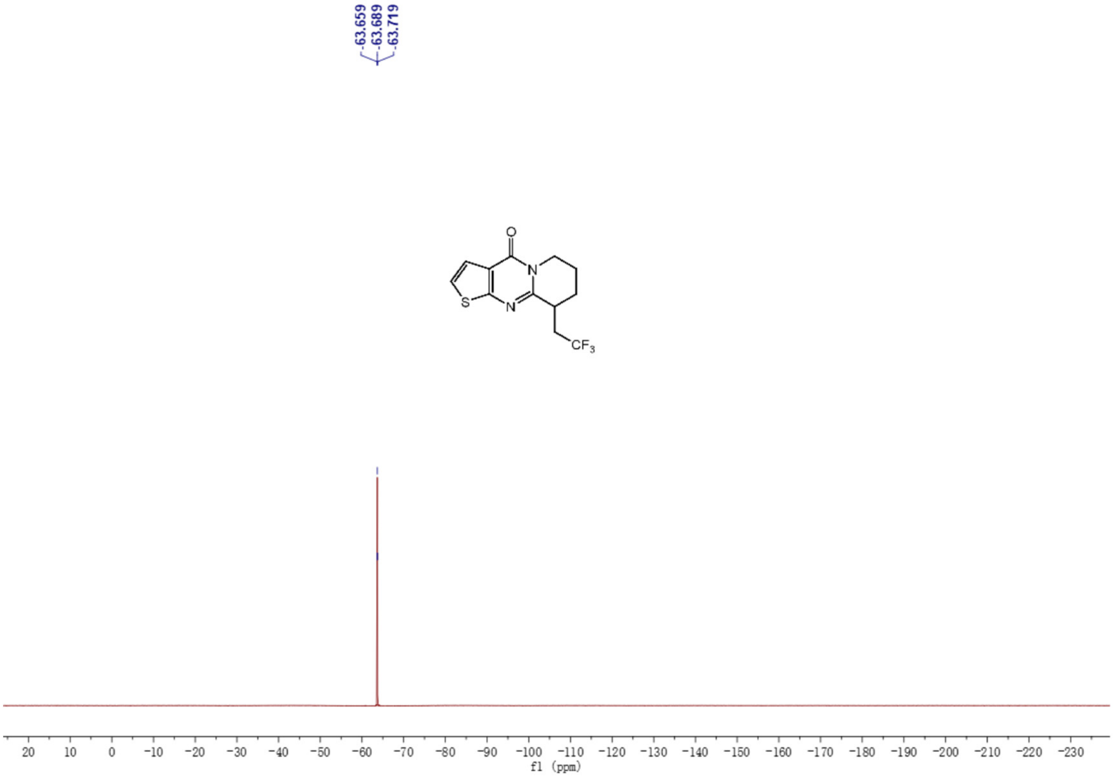

1-(1-(2,2,2-trifluoroethyl)-2,3-dihydro-1H-pyrrolizin-5-yl)ethan-1-one (**5a**) : ^{1}H NMR (400 MHz, $CDCl_3$)

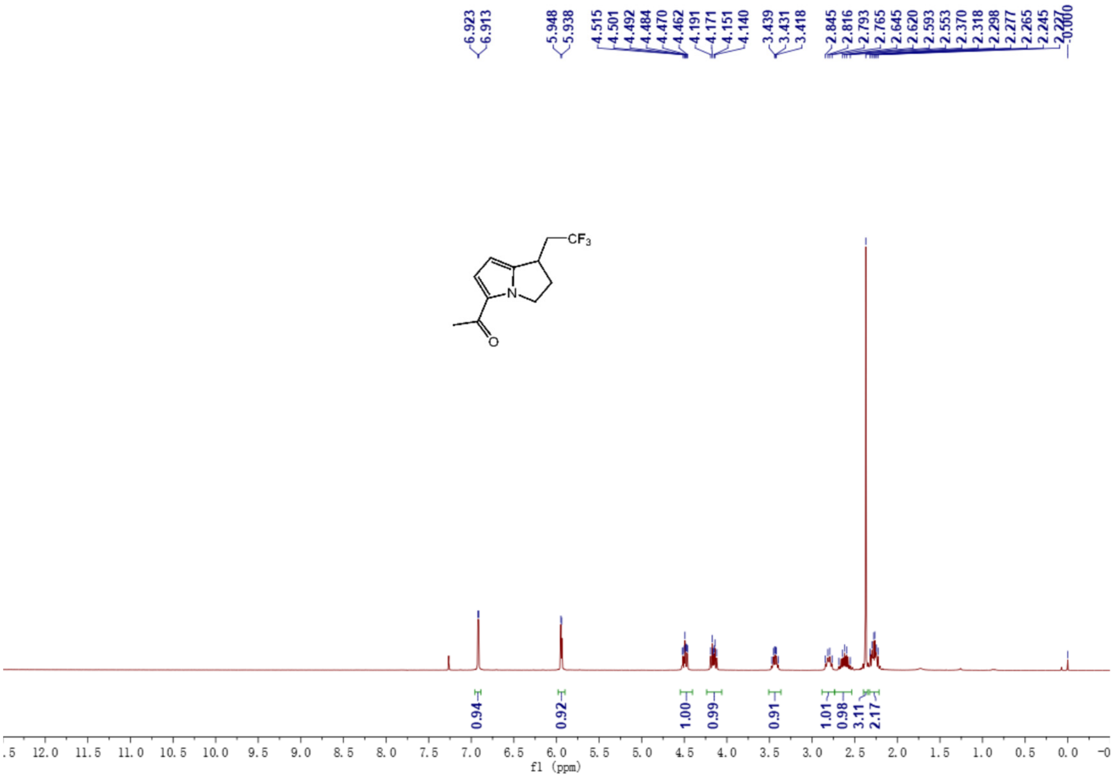

1-(1-(2,2,2-trifluoroethyl)-2,3-dihydro-1H-pyrrolizin-5-yl)ethan-1-one (**5a**) : ^{13}C NMR (150 MHz, $CDCl_3$)

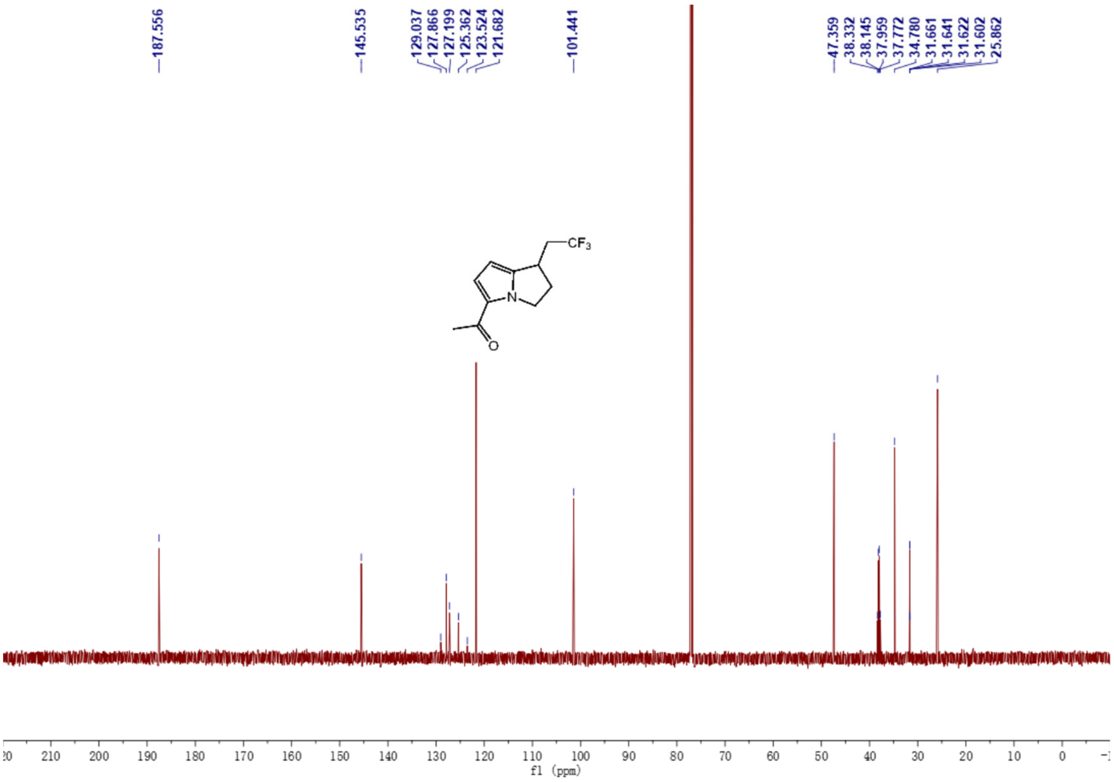

1-(1-(2,2,2-trifluoroethyl)-2,3-dihydro-1H-pyrrolizin-5-yl)ethan-1-one (**5a**) : ^{19}F NMR (376 MHz, $CDCl_3$)

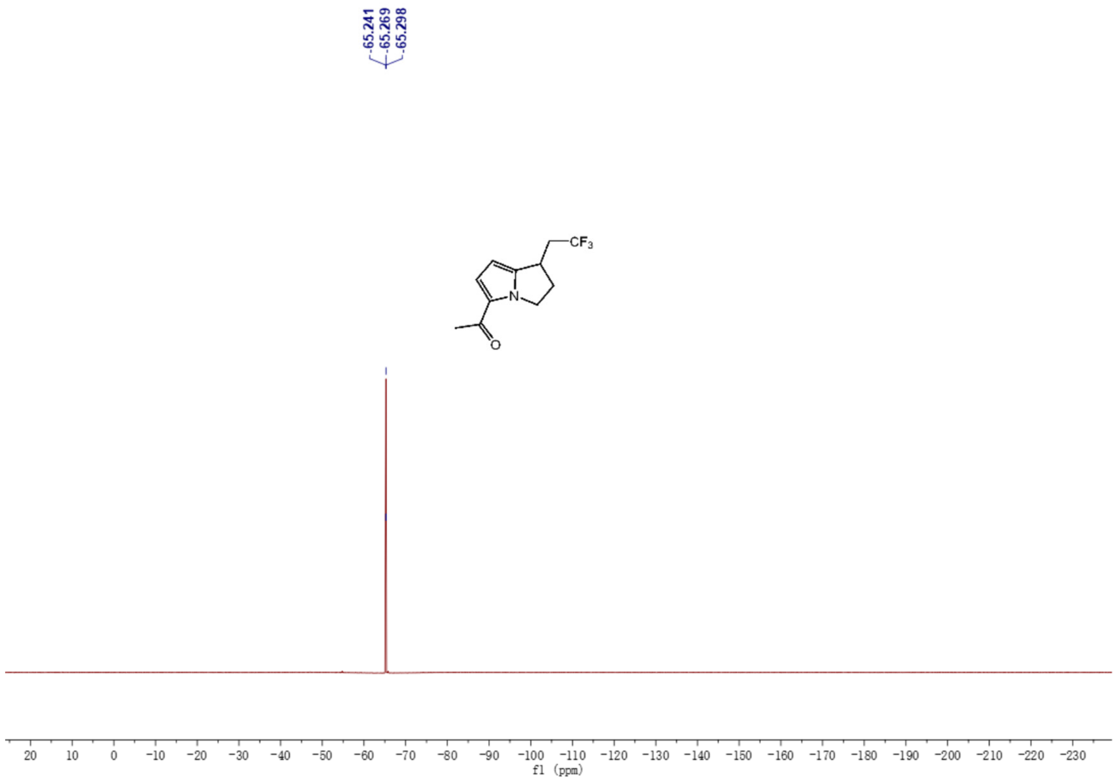

1-(8-(2,2,2-trifluoroethyl)-5,6,7,8-tetrahydroindolizin-3-yl)ethan-1-one (**5b**) : ^{1}H NMR (400 MHz, $CDCl_3$)

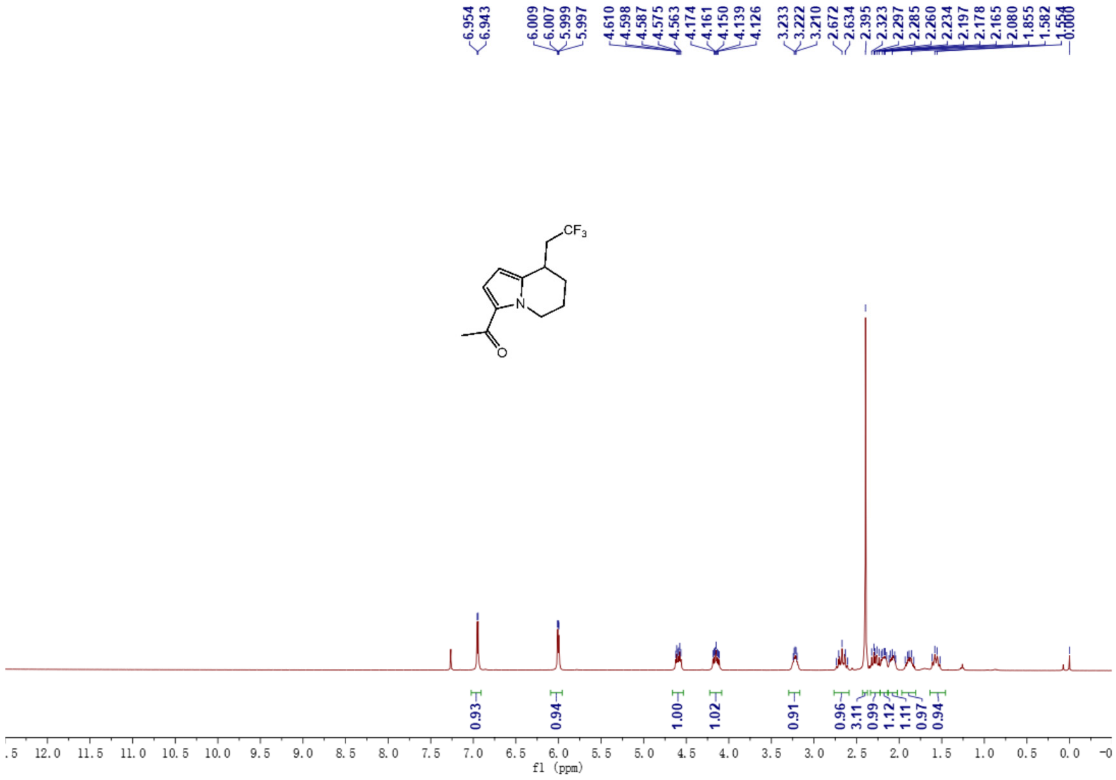

1-(8-(2,2,2-trifluoroethyl)-5,6,7,8-tetrahydroindolizin-3-yl)ethan-1-one (**5b**) **:** ^{13}C NMR (150 MHz, $CDCl_3$)

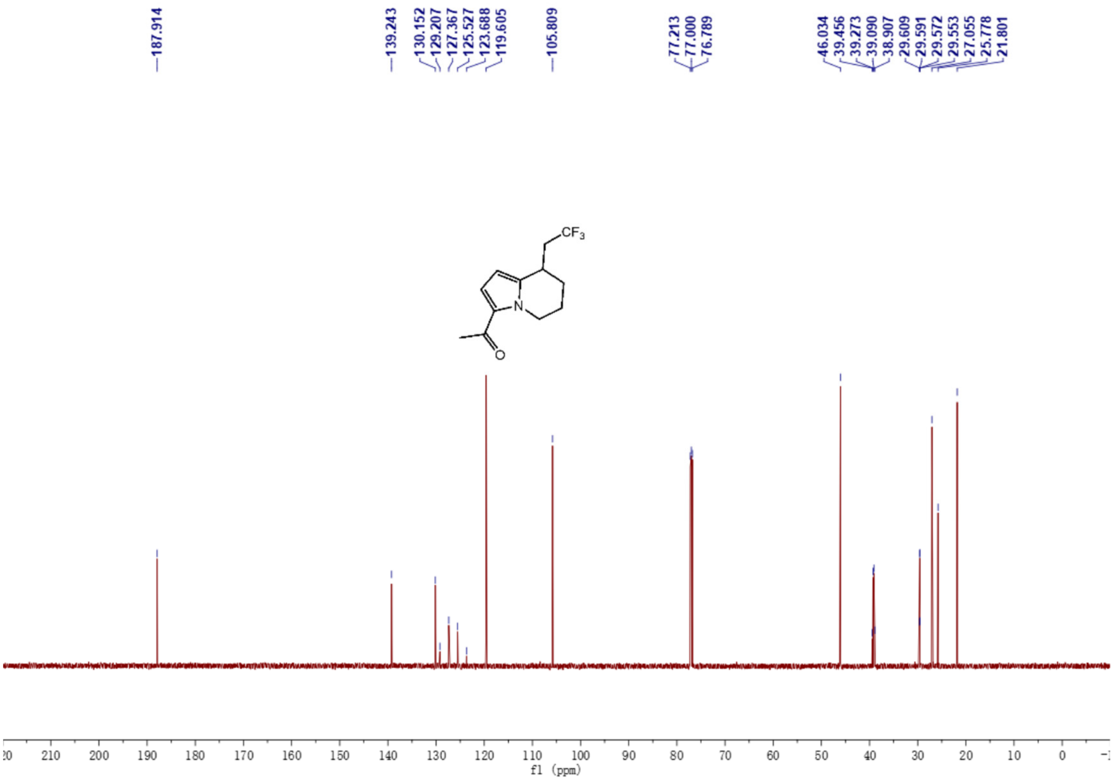

1-(8-(2,2,2-trifluoroethyl)-5,6,7,8-tetrahydroindolizin-3-yl)ethan-1-one (**5b**) **:** ^{19}F NMR (376 MHz, $CDCl_3$)

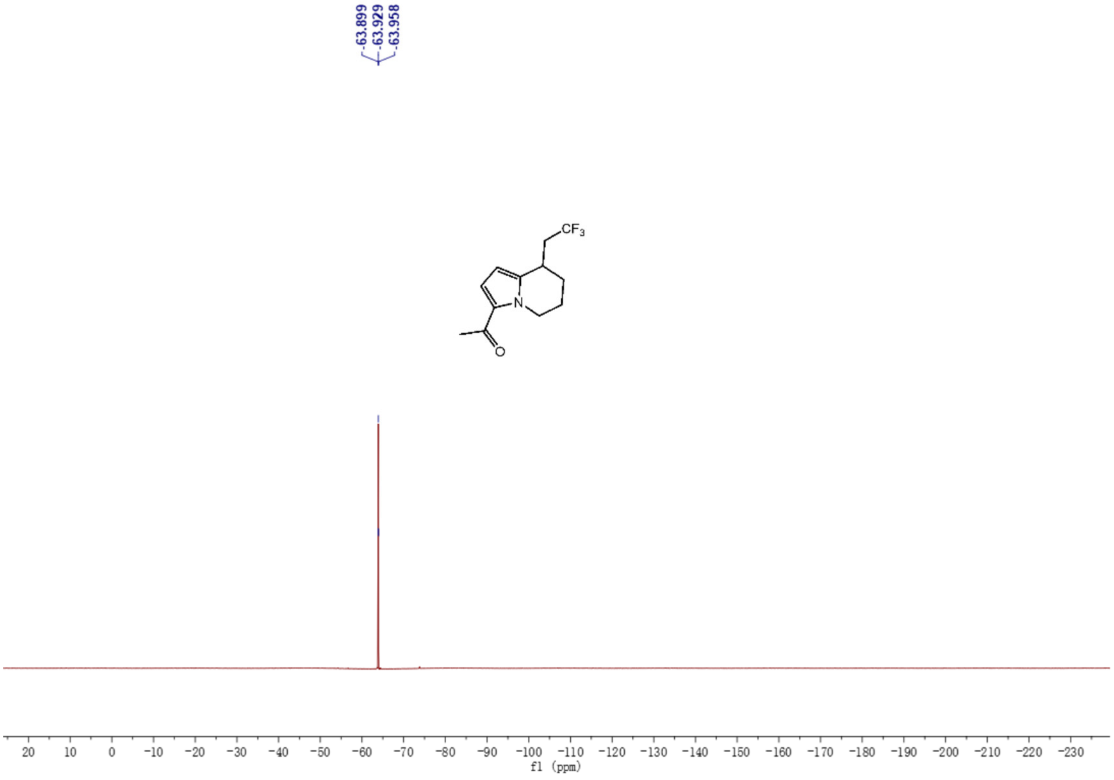

1-(9-(2,2,2-trifluoroethyl)-6,7,8,9-tetrahydropyrido[1,2-*a*]indol-10-yl)ethan-1-one (**5e**) : ^{1}H NMR (400 MHz, $CDCl_3$)

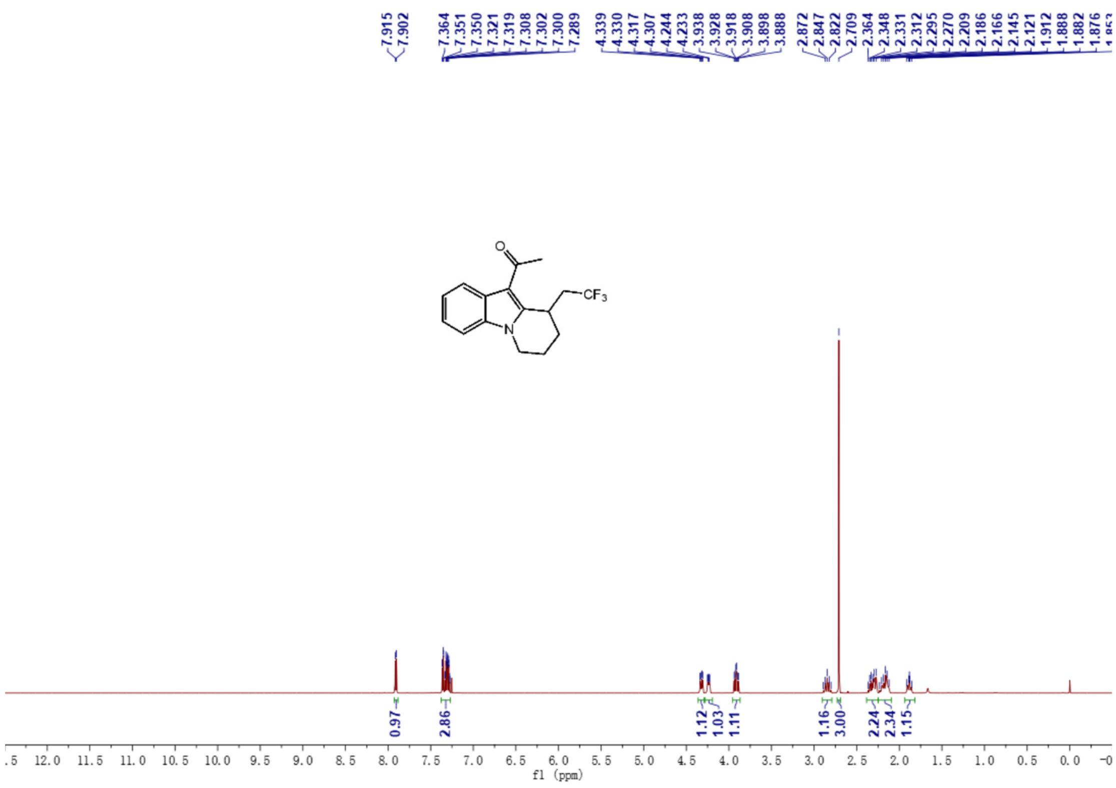

1-(9-(2,2,2-trifluoroethyl)-6,7,8,9-tetrahydropyrido[1,2-*a*]indol-10-yl)ethan-1-one (**5e**) : ^{13}C NMR (150 MHz, CDCl$_3$)

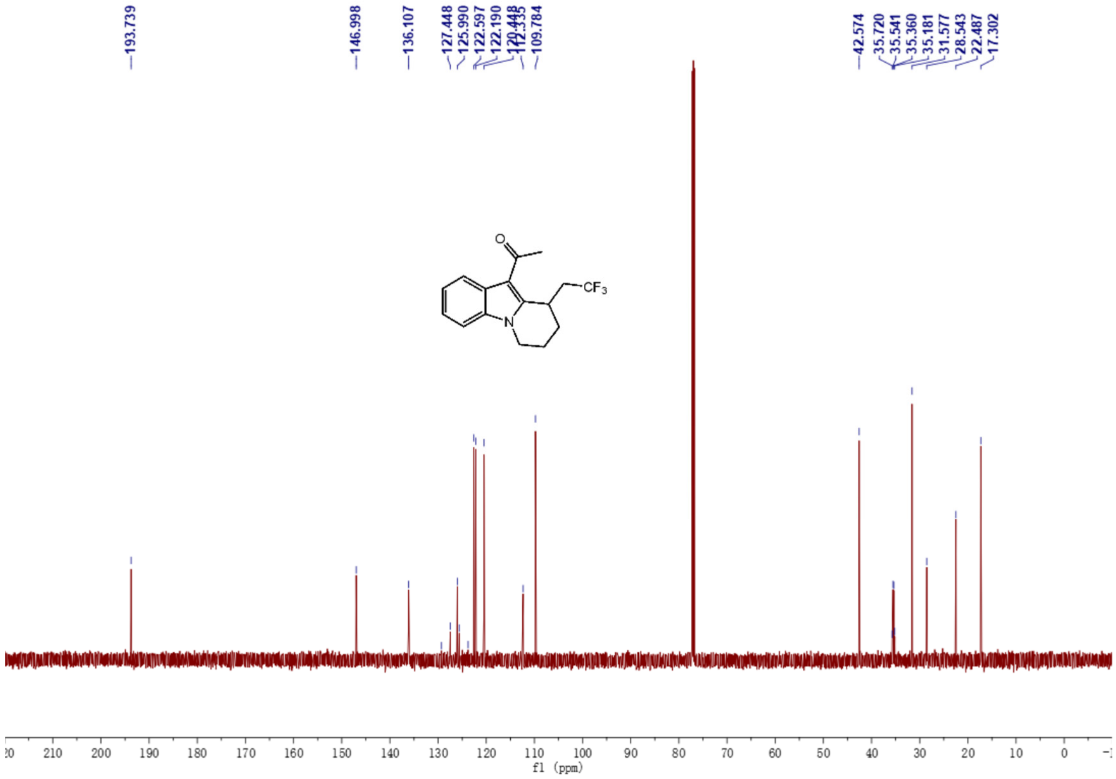

1-(9-(2,2,2-trifluoroethyl)-6,7,8,9-tetrahydropyrido[1,2-*a*]indol-10-yl)ethan-1-one

(**5e**) : ^{19}F NMR (376 MHz, CDCl$_3$)

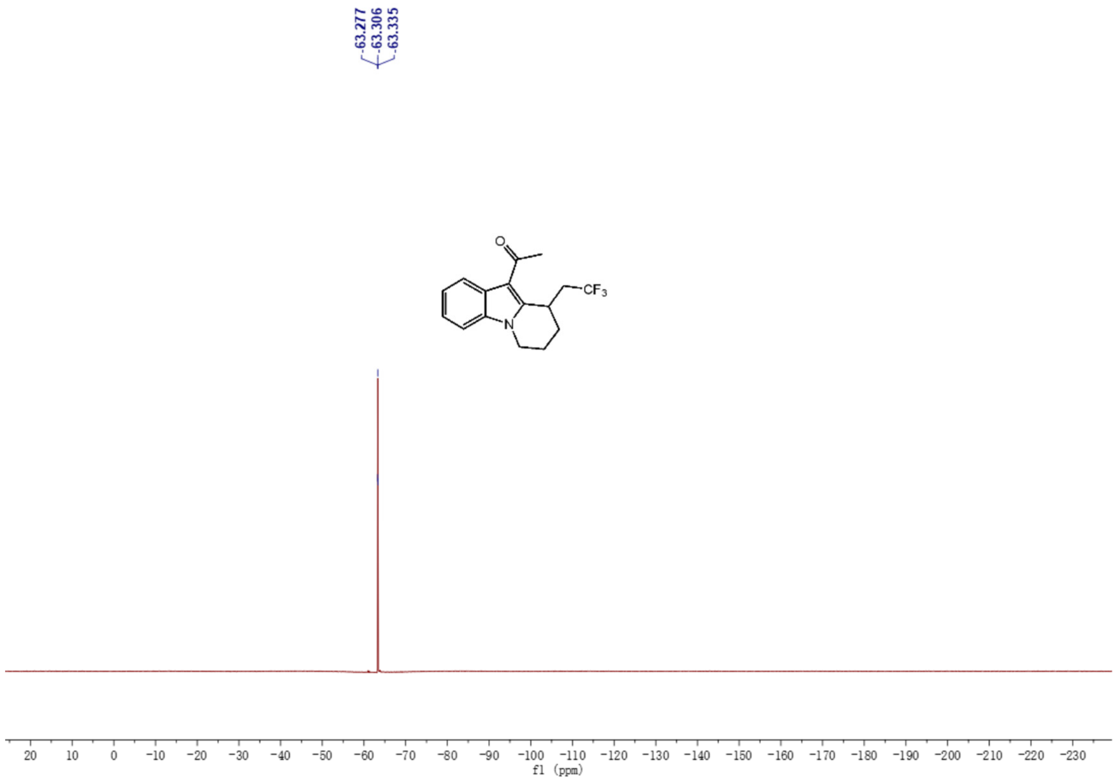

1-(1-(2,2,2-trifluoroethyl)-2,3-dihydro-1H-pyrrolo[1,2-*a*]indol-9-yl)ethan-1-one (**5f**) : ^{1}H NMR (400 MHz, $CDCl_3$)

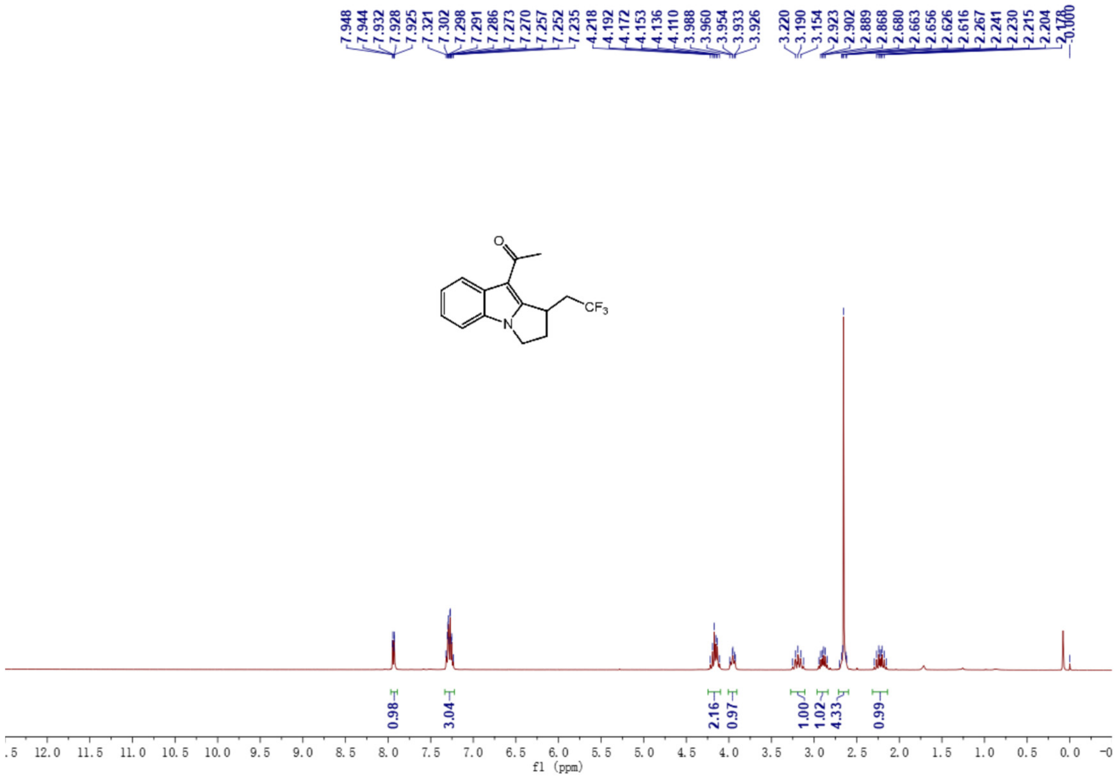

1-(1-(2,2,2-trifluoroethyl)-2,3-dihydro-1H-pyrrolo[1,2-*a*]indol-9-yl)ethan-1-one (**5f**) : ^{13}C NMR (150 MHz, $CDCl_3$)

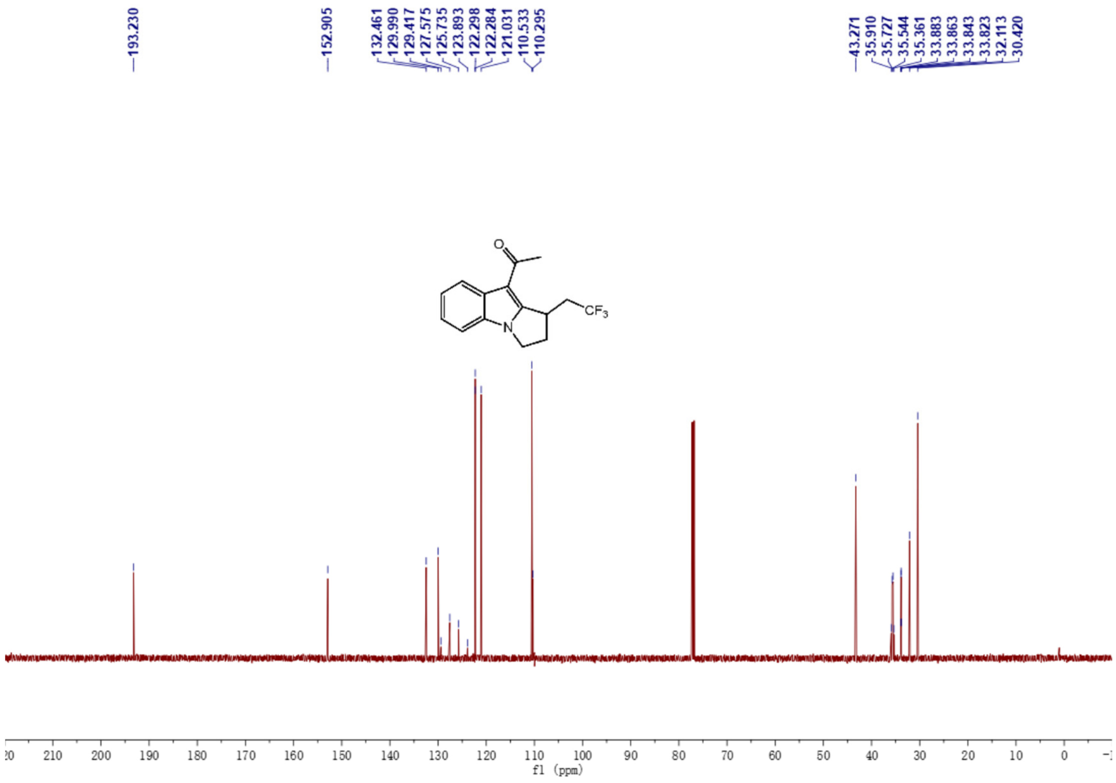

1-(1-(2,2,2-trifluoroethyl)-2,3-dihydro-1H-pyrrolo[1,2-*a*]indol-9-yl)ethan-1-one (**5f**) : ^{19}F NMR (376 MHz, $CDCl_3$)

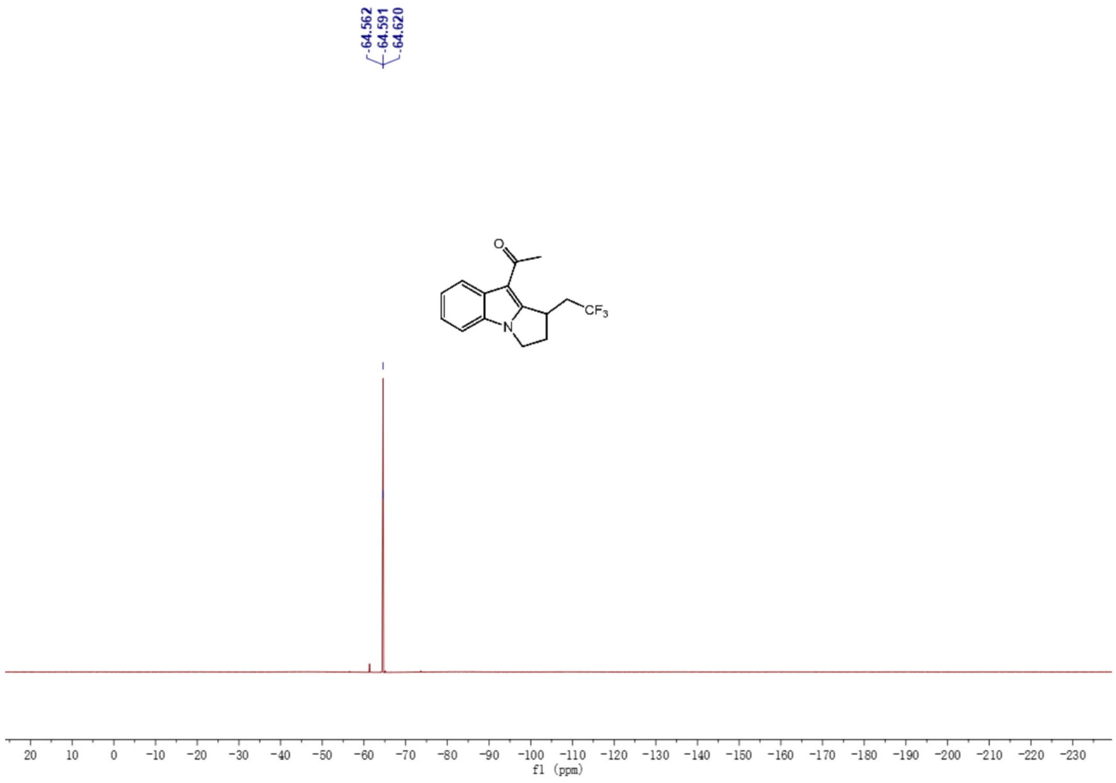

2,2,2-trifluoro-1-(1-(2,2,2-trifluoroethyl)-2,3-dihydro-1H-pyrrolo[1,2-*a*]indol-9-yl)ethan-1-one (**5g**) : ^{1}H NMR (400 MHz, $CDCl_3$)

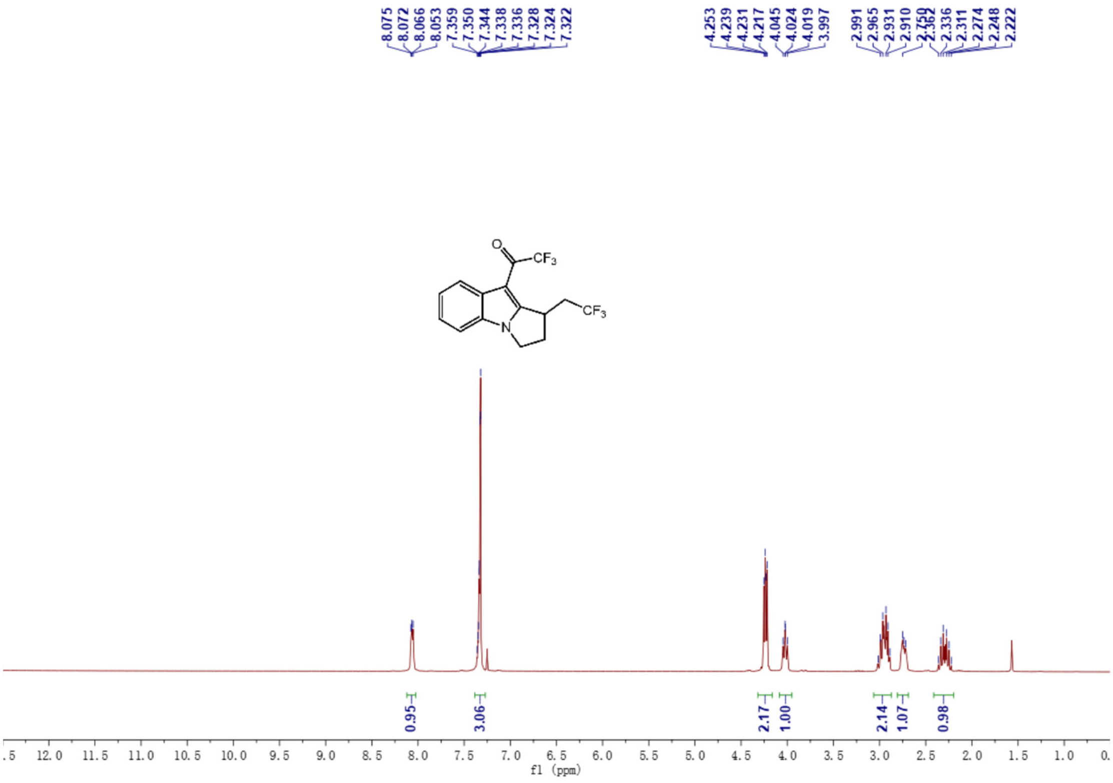

2,2,2-trifluoro-1-(1-(2,2,2-trifluoroethyl)-2,3-dihydro-1H-pyrrolo[1,2-*a*]indol-9-yl)ethan-1-one (**5g**) : ^{13}C NMR (150 MHz, $CDCl_3$)

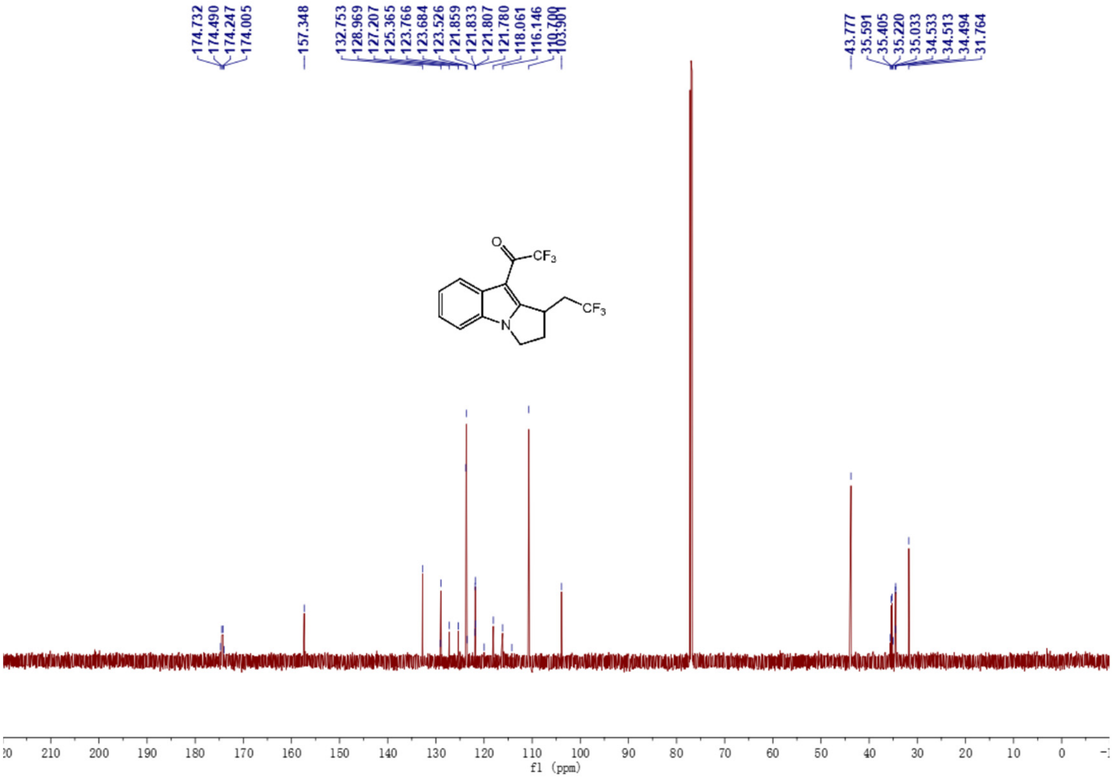

2,2,2-trifluoro-1-(1-(2,2,2-trifluoroethyl)-2,3-dihydro-1H-pyrrolo[1,2-*a*]indol-9-yl)ethan-1-one (**5g**) : ^{19}F NMR (376 MHz, $CDCl_3$)

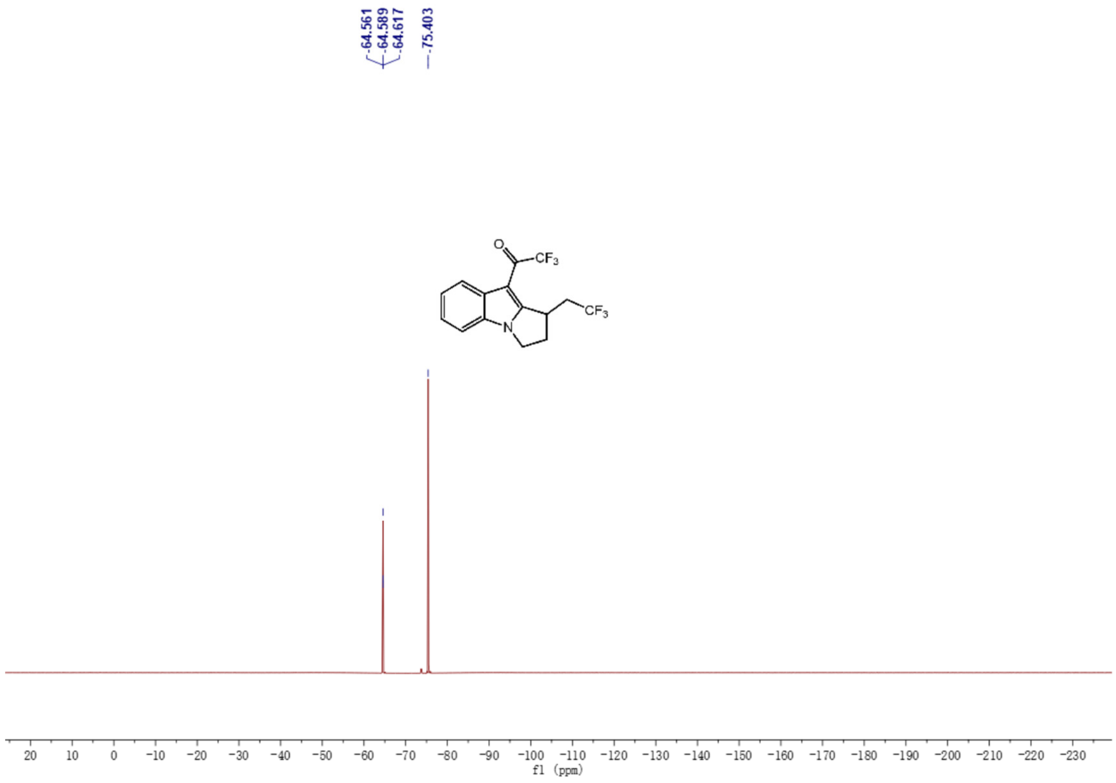

methyl 1-(2,2,2-trifluoroethyl)-2,3-dihydro-1H-pyrrolo[1,2-*a*]indole-9-carboxylate (**5h**) : ^{1}H NMR (400 MHz, $CDCl_3$)

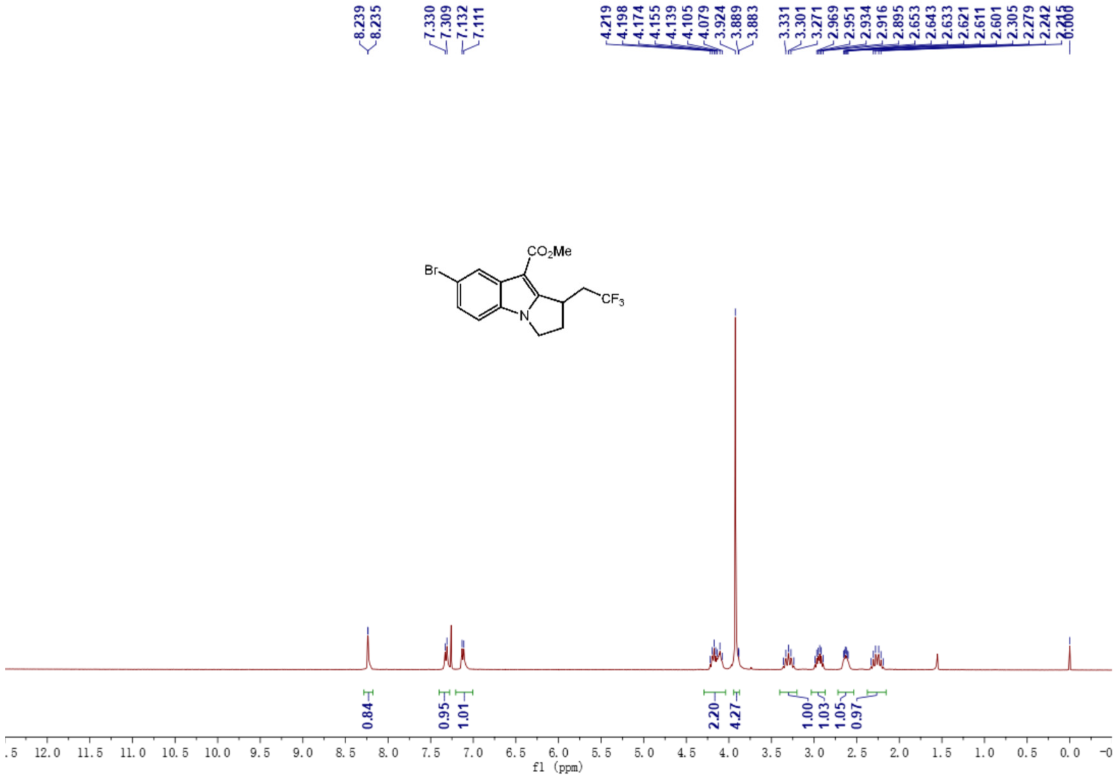

methyl 1-(2,2,2-trifluoroethyl)-2,3-dihydro-1H-pyrrolo[1,2-*a*]indole-9-carboxylate (**5h**) : ^{13}C NMR (150 MHz, $CDCl_3$)

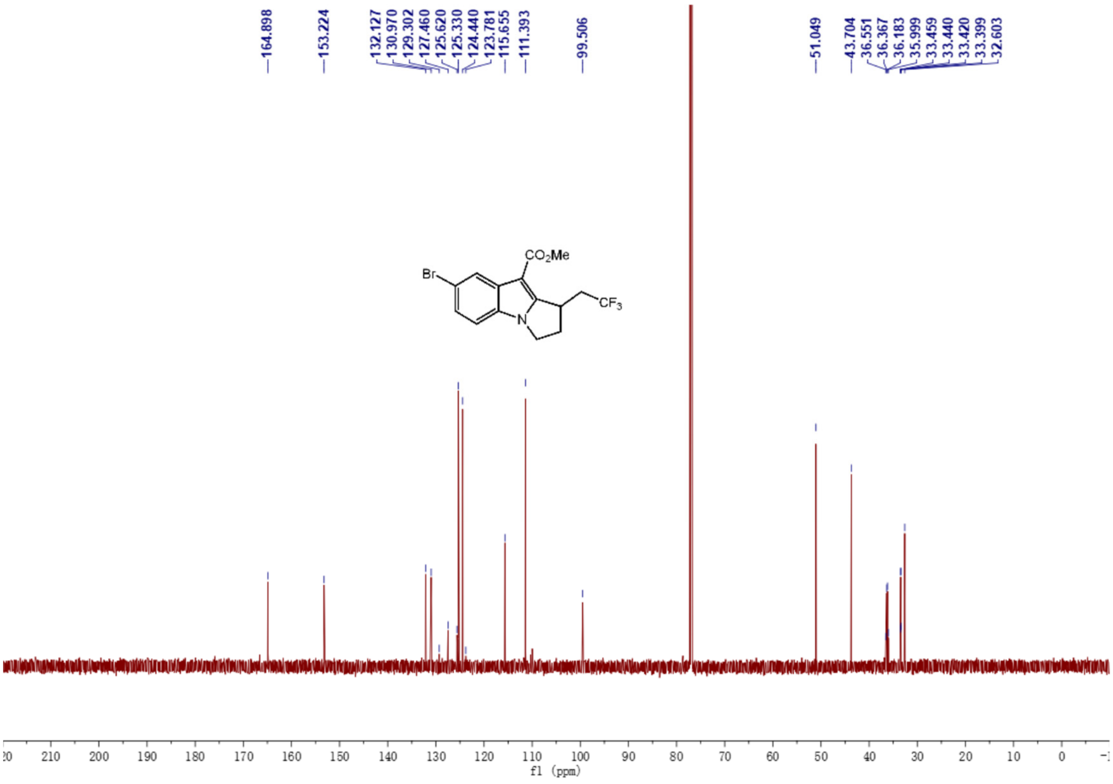

methyl 1-(2,2,2-trifluoroethyl)-2,3-dihydro-1H-pyrrolo[1,2-*a*]indole-9-carboxylate (**5h**) : ^{19}F NMR (376 MHz, $CDCl_3$)

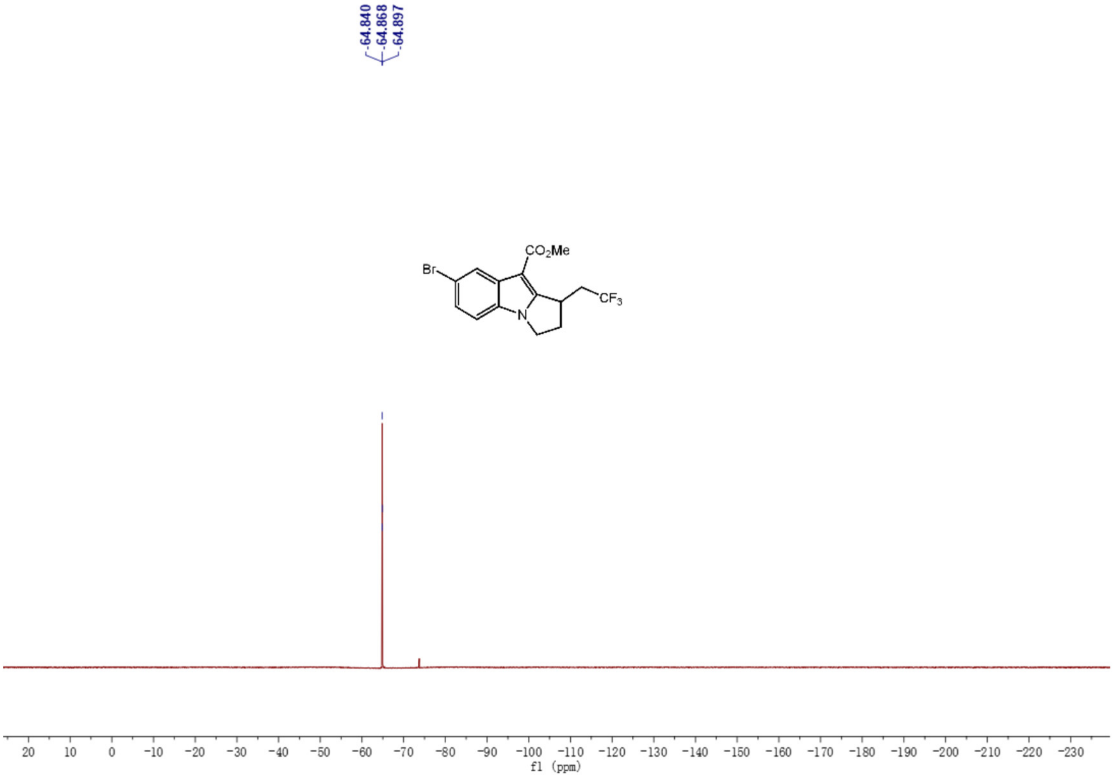

4-(2,2,2-trifluoroethyl)-1,2,3,4-tetrahydrobenzo[4,5]imidazo[1,2-*a*]pyridine (**7a**) : ^{1}H NMR (400 MHz, $CDCl_3$)

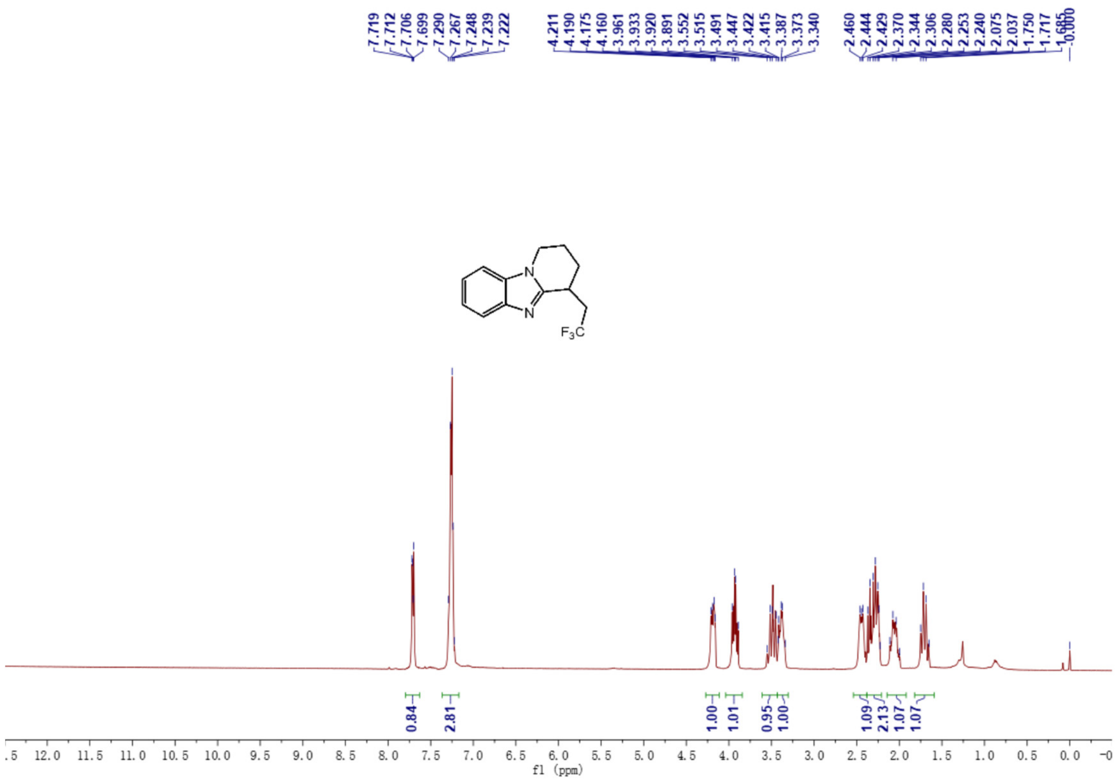

4-(2,2,2-trifluoroethyl)-1,2,3,4-tetrahydrobenzo[4,5]imidazo[1,2-*a*]pyridine (**7a**) : ^{13}C NMR (150 MHz, $CDCl_3$)

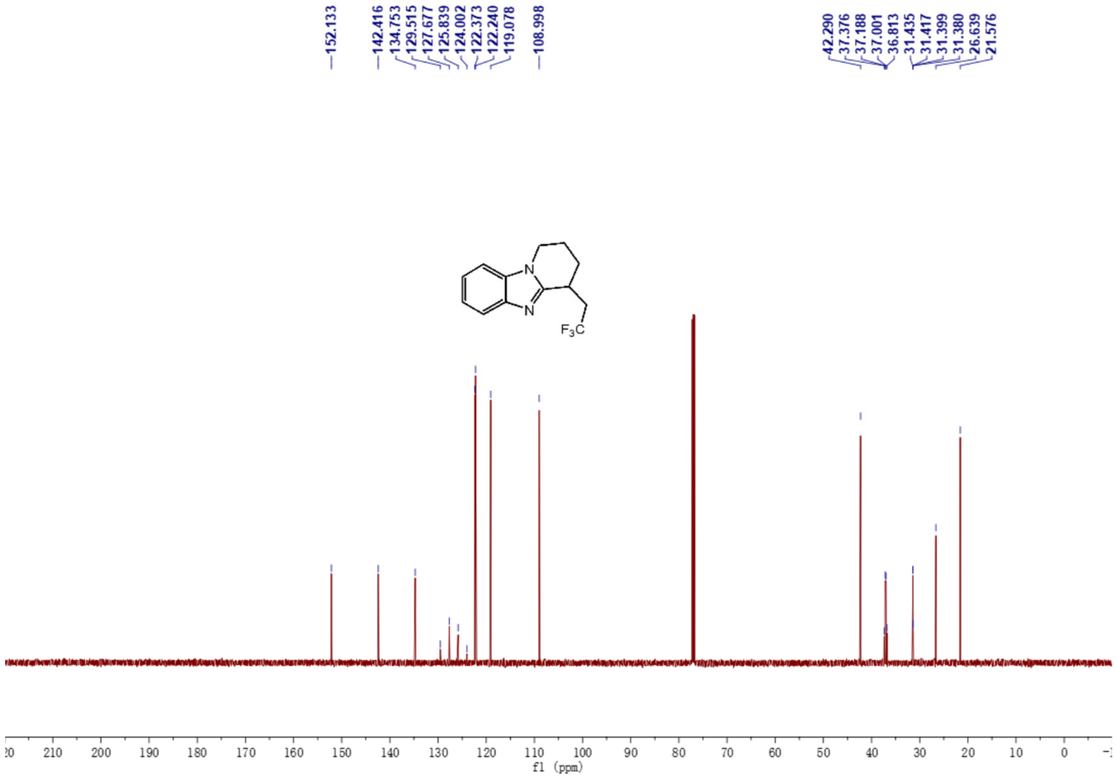

4-(2,2,2-trifluoroethyl)-1,2,3,4-tetrahydrobenzo[4,5]imidazo[1,2-*a*]pyridine (**7a**) : ^{19}F NMR (376 MHz, $CDCl_3$)

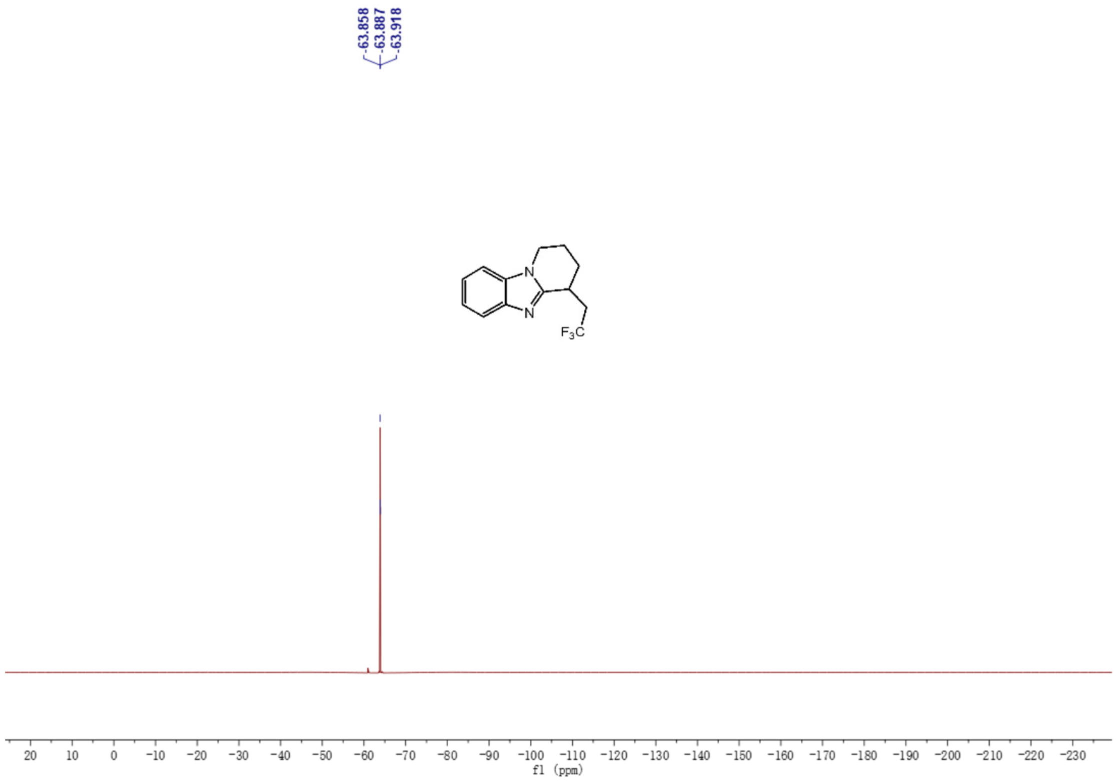

8-methyl-4-(2,2,2-trifluoroethyl)-1,2,3,4-tetrahydrobenzo[4,5]imidazo[1,2-*a*]pyridine (**7b**) : ^{1}H NMR (400 MHz, $CDCl_3$)

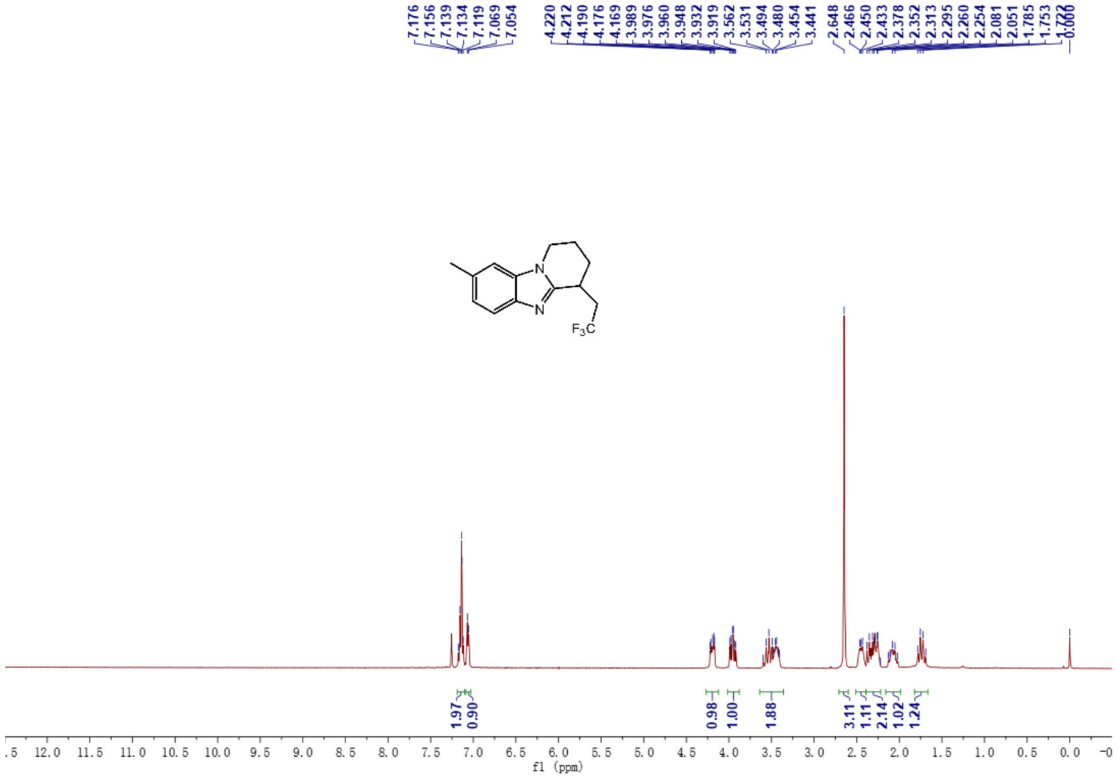

8-methyl-4-(2,2,2-trifluoroethyl)-1,2,3,4-tetrahydrobenzo[4,5]imidazo[1,2-*a*]pyridine (**7b**) **:** ^{13}C NMR (150 MHz, $CDCl_3$)

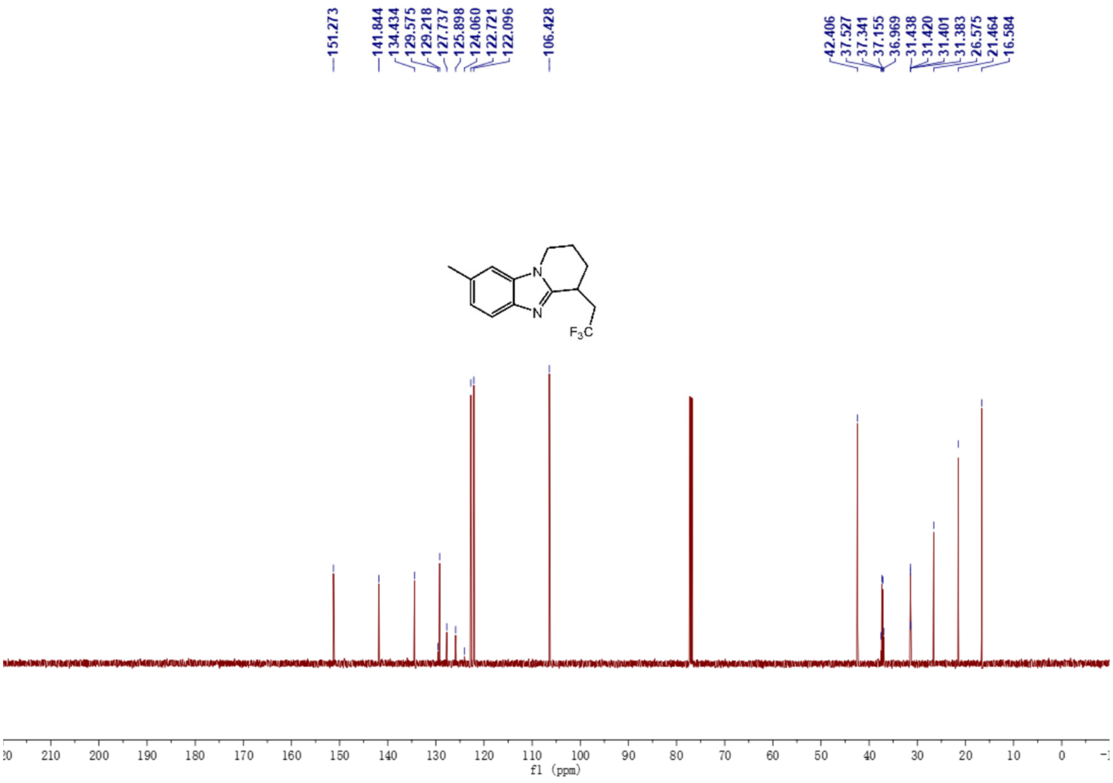

8-methyl-4-(2,2,2-trifluoroethyl)-1,2,3,4-tetrahydrobenzo[4,5]imidazo[1,2-*a*]pyridine (**7b**) : ^{19}F NMR (376 MHz, $CDCl_3$)

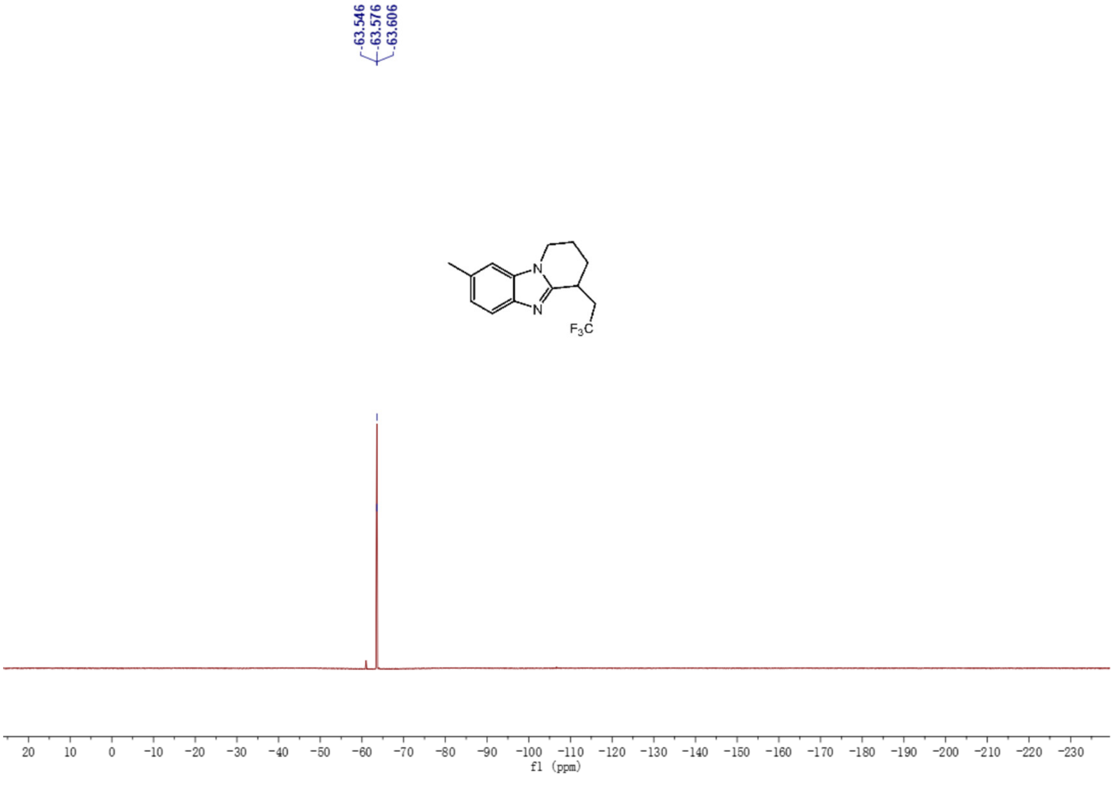

8-methoxy-4-(2,2,2-trifluoroethyl)-1,2,3,4-tetrahydrobenzo[4,5]imidazo[1,2-*a*]pyridine (**7c**) : ^{1}H NMR (400 MHz, $CDCl_3$)

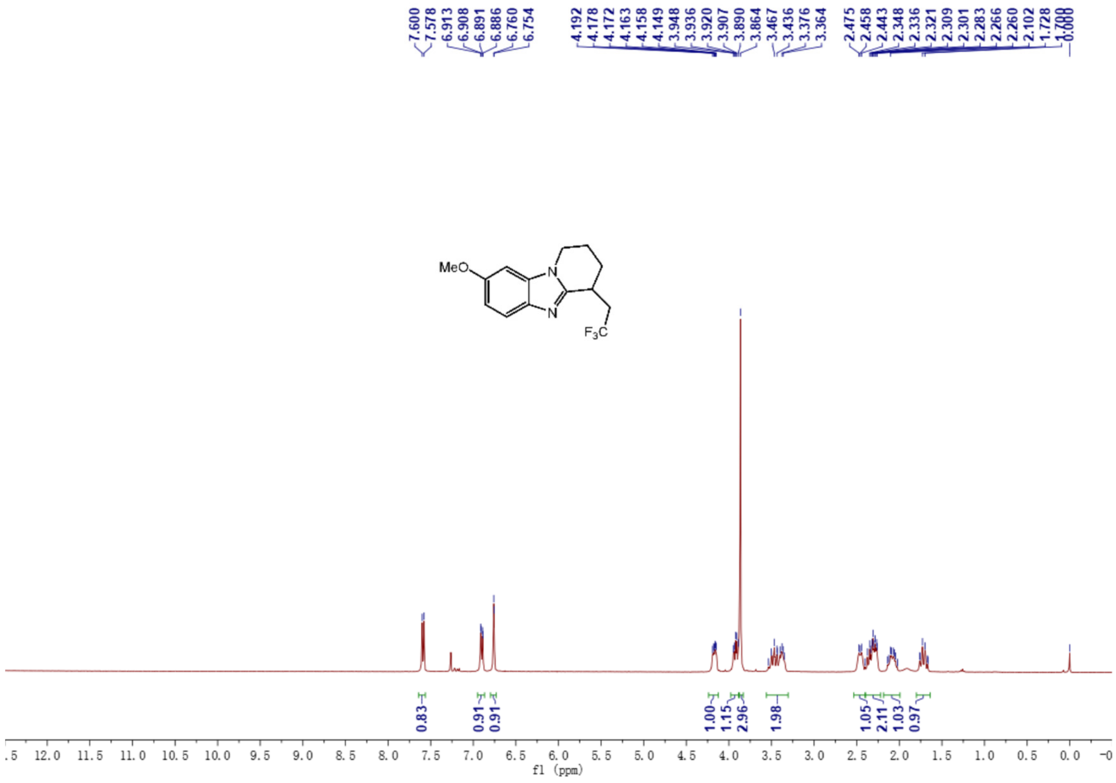

8-methoxy-4-(2,2,2-trifluoroethyl)-1,2,3,4-tetrahydrobenzo[4,5]imidazo[1,2-*a*]pyridine (**7c**) : ^{13}C NMR (150 MHz, CDCl$_3$)

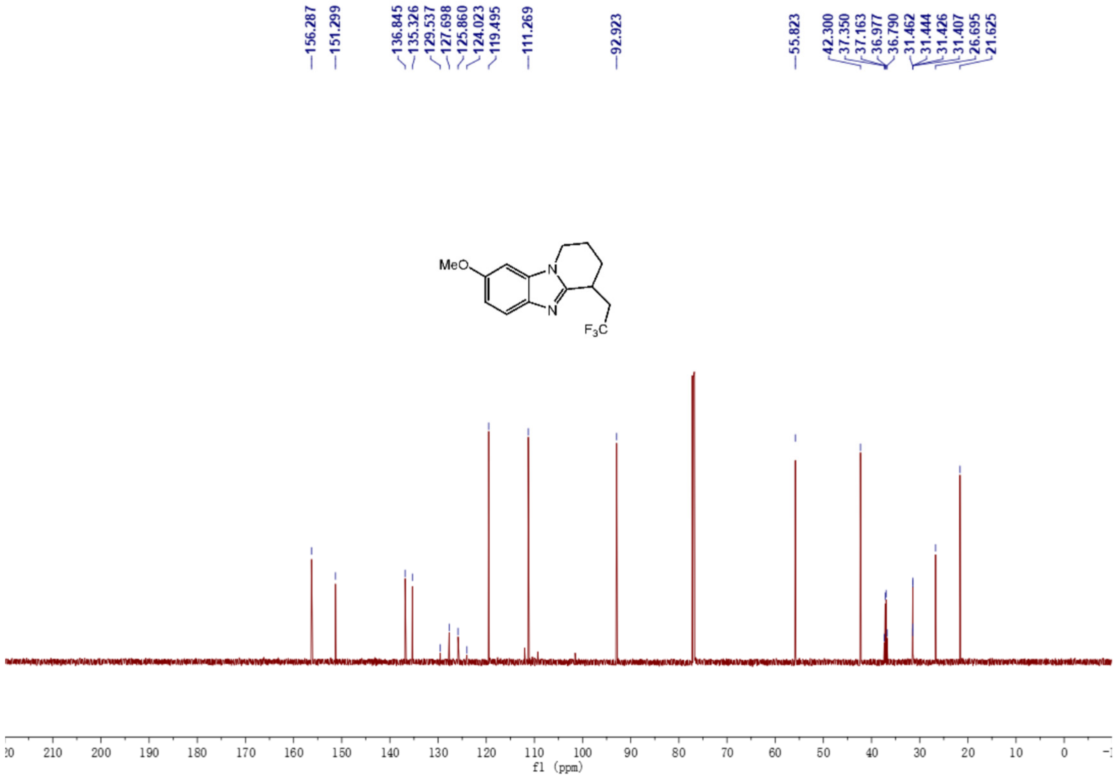

8-methoxy-4-(2,2,2-trifluoroethyl)-1,2,3,4-tetrahydrobenzo[4,5]imidazo[1,2-*a*]pyridine (**7c**) : ^{19}F NMR (376 MHz, CDCl$_3$)

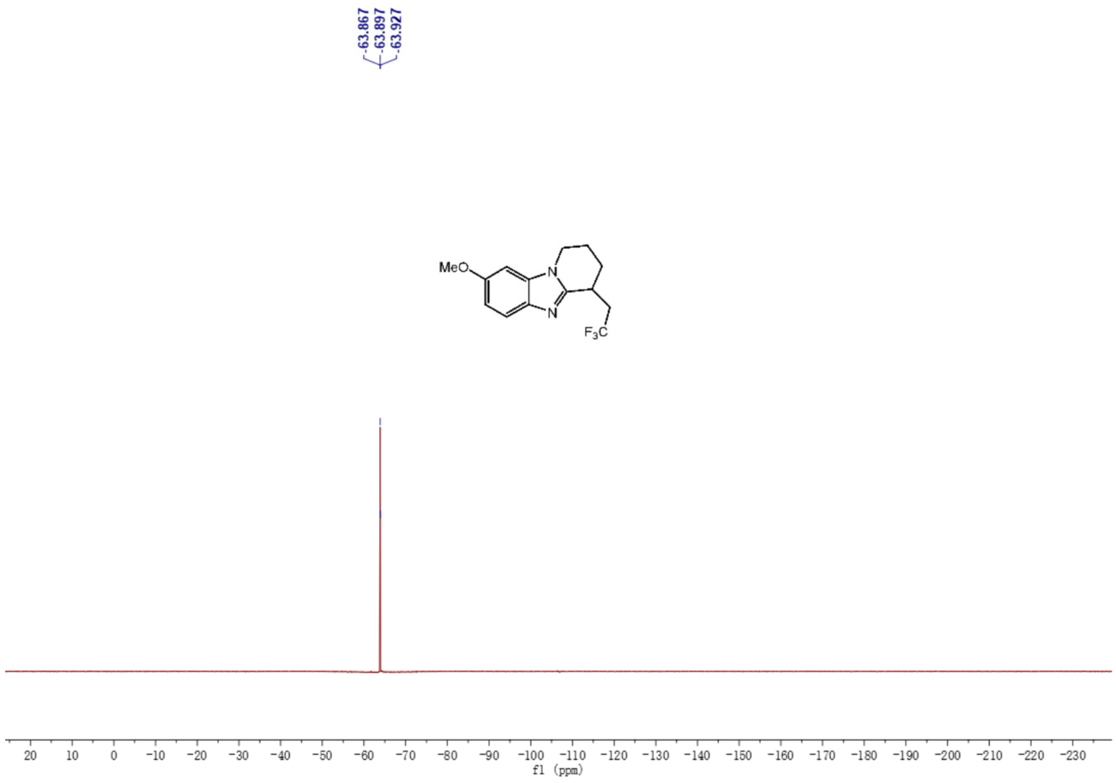

7,8-dimethoxy-4-(2,2,2-trifluoroethyl)-1,2,3,4-tetrahydrobenzo[4,5]imidazo[1,2-a]pyridine (**7d**) : ^{1}H NMR (400 MHz, $CDCl_3$)

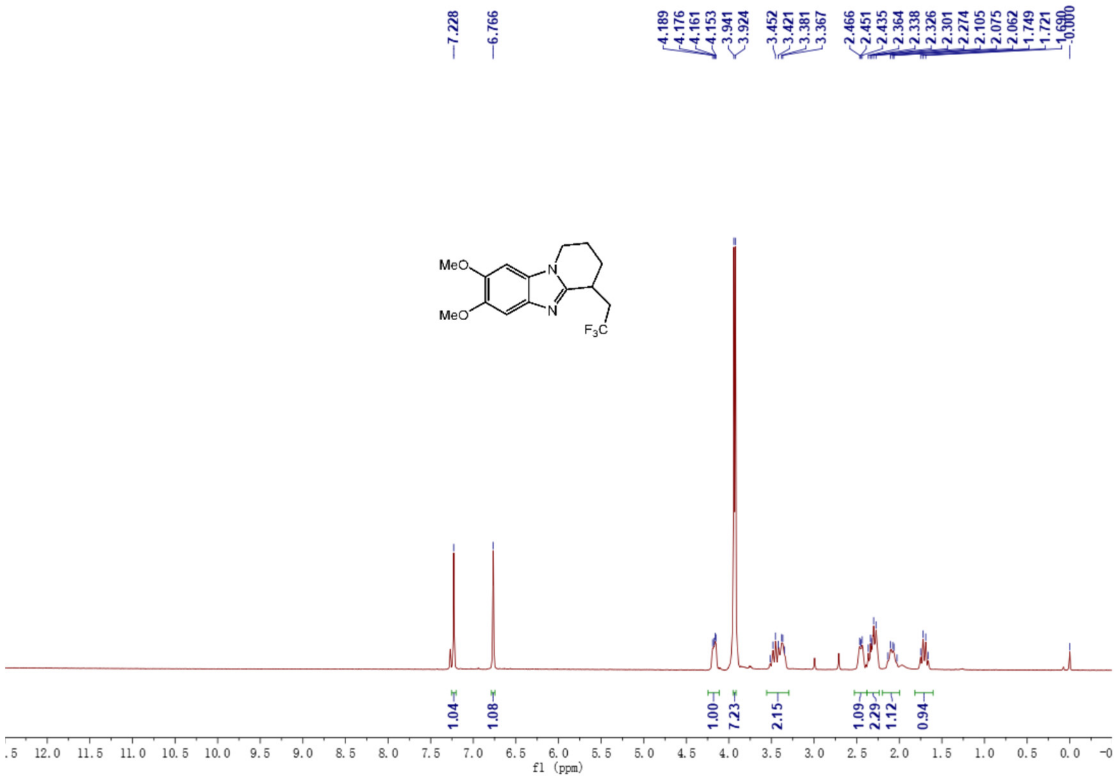

7,8-dimethoxy-4-(2,2,2-trifluoroethyl)-1,2,3,4-tetrahydrobenzo[4,5]imidazo[1,2-a]pyridine (**7d**) : ^{13}C NMR (150 MHz, $CDCl_3$)

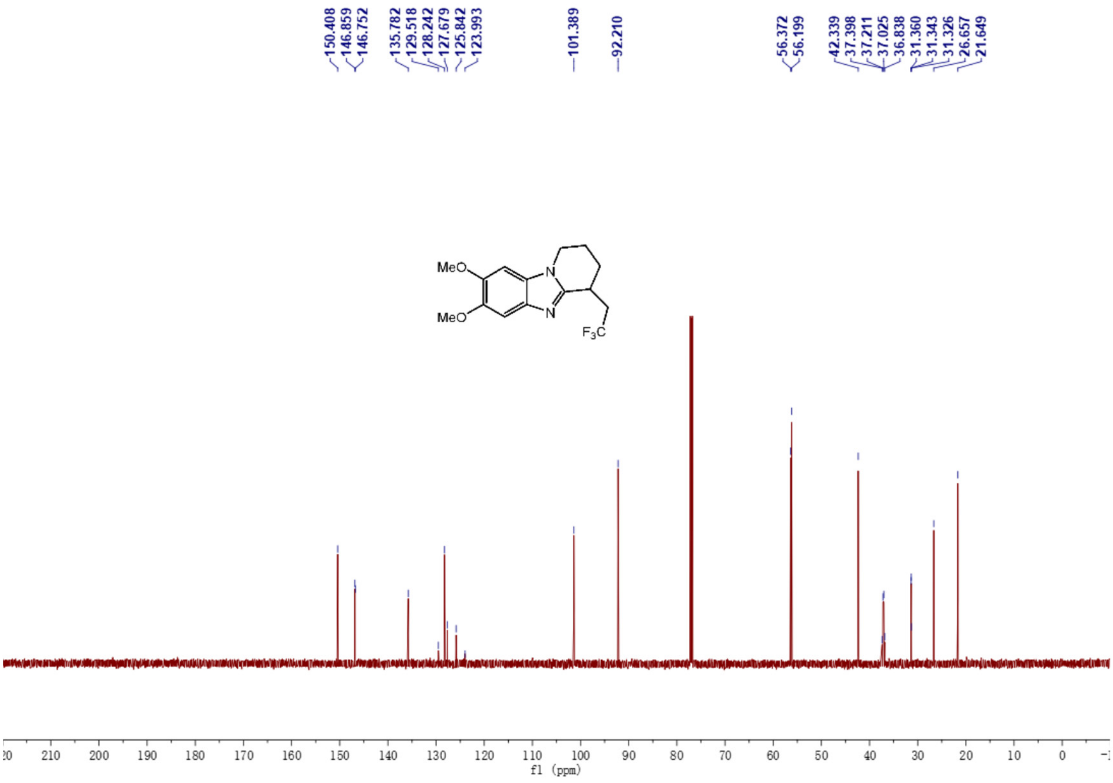

7,8-dimethoxy-4-(2,2,2-trifluoroethyl)-1,2,3,4-tetrahydrobenzo[4,5]imidazo[1,2-a]pyridine (**7d**) : ^{19}F NMR (376 MHz, $CDCl_3$)

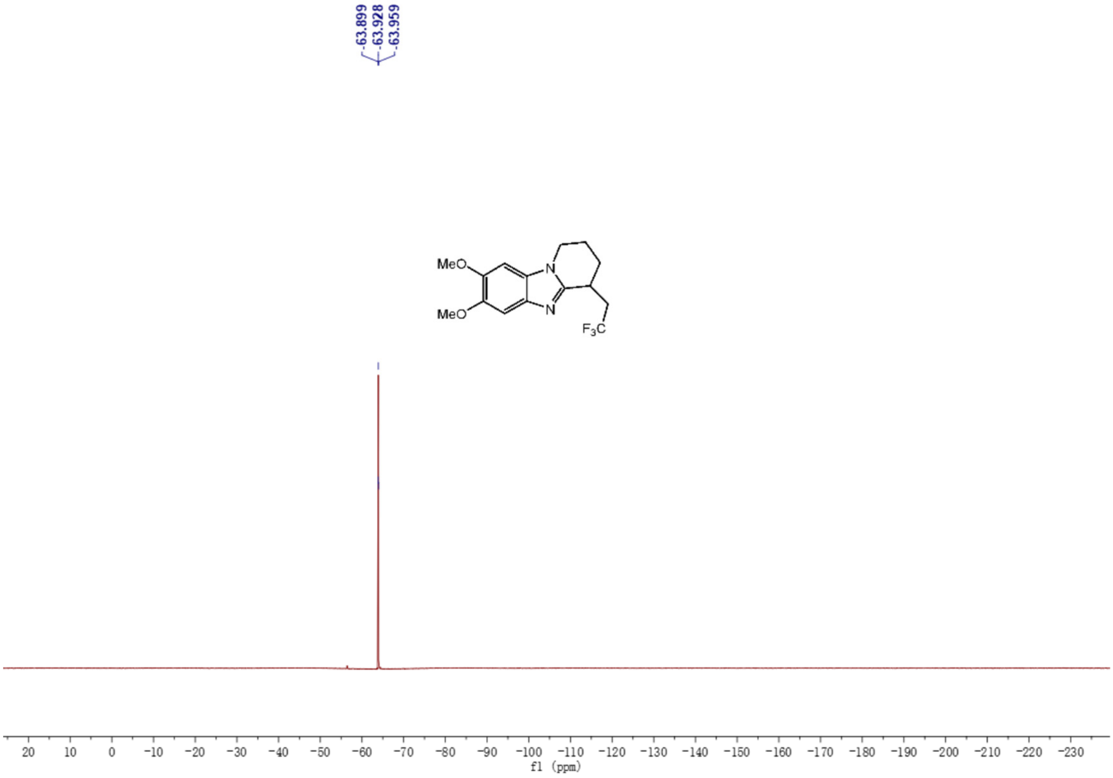

7-fluoro-4-(2,2,2-trifluoroethyl)-1,2,3,4-tetrahydrobenzo[4,5]imidazo[1,2-a]pyridine (**7e**) : ^{1}H NMR (400 MHz, $CDCl_3$)

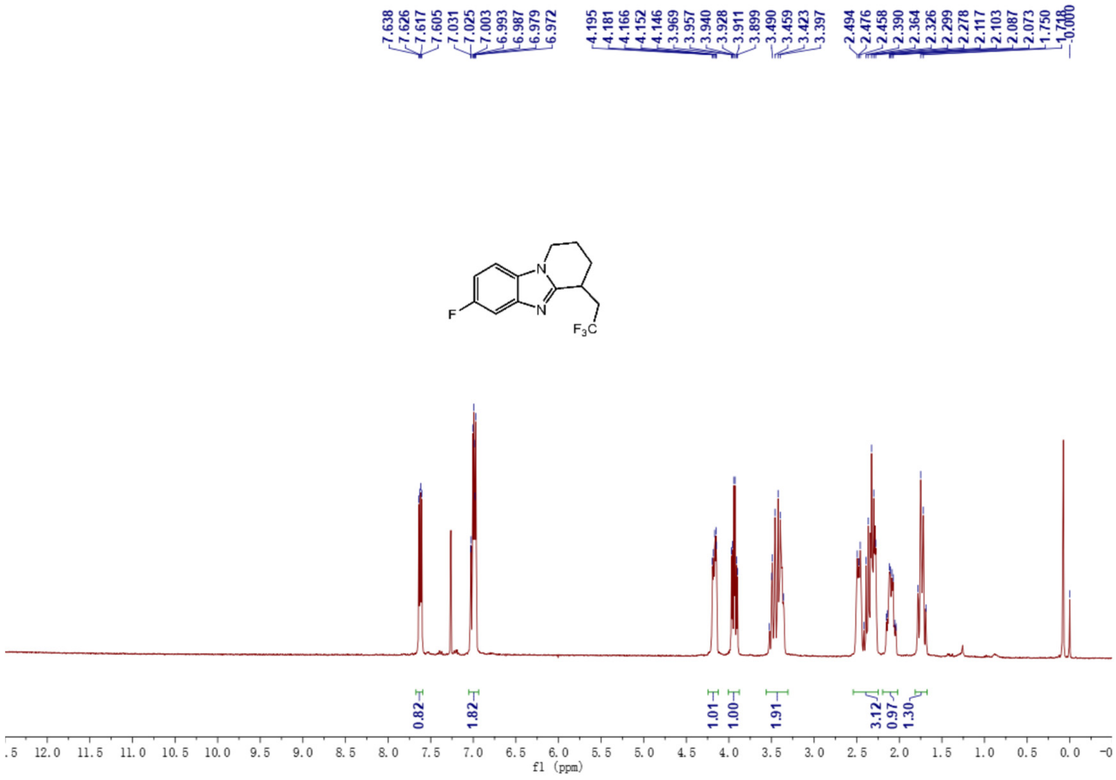

7-fluoro-4-(2,2,2-trifluoroethyl)-1,2,3,4-tetrahydrobenzo[4,5]imidazo[1,2-a]pyridine (**7e**) : ^{13}C NMR (150 MHz, $CDCl_3$)

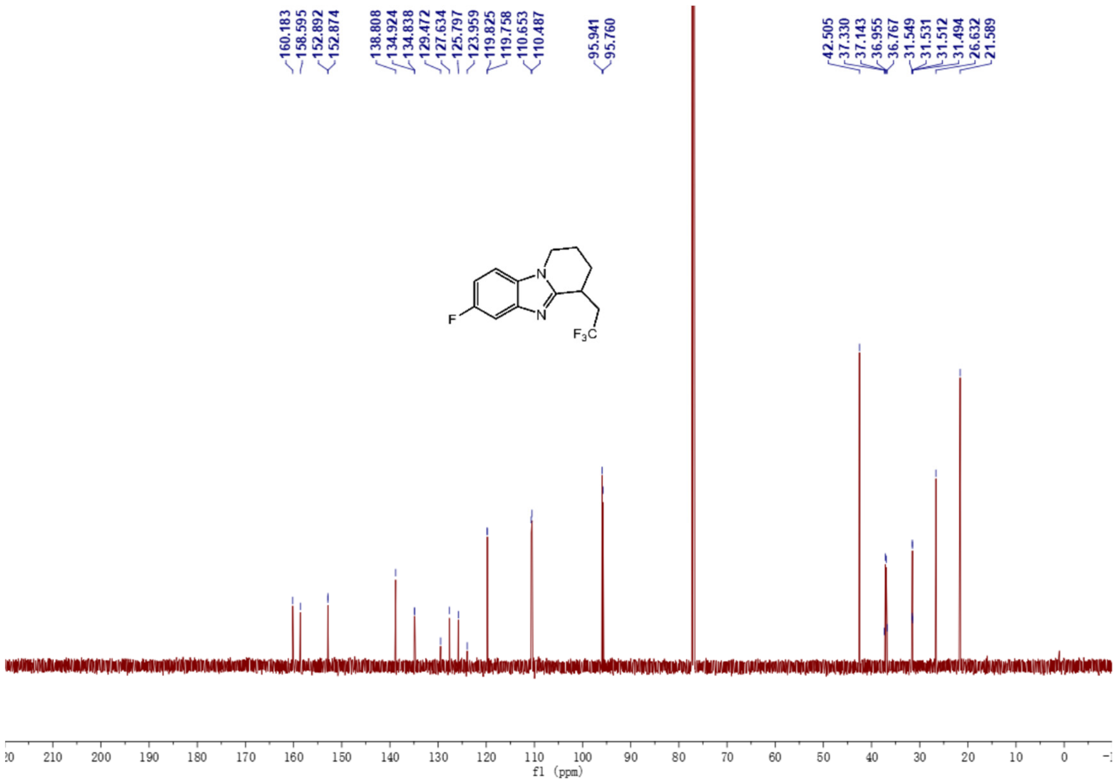

7-fluoro-4-(2,2,2-trifluoroethyl)-1,2,3,4-tetrahydrobenzo[4,5]imidazo[1,2-a]pyridine

(**7e**) : ^{19}F NMR (376 MHz, $CDCl_3$)

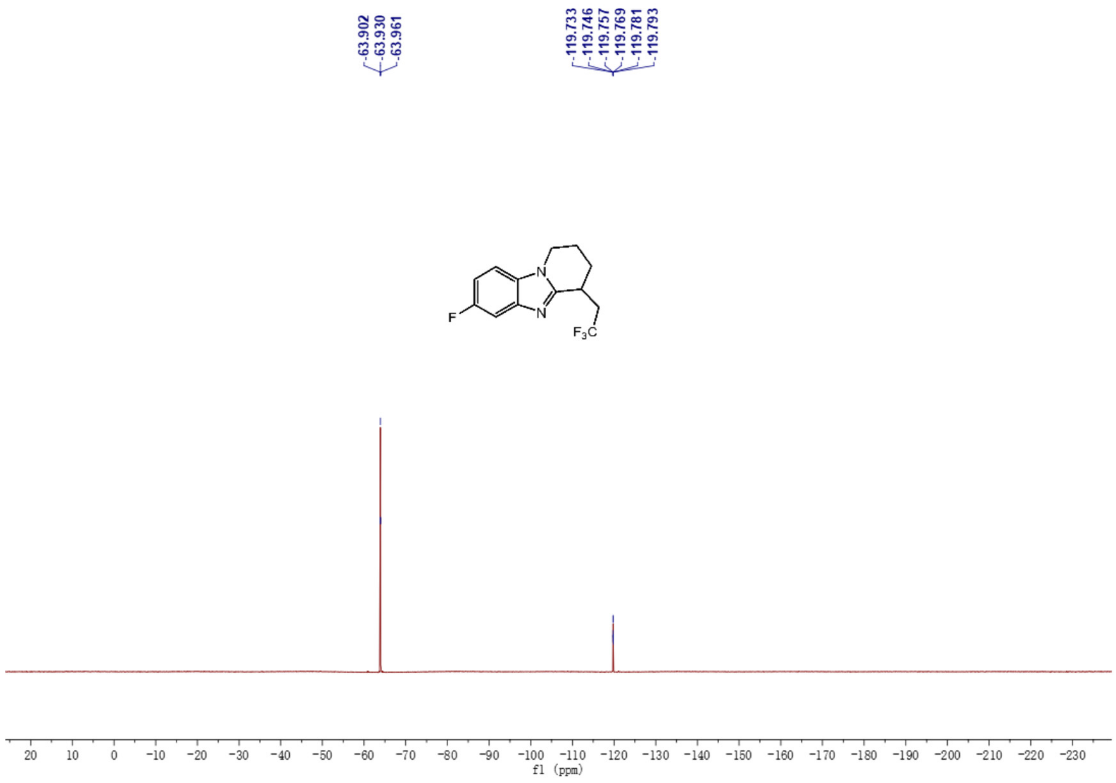

methyl 4-(2,2,2-trifluoroethyl)-1,2,3,4-tetrahydrobenzo[4,5]imidazo[1,2-a]pyridine-8-carboxylate (**7f**) : ^{1}H NMR (400 MHz, $CDCl_3$)

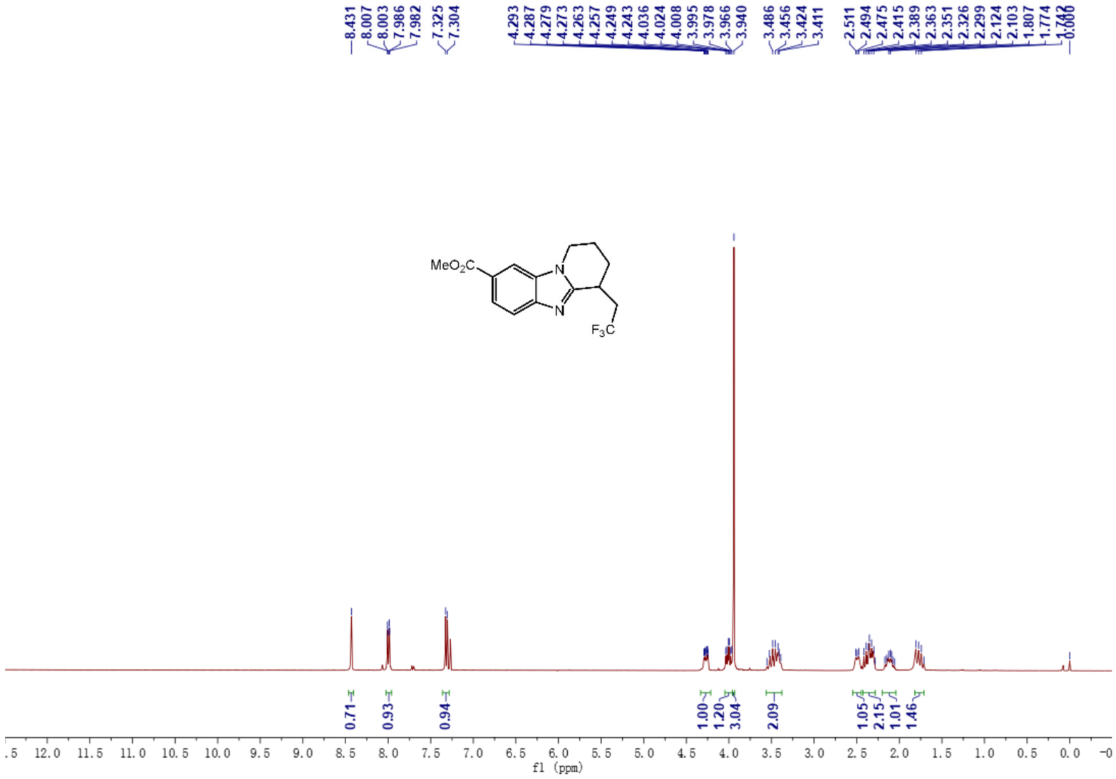

methyl 4-(2,2,2-trifluoroethyl)-1,2,3,4-tetrahydrobenzo[4,5]imidazo[1,2-a]pyridine-8-carboxylate (**7f**) **:** ^{13}C NMR (150 MHz, $CDCl_3$)

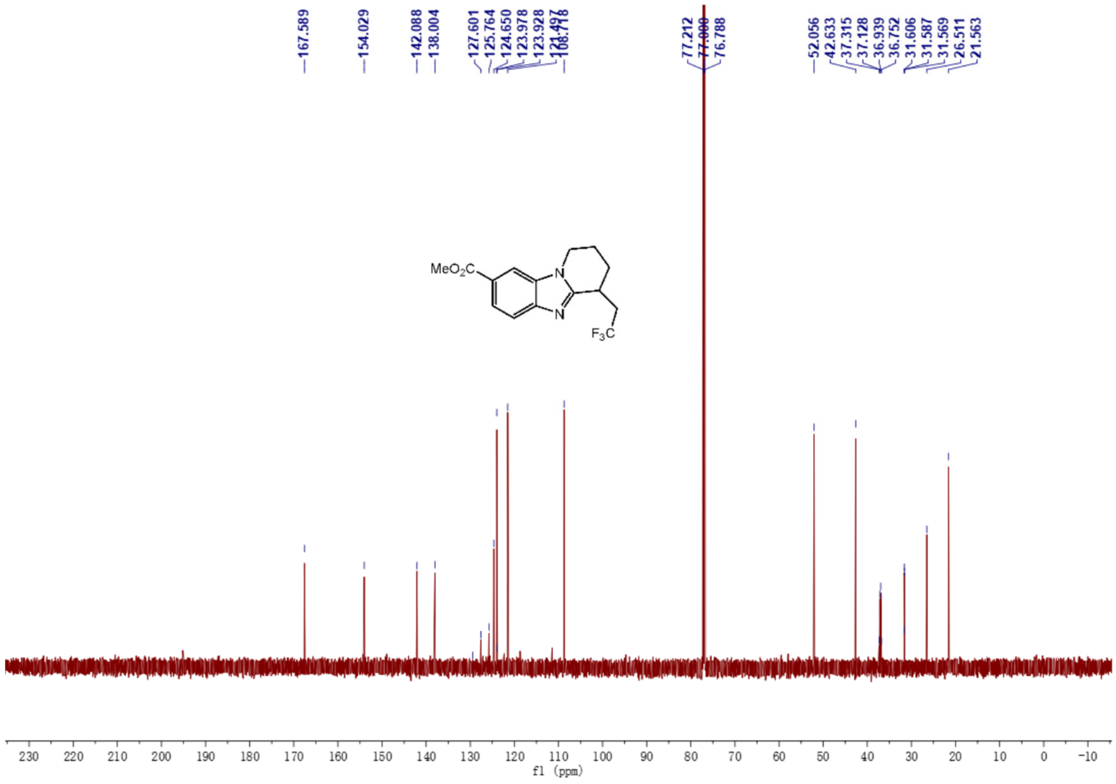

methyl 4-(2,2,2-trifluoroethyl)-1,2,3,4-tetrahydrobenzo[4,5]imidazo[1,2-a]pyridine-8-carboxylate (**7f**) **:** ^{19}F NMR (376 MHz, $CDCl_3$)

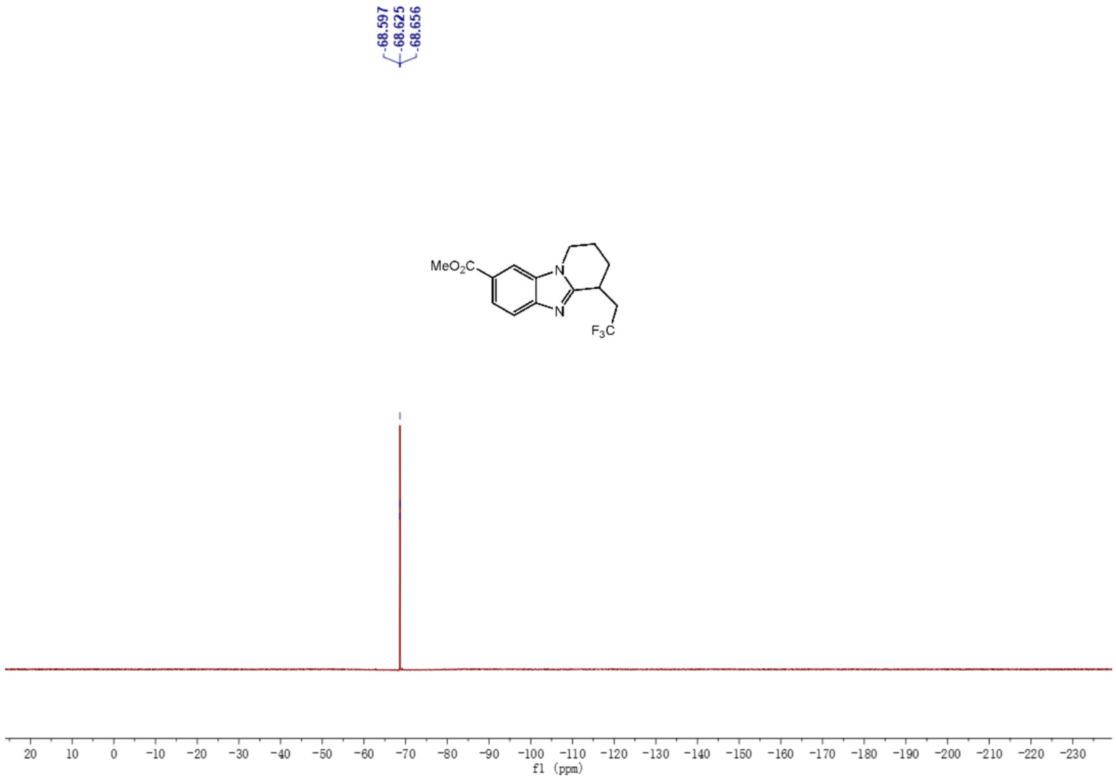

3-(2,2,2-trifluoroethyl)-2,3-dihydro-1H-benzo[*d*]pyrrolo[1,2-*a*]imidazole (**7g**) : ^{1}H NMR (400 MHz, $CDCl_3$)

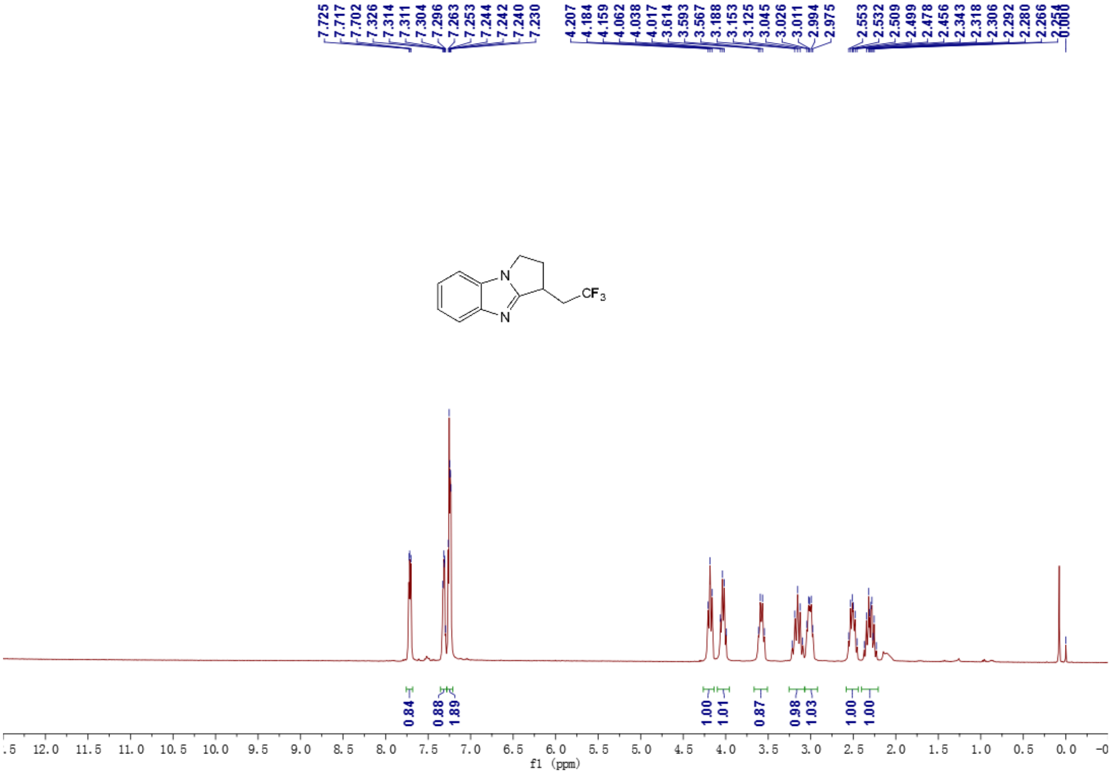

3-(2,2,2-trifluoroethyl)-2,3-dihydro-1H-benzo[*d*]pyrrolo[1,2-*a*]imidazole (**7g**) : ^{13}C NMR (150 MHz, $CDCl_3$)

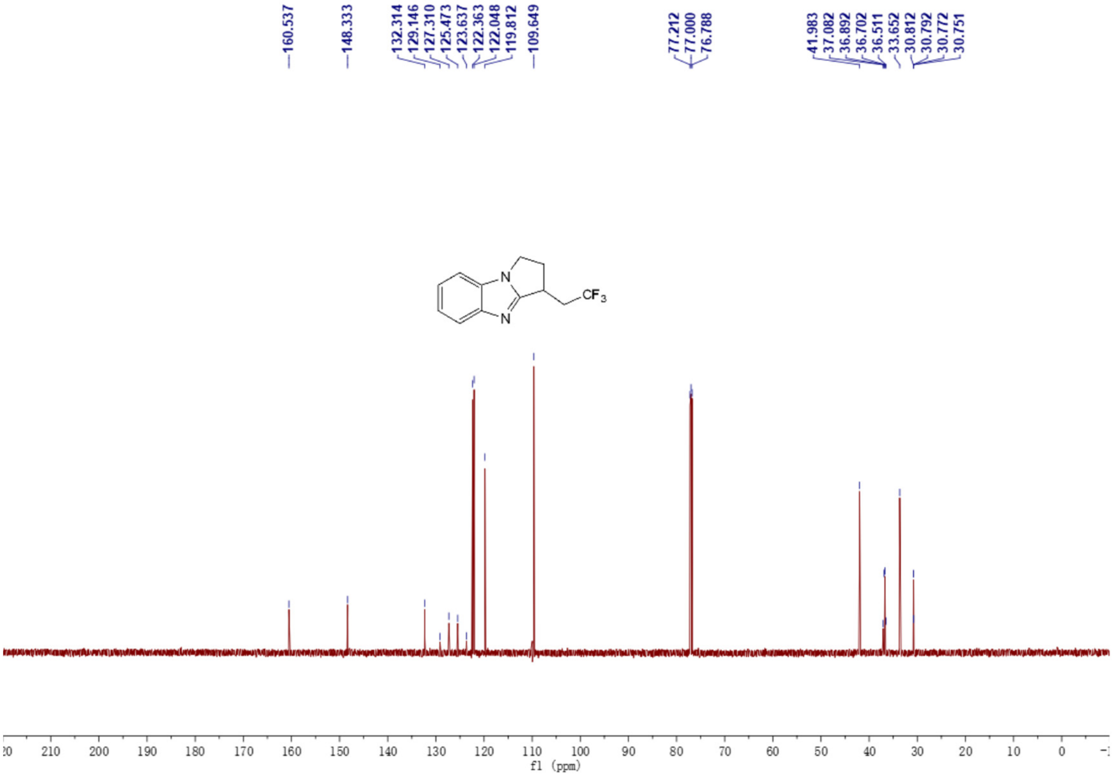

3-(2,2,2-trifluoroethyl)-2,3-dihydro-1H-benzo[*d*]pyrrolo[1,2-*a*]imidazole (**7g**) **:** ^{19}F NMR (376 MHz, $CDCl_3$)

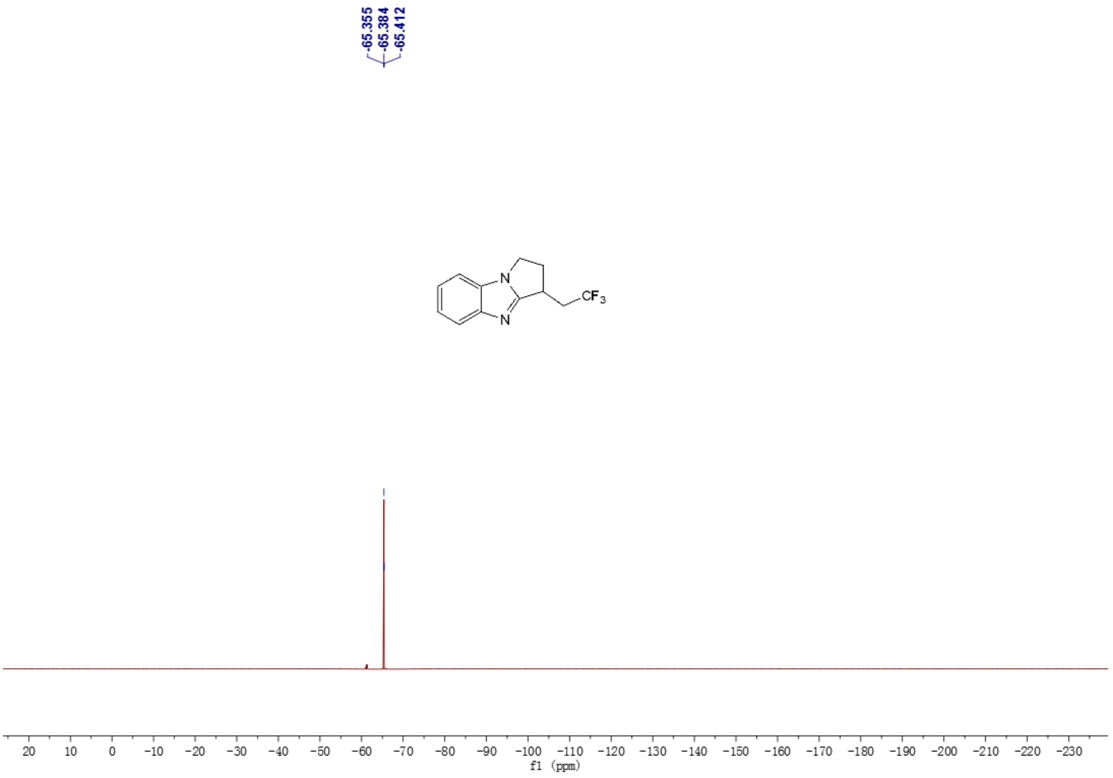

3-methyl-3-(2,2,2-trifluoroethyl)-2,3-dihydro-1H-benzo[*d*]pyrrolo[1,2-*a*]imidazole (**7h**) : ^{1}H NMR (400 MHz, $CDCl_3$)

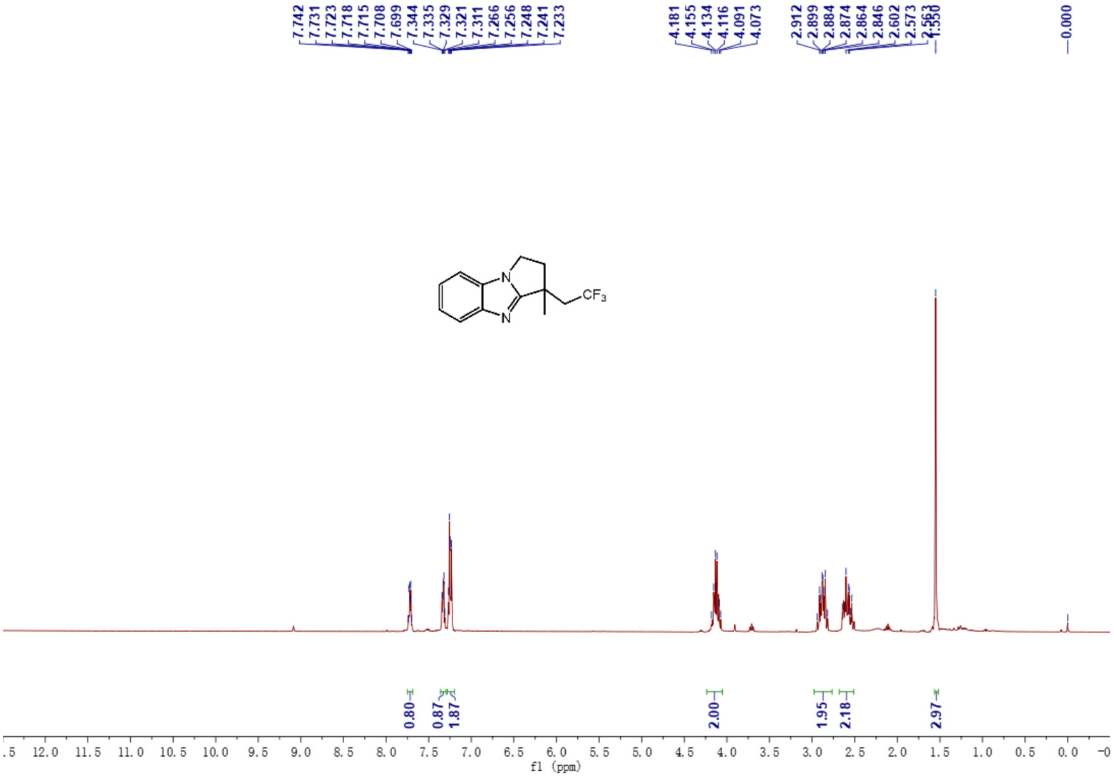

3-methyl-3-(2,2,2-trifluoroethyl)-2,3-dihydro-1H-benzo[*d*]pyrrolo[1,2-*a*]imidazole (**7h**) : ^{13}C NMR (150 MHz, $CDCl_3$)

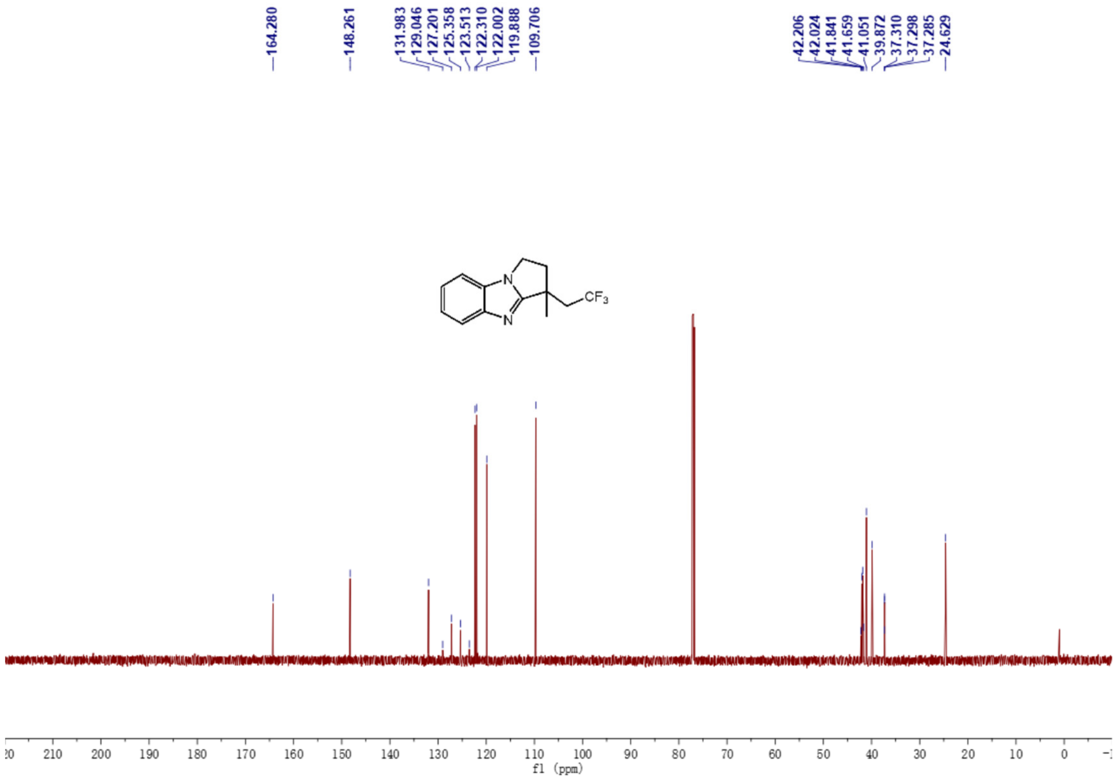

3-methyl-3-(2,2,2-trifluoroethyl)-2,3-dihydro-1H-benzo[*d*]pyrrolo[1,2-*a*]imidazole (**7h**) : ^{19}F NMR (376 MHz, $CDCl_3$)

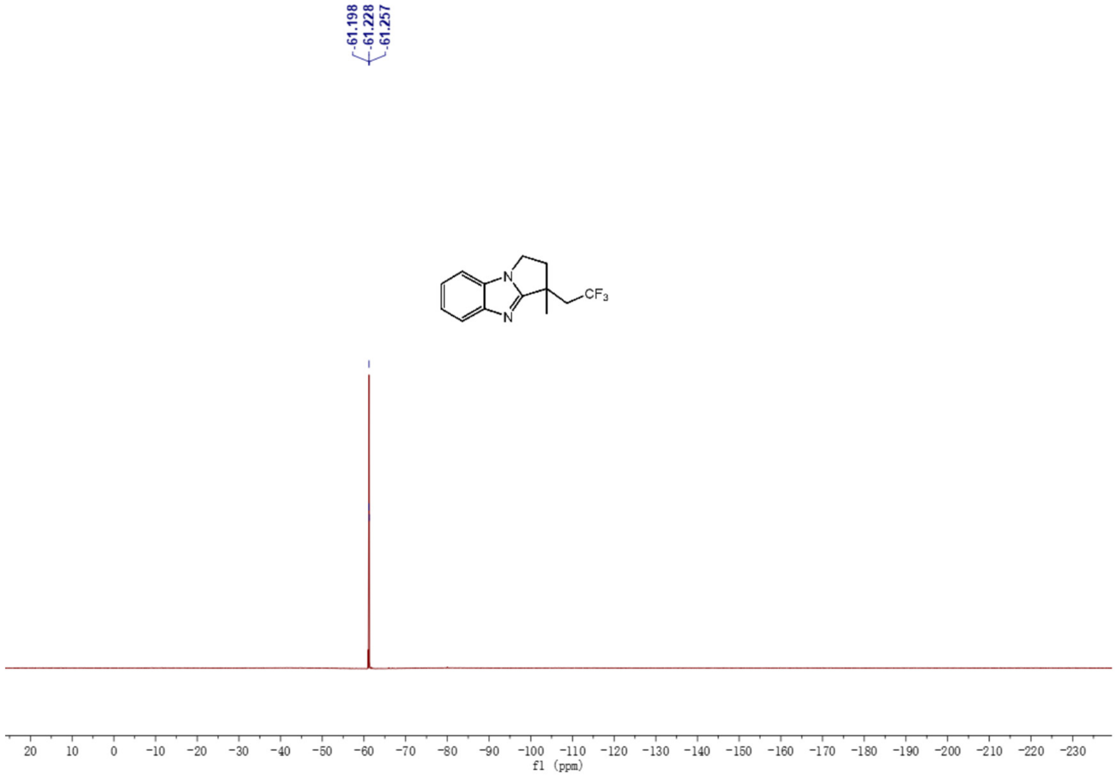

6-(2,2,2-trifluoroethyl)-6,7,8,9-tetrahydroimidazo[1,2-*a*:5,4-*b*']dipyridine (**7i**) : ^{1}H NMR (400 MHz, $CDCl_3$)

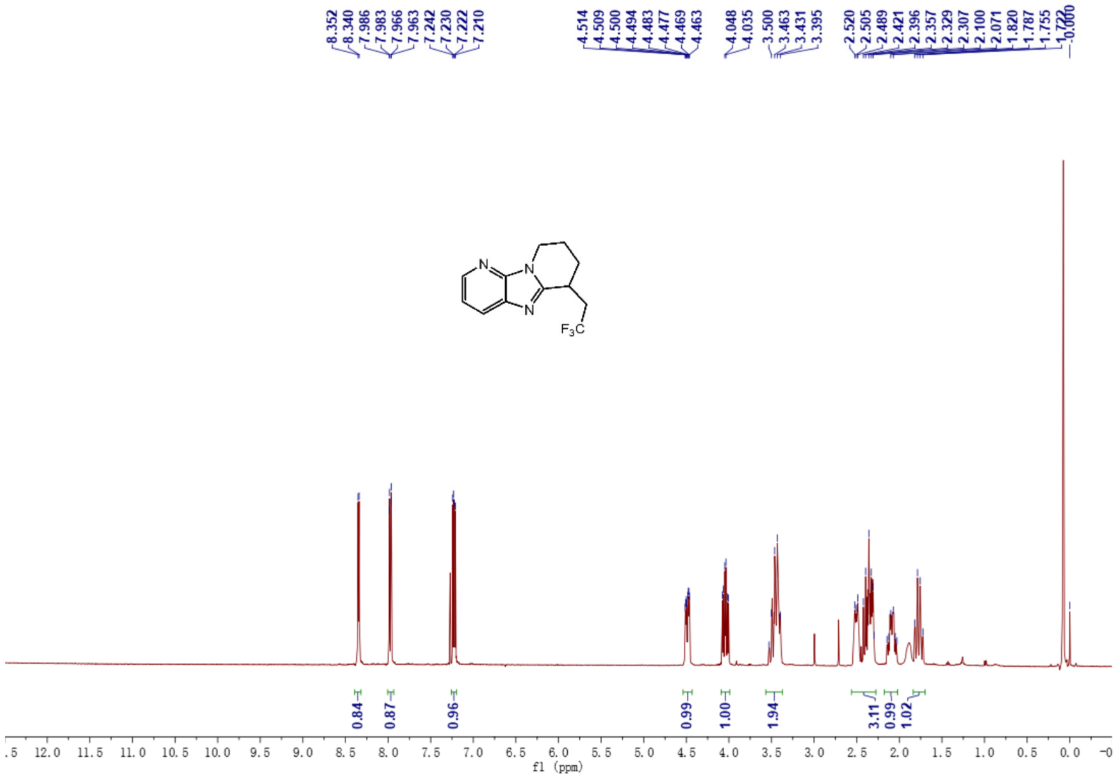

6-(2,2,2-trifluoroethyl)-6,7,8,9-tetrahydroimidazo[1,2-*a*:5,4-*b*']dipyridine (**7i**) **:** ^{13}C NMR (150 MHz, $CDCl_3$)

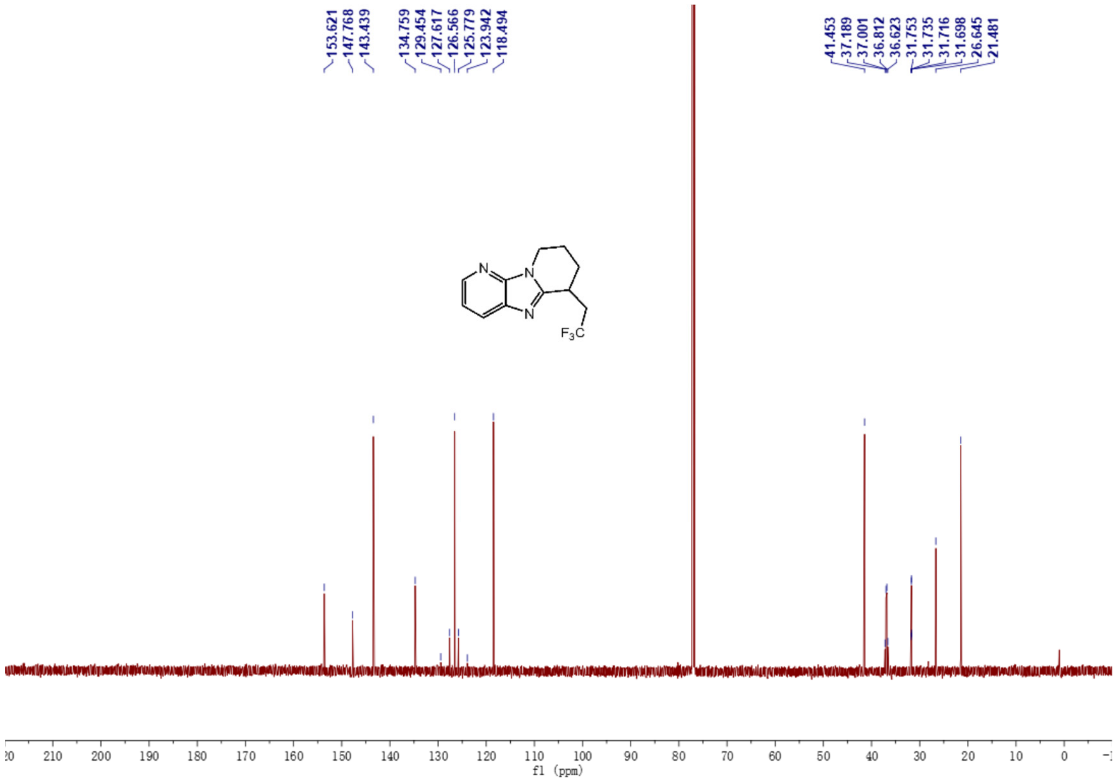

6-(2,2,2-trifluoroethyl)-6,7,8,9-tetrahydroimidazo[1,2-*a*:5,4-*b*']dipyridine (**7i**) **:** ^{19}F NMR (376 MHz, $CDCl_3$)

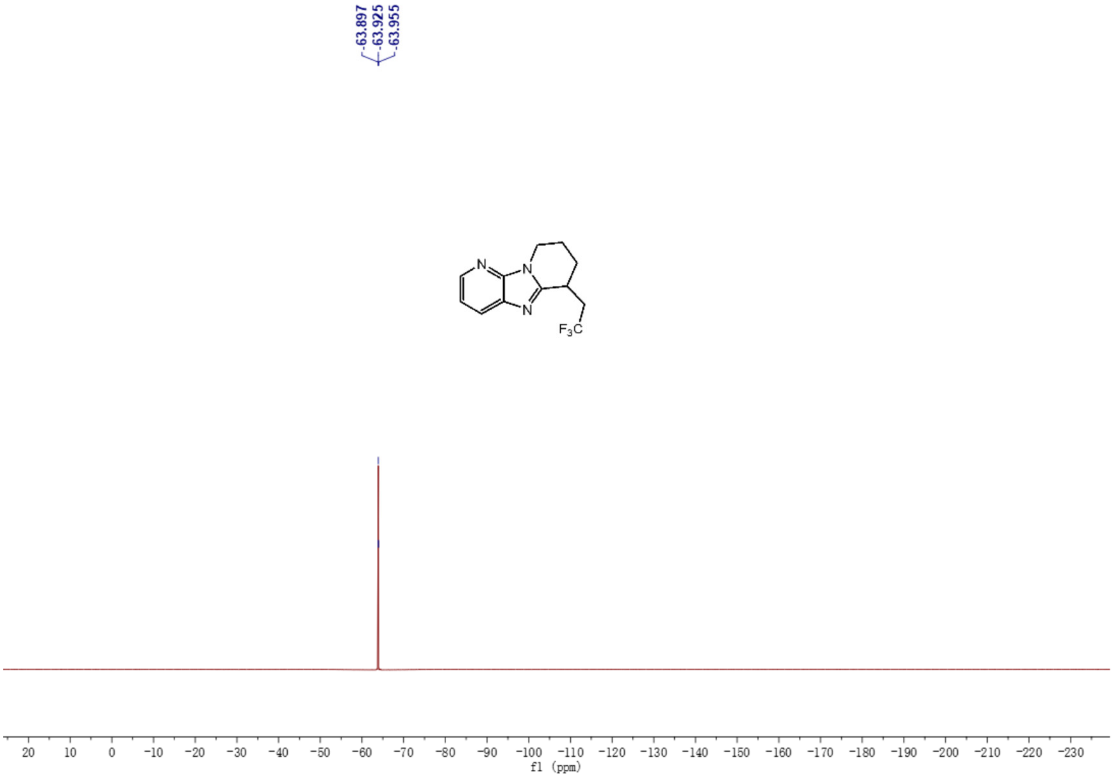

1,3-dimethyl-9-(2,2,2-trifluoroethyl)-6,7,8,9-tetrahydropyrido[2,1-*f*]purine-2,4(1H,3H)-dione (**7j**) : ^{1}H NMR (400 MHz, $CDCl_3$)

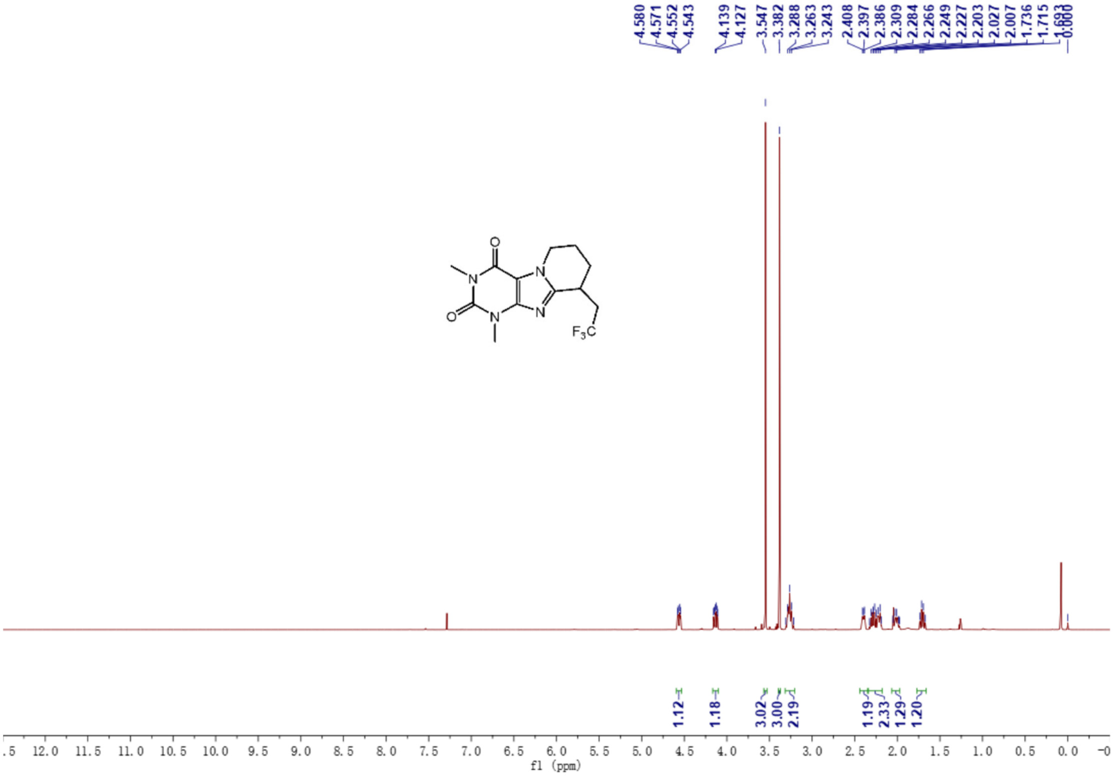

1,3-dimethyl-9-(2,2,2-trifluoroethyl)-6,7,8,9-tetrahydropyrido[2,1-*f*]purine-2,4(1H,3H)-dione (**7j**) : ^{13}C NMR (150 MHz, $CDCl_3$)

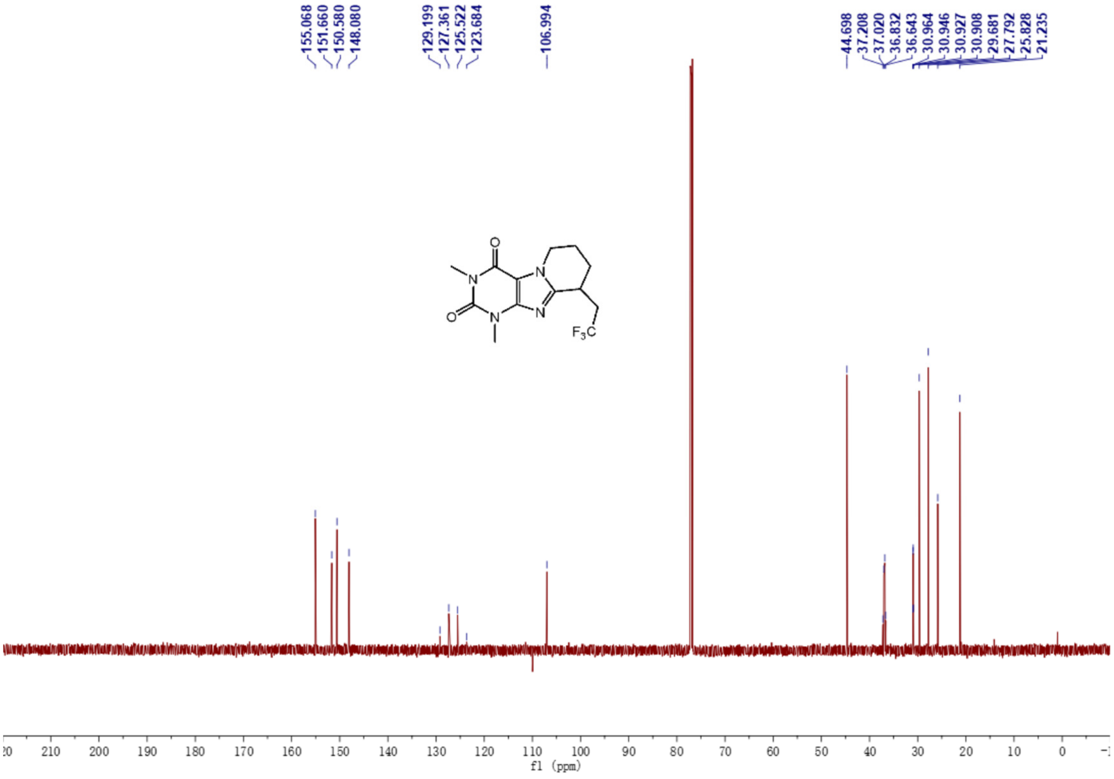

1,3-dimethyl-9-(2,2,2-trifluoroethyl)-6,7,8,9-tetrahydropyrido[2,1-*f*]purine-2,4(1H,3H)-dione (**7j**) : ^{19}F NMR (376 MHz, $CDCl_3$)

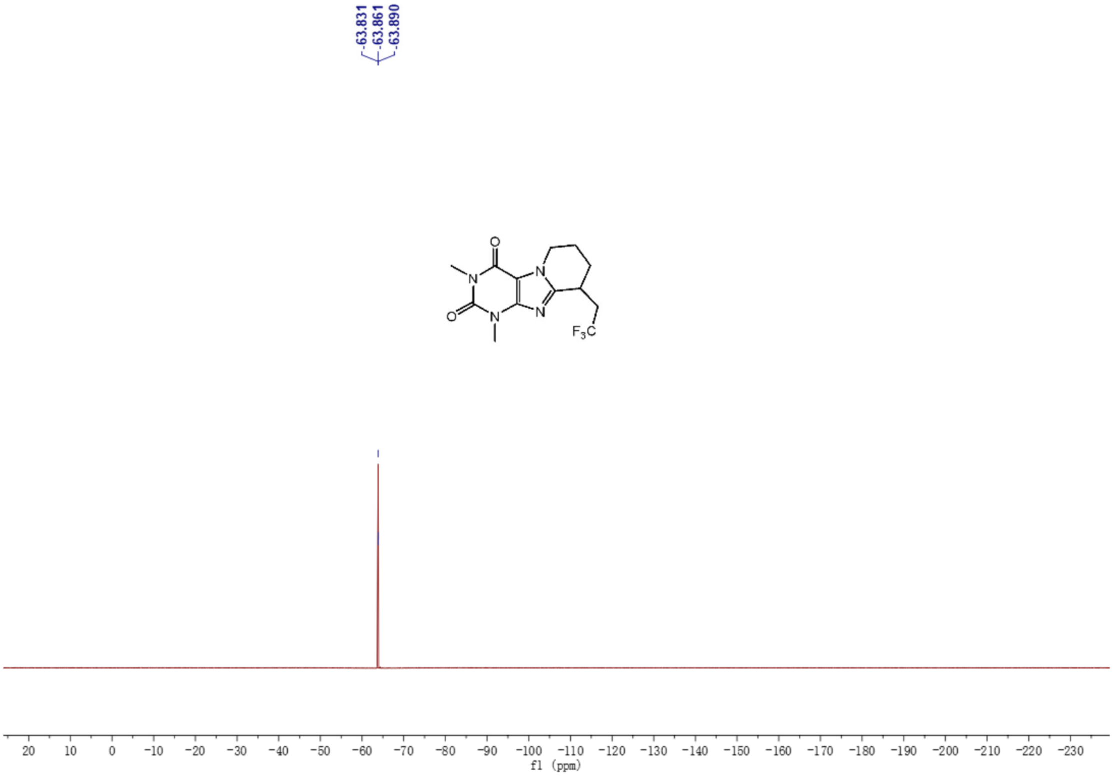

Supplement: Supplementary file 1 [file molecules-27-08389-s001.zip › molecules-2035114-supplementary.pdf]
